# Global Burden of Disease of interstitial lung disease and pulmonary sarcoidosis in adolescents and young adults (1990–2019), and projections for the next 30 years

**DOI:** 10.1186/s40001-025-03141-x

**Published:** 2025-09-26

**Authors:** Xiaoshuang He, Lu Wang, Yu Zhao, Yuanyuan Qu, Wenyan Xin, Lina Xu, Wanyu Li, Chao Wu

**Affiliations:** 1 First Affiliated Hospital of Shihezi University, No. 107, 32 Residential Area, North Second Road, Shihezi, 832000 Xinjiang China; 2 School of Public Health, Xinjiang Medical University, Urumqi, 830011 Xinjiang China

**Keywords:** Global Burden of Disease, Interstitial lung disease, Pulmonary sarcoidosis, Adolescents

## Abstract

**Background:**

Interstitial lung disease (ILD) and pulmonary sarcoidosis are common respiratory diseases that are difficult to diagnose and costly to treat. Adolescents and young adults (AYAs) aged 15–39 years are frequently overlooked in global burden estimates despite their unique epidemiological and societal implications. Comprehensive global burden estimates are lacking. To address this gap, we analysed the results of the Global Burden of Disease Study (GBD) 2019, focusing on Disability-Adjusted Life Years (DALYs).

**Methods:**

Using the GBD 2019 dataset, we calculated age-standardised rate of incidence, mortality, and DALYs and their average annual percentage change from 1990 to 2019 across various factors, including sex, socio-demographic index (SDI), and geographic region. Decomposition analyses explored the impacts of population growth, changing age structures, and epidemiological changes on ILD incidence, DALYs, and mortality in AYAs globally and across different SDI and GBD regions.interstitial lung disease and pulmonary sarcoidosis among adolescents and young adults trends from 2020 to 2049 were predicted using Bayesian Age-Period-Cohort (BAPC).

**Results:**

In 2019, there were 2.6 million cases of ILD and pulmonary sarcoidosis in AYAs, and 3,428 deaths were attributed to these diseases. Age-standardised incidence and DALY rates significantly increased between 1990 and 2019. Females experienced higher rates than males. Standardised DALY rates decreased in high and middle–high SDI countries but increased in low, middle–low, and low-SDI countries. The primary reason was population growth, significantly impacting areas in the lower SDI quintiles.The BAPC model projected a gradual increase in the interstitial lung disease and pulmonary sarcoidosis in adolescents and young adults burden from 2020 to 2049.

**Conclusions:**

This study provides recent epidemiological data on the burden of ILD and pulmonary sarcoidosis in AYAs. These findings highlight the importance of targeted control measures to reduce the burden in this age group.

**Supplementary Information:**

The online version contains supplementary material available at 10.1186/s40001-025-03141-x.

## Introduction

Interstitial lung diseases (ILD), also known as pulmonary fibrosis, are a group of clinically recognised disorders that lead to interstitial scarring, also referred to as pulmonary fibrosis [1, 2]. ILD is classified based on its aetiology and includes connective tissue disease-associated ILD (CTD-ILD), hypersensitivity pneumonitis, drug-induced ILD, post-infectious ILD, and idiopathic interstitial pneumonia. These diseases share similar clinical features but are distinguished by their unique histopathological manifestations and findings. The most common ILDs are idiopathic pulmonary fibrosis (IPF) (> 30% of ILD cases), hypersensitivity pneumonitis (approximately 15% of ILD cases), and connective tissue disease (CTD) (approximately 25% of ILD cases). Other ILDs include pharmacological and post-infectious ILD (e.g., post-covid disease 2019) [3]. Approximately 14% of newly diagnosed patients with IPF have pulmonary hypertension [4], and this figure increases to approximately 86% in patients with pulmonary fibrosis awaiting lung transplantation [5, 6]. The overall prevalence of ILD in the United States is approximately 200 per 100,000 people, and has increased by 19.1% over the past decade, coinciding with an ageing population [7, 8]. Although nodular disease is classified as ILD, it exhibits various pulmonary symptoms, including airway obstruction [9], with an incidence of 126–160 per 100,000 people [10] and as high as 519 per 100,000 people in African American women [11]. Pulmonary sarcoidosis is a disorder characterised by an unexplained inflammatory granulomatous disease that affects a large proportion of the global population, ranging from 2–160 per 100,000 people, and can affect any organ in the body. While some patients with pulmonary sarcoidosis May experience spontaneous remission without significant consequences, others May experience disease progression, leading to fibrocystic structures of the lungs being distorted. This progression is associated with a 12–18% mortality rate over 5 years. Overall, the mortality rate of pulmonary sarcoidosis is approximately 7% at 5 years, with over 60% of deaths attributed to lung involvement [12]. Due to the diagnostic complexity of pulmonary sarcoidosis, the financial burden and stress on patients increase the challenges faced by individuals with this disease [13, 14]. The burden of ILD and pulmonary sarcoidosis is unevenly distributed across age groups, with adolescents and young adults (AYAs) categorised as individuals aged 15–39 years, facing unique challenges. Although the prevalence of ILD and pulmonary sarcoidosis may be lower in adolescents than in older adults, their impact on the loss of healthy life expectancy is disproportionately high [15, 16]. Using data from the Global Burden of Disease (GBD) Study 2019, this study investigated the global impact of ILD and pulmonary sarcoidosis on AYAs between 1990 and 2019. These diseases affect respiratory function, cause various symptoms, and seriously impact lung health. This study aimed to investigate the progression of these diseases in this age group, providing valuable insights into international patterns and differences in geography, age, and sex. This analysis is essential for developing focused public health interventions and improving the well-being of AYAs.

## Materials and methods

### Data source

The Institute for Health Metrics and Evaluation conducted a GBD study that provides a systematic scientific assessment of the prevalence, incidence, and mortality of diseases and injuries. The GBD 2019 study offers a comprehensive epidemiological assessment of the burden of 369 diseases and injuries across 204 countries and territories. Our study focused on patients with ILD and pulmonary sarcoidosis with an age onset of 15–39 years, using data from the Global Health Data Exchange (GHD) at https://ghdx.healthdata.org. Age-, sex-, and region-specific incidence, DALY, and mortality rates for ILD and pulmonary nodular disease in the 15–39-year age group were obtained from the GHD. Patient informed consent was not required to access the use of GHDx data.

#### Definition

Interstitial lung disease (ILD) and pulmonary sarcoidosis are long-term respiratory conditions that impact lung function and oxygen absorption as a result of scarring and inflammation. The ICD-10 codes for these conditions are D86 to D86.2 and D86.9, and J84 to J84.9, respectively. In contrast, the ICD-9 codes range from 135 to 135.9 and 515 to 516.9. It is important to note that this classification specifically does not include lung disorders caused by external factors, drug-induced interstitial lung issues, systemic or rheumatic diseases, or any other conditions leading to ILD.

### Statistical analyses

Age-standardised rates and their corresponding 95% confidence intervals (CIs) were calculated using the world standard population, as shown in the GBD Study 2019 [16]. Age-standardised rates for each indicator in the population aged 15–39 years were calculated using direct standardisation based on the world population [17]. The rates are expressed per 100,000 people. To assess the trends in the incidence, DALYs, and mortality associated with ILD and pulmonary sarcoidosis in AYAs, we calculated the mean annual percentage changes and corresponding 95% CIs using joint-point regression [18]. Comparisons were made based on sex and socio-demographic indices (SDI) divided into five groups. Detailed information on SDI is available in the Supplementary Material. Least-squares regression and generalised additive models were used to explore potential linear or non-linear associations between SDI and age-standardised incidence rate(ASIR), age-standardised mortality rates(ASMR), and age-standardised DALY rate. Pearson's correlation analyses were used to determine the strength and direction of the relationships between the covariates. Decomposition analyses were used to calculate the impact of population growth, age-structural changes, and epidemiological changes on the burden of ILD and pulmonary sarcoidosis in young sex-specific populations, with a special focus on global, SDI, and GBD regions [19]. The Bayesian Age-Period-Cohort (BAPC) model integrates age, period, and cohort effects to analyze and predict disease trends, while accounting for population changes. By considering the complex interplays of population changes over time, the model provides a nuanced approach to forecasting disease trends. Compared to traditional models, the BAPC model is better equipped to handle the uncertainty and complexity of the data. This study utilized the BAPC model to predict the development trends of interstitial lung disease and pulmonary sarcoidosis in adolescents and young adults. over the next 30 years, aiming to provide guidance for the prevention and treatment of interstitial lung disease and pulmonary sarcoidosis in adolescents and young adults.All statistical analyses and graphical representations were generated using R version 4.2.1. The R program V.3.6.3 was used for derivative analyses and plotting, with the significance level set at P < 0.05.

## Results

### Global distribution of ILD and pulmonary sarcoidosis in AYas in 2019

In 2019, there were 2.6 million (95% CI: 1.5–4.4) cases of ILD and pulmonary sarcoidosis in AYAs, with 3,428 (2,759–4,266) deaths from these conditions in this age group.

The global age-standardised incidence, DALY, and mortality rates per 100,000 population were 87.58 (95% CI 51.66–140.35), 8.02 (95% CI 6.45–9.96), and 0.11 (95% CI 0.09–0.14), respectively. When grouped based on SDI, low-index countries had the highest age-standardised prevalence rates (100.28, 95% CI 58.28–162.21), while middle-index countries had the lowest age-standardised prevalence rates (72.64, 95% CI 42.27–117.46). Similarly, age-standardised DALY rates were highest in low- and middle-index countries (10.64, 95% CI 7.84–14.57) and lowest in high- and middle-index countries (6.01, 95% CI 4.76–7.46). ASMR were also highest in low- and middle-index countries (0.16, 95% CI 0.11–0.23) and lowest in high- and middle-index countries (0.08, 95% CI 0.06–0.09). Oceania and Andean Latin America showed the greatest burden of ILD and pulmonary sarcoidosis among AYAs, with Belize having the highest age-standardised incidence, DALY, and mortality rates (Tables 1, 2, 3, 4, 5, 6, Appendix Tables 1–12).

**Table 1:** ASIR of interstitial lung disease and pulmonary sarcoidosis in adolescents and young adultsr in 1990 and 2019 for female and all locations, with AAPC from 2009 and 2019

Location	Numbers in 1990	Numbers in 2019	Age-standardized rates in 1990 (95% CI)	Age standardized rates in 2019 (95% CI)	AAPC, % (95% CI)	P
Global	853,342.44 (494,195.54,1,394,724.82)	1,308,087.88 (771,632.08,2,109,747.89)	82.77 (48.09,135.33)	87.77 (51.72,141.67)	0.23 (0.09,0.37)	0.001
High SDI	169,649.88 (98,843.00,276,866.58)	169,043.69 (101,903.16,264,254.68)	102.43 (59.56,167.22)	96.53 (58.00,150.84)	− 0.15 (− 0.28,− 0.03)	0.016
High-middle SDI	219,642.98 (127,896.58,359,275.03)	234,853.74 (138,399.84,379,835.70)	92.26 (53.72,150.92)	84.04 (49.26,136.01)	− 0.32 (− 0.36,− 0.27)	0
Middle SDI	211,091.62 (120,205.57,348,019.00)	349,448.73 (203,717.64,567,688.57)	61.81 (35.40,101.92)	72.74 (42.29,118.36)	0.57 (0.52,0.61)	0
Low-middle SDI	168,880.10 (97,488.82,274,521.05)	342,372.92 (199,628.54,553,919.87)	84.45 (49.09,137.35)	96.95 (56.66,156.98)	0.51 (0.39,0.63)	0
Low SDI	83,767.75 (48,282.17,135,356.98)	211,728.09 (121,631.47,342,044.24)	94.02 (54.55,151.97)	102.70 (59.40,166.08)	0.33 (0.22,0.45)	0
Andean Latin America	4832.05 (2809.25,7810.22)	10,644.21 (6363.93,17,054.63)	68.01 (39.83,109.96)	83.59 (49.99,133.97)	0.70 (0.63,0.78)	0
Australasia	2878.04 (1639.73,4729.51)	3330.45 (1921.44,5434.97)	68.90 (39.20,113.21)	64.21 (36.87,104.93)	− 0.23 (− 0.26,− 0.21)	0
Caribbean	3557.52 (2010.92,5885.96)	5214.06 (3012.04,8666.10)	50.87 (28.92,84.26)	56.97 (32.90,94.79)	0.38 (0.34,0.42)	0
Central Asia	15,595.69 (9228.39,24,842.33)	22,240.02 (13,056.76,36,158.31)	114.28 (67.83,182.73)	114.67 (67.23,186.91)	0.01 (− 0.02,0.04)	0.532
Central Europe	39,843.54 (23,451.91,64,829.14)	34,922.45 (21,311.13,55,033.97)	164.71 (96.67,267.26)	173.50 (105.38,272.68)	0.20 (0.12,0.27)	0
Central Latin America	17,786.21 (10,035.60,29,793.81)	23,846.96 (13,841.65,38,908.68)	56.85 (32.26,95.48)	46.44 (26.95,75.75)	− 0.70 (− 0.76,− 0.64)	0
Central Sub-Saharan Africa	9118.96 (5176.89,14,721.21)	24,356.22 (13,944.45,39,266.54)	95.18 (54.42,153.83)	101.75 (58.71,164.10)	0.24 (0.20,0.27)	0
East Asia	108,313.38 (60,124.68,184,009.79)	85,575.53 (49,139.08,140,652.49)	41.36 (23.04,70.13)	30.58 (17.40,50.54)	− 1.06 (− 1.19,− 0.92)	0
Eastern Europe	93,740.17 (55,242.32,152,020.42)	85,567.29 (50,032.53,140,344.09)	201.28 (118.23,326.50)	210.11 (122.12,343.76)	0.16 (0.12,0.21)	0
Eastern Sub-Saharan Africa	30,134.67 (17,262.29,48,769.58)	74,578.92 (42,556.80,121,245.33)	91.69 (52.92,148.59)	96.11 (55.23,156.55)	0.18 (0.11,0.25)	0
High-income Asia Pacific	28,445.29 (16,137.09,46,918.37)	19,172.51 (11,132.82,31,132.64)	85.22 (48.34,140.35)	71.68 (41.44,116.05)	− 0.58 (− 0.66,− 0.49)	0
High-income North America	115,904.48 (67,797.45,189,709.92)	106,022.24 (64,393.29,164,607.13)	190.55 (111.18,312.04)	166.01 (100.69,257.49)	− 0.42 (−0.57,−0.27)	0
North Africa and Middle East	81,219.45 (46,972.25,130,861.92)	191,696.72 (112,208.12,309,624.23)	135.62 (78.84,218.81)	151.84 (88.79,245.26)	0.40 (0.35,0.45)	0
Oceania	1280.87 (741.56,2057.47)	3194.71 (1883.10,5076.08)	108.63 (63.18,174.42)	123.71 (73.03,196.53)	0.44 (0.36,0.51)	0
South Asia	186,532.19 (108,424.17,301,908.97)	431,791.23 (253,073.16,694,503.14)	96.71 (56.52,156.58)	118.27 (69.44,190.26)	0.73 (0.59,0.86)	0
Southeast Asia	22,990.12 (12,624.71,38,417.10)	35,190.10 (19,538.03,58,736.00)	24.48 (13.52,41.04)	25.77 (14.29,42.99)	0.18 (0.16,0.21)	0
Southern Latin America	5955.25 (3422.87,9770.83)	7410.26 (4231.76,11,941.62)	62.94 (36.23,103.27)	56.58 (32.24,91.24)	− 0.25 (− 0.60,0.10)	0.163
Southern Sub-Saharan Africa	16,258.81 (9472.30,26,055.32)	26,831.37 (15,648.12,43,278.15)	156.97 (91.98,252.24)	157.92 (92.08,255.28)	0.06 (− 0.03,0.14)	0.212
Tropical Latin America	24,130.83 (13,759.35,39,987.29)	14,322.87 (8325.72,23,082.08)	79.16 (45.29,131.39)	30.32 (17.55,48.84)	− 3.27 (− 3.41,− 3.13)	0
Western Europe	11,319.65 (6368.31,18,468.39)	10,756.46 (6109.84,17,666.73)	15.54 (8.72,25.38)	15.56 (8.78,25.56)	0.05 (− 0.02,0.12)	0.178
Western Sub-Saharan Africa	33,505.27 (19,181.32,54,129.15)	91,423.29 (52,362.84,147,645.34)	100.65 (58.02,163.17)	106.53 (61.38,172.39)	0.24 (0.19,0.29)	0

ASIR,age-standardised incidence rate;APPC,average annual percentage change

**Table 2:** DALYs of interstitial lung disease and pulmonary sarcoidosis in adolescents and young adultsr in 1990 and 2019 for female and all locations, with AAPC from 2009 and 2019

Location	Numbers in 1990	Numbers in 2019	Age-standardized rates in 1990 (95% CI)	Age standardized rates in 2019 (95% CI)	AAPC, % (95% CI)	P
Global	68,897.02 (50,581.11,90,755.16)	108,031.76 (81,252.10,140,896.91)	6.66 (4.89,8.77)	7.26 (5.46,9.47)	0.31 (0.12,0.50)	0.001
High SDI	15,800.22 (11,983.75,21,167.65)	15,997.80 (12,289.88,21,074.17)	9.57 (7.26,12.84)	9.26 (7.11,12.24)	− 0.10 (− 0.22,0.01)	0.072
High-middle SDI	14,303.10 (10,395.75,18,810.29)	15,295.77 (11,358.71,20,189.22)	6.01 (4.37,7.90)	5.66 (4.19,7.47)	− 0.17 (− 0.33,− 0.01)	0.034
Middle SDI	18,179.49 (13,462.91,25,493.88)	30,331.38 (22,981.14,40,687.14)	5.28 (3.90,7.40)	6.35 (4.81,8.52)	0.68 (0.47,0.89)	0
Low-middle SDI	14,812.29 (9376.73,22,059.34)	31,465.37 (21,030.75,45,178.71)	7.47 (4.72,11.08)	8.93 (5.97,12.83)	0.69 (0.44,0.95)	0
Low SDI	5755.16 (3115.48,9286.95)	14,843.69 (9106.08,22,259.30)	6.63 (3.59,10.61)	7.34 (4.52,10.96)	0.43 (0.17,0.70)	0.001
Andean Latin America	1560.80 (888.27,2617.16)	2653.64 (1648.06,3958.45)	20.86 (11.97,34.91)	20.79 (12.91,31.02)	0.01 (− 0.28,0.30)	0.945
Australasia	125.00 (81.20,200.78)	275.58 (146.52,429.65)	2.98 (1.93,4.78)	5.23 (2.78,8.15)	1.95 (1.75,2.15)	0
Caribbean	472.75 (339.24,655.91)	982.59 (600.17,1520.58)	6.75 (4.85,9.36)	10.70 (6.54,16.57)	1.60 (1.35,1.85)	0
Central Asia	2332.12 (1411.66,3264.19)	2513.87 (1698.59,3664.72)	16.94 (10.29,23.66)	12.97 (8.76,18.97)	− 0.90 (− 1.06,− 0.74)	0
Central Europe	2135.53 (1417.96,2871.52)	1385.47 (1005.21,1963.12)	8.96 (5.92,12.07)	7.10 (5.14,10.06)	− 0.78 (− 0.87,− 0.69)	0
Central Latin America	3219.44 (2358.83,4728.05)	6589.37 (4098.51,9018.63)	9.91 (7.24,14.41)	12.80 (7.96,17.52)	0.91 (0.71,1.10)	0
Central Sub-Saharan Africa	518.56 (170.93,1171.61)	1260.64 (553.83,2525.24)	5.50 (1.80,12.40)	5.31 (2.33,10.64)	− 0.14 (− 0.35,0.08)	0.217
East Asia	7141.88 (4700.98,12,435.11)	5807.00 (3851.78,8562.01)	2.71 (1.78,4.72)	2.12 (1.40,3.13)	− 0.87 (− 1.06,− 0.69)	0
Eastern Europe	3479.01 (2257.03,5141.92)	2467.73 (1561.11,4209.70)	7.61 (4.94,11.28)	6.28 (3.96,10.93)	− 0.66 (− 0.88,− 0.44)	0
Eastern Sub-Saharan Africa	1551.93 (584.31,2976.72)	4010.49 (1886.36,6939.04)	4.78 (1.80,9.13)	5.19 (2.45,8.96)	0.28 (0.07,0.49)	0.009
High-income Asia Pacific	2306.38 (1593.13,3405.58)	1704.14 (1193.52,2777.99)	6.89 (4.76,10.18)	6.28 (4.41,10.30)	− 0.34 (− 0.53,− 0.15)	0
High-income North America	9770.44 (7131.40,12,915.44)	8441.23 (6525.38,11,703.03)	16.10 (11.74,21.32)	13.31 (10.27,18.47)	− 0.63 (− 0.76,− 0.51)	0
North Africa and Middle East	4153.07 (2698.58,6494.67)	9980.63 (6942.75,15,068.30)	6.76 (4.41,10.57)	7.95 (5.53,12.01)	0.56 (0.44,0.69)	0
Oceania	382.69 (202.13,745.34)	899.70 (452.77,1787.62)	31.96 (16.94,61.68)	34.64 (17.49,68.61)	0.27 (0.07,0.47)	0.007
South Asia	17,613.49 (10,368.40,26,901.34)	40,791.97 (26,411.76,60,281.59)	9.25 (5.46,14.05)	11.21 (7.25,16.55)	0.73 (0.41,1.04)	0
Southeast Asia	2018.55 (1337.32,3217.71)	3570.99 (2350.06,5787.32)	2.21 (1.46,3.54)	2.60 (1.71,4.20)	0.55 (0.45,0.65)	0
Southern Latin America	1196.22 (764.68,1739.05)	1608.65 (995.12,2318.64)	12.59 (8.05,18.29)	12.35 (7.63,17.83)	− 0.07 (− 0.35,0.20)	0.595
Southern Sub-Saharan Africa	1481.60 (712.82,2251.47)	1603.52 (858.04,3034.99)	14.47 (6.94,21.96)	9.37 (5.03,17.70)	− 1.50 (− 2.01,− 1.00)	0
Tropical Latin America	2790.15 (1910.88,3874.58)	4236.28 (2749.55,5915.45)	8.91 (6.11,12.35)	9.16 (5.92,12.77)	0.08 (− 0.30,0.47)	0.682
Western Europe	3497.86 (2512.39,5121.30)	4227.67 (2682.28,5375.88)	4.81 (3.46,7.06)	6.21 (3.92,7.89)	0.88 (0.78,0.99)	0
Western Sub-Saharan Africa	1149.53 (652.64,1747.89)	3020.60 (1855.45,4539.57)	3.49 (1.98,5.30)	3.52 (2.17,5.28)	0.02 (−0.10,0.14)	0.717

APPC,average annual percentage change

**Table 3:** ASMR of interstitial lung disease and pulmonary sarcoidosis in adolescents and young adultsr in 1990 and 2019 for female and all locations, with AAPC from 2009 and 2019

location	Numbers in 1990	Numbers in 2019	Age-standardized rates in 1990 (95% CI)	Age standardized rates in 2019 (95% CI)	AAPC, % (95% CI)	P
Global	927.76 (641.49,1237.68)	1501.61 (1082.68,2055.09)	0.09 (0.06,0.12)	0.10 (0.07,0.14)	0.41 (0.19,0.63)	0
High SDI	208.67 (149.77,289.71)	215.47 (159.53,293.74)	0.13 (0.09,0.18)	0.12 (0.09,0.17)	− 0.06 (− 0.21,0.08)	0.391
High-middle SDI	179.67 (123.63,238.56)	197.44 (139.98,263.57)	0.08 (0.05,0.10)	0.07 (0.05,0.10)	− 0.10 (− 0.34,0.14)	0.415
Middle SDI	250.44 (177.20,367.09)	429.69 (316.46,601.08)	0.07 (0.05,0.11)	0.09 (0.07,0.13)	0.72 (0.48,0.95)	0
Low-middle SDI	210.50 (120.69,332.04)	456.54 (286.79,684.76)	0.11 (0.06,0.17)	0.13 (0.08,0.20)	0.70 (0.34,1.07)	0
Low SDI	77.79 (34.16,134.90)	201.00 (108.38,320.69)	0.09 (0.04,0.16)	0.10 (0.06,0.16)	0.39 (0.09,0.70)	0.012
Andean Latin America	24.65 (13.63,42.09)	42.15 (25.25,64.40)	0.33 (0.19,0.57)	0.33 (0.20,0.50)	− 0.01 (−0.35,0.33)	0.946
Australasia	1.43 (0.84,2.62)	3.99 (1.81,6.62)	0.03 (0.02,0.06)	0.07 (0.03,0.12)	2.74 (2.25,3.22)	0
Caribbean	7.19 (4.99,10.24)	15.67 (9.06,24.83)	0.10 (0.07,0.15)	0.17 (0.10,0.27)	1.72 (1.47,1.96)	0
Central Asia	35.60 (20.20,51.45)	37.75 (24.04,57.45)	0.26 (0.15,0.38)	0.19 (0.12,0.30)	− 0.99 (− 1.17,− 0.81)	0
Central Europe	26.12 (15.61,35.22)	14.67 (10.19,22.13)	0.11 (0.06,0.15)	0.08 (0.05,0.11)	− 1.28 (− 1.38,− 1.18)	0
Central Latin America	49.55 (35.14,74.42)	106.48 (63.83,148.06)	0.15 (0.11,0.23)	0.21 (0.12,0.29)	1.08 (0.95,1.20)	0
Central Sub-Saharan Africa	6.48 (1.04,17.21)	15.15 (4.09,36.10)	0.07 (0.01,0.19)	0.07 (0.02,0.16)	− 0.31 (− 0.63,0.00)	0.051
East Asia	86.24 (51.20,174.16)	75.82 (44.04,120.72)	0.03 (0.02,0.07)	0.03 (0.02,0.04)	− 0.56 (− 0.95,− 0.17)	0.005
Eastern Europe	35.10 (19.05,55.56)	19.98 (11.57,43.93)	0.08 (0.04,0.12)	0.05 (0.03,0.12)	− 1.34 (−1.60,−1.08)	0
Eastern Sub-Saharan Africa	18.59 (3.94,41.35)	48.90 (16.32,94.77)	0.06 (0.01,0.13)	0.06 (0.02,0.13)	0.33 (0.03,0.64)	0.03
High-income Asia Pacific	23.97 (16.96,37.38)	17.93 (12.99,34.99)	0.07 (0.05,0.11)	0.07 (0.05,0.13)	− 0.28 (− 0.53,− 0.04)	0.025
High-income North America	133.70 (91.34,181.76)	114.18 (84.36,169.33)	0.22 (0.15,0.30)	0.18 (0.13,0.27)	− 0.68 (− 0.79,− 0.57)	0
North Africa and Middle East	48.30 (29.68,86.32)	120.26 (78.20,202.14)	0.08 (0.05,0.14)	0.10 (0.06,0.16)	0.72 (0.63,0.81)	0
Oceania	5.90 (2.91,11.86)	13.77 (6.33,28.63)	0.50 (0.25,1.00)	0.53 (0.25,1.11)	0.16 (0.02,0.29)	0.023
South Asia	258.55 (137.61,413.34)	599.81 (357.69,919.57)	0.14 (0.07,0.22)	0.17 (0.10,0.25)	0.68 (0.21,1.15)	0.005
Southeast Asia	28.81 (18.04,49.64)	53.02 (32.70,92.49)	0.03 (0.02,0.06)	0.04 (0.02,0.07)	0.59 (0.46,0.71)	0
Southern Latin America	18.43 (11.14,27.59)	24.83 (14.57,36.62)	0.19 (0.12,0.29)	0.19 (0.11,0.28)	− 0.08 (− 0.41,0.24)	0.62
Southern Sub-Saharan Africa	21.12 (8.37,34.14)	20.71 (9.09,45.39)	0.21 (0.08,0.34)	0.12 (0.05,0.26)	− 1.90 (− 2.59,− 1.19)	0
Tropical Latin America	40.13 (25.57,57.83)	68.74 (43.41,98.01)	0.13 (0.08,0.19)	0.15 (0.09,0.21)	0.47 (0.23,0.71)	0
Western Europe	47.15 (32.03,73.43)	60.62 (35.34,76.77)	0.06 (0.04,0.10)	0.09 (0.05,0.11)	1.07 (0.91,1.24)	0
Western Sub-Saharan Africa	10.76 (4.45,17.45)	27.18 (13.39,43.83)	0.03 (0.01,0.05)	0.03 (0.02,0.05)	− 0.16 (− 0.36,0.05)	0.146

*ASMR* age-standardised mortality rates, *APPC* average annual percentage change;

**Table 4:** ASIR of interstitial lung disease and pulmonary sarcoidosis in adolescents and young adultsr in 1990 and 2019 for Male and all locations, with AAPC from 2009 and 2019

Location	Numbers in 1990	Numbers in 2019	Age-standardized rates in 1990 (95% CI)	Age standardized rates in 2019 (95% CI)	AAPC, % (95% CI)	P
Global	893,445.39 (521,837.50,1,442,984.51)	1,328,352.15 (785,845.63,2,114,568.54)	84.35 (49.43,136.17)	87.40 (51.66,139.23)	0.13 (0.08,0.19)	0
High SDI	168,050.73 (98,071.86,273,494.19)	185,147.86 (112,565.17,289,833.33)	98.65 (57.46,160.66)	98.68 (59.74,154.46)	0.01 (− 0.07,0.08)	0.876
High-middle SDI	239,424.29 (140,272.17,386,797.76)	253,542.85 (150,554.55,402,709.97)	97.26 (56.98,157.15)	85.71 (50.63,136.45)	− 0.42 (− 0.54,− 0.29)	0
Middle SDI	225,458.72 (128,808.95,368,186.12)	351,988.17 (205,561.93,568,279.18)	63.85 (36.68,104.21)	72.55 (42.28,117.27)	0.45 (0.34,0.56)	0
Low-middle SDI	180,721.67 (105,350.01,290,666.49)	340,333.72 (199,035.66,545,538.68)	88.13 (51.71,141.61)	96.07 (56.35,154.01)	0.31 (0.25,0.37)	0
Low SDI	79,442.18 (45,912.50,127,198.52)	196,626.75 (113,854.56,316,137.78)	90.98 (53.01,145.47)	97.77 (57.05,157.13)	0.26 (0.21,0.31)	0
Andean Latin America	5597.36 (3285.62,8947.63)	12,598.97 (7611.25,19,695.17)	82.26 (48.65,131.63)	100.37 (60.68,156.97)	0.70 (0.60,0.81)	0
Australasia	3172.23 (1841.56,5202.32)	3732.55 (2159.10,5992.90)	76.19 (44.16,124.93)	72.95 (42.10,117.28)	− 0.16 (− 0.20,− 0.13)	0
Caribbean	3373.49 (1900.73,5523.90)	4933.35 (2831.81,8011.23)	50.19 (28.47,82.24)	55.27 (31.75,89.89)	0.34 (0.31,0.37)	0
Central Asia	14,689.99 (8649.62,23,452.33)	22,589.70 (13,356.18,36,001.46)	109.61 (64.76,175.60)	115.23 (68.04,184.32)	0.19 (0.14,0.24)	0
Central Europe	51,490.01 (30,477.02,82,697.45)	49,893.00 (30,607.41,76,993.67)	207.96 (122.75,333.65)	237.18 (144.95,365.80)	0.48 (0.35,0.62)	0
Central Latin America	21,718.75 (12,483.49,35,655.88)	31,788.50 (18,729.77,50,827.49)	72.19 (41.78,118.79)	65.31 (38.53,104.42)	− 0.36 (− 0.39,− 0.32)	0
Central Sub-Saharan Africa	8351.95 (4808.55,13,591.91)	22,794.54 (13,030.47,36,942.70)	88.24 (51.25,143.76)	96.01 (55.26,155.38)	0.30 (0.24,0.35)	0
East Asia	122,287.43 (68,286.49,207,256.06)	92,421.22 (53,739.04,150,393.58)	43.87 (24.58,74.23)	31.54 (18.20,51.54)	− 1.09 (− 1.30,− 0.89)	0
Eastern Europe	97,805.03 (58,035.58,155,594.05)	86,881.43 (51,297.81,139,022.72)	211.20 (124.90,336.56)	216.66 (127.19,346.99)	0.11 (0.04,0.19)	0.003
Eastern Sub-Saharan Africa	24,495.58 (13,992.40,39,605.59)	62,277.12 (35,576.59,101,167.18)	79.71 (45.94,129.05)	84.27 (48.55,137.11)	0.20 (0.16,0.25)	0
High-income Asia Pacific	35,961.35 (20,598.60,59,227.44)	29,674.49 (17,374.27,48,403.39)	104.62 (59.92,171.97)	101.81 (59.38,165.42)	− 0.06 (− 0.14,0.02)	0.137
High-income North America	100,572.10 (59,318.51,163,822.08)	101,365.19 (62,296.76,155,432.36)	165.99 (97.64,270.68)	158.10 (97.05,242.35)	− 0.19 (− 0.46,0.08)	0.169
North Africa and Middle East	73,704.74 (42,622.04,119,529.98)	179,463.46 (104,949.36,288,110.04)	115.10 (66.96,187.00)	129.19 (75.39,207.47)	0.41 (0.37,0.46)	0
Oceania	2158.09 (1272.15,3389.30)	5200.18 (3106.89,8229.24)	171.24 (101.12,268.54)	194.98 (116.59,308.21)	0.44 (0.40,0.47)	0
South Asia	215,938.76 (126,708.40,345,029.66)	443,651.48 (260,306.47,709,230.79)	104.49 (61.64,166.85)	117.26 (68.96,187.46)	0.41 (0.37,0.45)	0
Southeast Asia	23,883.75 (13,111.11,39,848.09)	39,481.75 (21,971.29,66,025.89)	26.09 (14.39,43.60)	28.39 (15.78,47.44)	0.30 (0.27,0.32)	0
Southern Latin America	5871.16 (3372.58,9588.55)	8534.15 (4891.21,13,818.65)	63.73 (36.67,104.13)	66.17 (37.88,107.18)	0.12 (0.08,0.16)	0
Southern Sub-Saharan Africa	14,674.41 (8647.15,23,260.36)	25,231.07 (14,797.32,40,429.52)	150.42 (89.26,238.49)	149.29 (87.51,239.62)	− 0.01 (− 0.06,0.04)	0.601
Tropical Latin America	22,255.40 (12,863.76,36,098.93)	13,646.95 (7976.62,21,850.58)	75.22 (43.67,122.22)	29.75 (17.33,47.61)	− 3.14 (− 3.29,− 3.00)	0
Western Europe	15,850.92 (8921.50,25,965.37)	15,032.40 (8597.08,24,462.65)	21.28 (11.96,34.89)	21.10 (12.01,34.37)	0.03 (− 0.08,0.14)	0.614
Western Sub-Saharan Africa	29,592.90 (16,983.26,47,880.34)	77,160.62 (44,407.41,123,682.02)	92.45 (53.40,149.64)	98.63 (57.18,158.14)	0.24 (0.18,0.29)	0

ASIR,age-standardised incidence rate;APPC,average annual percentage change

**Table 5:** DALYs of interstitial lung disease and pulmonary sarcoidosis in adolescents and young adultsr in 1990 and 2019 for Male and all locations, with AAPC from 2009 and 2019

Location	Numbers in 1990	Numbers in 2019	Age-standardized rates in 1990 (95% CI)	Age standardized rates in 2019 (95% CI)	AAPC, % (95% CI)	P
Global	89,757.09 (65,350.77,125,630.23)	133,118.92 (99,293.52,175,061.00)	8.44 (6.14,11.80)	8.76 (6.54,11.53)	0.17 (− 0.01,0.34)	0.061
High SDI	17,789.28 (14,082.11,24,755.58)	18,419.70 (14,631.81,23,404.41)	10.47 (8.28,14.61)	9.91 (7.85,12.59)	− 0.19 (− 0.33,− 0.04)	0.011
High-middle SDI	17,350.76 (13,390.87,22,661.58)	18,336.41 (13,665.82,23,006.84)	7.05 (5.44,9.20)	6.34 (4.71,7.94)	− 0.37 (− 0.57,− 0.17)	0
Middle SDI	21,752.19 (16,041.35,31,997.46)	34,599.46 (26,300.09,44,053.44)	6.11 (4.51,8.96)	7.15 (5.43,9.11)	0.59 (0.43,0.75)	0
Low-middle SDI	24,558.53 (13,563.11,42,990.22)	43,587.58 (27,738.85,67,308.29)	12.04 (6.61,21.10)	12.35 (7.84,19.10)	0.12 (− 0.14,0.38)	0.361
Low SDI	8237.29 (4215.04,14,496.25)	18,044.44 (10,936.07,27,077.67)	9.50 (4.86,16.72)	9.02 (5.46,13.54)	− 0.17 (− 0.37,0.03)	0.092
Andean Latin America	2374.30 (1270.11,4074.79)	3896.82 (2411.22,5831.02)	33.18 (17.87,56.77)	30.62 (18.96,45.79)	− 0.16 (− 0.70,0.37)	0.552
Australasia	172.58 (107.14,281.89)	387.30 (186.91,634.03)	4.14 (2.57,6.75)	7.57 (3.62,12.47)	2.13 (1.51,2.75)	0
Caribbean	443.56 (297.01,694.82)	904.31 (592.00,1399.64)	6.56 (4.40,10.27)	10.13 (6.63,15.67)	1.51 (1.34,1.69)	0
Central Asia	1620.00 (1041.04,2267.08)	2360.02 (1373.62,3370.74)	11.99 (7.71,16.75)	12.05 (7.03,17.19)	0.07 (− 0.36,0.50)	0.765
Central Europe	2848.79 (1866.79,3947.22)	1886.91 (1313.91,2585.00)	11.58 (7.58,16.06)	9.21 (6.39,12.58)	− 0.79 (− 0.92,− 0.66)	0
Central Latin America	2939.51 (2247.24,4319.92)	7112.31 (3767.94,10,029.23)	9.57 (7.33,14.03)	14.53 (7.68,20.50)	1.53 (0.93,2.13)	0
Central Sub-Saharan Africa	675.90 (245.13,1509.03)	1639.70 (751.43,3357.63)	7.21 (2.61,16.14)	6.94 (3.17,14.16)	− 0.12 (− 0.20,− 0.05)	0.001
East Asia	9224.66 (6301.81,13,813.28)	7648.89 (5458.07,10,259.22)	3.26 (2.23,4.88)	2.66 (1.89,3.58)	− 0.68 (− 0.93,− 0.43)	0
Eastern Europe	3730.32 (2422.68,5345.60)	2753.91 (1811.82,4039.56)	8.08 (5.26,11.58)	6.88 (4.53,10.11)	− 0.48 (− 1.08,0.12)	0.118
Eastern Sub-Saharan Africa	2229.91 (801.45,3998.63)	5058.83 (2402.70,8012.75)	7.17 (2.58,12.82)	6.79 (3.22,10.71)	− 0.21 (− 0.43,0.00)	0.05
High-income Asia Pacific	3496.27 (2100.92,5949.96)	2684.50 (1863.45,3785.64)	10.17 (6.10,17.31)	9.19 (6.37,12.97)	− 0.29 (− 0.46,− 0.13)	0
High-income North America	9876.28 (7455.57,12,520.55)	8155.86 (6341.61,10,782.23)	16.31 (12.31,20.73)	12.79 (9.93,16.91)	− 0.83 (− 1.14,− 0.53)	0
North Africa and Middle East	4631.12 (2843.62,7697.76)	10,878.59 (7636.13,16,078.58)	7.07 (4.37,11.77)	7.85 (5.51,11.62)	0.35 (0.13,0.57)	0.002
Oceania	753.66 (411.65,1347.89)	1580.74 (857.09,2825.77)	56.81 (31.25,101.06)	57.68 (31.39,102.90)	0.04 (− 0.03,0.11)	0.274
South Asia	30,504.79 (15,672.34,54,511.48)	55,478.33 (33,584.29,87,130.19)	14.85 (7.61,26.53)	14.70 (8.89,23.09)	− 0.02 (− 0.31,0.27)	0.897
Southeast Asia	2878.32 (1701.64,5949.46)	4625.03 (3110.02,7776.27)	3.02 (1.79,6.19)	3.35 (2.25,5.65)	0.39 (0.20,0.58)	0
Southern Latin America	1000.01 (655.12,1545.74)	1573.78 (925.23,2362.78)	10.83 (7.11,16.75)	12.22 (7.17,18.38)	0.42 (− 0.11,0.94)	0.122
Southern Sub-Saharan Africa	1287.24 (785.06,1787.04)	1729.65 (1180.42,2628.47)	13.84 (8.40,19.20)	10.16 (6.93,15.43)	− 1.15 (− 1.87,− 0.43)	0.002
Tropical Latin America	3518.84 (2429.63,4634.35)	4774.22 (3076.17,6153.85)	11.84 (8.17,15.60)	10.44 (6.72,13.46)	− 0.44 (− 0.79,− 0.09)	0.015
Western Europe	4423.25 (3215.25,7168.89)	5316.73 (3111.99,6759.03)	5.94 (4.32,9.63)	7.47 (4.32,9.51)	0.75 (0.54,0.97)	0
Western Sub-Saharan Africa	1127.78 (612.53,1743.41)	2672.50 (1715.75,3964.13)	3.55 (1.93,5.47)	3.46 (2.22,5.10)	− 0.10 (− 0.23,0.03)	0.131

APPC,average annual percentage change

**Table 6:** ASMR of interstitial lung disease and pulmonary sarcoidosis in adolescents and young adultsr in 1990 and 2019 for Male and all locations, with AAPC from 2009 and 2019

location	Numbers in 1990	Numbers in 2019	Age-standardized rates in 1990 (95% CI)	Age standardized rates in 2019 (95% CI)	AAPC, % (95% CI)	P
Global	1272.37 (884.72,1831.56)	1926.42 (1379.55,2582.66)	0.12 (0.08,0.17)	0.13 (0.09,0.17)	0.22 (0.00,0.43)	0.052
High SDI	242.36 (193.93,353.55)	251.89 (197.55,321.42)	0.14 (0.11,0.21)	0.13 (0.10,0.17)	− 0.19 (− 0.42,0.04)	0.103
High-middle SDI	227.25 (170.48,299.83)	245.91 (172.76,301.62)	0.09 (0.07,0.12)	0.08 (0.06,0.10)	− 0.31 (− 0.60,− 0.02)	0.033
Middle SDI	306.77 (214.63,466.41)	502.55 (366.16,652.78)	0.09 (0.06,0.13)	0.10 (0.08,0.13)	0.62 (0.26,0.98)	0.001
Low-middle SDI	375.60 (185.50,691.39)	669.49 (395.91,1084.62)	0.19 (0.09,0.34)	0.19 (0.11,0.31)	0.10 (− 0.21,0.41)	0.527
Low SDI	119.38 (51.88,225.71)	254.67 (136.97,405.62)	0.14 (0.06,0.27)	0.13 (0.07,0.21)	− 0.28 (− 0.53,− 0.03)	0.03
Andean Latin America	38.06 (19.84,66.23)	62.66 (38.15,95.14)	0.54 (0.28,0.94)	0.49 (0.30,0.75)	− 0.20 (− 0.74,0.35)	0.482
Australasia	2.16 (1.20,3.99)	5.76 (2.41,9.94)	0.05 (0.03,0.10)	0.11 (0.05,0.19)	2.75 (1.98,3.52)	0
Caribbean	6.62 (4.23,10.95)	14.18 (9.03,22.67)	0.10 (0.06,0.16)	0.16 (0.10,0.25)	1.63 (1.43,1.82)	0
Central Asia	23.72 (14.09,34.25)	35.17 (18.35,52.40)	0.18 (0.11,0.26)	0.18 (0.09,0.27)	0.09 (− 0.41,0.58)	0.731
Central Europe	35.60 (21.58,50.90)	19.27 (12.11,25.56)	0.14 (0.09,0.21)	0.09 (0.06,0.12)	− 1.51 (− 1.80,− 1.22)	0
Central Latin America	43.62 (32.72,66.61)	113.01 (56.06,162.60)	0.14 (0.11,0.22)	0.23 (0.11,0.33)	1.73 (1.07,2.39)	0
Central Sub-Saharan Africa	9.26 (2.25,23.41)	21.85 (7.68,50.06)	0.10 (0.02,0.26)	0.09 (0.03,0.21)	− 0.30 (− 0.44,− 0.15)	0
East Asia	117.52 (74.56,189.17)	106.52 (70.36,148.03)	0.04 (0.03,0.07)	0.04 (0.02,0.05)	− 0.41 (− 0.74,− 0.07)	0.017
Eastern Europe	38.70 (23.58,58.34)	24.75 (16.02,37.69)	0.08 (0.05,0.13)	0.06 (0.04,0.09)	− 1.01 (− 1.99,− 0.02)	0.045
Eastern Sub-Saharan Africa	30.83 (7.82,59.88)	68.67 (25.53,116.06)	0.10 (0.03,0.20)	0.09 (0.03,0.16)	− 0.26 (− 0.48,− 0.04)	0.021
High-income Asia Pacific	38.11 (22.52,74.05)	27.38 (20.31,39.39)	0.11 (0.07,0.22)	0.09 (0.07,0.14)	− 0.53 (− 0.77,− 0.29)	0
High-income North America	142.37 (104.63,183.71)	112.34 (85.91,155.57)	0.23 (0.17,0.30)	0.18 (0.13,0.24)	− 0.96 (− 1.26,− 0.66)	0
North Africa and Middle East	58.11 (31.54,108.47)	139.83 (93.79,223.10)	0.09 (0.05,0.17)	0.10 (0.07,0.16)	0.36 (0.07,0.65)	0.016
Oceania	11.12 (5.67,20.70)	22.75 (11.17,43.17)	0.85 (0.44,1.58)	0.84 (0.41,1.59)	− 0.08 (− 0.15,0.00)	0.05
South Asia	473.77 (215.12,883.66)	854.35 (477.63,1401.05)	0.23 (0.11,0.44)	0.23 (0.13,0.37)	− 0.08 (− 0.42,0.27)	0.665
Southeast Asia	40.69 (22.45,88.10)	66.58 (43.44,118.31)	0.04 (0.02,0.09)	0.05 (0.03,0.09)	0.42 (0.20,0.64)	0
Southern Latin America	15.39 (9.70,24.73)	24.43 (13.62,37.54)	0.17 (0.11,0.27)	0.19 (0.11,0.29)	0.49 (0.23,0.75)	0
Southern Sub-Saharan Africa	18.66 (10.02,26.66)	23.73 (15.05,39.03)	0.20 (0.11,0.29)	0.14 (0.09,0.23)	− 1.46 (− 2.49,− 0.42)	0.006
Tropical Latin America	54.15 (35.98,73.26)	79.22 (49.90,103.45)	0.18 (0.12,0.25)	0.17 (0.11,0.22)	− 0.23 (− 0.62,0.16)	0.249
Western Europe	62.60 (44.85,108.56)	78.90 (41.35,100.15)	0.08 (0.06,0.15)	0.11 (0.06,0.14)	0.87 (0.66,1.08)	0
Western Sub-Saharan Africa	11.31 (4.60,19.27)	25.06 (14.50,38.23)	0.04 (0.01,0.06)	0.03 (0.02,0.05)	− 0.30 (− 0.42,− 0.17)	0

**Fig. 1:**
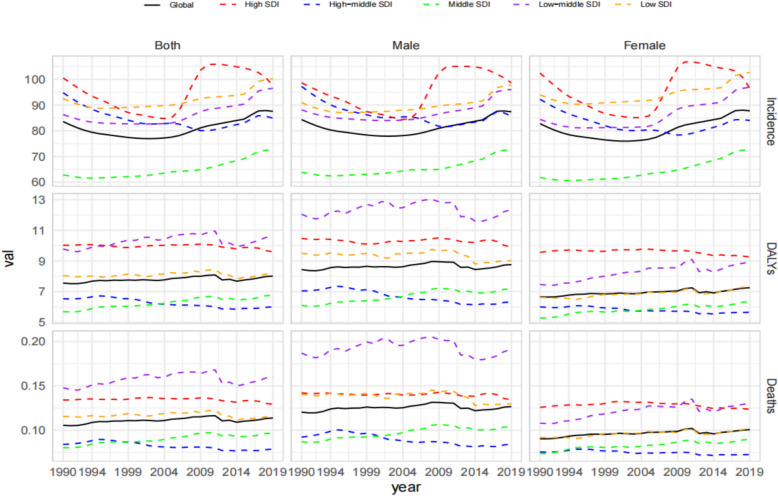
Temporal trend of age-standardised incidence rate, age-standardised disability-adjusted life years rate, and age-standardised mortality rate for the burden of interstitial lung disease and pulmonary sarcoidosis in adolescents and young adults, globally and by socio-demographic index (SDI). Five categories: countries with a high, high–middle, middle, low–middle, or low SDI from 1990 to 2019

Countries in Andean Latin America and Oceania DALYs were concentrated in the highest quintile. Eastern and Central Europe, as well as Oceania and Andean Latin America, were in the highest quintile (Fig. 1). Age-standardised incidence rates were higher for women (87.77) than for men (86.77), while age-standardised DALY and mortality rates were higher for men (8.76% and 0.13%, respectively) than for women (7.26 and 0.10, respectively).

### Temporal trend of ILD and pulmonary sarcoidosis in AYAs from 1990 to 2019

**Fig. 2:**
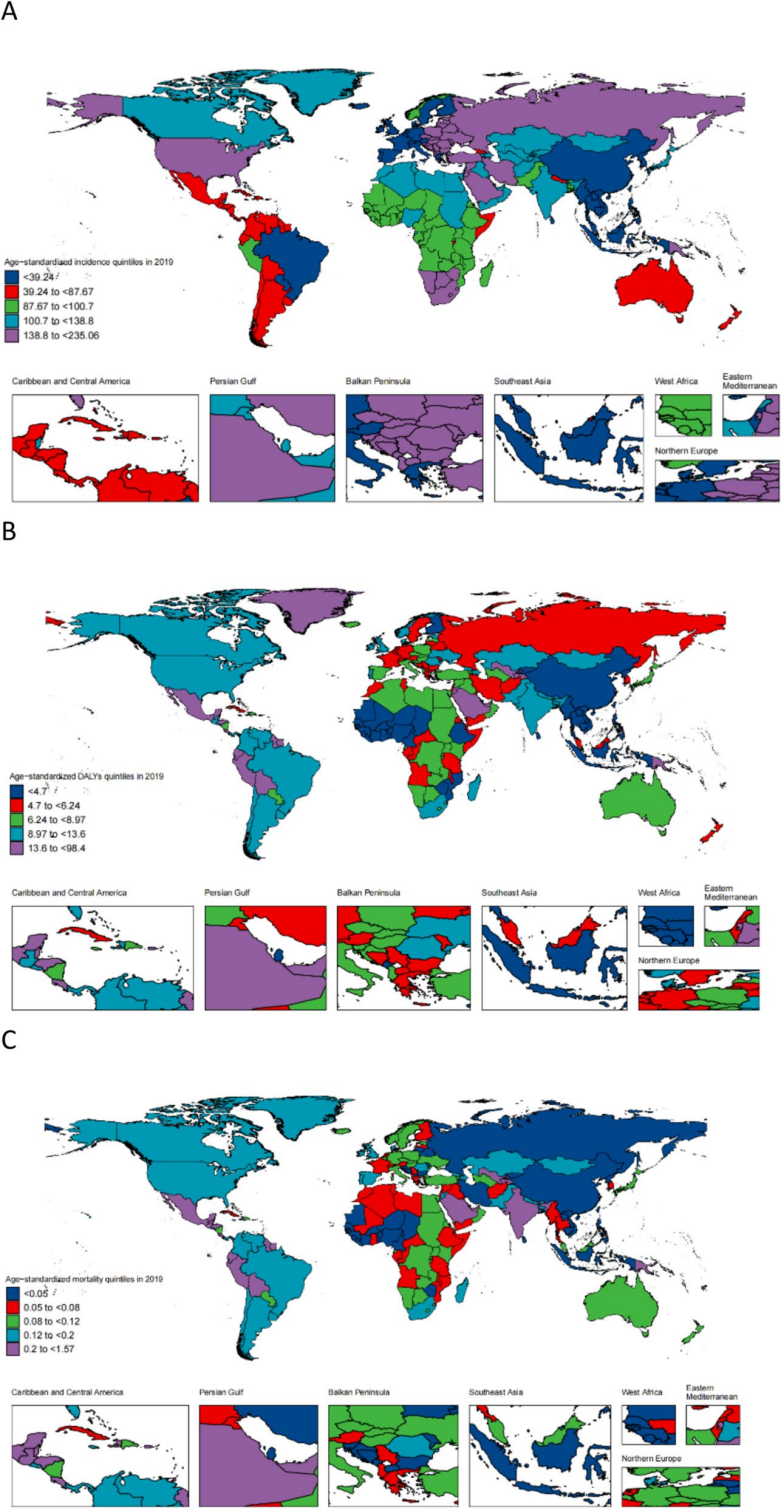
Global map of age-standardised disability-adjusted life years (DALYs) rates for both sexes combined in 2019, categorised based on age-standardised incidence, DALYs, mortality rate quintiles for interstitial and pulmonary sarcoidosis in AYAs. (**A**) Age-standardised incidence quintiles in 2019. (**B**) Age-standardised DALYs quintiles in 2019. (**C**) Age-standardised mortality quintiles in 2019

The global age-standardised incidence rate increased significantly from 83.56 (95% CI 48.79–135.72) in 1990 to 87.58 (51.66–140.35) in 2019. Nationally,Age- standardised incidence rate increased more rapidly in the middle SDI countries, with mean annual percentage changes of 0.29% (95% CI 0.25–0.32%) and 0.51% (0.43–0.58%), respectively. Global age- standardised DALY rate increased from 7.58 (95% CI 6.10–9.68) in 1990 to 8.02 (6.45–9.96) in 2019.Age-standardised DALY rates decreased in high- and middle–high-SDI countries but increased in low-, middle–low-, and low-SDI countries. Middle-SDI countries'average annual percentage change was 0.59% (0.07–0.90%). The Age-standardised incidence and DALY rates for ILD and pulmonary sarcoidosis in AYAs in the Andean Latin American and South Australasian regions increased the fastest between 1990 and 2019. At the national level, the Taiwan Province of China and Colombia showed the fastest increases in age-standardised incidence and DALY rates, respectively. Overall, the global age-standardised mortality rate fluctuated and changed relatively Little from 1990 to 2019, increasing in countries with low to middle SDI and remaining stable in countries with high, middle–high, and low SDI (Fig. 2).

**Fig. 3:**
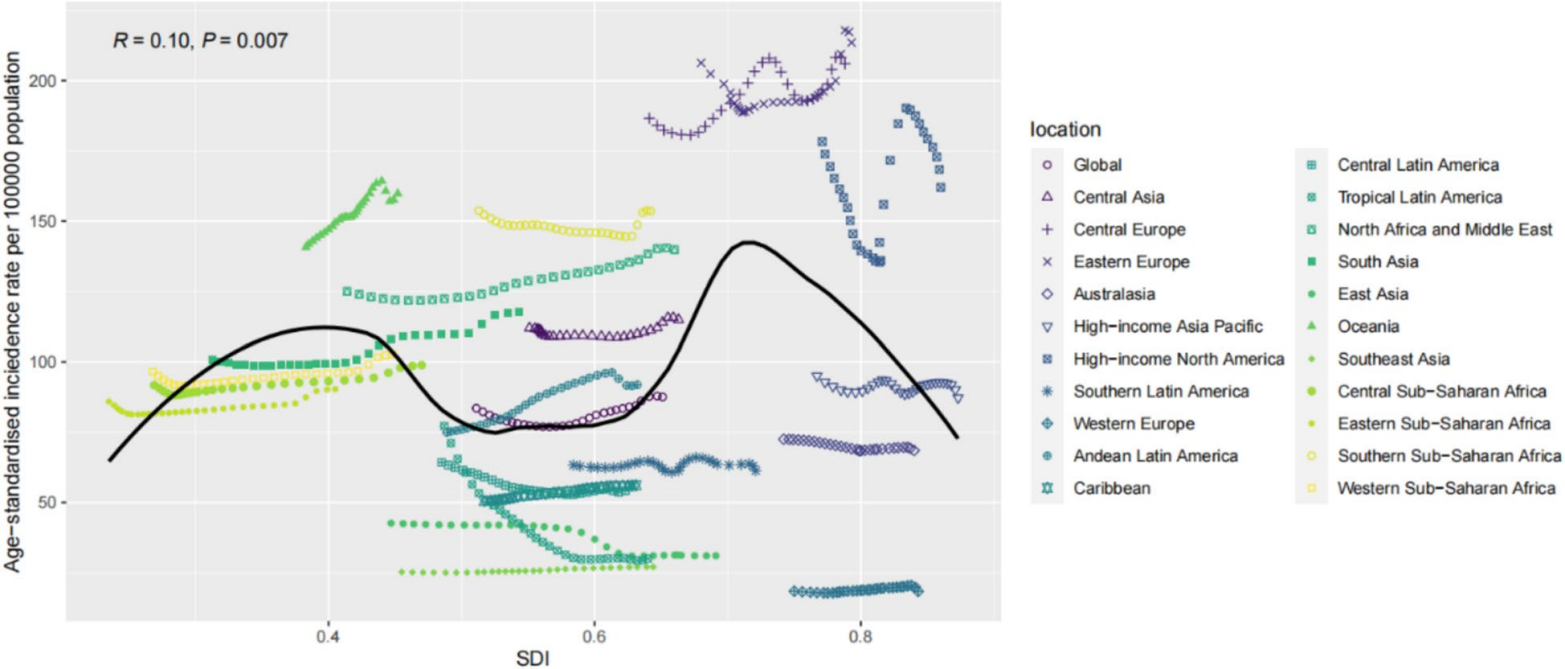
Age-standardised incidence rate (ASIR) of interstitial lung disease and pulmonary sarcoidosis per 100,000 individuals among regions based on socio-demographic indices of each year

**Fig. 4:**
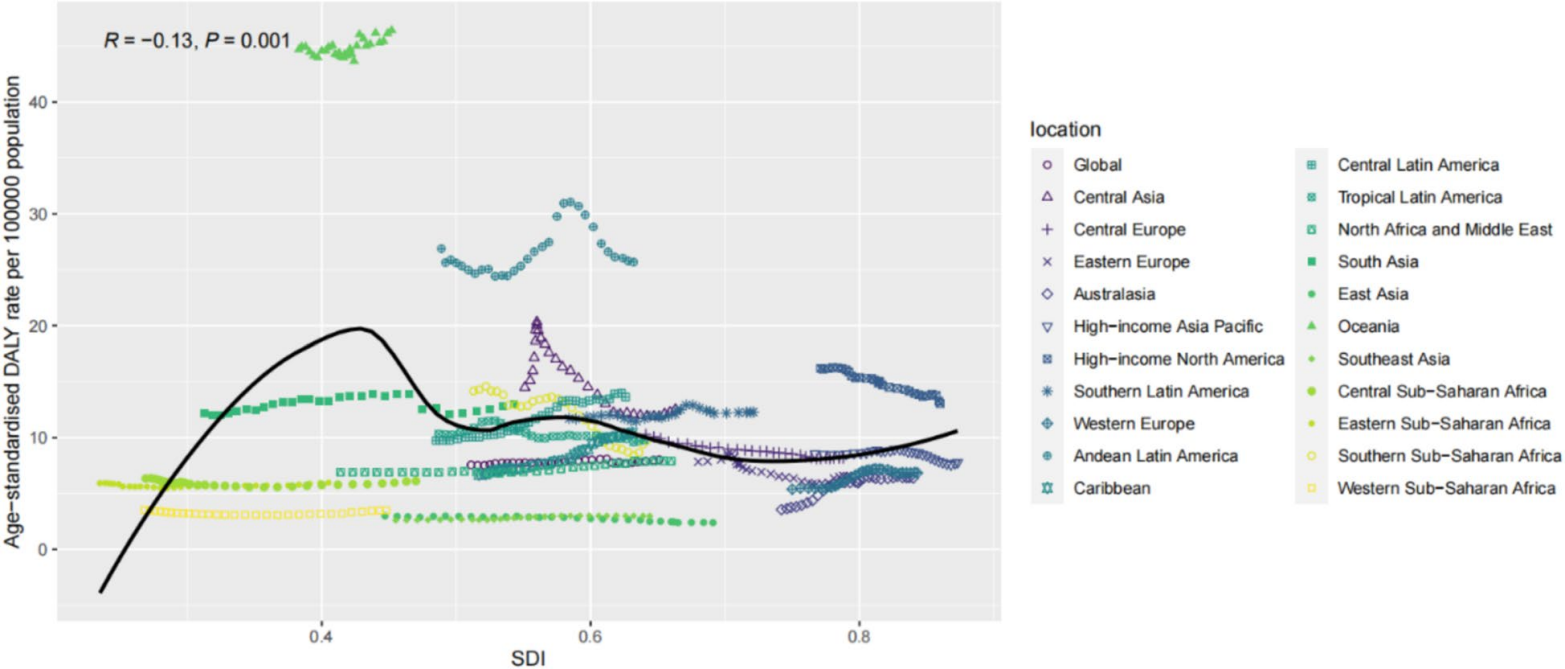
Age-standardised DALY rate of interstitial lung disease and pulmonary sarcoidosis per 100,000 individuals among regions based on the SDI of each year

**Fig. 5:**
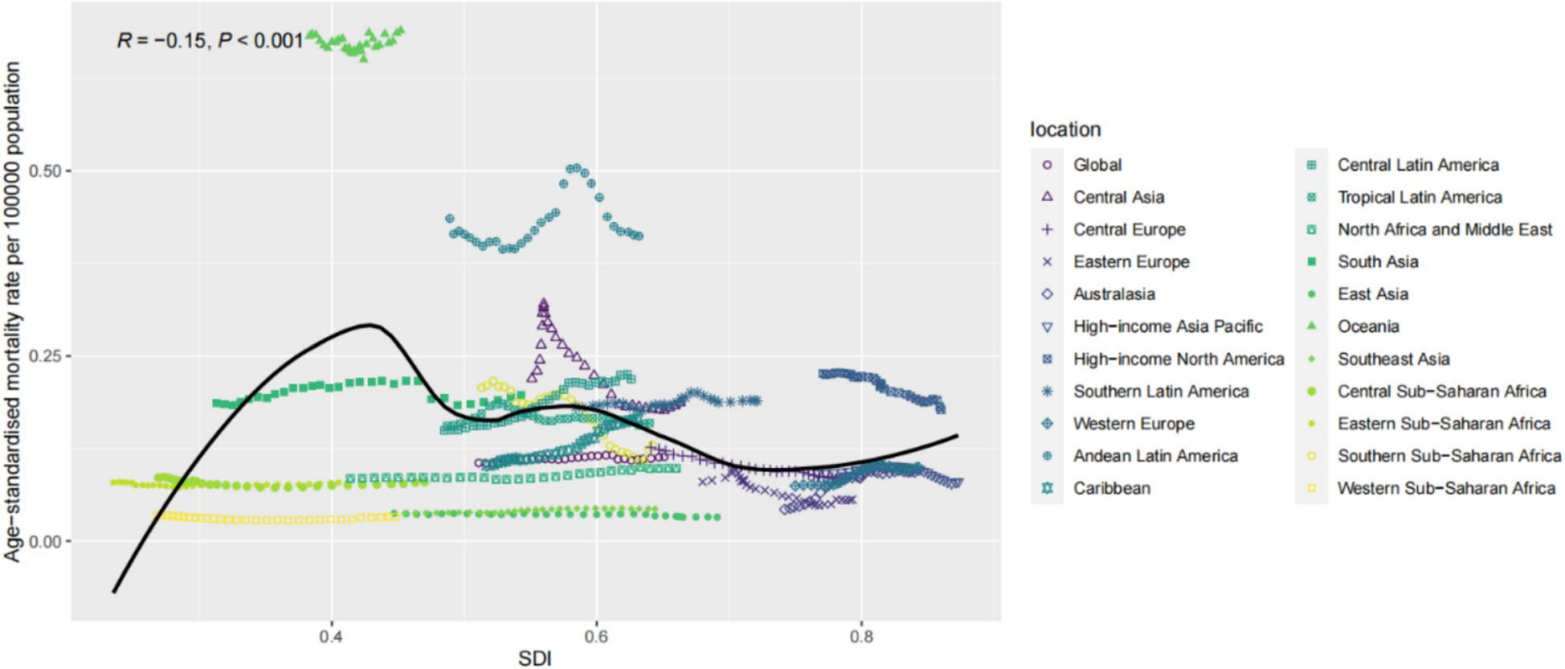
Age-standardised mortality rate (ASMR) of interstitial lung disease and pulmonary sarcoidosis per 100,000 individuals among regions based on socio-demographic indices of each year

From 1990 to 2019, a significant association was found between age-standardised DALY rate and the SDI (R = –0.13, P = 0.001). When the SDI was < 0.43, the age-standardised DALY rate increased significantly as the SDI increased. In contrast, when the SDI was > 0.43, the age-standardised DALY rate gradually decreased as the SDI increased, eventually stabilising at 10 per 100,000. The overall age-standardised DALY rate remained consistently high in Andean-Latin American cities, while the pattern was not linear in Central Asia. A similar pattern was observed in age-standardised mortality rate, which was also significantly correlated with the SDI (R = –0.15, P = 0.001). Additionally, a correlation was found between age-standardised morbidity rate and the SDI (R = 0.10, P = 0.007). When the SDI was < 0.40, the age-standardised incidence rate increased significantly with increasing SDI. At SDI values between 0.43 and 0.6, age-standardised DALY rate decreased with increasing SDI. When the SDI was between 0.6 and 0.72, the incidence rate tended to increase significantly with increasing SDI. When the SDI was > 0.72, the age-standardised DALY rate decreased with increasing SDI and stabilised at 10 per 100,000. Overall, age-standardised incidence rates continued to increase in Central Europe and high-income North America (Fig 3, 4, 5).

### Decomposition analysis of incidence, DALYs, and mortality rates (Appendix tables 13–15, Fig. 6)

**Fig. 6:**
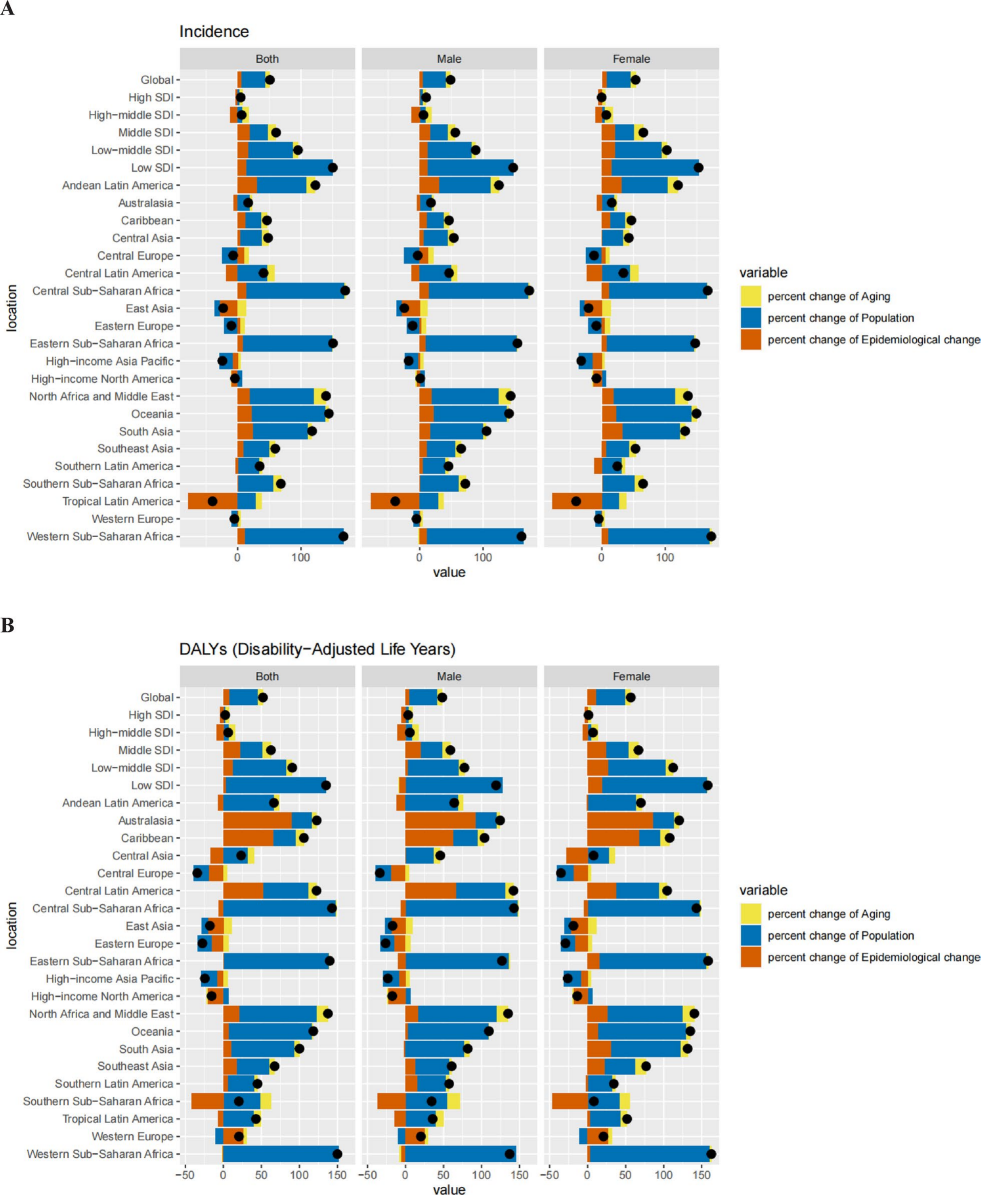
Changes in interstitial lung disease and pulmonary sarcoidosis in adolescent and young adults. **A**; incidence, **B**; disability-adjusted Life years, and C; deaths according to population-level determinants of population growth, ageing, and epidemiological change from 1990 to 2019 across locations. Black dot represents the overall value of change contributed by all three components. For each component, the magnitude of a positive value indicates a corresponding increase attributed to the component; the magnitude of a negative value indicates a corresponding decrease attributed to the related component

The incidence of DALYs associated with ILD and pulmonary sarcoidosis has increased significantly globally over the past 3 decades. This surge was particularly pronounced in regions with low SDIs primarily driven by population growth. This had a significant impact on areas in the low SDI quintiles. This pattern was consistent for both males and females. Globally, AYAs have experienced a significant increase in mortality due to ILD and pulmonary sarcoidosis, with the most pronounced increases observed in areas in the low SDI quintiles. Population growth and ageing have played major roles in these trends, leading to an increase in the number of deaths associated with ILD and pulmonary sarcoidosis. Western Saharan Africa made the largest contribution to population growth, and epidemiological changes had a relatively small impact on morbidity growth. Ageing also negatively affects morbidity. The demographic and epidemiological impact on disease incidence varies by country and region; however, the pattern remains stable for both males and females. Deaths associated with ILD and pulmonary nodular disease surged in most regions, except Central Europe, East Asia, Eastern Europe, high-income Asia–Pacific, and high-income North America. Epidemiological changes had the greatest impact on the increase in cases of ILD and pulmonary nodular disease among young people in Australasia (132.7%), followed by the Caribbean (72.44%) and Central Latin America (60.29%). Population expansion emerged as the main factor contributing to the surge in mortality from ILD and pulmonary nodular disease in western sub-Saharan Africa (147.35%), eastern sub-Saharan Africa (136.92%), North Africa, and the Middle East (102.7%). This pattern was observed in both men and women.

### Predictions for ILD and pulmonary sarcoidosis in AYAs over the next 30 years(Fig. 7)

**Fig. 7:**
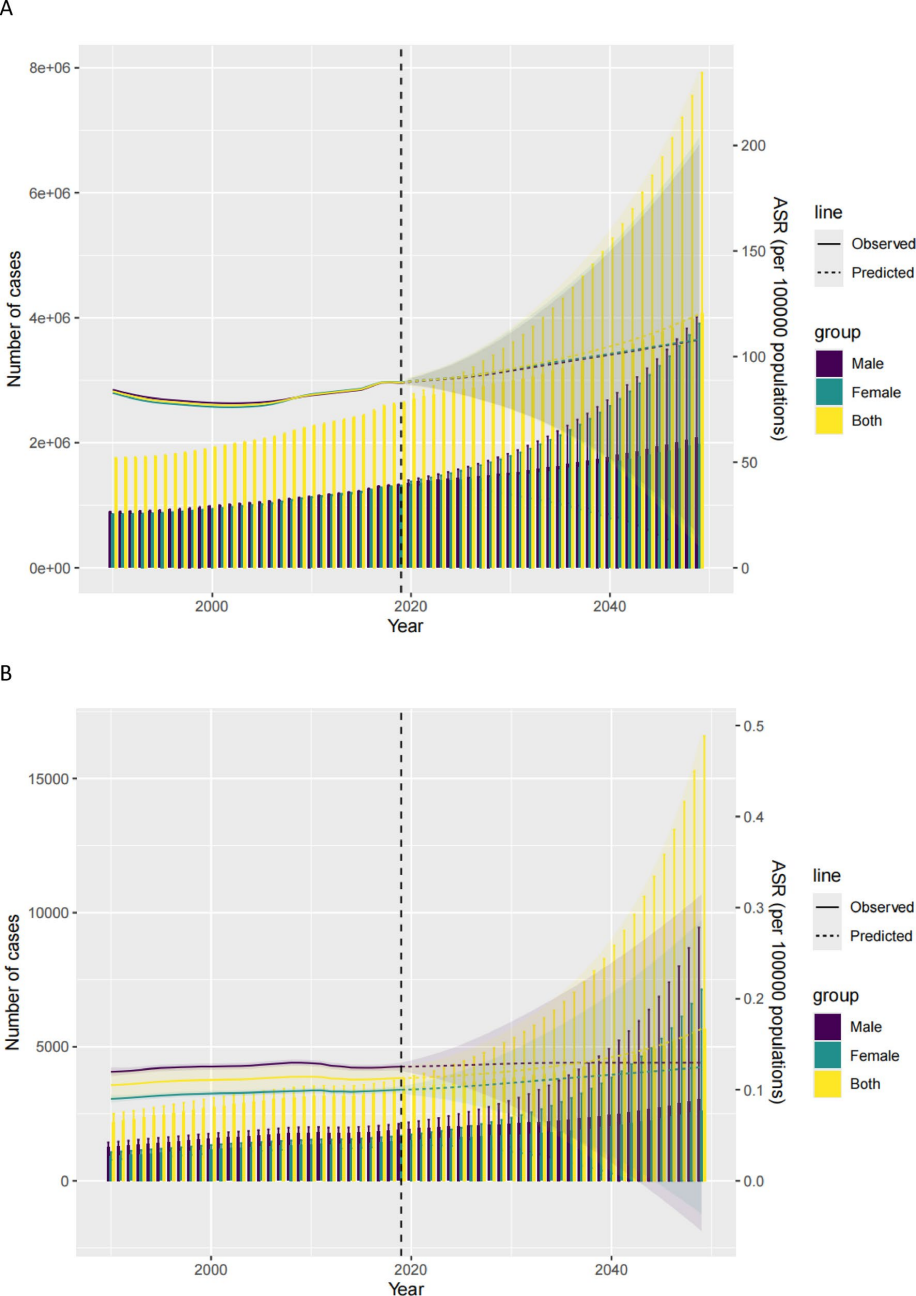
Trends in observed and predicted ASRs of nterstitial lung disease and pulmonary sarcoidosis in adolescent and young adults. from 1990 to 2049 by Bayesian Age-Period-Cohort model. (**A**) ASIR. (**B**) ASMR. (**C**) ASDR. ASIR, age-standardized incidence rate; ASMR, age-standardized mortality rate; ASDR, age-standardized disabilityadjusted life years rate

The BAPC model was employed to observe and predict the trends of ASIR, ASMR, and ASDR for interstitial lung disease and pulmonary sarcoidosis among adolescents and young adults from 2020 to 2049. Over the next 30 years, the number of cases and ASR predictions for interstitial lung disease and pulmonary sarcoidosis among adolescents and young adults globally are expected to show a significant upward trend. From 2020 to 2049, the global ASIR for interstitial lung disease and pulmonary sarcoidosis among adolescents and young adults increased from 88.23 to 120.16. Meanwhile, the global ASMR for interstitial lung disease and pulmonary sarcoidosis adolescents and young adults Rose from 0.11 in 2020 to 0.17 in 2049. Moreover, the age-standardized mortality rate of interstitial lung disease and pulmonary sarcoidosis among adolescents and young adults worldwide increased from 8.09 in 2020 to 11.91 in 2049.

## Discussion

Previous studies on ILD and pulmonary sarcoidosis have predominantly focused on the general population, with a particular emphasis on the elderly, while rarely addressing adolescents and young adults. However, the impact of these conditions on healthy Life expectancy loss is exceptionally high. Therefore, this study is the first to utilize data from the 2019 Global Burden of Disease (GBD) study to investigate the global impact of interstitial lung disease (ILD) and pulmonary sarcoidosis on adolescents and young adults (AYAs) from 1990 to 2019, and to predict disease trends over the next 30 years.

In this study, we collected data on the incidence, mortality, and DALYs in AYAs from 204 countries and territories between 1990 and 2019, focusing on ILD and publicly available information on pulmonary sarcoidosis. The findings of this study contribute to our understanding of ILD and pulmonary sarcoidosis patterns in AYAs. Consistent with a previous study [20], an increasing trend in the global age-standardised incidence and DALY rates was observed in AYAs. Additionally, age-standardised incidence rates were highest in countries with low SDIs. This trend is associated with factors such as increased industrial activity, air pollution, tobacco smoke, automobile traffic, and higher levels of occupational exposure in these densely populated areas [21, 22]. Furthermore, age-standardised DALY and mortality rates among AYAs were highest in low SDI countries. Several factors contribute to this: (1) The diagnosis of ILD or pulmonary sarcoidosis imposes a significant financial burden on individuals, particularly affecting low-income populations [23]. (2) Rising treatment costs over time exacerbate financial pressures. Individuals with low incomes or living in low-income areas frequently face challenges accessing necessary medications and medical services [24]. Owing to advances in healthcare and public health, the global DALYs is expected to decrease. However, our findings show that standardised DALY rates decreased in high and middle–high SDI countries but increased in low, middle–low, and low-SDI countries. These inter-regional differences were evident. Low-SDI countries could benefit from observing and adopting best practices by high-SDI countries to advocate for a more efficient allocation of healthcare resources. Our analyses showed that age-standardised morbidity in AYAs was higher in women, whereas age-standardised DALY and mortality were higher in men. Despite the higher incidence in women, men tended to have a poorer prognosis. Additionally, CTD-ILD is more common in women but tends to progress more rapidly in men, with a higher prevalence of common interstitial pneumonia [25, 26].Between 1990 and 2019, age-standardised DALY and mortality rates were negatively correlated with SDI in AYAs. Conversely, age-standardised morbidity was positively correlated with the SDI. This can be attributed to the increased accessibility of healthcare in more developed regions, leading to early detection and timely treatment [27]. Our study showed a significant increase in global morbidity among AYAs, especially in low-SDI areas, Mainly due to population growth and epidemiological changes. Analyses of global mortality and DALYs among AYAs showed that epidemiological changes and population growth led to increases in mortality and DALYs. Over the next 30 years, the number of cases of interstitial lung disease and pulmonary sarcoidosis among adolescents and young adults globally, as well as the ASR predictions, are expected to show a significant upward trend. This further underscores the need to strengthen early diagnosis and early treatment of these diseases to reduce the medical burden.

Our study was Limited by a lack of primary sources, and the GBD 2019 data May have underestimated the total burden of ILD. The GBD 2019 data excluded external causes of ILD, pharmacological ILD, ILD due to systemic rheumatic diseases, and other specific types of ILD under the categories of ILD and pulmonary sarcoidosis [28]. Therefore, future GBD reports should include these subtypes to provide a more comprehensive overview of the burden of ILD. The complexity and country-specific nature of the classification of ILD and pulmonary nodular disease present challenges in identifying cases and determining data sources. Including a broader and more detailed classification of ILD in the GBD study will improve our understanding of the disease burden. Moreover, the global COVID-19 pandemic has had a relatively significant impact on respiratory diseases. Therefore, we initially conducted an analysis based on data prior to 2019, as these data were entirely unaffected by COVID-19. We hope to be able to make comparisons over the next five or even ten years as new data become available.

## Conclusions

AYAs with ILD and pulmonary sarcoidosis are at a greater risk of long-term effects that may impact productivity growth and social structure than older patients. Therefore, recognising and addressing the global burden of ILD and pulmonary sarcoidosis in AYAs is critical for developing effective healthcare policies and improving the outcomes associated with these diseases.

## Supplementary Information

Supplementary material 1

## Data Availability

No datasets were generated or analysed during the current study.
