# Supplementary material for: Global Burden of Disease of interstitial lung disease and pulmonary sarcoidosis in adolescents and young adults (1990–2019), and projections for the next 30 years
Source: Eur J Med Res. 2025 Sep 26;30:878. doi: 10.1186/s40001-025-03141-x (PMC12465843; doi:10.1186/s40001-025-03141-x)
Supplement: Supplementary file 1 — Supplementary material 1 [file 40001_2025_3141_MOESM1_ESM.doc]

**Appendices A**

SDI：Considering its composite nature, a locale with an SDI of 0 would indicate the lowest conceivable level of development pertinent to health, whereas an SDI of 1 would represent the utmost theoretical level. Using the SDI value, all nations are categorized into ranges including low SDI (0-0.454743), lowemiddle SDI (0.454743-0.607679), middle SDI (0.607679-0.689504), highemiddle SDI (0.689504- 0.805129), and high SDI (0.805129-1) segments.

**Appendices B**

| | **Table 1.ASIR of interstitial lung disease and pulmonary sarcoidosis in adolescents and young adultsr in 1990 and 2019 for female and all locations, with AAPC from 2009 and 2019** | | | | | | |  | | --- | --- | --- | --- | --- | --- | --- | --- | | location | Numbers in 1990 | Numbers in 2019 | Age-standardized rates in 1990   (95% CI) | Age standardized rates in 2019   (95% CI) | AAPC, %   (95% CI) | P |  | | Global | 853342.44 (494195.54,1394724.82) | 1308087.88 (771632.08,2109747.89) | 82.77 (48.09,135.33) | 87.77 (51.72,141.67) | 0.23 (0.09,0.37) | 0.001 |  | | High SDI | 169649.88 (98843.00,276866.58) | 169043.69 (101903.16,264254.68) | 102.43 (59.56,167.22) | 96.53 (58.00,150.84) | -0.15 (-0.28,-0.03) | 0.016 |  | | High-middle SDI | 219642.98 (127896.58,359275.03) | 234853.74 (138399.84,379835.70) | 92.26 (53.72,150.92) | 84.04 (49.26,136.01) | -0.32 (-0.36,-0.27) | 0 |  | | Middle SDI | 211091.62 (120205.57,348019.00) | 349448.73 (203717.64,567688.57) | 61.81 (35.40,101.92) | 72.74 (42.29,118.36) | 0.57 (0.52,0.61) | 0 |  | | Low-middle SDI | 168880.10 (97488.82,274521.05) | 342372.92 (199628.54,553919.87) | 84.45 (49.09,137.35) | 96.95 (56.66,156.98) | 0.51 (0.39,0.63) | 0 |  | | Low SDI | 83767.75 (48282.17,135356.98) | 211728.09 (121631.47,342044.24) | 94.02 (54.55,151.97) | 102.70 (59.40,166.08) | 0.33 (0.22,0.45) | 0 |  | | Andean Latin America | 4832.05 (2809.25,7810.22) | 10644.21 (6363.93,17054.63) | 68.01 (39.83,109.96) | 83.59 (49.99,133.97) | 0.70 (0.63,0.78) | 0 |  | | Australasia | 2878.04 (1639.73,4729.51) | 3330.45 (1921.44,5434.97) | 68.90 (39.20,113.21) | 64.21 (36.87,104.93) | -0.23 (-0.26,-0.21) | 0 |  | | Caribbean | 3557.52 (2010.92,5885.96) | 5214.06 (3012.04,8666.10) | 50.87 (28.92,84.26) | 56.97 (32.90,94.79) | 0.38 (0.34,0.42) | 0 |  | | Central Asia | 15595.69 (9228.39,24842.33) | 22240.02 (13056.76,36158.31) | 114.28 (67.83,182.73) | 114.67 (67.23,186.91) | 0.01 (-0.02,0.04) | 0.532 |  | | Central Europe | 39843.54 (23451.91,64829.14) | 34922.45 (21311.13,55033.97) | 164.71 (96.67,267.26) | 173.50 (105.38,272.68) | 0.20 (0.12,0.27) | 0 |  | | Central Latin America | 17786.21 (10035.60,29793.81) | 23846.96 (13841.65,38908.68) | 56.85 (32.26,95.48) | 46.44 (26.95,75.75) | -0.70 (-0.76,-0.64) | 0 |  | | Central Sub-Saharan Africa | 9118.96 (5176.89,14721.21) | 24356.22 (13944.45,39266.54) | 95.18 (54.42,153.83) | 101.75 (58.71,164.10) | 0.24 (0.20,0.27) | 0 |  | | East Asia | 108313.38 (60124.68,184009.79) | 85575.53 (49139.08,140652.49) | 41.36 (23.04,70.13) | 30.58 (17.40,50.54) | -1.06 (-1.19,-0.92) | 0 |  | | Eastern Europe | 93740.17 (55242.32,152020.42) | 85567.29 (50032.53,140344.09) | 201.28 (118.23,326.50) | 210.11 (122.12,343.76) | 0.16 (0.12,0.21) | 0 |  | | Eastern Sub-Saharan Africa | 30134.67 (17262.29,48769.58) | 74578.92 (42556.80,121245.33) | 91.69 (52.92,148.59) | 96.11 (55.23,156.55) | 0.18 (0.11,0.25) | 0 |  | | High-income Asia Pacific | 28445.29 (16137.09,46918.37) | 19172.51 (11132.82,31132.64) | 85.22 (48.34,140.35) | 71.68 (41.44,116.05) | -0.58 (-0.66,-0.49) | 0 |  | | High-income North America | 115904.48 (67797.45,189709.92) | 106022.24 (64393.29,164607.13) | 190.55 (111.18,312.04) | 166.01 (100.69,257.49) | -0.42 (-0.57,-0.27) | 0 |  | | North Africa and Middle East | 81219.45 (46972.25,130861.92) | 191696.72 (112208.12,309624.23) | 135.62 (78.84,218.81) | 151.84 (88.79,245.26) | 0.40 (0.35,0.45) | 0 |  | | Oceania | 1280.87 (741.56,2057.47) | 3194.71 (1883.10,5076.08) | 108.63 (63.18,174.42) | 123.71 (73.03,196.53) | 0.44 (0.36,0.51) | 0 |  | | South Asia | 186532.19 (108424.17,301908.97) | 431791.23 (253073.16,694503.14) | 96.71 (56.52,156.58) | 118.27 (69.44,190.26) | 0.73 (0.59,0.86) | 0 |  | | Southeast Asia | 22990.12 (12624.71,38417.10) | 35190.10 (19538.03,58736.00) | 24.48 (13.52,41.04) | 25.77 (14.29,42.99) | 0.18 (0.16,0.21) | 0 |  | | Southern Latin America | 5955.25 (3422.87,9770.83) | 7410.26 (4231.76,11941.62) | 62.94 (36.23,103.27) | 56.58 (32.24,91.24) | -0.25 (-0.60,0.10) | 0.163 |  | | Southern Sub-Saharan Africa | 16258.81 (9472.30,26055.32) | 26831.37 (15648.12,43278.15) | 156.97 (91.98,252.24) | 157.92 (92.08,255.28) | 0.06 (-0.03,0.14) | 0.212 |  | | Tropical Latin America | 24130.83 (13759.35,39987.29) | 14322.87 (8325.72,23082.08) | 79.16 (45.29,131.39) | 30.32 (17.55,48.84) | -3.27 (-3.41,-3.13) | 0 |  | | Western Europe | 11319.65 (6368.31,18468.39) | 10756.46 (6109.84,17666.73) | 15.54 (8.72,25.38) | 15.56 (8.78,25.56) | 0.05 (-0.02,0.12) | 0.178 |  | | Western Sub-Saharan Africa | 33505.27 (19181.32,54129.15) | 91423.29 (52362.84,147645.34) | 100.65 (58.02,163.17) | 106.53 (61.38,172.39) | 0.24 (0.19,0.29) | 0 |  | |
| --- | --- | --- | --- | --- | --- | --- | --- | --- | --- | --- | --- | --- | --- | --- | --- | --- | --- | --- | --- | --- | --- | --- | --- | --- | --- | --- | --- | --- | --- | --- | --- | --- | --- | --- | --- | --- | --- | --- | --- | --- | --- | --- | --- | --- | --- | --- | --- | --- | --- | --- | --- | --- | --- | --- | --- | --- | --- | --- | --- | --- | --- | --- | --- | --- | --- | --- | --- | --- | --- | --- | --- | --- | --- | --- | --- | --- | --- | --- | --- | --- | --- | --- | --- | --- | --- | --- | --- | --- | --- | --- | --- | --- | --- | --- | --- | --- | --- | --- | --- | --- | --- | --- | --- | --- | --- | --- | --- | --- | --- | --- | --- | --- | --- | --- | --- | --- | --- | --- | --- | --- | --- | --- | --- | --- | --- | --- | --- | --- | --- | --- | --- | --- | --- | --- | --- | --- | --- | --- | --- | --- | --- | --- | --- | --- | --- | --- | --- | --- | --- | --- | --- | --- | --- | --- | --- | --- | --- | --- | --- | --- | --- | --- | --- | --- | --- | --- | --- | --- | --- | --- | --- | --- | --- | --- | --- | --- | --- | --- | --- | --- | --- | --- | --- | --- | --- | --- | --- | --- | --- | --- | --- | --- | --- | --- | --- | --- | --- | --- | --- | --- | --- | --- | --- | --- | --- | --- | --- | --- | --- | --- | --- | --- | --- | --- | --- | --- | --- | --- | --- | --- | --- | --- | --- | --- | --- | --- | --- | --- | --- | --- | --- | --- |

NOTE:ASIR,age-standardised incidence rate;APPC,average annual percentage change;

| **Table 2. DALYs of interstitial lung disease and pulmonary sarcoidosis in adolescents and young adultsr in 1990 and 2019 for female and all locations, with AAPC from 2009 and 2019** | | | | | | |
| --- | --- | --- | --- | --- | --- | --- |
| location | Numbers in 1990 | Numbers in 2019 | Age-standardized rates in 1990   (95% CI) | Age standardized rates in 2019   (95% CI) | AAPC, %   (95% CI) | P |
| Global | 68897.02 (50581.11,90755.16) | 108031.76 (81252.10,140896.91) | 6.66 (4.89,8.77) | 7.26 (5.46,9.47) | 0.31 (0.12,0.50) | 0.001 |
| High SDI | 15800.22 (11983.75,21167.65) | 15997.80 (12289.88,21074.17) | 9.57 (7.26,12.84) | 9.26 (7.11,12.24) | -0.10 (-0.22,0.01) | 0.072 |
| High-middle SDI | 14303.10 (10395.75,18810.29) | 15295.77 (11358.71,20189.22) | 6.01 (4.37,7.90) | 5.66 (4.19,7.47) | -0.17 (-0.33,-0.01) | 0.034 |
| Middle SDI | 18179.49 (13462.91,25493.88) | 30331.38 (22981.14,40687.14) | 5.28 (3.90,7.40) | 6.35 (4.81,8.52) | 0.68 (0.47,0.89) | 0 |
| Low-middle SDI | 14812.29 (9376.73,22059.34) | 31465.37 (21030.75,45178.71) | 7.47 (4.72,11.08) | 8.93 (5.97,12.83) | 0.69 (0.44,0.95) | 0 |
| Low SDI | 5755.16 (3115.48,9286.95) | 14843.69 (9106.08,22259.30) | 6.63 (3.59,10.61) | 7.34 (4.52,10.96) | 0.43 (0.17,0.70) | 0.001 |
| Andean Latin America | 1560.80 (888.27,2617.16) | 2653.64 (1648.06,3958.45) | 20.86 (11.97,34.91) | 20.79 (12.91,31.02) | 0.01 (-0.28,0.30) | 0.945 |
| Australasia | 125.00 (81.20,200.78) | 275.58 (146.52,429.65) | 2.98 (1.93,4.78) | 5.23 (2.78,8.15) | 1.95 (1.75,2.15) | 0 |
| Caribbean | 472.75 (339.24,655.91) | 982.59 (600.17,1520.58) | 6.75 (4.85,9.36) | 10.70 (6.54,16.57) | 1.60 (1.35,1.85) | 0 |
| Central Asia | 2332.12 (1411.66,3264.19) | 2513.87 (1698.59,3664.72) | 16.94 (10.29,23.66) | 12.97 (8.76,18.97) | -0.90 (-1.06,-0.74) | 0 |
| Central Europe | 2135.53 (1417.96,2871.52) | 1385.47 (1005.21,1963.12) | 8.96 (5.92,12.07) | 7.10 (5.14,10.06) | -0.78 (-0.87,-0.69) | 0 |
| Central Latin America | 3219.44 (2358.83,4728.05) | 6589.37 (4098.51,9018.63) | 9.91 (7.24,14.41) | 12.80 (7.96,17.52) | 0.91 (0.71,1.10) | 0 |
| Central Sub-Saharan Africa | 518.56 (170.93,1171.61) | 1260.64 (553.83,2525.24) | 5.50 (1.80,12.40) | 5.31 (2.33,10.64) | -0.14 (-0.35,0.08) | 0.217 |
| East Asia | 7141.88 (4700.98,12435.11) | 5807.00 (3851.78,8562.01) | 2.71 (1.78,4.72) | 2.12 (1.40,3.13) | -0.87 (-1.06,-0.69) | 0 |
| Eastern Europe | 3479.01 (2257.03,5141.92) | 2467.73 (1561.11,4209.70) | 7.61 (4.94,11.28) | 6.28 (3.96,10.93) | -0.66 (-0.88,-0.44) | 0 |
| Eastern Sub-Saharan Africa | 1551.93 (584.31,2976.72) | 4010.49 (1886.36,6939.04) | 4.78 (1.80,9.13) | 5.19 (2.45,8.96) | 0.28 (0.07,0.49) | 0.009 |
| High-income Asia Pacific | 2306.38 (1593.13,3405.58) | 1704.14 (1193.52,2777.99) | 6.89 (4.76,10.18) | 6.28 (4.41,10.30) | -0.34 (-0.53,-0.15) | 0 |
| High-income North America | 9770.44 (7131.40,12915.44) | 8441.23 (6525.38,11703.03) | 16.10 (11.74,21.32) | 13.31 (10.27,18.47) | -0.63 (-0.76,-0.51) | 0 |
| North Africa and Middle East | 4153.07 (2698.58,6494.67) | 9980.63 (6942.75,15068.30) | 6.76 (4.41,10.57) | 7.95 (5.53,12.01) | 0.56 (0.44,0.69) | 0 |
| Oceania | 382.69 (202.13,745.34) | 899.70 (452.77,1787.62) | 31.96 (16.94,61.68) | 34.64 (17.49,68.61) | 0.27 (0.07,0.47) | 0.007 |
| South Asia | 17613.49 (10368.40,26901.34) | 40791.97 (26411.76,60281.59) | 9.25 (5.46,14.05) | 11.21 (7.25,16.55) | 0.73 (0.41,1.04) | 0 |
| Southeast Asia | 2018.55 (1337.32,3217.71) | 3570.99 (2350.06,5787.32) | 2.21 (1.46,3.54) | 2.60 (1.71,4.20) | 0.55 (0.45,0.65) | 0 |
| Southern Latin America | 1196.22 (764.68,1739.05) | 1608.65 (995.12,2318.64) | 12.59 (8.05,18.29) | 12.35 (7.63,17.83) | -0.07 (-0.35,0.20) | 0.595 |
| Southern Sub-Saharan Africa | 1481.60 (712.82,2251.47) | 1603.52 (858.04,3034.99) | 14.47 (6.94,21.96) | 9.37 (5.03,17.70) | -1.50 (-2.01,-1.00) | 0 |
| Tropical Latin America | 2790.15 (1910.88,3874.58) | 4236.28 (2749.55,5915.45) | 8.91 (6.11,12.35) | 9.16 (5.92,12.77) | 0.08 (-0.30,0.47) | 0.682 |
| Western Europe | 3497.86 (2512.39,5121.30) | 4227.67 (2682.28,5375.88) | 4.81 (3.46,7.06) | 6.21 (3.92,7.89) | 0.88 (0.78,0.99) | 0 |
| Western Sub-Saharan Africa | 1149.53 (652.64,1747.89) | 3020.60 (1855.45,4539.57) | 3.49 (1.98,5.30) | 3.52 (2.17,5.28) | 0.02 (-0.10,0.14) | 0.717 |

NOTE:APPC,average annual percentage change;

| **Table 3. ASMR of interstitial lung disease and pulmonary sarcoidosis in adolescents and young adultsr in 1990 and 2019 for female and all locations, with AAPC from 2009 and 2019** | | | | | | |
| --- | --- | --- | --- | --- | --- | --- |
| location | Numbers in 1990 | Numbers in 2019 | Age-standardized rates in 1990   (95% CI) | Age standardized rates in 2019   (95% CI) | AAPC, %   (95% CI) | P |
| Global | 927.76 (641.49,1237.68) | 1501.61 (1082.68,2055.09) | 0.09 (0.06,0.12) | 0.10 (0.07,0.14) | 0.41 (0.19,0.63) | 0 |
| High SDI | 208.67 (149.77,289.71) | 215.47 (159.53,293.74) | 0.13 (0.09,0.18) | 0.12 (0.09,0.17) | -0.06 (-0.21,0.08) | 0.391 |
| High-middle SDI | 179.67 (123.63,238.56) | 197.44 (139.98,263.57) | 0.08 (0.05,0.10) | 0.07 (0.05,0.10) | -0.10 (-0.34,0.14) | 0.415 |
| Middle SDI | 250.44 (177.20,367.09) | 429.69 (316.46,601.08) | 0.07 (0.05,0.11) | 0.09 (0.07,0.13) | 0.72 (0.48,0.95) | 0 |
| Low-middle SDI | 210.50 (120.69,332.04) | 456.54 (286.79,684.76) | 0.11 (0.06,0.17) | 0.13 (0.08,0.20) | 0.70 (0.34,1.07) | 0 |
| Low SDI | 77.79 (34.16,134.90) | 201.00 (108.38,320.69) | 0.09 (0.04,0.16) | 0.10 (0.06,0.16) | 0.39 (0.09,0.70) | 0.012 |
| Andean Latin America | 24.65 (13.63,42.09) | 42.15 (25.25,64.40) | 0.33 (0.19,0.57) | 0.33 (0.20,0.50) | -0.01 (-0.35,0.33) | 0.946 |
| Australasia | 1.43 (0.84,2.62) | 3.99 (1.81,6.62) | 0.03 (0.02,0.06) | 0.07 (0.03,0.12) | 2.74 (2.25,3.22) | 0 |
| Caribbean | 7.19 (4.99,10.24) | 15.67 (9.06,24.83) | 0.10 (0.07,0.15) | 0.17 (0.10,0.27) | 1.72 (1.47,1.96) | 0 |
| Central Asia | 35.60 (20.20,51.45) | 37.75 (24.04,57.45) | 0.26 (0.15,0.38) | 0.19 (0.12,0.30) | -0.99 (-1.17,-0.81) | 0 |
| Central Europe | 26.12 (15.61,35.22) | 14.67 (10.19,22.13) | 0.11 (0.06,0.15) | 0.08 (0.05,0.11) | -1.28 (-1.38,-1.18) | 0 |
| Central Latin America | 49.55 (35.14,74.42) | 106.48 (63.83,148.06) | 0.15 (0.11,0.23) | 0.21 (0.12,0.29) | 1.08 (0.95,1.20) | 0 |
| Central Sub-Saharan Africa | 6.48 (1.04,17.21) | 15.15 (4.09,36.10) | 0.07 (0.01,0.19) | 0.07 (0.02,0.16) | -0.31 (-0.63,0.00) | 0.051 |
| East Asia | 86.24 (51.20,174.16) | 75.82 (44.04,120.72) | 0.03 (0.02,0.07) | 0.03 (0.02,0.04) | -0.56 (-0.95,-0.17) | 0.005 |
| Eastern Europe | 35.10 (19.05,55.56) | 19.98 (11.57,43.93) | 0.08 (0.04,0.12) | 0.05 (0.03,0.12) | -1.34 (-1.60,-1.08) | 0 |
| Eastern Sub-Saharan Africa | 18.59 (3.94,41.35) | 48.90 (16.32,94.77) | 0.06 (0.01,0.13) | 0.06 (0.02,0.13) | 0.33 (0.03,0.64) | 0.03 |
| High-income Asia Pacific | 23.97 (16.96,37.38) | 17.93 (12.99,34.99) | 0.07 (0.05,0.11) | 0.07 (0.05,0.13) | -0.28 (-0.53,-0.04) | 0.025 |
| High-income North America | 133.70 (91.34,181.76) | 114.18 (84.36,169.33) | 0.22 (0.15,0.30) | 0.18 (0.13,0.27) | -0.68 (-0.79,-0.57) | 0 |
| North Africa and Middle East | 48.30 (29.68,86.32) | 120.26 (78.20,202.14) | 0.08 (0.05,0.14) | 0.10 (0.06,0.16) | 0.72 (0.63,0.81) | 0 |
| Oceania | 5.90 (2.91,11.86) | 13.77 (6.33,28.63) | 0.50 (0.25,1.00) | 0.53 (0.25,1.11) | 0.16 (0.02,0.29) | 0.023 |
| South Asia | 258.55 (137.61,413.34) | 599.81 (357.69,919.57) | 0.14 (0.07,0.22) | 0.17 (0.10,0.25) | 0.68 (0.21,1.15) | 0.005 |
| Southeast Asia | 28.81 (18.04,49.64) | 53.02 (32.70,92.49) | 0.03 (0.02,0.06) | 0.04 (0.02,0.07) | 0.59 (0.46,0.71) | 0 |
| Southern Latin America | 18.43 (11.14,27.59) | 24.83 (14.57,36.62) | 0.19 (0.12,0.29) | 0.19 (0.11,0.28) | -0.08 (-0.41,0.24) | 0.62 |
| Southern Sub-Saharan Africa | 21.12 (8.37,34.14) | 20.71 (9.09,45.39) | 0.21 (0.08,0.34) | 0.12 (0.05,0.26) | -1.90 (-2.59,-1.19) | 0 |
| Tropical Latin America | 40.13 (25.57,57.83) | 68.74 (43.41,98.01) | 0.13 (0.08,0.19) | 0.15 (0.09,0.21) | 0.47 (0.23,0.71) | 0 |
| Western Europe | 47.15 (32.03,73.43) | 60.62 (35.34,76.77) | 0.06 (0.04,0.10) | 0.09 (0.05,0.11) | 1.07 (0.91,1.24) | 0 |
| Western Sub-Saharan Africa | 10.76 (4.45,17.45) | 27.18 (13.39,43.83) | 0.03 (0.01,0.05) | 0.03 (0.02,0.05) | -0.16 (-0.36,0.05) | 0.146 |

NOTE:;ASMR,age-standardised mortality rates;APPC,average annual percentage change;

| **Table 4.ASIR of interstitial lung disease and pulmonary sarcoidosis in adolescents and young adultsr in 1990 and 2019 for male and all locations, with AAPC from 2009 and 2019** | | | | | | |
| --- | --- | --- | --- | --- | --- | --- |
| location | Numbers in 1990 | Numbers in 2019 | Age-standardized rates in 1990   (95% CI) | Age standardized rates in 2019   (95% CI) | AAPC, %   (95% CI) | P |
| Global | 893445.39 (521837.50,1442984.51) | 1328352.15 (785845.63,2114568.54) | 84.35 (49.43,136.17) | 87.40 (51.66,139.23) | 0.13 (0.08,0.19) | 0 |
| High SDI | 168050.73 (98071.86,273494.19) | 185147.86 (112565.17,289833.33) | 98.65 (57.46,160.66) | 98.68 (59.74,154.46) | 0.01 (-0.07,0.08) | 0.876 |
| High-middle SDI | 239424.29 (140272.17,386797.76) | 253542.85 (150554.55,402709.97) | 97.26 (56.98,157.15) | 85.71 (50.63,136.45) | -0.42 (-0.54,-0.29) | 0 |
| Middle SDI | 225458.72 (128808.95,368186.12) | 351988.17 (205561.93,568279.18) | 63.85 (36.68,104.21) | 72.55 (42.28,117.27) | 0.45 (0.34,0.56) | 0 |
| Low-middle SDI | 180721.67 (105350.01,290666.49) | 340333.72 (199035.66,545538.68) | 88.13 (51.71,141.61) | 96.07 (56.35,154.01) | 0.31 (0.25,0.37) | 0 |
| Low SDI | 79442.18 (45912.50,127198.52) | 196626.75 (113854.56,316137.78) | 90.98 (53.01,145.47) | 97.77 (57.05,157.13) | 0.26 (0.21,0.31) | 0 |
| Andean Latin America | 5597.36 (3285.62,8947.63) | 12598.97 (7611.25,19695.17) | 82.26 (48.65,131.63) | 100.37 (60.68,156.97) | 0.70 (0.60,0.81) | 0 |
| Australasia | 3172.23 (1841.56,5202.32) | 3732.55 (2159.10,5992.90) | 76.19 (44.16,124.93) | 72.95 (42.10,117.28) | -0.16 (-0.20,-0.13) | 0 |
| Caribbean | 3373.49 (1900.73,5523.90) | 4933.35 (2831.81,8011.23) | 50.19 (28.47,82.24) | 55.27 (31.75,89.89) | 0.34 (0.31,0.37) | 0 |
| Central Asia | 14689.99 (8649.62,23452.33) | 22589.70 (13356.18,36001.46) | 109.61 (64.76,175.60) | 115.23 (68.04,184.32) | 0.19 (0.14,0.24) | 0 |
| Central Europe | 51490.01 (30477.02,82697.45) | 49893.00 (30607.41,76993.67) | 207.96 (122.75,333.65) | 237.18 (144.95,365.80) | 0.48 (0.35,0.62) | 0 |
| Central Latin America | 21718.75 (12483.49,35655.88) | 31788.50 (18729.77,50827.49) | 72.19 (41.78,118.79) | 65.31 (38.53,104.42) | -0.36 (-0.39,-0.32) | 0 |
| Central Sub-Saharan Africa | 8351.95 (4808.55,13591.91) | 22794.54 (13030.47,36942.70) | 88.24 (51.25,143.76) | 96.01 (55.26,155.38) | 0.30 (0.24,0.35) | 0 |
| East Asia | 122287.43 (68286.49,207256.06) | 92421.22 (53739.04,150393.58) | 43.87 (24.58,74.23) | 31.54 (18.20,51.54) | -1.09 (-1.30,-0.89) | 0 |
| Eastern Europe | 97805.03 (58035.58,155594.05) | 86881.43 (51297.81,139022.72) | 211.20 (124.90,336.56) | 216.66 (127.19,346.99) | 0.11 (0.04,0.19) | 0.003 |
| Eastern Sub-Saharan Africa | 24495.58 (13992.40,39605.59) | 62277.12 (35576.59,101167.18) | 79.71 (45.94,129.05) | 84.27 (48.55,137.11) | 0.20 (0.16,0.25) | 0 |
| High-income Asia Pacific | 35961.35 (20598.60,59227.44) | 29674.49 (17374.27,48403.39) | 104.62 (59.92,171.97) | 101.81 (59.38,165.42) | -0.06 (-0.14,0.02) | 0.137 |
| High-income North America | 100572.10 (59318.51,163822.08) | 101365.19 (62296.76,155432.36) | 165.99 (97.64,270.68) | 158.10 (97.05,242.35) | -0.19 (-0.46,0.08) | 0.169 |
| North Africa and Middle East | 73704.74 (42622.04,119529.98) | 179463.46 (104949.36,288110.04) | 115.10 (66.96,187.00) | 129.19 (75.39,207.47) | 0.41 (0.37,0.46) | 0 |
| Oceania | 2158.09 (1272.15,3389.30) | 5200.18 (3106.89,8229.24) | 171.24 (101.12,268.54) | 194.98 (116.59,308.21) | 0.44 (0.40,0.47) | 0 |
| South Asia | 215938.76 (126708.40,345029.66) | 443651.48 (260306.47,709230.79) | 104.49 (61.64,166.85) | 117.26 (68.96,187.46) | 0.41 (0.37,0.45) | 0 |
| Southeast Asia | 23883.75 (13111.11,39848.09) | 39481.75 (21971.29,66025.89) | 26.09 (14.39,43.60) | 28.39 (15.78,47.44) | 0.30 (0.27,0.32) | 0 |
| Southern Latin America | 5871.16 (3372.58,9588.55) | 8534.15 (4891.21,13818.65) | 63.73 (36.67,104.13) | 66.17 (37.88,107.18) | 0.12 (0.08,0.16) | 0 |
| Southern Sub-Saharan Africa | 14674.41 (8647.15,23260.36) | 25231.07 (14797.32,40429.52) | 150.42 (89.26,238.49) | 149.29 (87.51,239.62) | -0.01 (-0.06,0.04) | 0.601 |
| Tropical Latin America | 22255.40 (12863.76,36098.93) | 13646.95 (7976.62,21850.58) | 75.22 (43.67,122.22) | 29.75 (17.33,47.61) | -3.14 (-3.29,-3.00) | 0 |
| Western Europe | 15850.92 (8921.50,25965.37) | 15032.40 (8597.08,24462.65) | 21.28 (11.96,34.89) | 21.10 (12.01,34.37) | 0.03 (-0.08,0.14) | 0.614 |
| Western Sub-Saharan Africa | 29592.90 (16983.26,47880.34) | 77160.62 (44407.41,123682.02) | 92.45 (53.40,149.64) | 98.63 (57.18,158.14) | 0.24 (0.18,0.29) | 0 |

NOTE:ASIR,age-standardised incidence rate;APPC,average annual percentage change;

| **Table 5. DALYs of interstitial lung disease and pulmonary sarcoidosis in adolescents and young adultsr in 1990 and 2019 for male and all locations, with AAPC from 2009 and 2019** | | | | | | |
| --- | --- | --- | --- | --- | --- | --- |
| location | Numbers in 1990 | Numbers in 2019 | Age-standardized rates in 1990   (95% CI) | Age standardized rates in 2019   (95% CI) | AAPC, %   (95% CI) | P |
| Global | 89757.09 (65350.77,125630.23) | 133118.92 (99293.52,175061.00) | 8.44 (6.14,11.80) | 8.76 (6.54,11.53) | 0.17 (-0.01,0.34) | 0.061 |
| High SDI | 17789.28 (14082.11,24755.58) | 18419.70 (14631.81,23404.41) | 10.47 (8.28,14.61) | 9.91 (7.85,12.59) | -0.19 (-0.33,-0.04) | 0.011 |
| High-middle SDI | 17350.76 (13390.87,22661.58) | 18336.41 (13665.82,23006.84) | 7.05 (5.44,9.20) | 6.34 (4.71,7.94) | -0.37 (-0.57,-0.17) | 0 |
| Middle SDI | 21752.19 (16041.35,31997.46) | 34599.46 (26300.09,44053.44) | 6.11 (4.51,8.96) | 7.15 (5.43,9.11) | 0.59 (0.43,0.75) | 0 |
| Low-middle SDI | 24558.53 (13563.11,42990.22) | 43587.58 (27738.85,67308.29) | 12.04 (6.61,21.10) | 12.35 (7.84,19.10) | 0.12 (-0.14,0.38) | 0.361 |
| Low SDI | 8237.29 (4215.04,14496.25) | 18044.44 (10936.07,27077.67) | 9.50 (4.86,16.72) | 9.02 (5.46,13.54) | -0.17 (-0.37,0.03) | 0.092 |
| Andean Latin America | 2374.30 (1270.11,4074.79) | 3896.82 (2411.22,5831.02) | 33.18 (17.87,56.77) | 30.62 (18.96,45.79) | -0.16 (-0.70,0.37) | 0.552 |
| Australasia | 172.58 (107.14,281.89) | 387.30 (186.91,634.03) | 4.14 (2.57,6.75) | 7.57 (3.62,12.47) | 2.13 (1.51,2.75) | 0 |
| Caribbean | 443.56 (297.01,694.82) | 904.31 (592.00,1399.64) | 6.56 (4.40,10.27) | 10.13 (6.63,15.67) | 1.51 (1.34,1.69) | 0 |
| Central Asia | 1620.00 (1041.04,2267.08) | 2360.02 (1373.62,3370.74) | 11.99 (7.71,16.75) | 12.05 (7.03,17.19) | 0.07 (-0.36,0.50) | 0.765 |
| Central Europe | 2848.79 (1866.79,3947.22) | 1886.91 (1313.91,2585.00) | 11.58 (7.58,16.06) | 9.21 (6.39,12.58) | -0.79 (-0.92,-0.66) | 0 |
| Central Latin America | 2939.51 (2247.24,4319.92) | 7112.31 (3767.94,10029.23) | 9.57 (7.33,14.03) | 14.53 (7.68,20.50) | 1.53 (0.93,2.13) | 0 |
| Central Sub-Saharan Africa | 675.90 (245.13,1509.03) | 1639.70 (751.43,3357.63) | 7.21 (2.61,16.14) | 6.94 (3.17,14.16) | -0.12 (-0.20,-0.05) | 0.001 |
| East Asia | 9224.66 (6301.81,13813.28) | 7648.89 (5458.07,10259.22) | 3.26 (2.23,4.88) | 2.66 (1.89,3.58) | -0.68 (-0.93,-0.43) | 0 |
| Eastern Europe | 3730.32 (2422.68,5345.60) | 2753.91 (1811.82,4039.56) | 8.08 (5.26,11.58) | 6.88 (4.53,10.11) | -0.48 (-1.08,0.12) | 0.118 |
| Eastern Sub-Saharan Africa | 2229.91 (801.45,3998.63) | 5058.83 (2402.70,8012.75) | 7.17 (2.58,12.82) | 6.79 (3.22,10.71) | -0.21 (-0.43,0.00) | 0.05 |
| High-income Asia Pacific | 3496.27 (2100.92,5949.96) | 2684.50 (1863.45,3785.64) | 10.17 (6.10,17.31) | 9.19 (6.37,12.97) | -0.29 (-0.46,-0.13) | 0 |
| High-income North America | 9876.28 (7455.57,12520.55) | 8155.86 (6341.61,10782.23) | 16.31 (12.31,20.73) | 12.79 (9.93,16.91) | -0.83 (-1.14,-0.53) | 0 |
| North Africa and Middle East | 4631.12 (2843.62,7697.76) | 10878.59 (7636.13,16078.58) | 7.07 (4.37,11.77) | 7.85 (5.51,11.62) | 0.35 (0.13,0.57) | 0.002 |
| Oceania | 753.66 (411.65,1347.89) | 1580.74 (857.09,2825.77) | 56.81 (31.25,101.06) | 57.68 (31.39,102.90) | 0.04 (-0.03,0.11) | 0.274 |
| South Asia | 30504.79 (15672.34,54511.48) | 55478.33 (33584.29,87130.19) | 14.85 (7.61,26.53) | 14.70 (8.89,23.09) | -0.02 (-0.31,0.27) | 0.897 |
| Southeast Asia | 2878.32 (1701.64,5949.46) | 4625.03 (3110.02,7776.27) | 3.02 (1.79,6.19) | 3.35 (2.25,5.65) | 0.39 (0.20,0.58) | 0 |
| Southern Latin America | 1000.01 (655.12,1545.74) | 1573.78 (925.23,2362.78) | 10.83 (7.11,16.75) | 12.22 (7.17,18.38) | 0.42 (-0.11,0.94) | 0.122 |
| Southern Sub-Saharan Africa | 1287.24 (785.06,1787.04) | 1729.65 (1180.42,2628.47) | 13.84 (8.40,19.20) | 10.16 (6.93,15.43) | -1.15 (-1.87,-0.43) | 0.002 |
| Tropical Latin America | 3518.84 (2429.63,4634.35) | 4774.22 (3076.17,6153.85) | 11.84 (8.17,15.60) | 10.44 (6.72,13.46) | -0.44 (-0.79,-0.09) | 0.015 |
| Western Europe | 4423.25 (3215.25,7168.89) | 5316.73 (3111.99,6759.03) | 5.94 (4.32,9.63) | 7.47 (4.32,9.51) | 0.75 (0.54,0.97) | 0 |
| Western Sub-Saharan Africa | 1127.78 (612.53,1743.41) | 2672.50 (1715.75,3964.13) | 3.55 (1.93,5.47) | 3.46 (2.22,5.10) | -0.10 (-0.23,0.03) | 0.131 |

NOTE:APPC,average annual percentage change;

| **Table 6. ASMR of interstitial lung disease and pulmonary sarcoidosis in adolescents and young adultsr in 1990 and 2019 for male and all locations, with AAPC from 2009 and 2019** | | | | | | |
| --- | --- | --- | --- | --- | --- | --- |
| location | Numbers in 1990 | Numbers in 2019 | Age-standardized rates in 1990   (95% CI) | Age standardized rates in 2019   (95% CI) | AAPC, %   (95% CI) | P |
| Global | 1272.37 (884.72,1831.56) | 1926.42 (1379.55,2582.66) | 0.12 (0.08,0.17) | 0.13 (0.09,0.17) | 0.22 (0.00,0.43) | 0.052 |
| High SDI | 242.36 (193.93,353.55) | 251.89 (197.55,321.42) | 0.14 (0.11,0.21) | 0.13 (0.10,0.17) | -0.19 (-0.42,0.04) | 0.103 |
| High-middle SDI | 227.25 (170.48,299.83) | 245.91 (172.76,301.62) | 0.09 (0.07,0.12) | 0.08 (0.06,0.10) | -0.31 (-0.60,-0.02) | 0.033 |
| Middle SDI | 306.77 (214.63,466.41) | 502.55 (366.16,652.78) | 0.09 (0.06,0.13) | 0.10 (0.08,0.13) | 0.62 (0.26,0.98) | 0.001 |
| Low-middle SDI | 375.60 (185.50,691.39) | 669.49 (395.91,1084.62) | 0.19 (0.09,0.34) | 0.19 (0.11,0.31) | 0.10 (-0.21,0.41) | 0.527 |
| Low SDI | 119.38 (51.88,225.71) | 254.67 (136.97,405.62) | 0.14 (0.06,0.27) | 0.13 (0.07,0.21) | -0.28 (-0.53,-0.03) | 0.03 |
| Andean Latin America | 38.06 (19.84,66.23) | 62.66 (38.15,95.14) | 0.54 (0.28,0.94) | 0.49 (0.30,0.75) | -0.20 (-0.74,0.35) | 0.482 |
| Australasia | 2.16 (1.20,3.99) | 5.76 (2.41,9.94) | 0.05 (0.03,0.10) | 0.11 (0.05,0.19) | 2.75 (1.98,3.52) | 0 |
| Caribbean | 6.62 (4.23,10.95) | 14.18 (9.03,22.67) | 0.10 (0.06,0.16) | 0.16 (0.10,0.25) | 1.63 (1.43,1.82) | 0 |
| Central Asia | 23.72 (14.09,34.25) | 35.17 (18.35,52.40) | 0.18 (0.11,0.26) | 0.18 (0.09,0.27) | 0.09 (-0.41,0.58) | 0.731 |
| Central Europe | 35.60 (21.58,50.90) | 19.27 (12.11,25.56) | 0.14 (0.09,0.21) | 0.09 (0.06,0.12) | -1.51 (-1.80,-1.22) | 0 |
| Central Latin America | 43.62 (32.72,66.61) | 113.01 (56.06,162.60) | 0.14 (0.11,0.22) | 0.23 (0.11,0.33) | 1.73 (1.07,2.39) | 0 |
| Central Sub-Saharan Africa | 9.26 (2.25,23.41) | 21.85 (7.68,50.06) | 0.10 (0.02,0.26) | 0.09 (0.03,0.21) | -0.30 (-0.44,-0.15) | 0 |
| East Asia | 117.52 (74.56,189.17) | 106.52 (70.36,148.03) | 0.04 (0.03,0.07) | 0.04 (0.02,0.05) | -0.41 (-0.74,-0.07) | 0.017 |
| Eastern Europe | 38.70 (23.58,58.34) | 24.75 (16.02,37.69) | 0.08 (0.05,0.13) | 0.06 (0.04,0.09) | -1.01 (-1.99,-0.02) | 0.045 |
| Eastern Sub-Saharan Africa | 30.83 (7.82,59.88) | 68.67 (25.53,116.06) | 0.10 (0.03,0.20) | 0.09 (0.03,0.16) | -0.26 (-0.48,-0.04) | 0.021 |
| High-income Asia Pacific | 38.11 (22.52,74.05) | 27.38 (20.31,39.39) | 0.11 (0.07,0.22) | 0.09 (0.07,0.14) | -0.53 (-0.77,-0.29) | 0 |
| High-income North America | 142.37 (104.63,183.71) | 112.34 (85.91,155.57) | 0.23 (0.17,0.30) | 0.18 (0.13,0.24) | -0.96 (-1.26,-0.66) | 0 |
| North Africa and Middle East | 58.11 (31.54,108.47) | 139.83 (93.79,223.10) | 0.09 (0.05,0.17) | 0.10 (0.07,0.16) | 0.36 (0.07,0.65) | 0.016 |
| Oceania | 11.12 (5.67,20.70) | 22.75 (11.17,43.17) | 0.85 (0.44,1.58) | 0.84 (0.41,1.59) | -0.08 (-0.15,0.00) | 0.05 |
| South Asia | 473.77 (215.12,883.66) | 854.35 (477.63,1401.05) | 0.23 (0.11,0.44) | 0.23 (0.13,0.37) | -0.08 (-0.42,0.27) | 0.665 |
| Southeast Asia | 40.69 (22.45,88.10) | 66.58 (43.44,118.31) | 0.04 (0.02,0.09) | 0.05 (0.03,0.09) | 0.42 (0.20,0.64) | 0 |
| Southern Latin America | 15.39 (9.70,24.73) | 24.43 (13.62,37.54) | 0.17 (0.11,0.27) | 0.19 (0.11,0.29) | 0.49 (0.23,0.75) | 0 |
| Southern Sub-Saharan Africa | 18.66 (10.02,26.66) | 23.73 (15.05,39.03) | 0.20 (0.11,0.29) | 0.14 (0.09,0.23) | -1.46 (-2.49,-0.42) | 0.006 |
| Tropical Latin America | 54.15 (35.98,73.26) | 79.22 (49.90,103.45) | 0.18 (0.12,0.25) | 0.17 (0.11,0.22) | -0.23 (-0.62,0.16) | 0.249 |
| Western Europe | 62.60 (44.85,108.56) | 78.90 (41.35,100.15) | 0.08 (0.06,0.15) | 0.11 (0.06,0.14) | 0.87 (0.66,1.08) | 0 |
| Western Sub-Saharan Africa | 11.31 (4.60,19.27) | 25.06 (14.50,38.23) | 0.04 (0.01,0.06) | 0.03 (0.02,0.05) | -0.30 (-0.42,-0.17) | 0 |

NOTE:;ASMR,age-standardised mortality rates;APPC,average annual percentage change;

| **Table 7.ASIR of interstitial lung disease and pulmonary sarcoidosis in adolescents and young adultsr in 1990 and 2019 for female and all nation, with AAPC from 2009 and 2019** | | | | | | |
| --- | --- | --- | --- | --- | --- | --- |
| location | Numbers in 1990 | Numbers in 2019 | Age-standardized rates in 1990   (95% CI) | Age standardized rates in 2019   (95% CI) | AAPC, %   (95% CI) | P |
| Afghanistan | 2213.33 (1263.58,3599.98) | 8806.28 (5024.24,14244.98) | 131.23 (75.43,211.61) | 142.78 (82.11,232.64) | 0.30 (0.27,0.33) | 0 |
| Albania | 1007.31 (584.46,1649.79) | 836.41 (480.43,1368.44) | 153.51 (89.20,253.02) | 180.07 (103.41,294.95) | 0.54 (0.48,0.60) | 0 |
| Algeria | 5837.93 (3334.20,9421.36) | 13520.11 (7803.96,22205.40) | 133.46 (76.77,215.47) | 148.08 (85.27,242.99) | 0.37 (0.34,0.40) | 0 |
| American Samoa | 8.89 (5.05,14.88) | 9.65 (5.52,16.14) | 95.16 (54.34,159.37) | 99.32 (57.12,165.89) | 0.15 (0.12,0.18) | 0 |
| Andorra | 1.63 (0.92,2.74) | 1.89 (1.06,3.17) | 13.36 (7.47,22.49) | 13.39 (7.46,22.45) | 0.03 (-0.07,0.14) | 0.525 |
| Angola | 1709.11 (971.23,2749.68) | 5546.48 (3157.45,8955.10) | 94.74 (54.14,152.70) | 102.72 (58.87,165.80) | 0.29 (0.23,0.34) | 0 |
| Antigua and Barbuda | 6.39 (3.57,10.61) | 10.17 (5.83,16.95) | 49.71 (27.84,82.66) | 55.34 (31.64,92.08) | 0.37 (0.35,0.39) | 0 |
| Argentina | 3875.36 (2219.57,6424.62) | 5076.56 (2883.90,8204.65) | 63.73 (36.54,105.59) | 56.65 (32.13,91.57) | -0.35 (-0.55,-0.14) | 0.001 |
| Armenia | 789.05 (461.26,1267.96) | 745.82 (431.10,1224.42) | 106.61 (62.30,172.01) | 116.92 (67.29,192.13) | 0.33 (0.29,0.36) | 0 |
| Australia | 2319.22 (1322.71,3818.47) | 2976.90 (1703.99,4873.45) | 66.67 (37.96,109.70) | 66.95 (38.13,109.85) | 0.03 (0.00,0.06) | 0.079 |
| Austria | 333.38 (191.96,529.78) | 274.81 (155.69,445.46) | 22.24 (12.79,35.39) | 18.39 (10.35,29.88) | -0.66 (-1.00,-0.31) | 0 |
| Azerbaijan | 1713.16 (1009.71,2696.86) | 2666.86 (1571.67,4315.22) | 111.40 (65.96,176.08) | 117.35 (68.93,190.40) | 0.18 (0.16,0.21) | 0 |
| Bahamas | 32.58 (19.00,52.99) | 53.70 (31.30,88.15) | 57.84 (33.87,94.31) | 68.29 (39.75,112.05) | 0.55 (0.47,0.64) | 0 |
| Bahrain | 133.88 (77.15,213.04) | 424.76 (248.17,696.52) | 135.64 (78.23,217.70) | 154.16 (89.34,250.96) | 0.44 (0.42,0.46) | 0 |
| Bangladesh | 13670.28 (7865.26,22334.18) | 29567.83 (16786.97,48048.54) | 75.41 (43.75,123.30) | 87.14 (49.55,141.76) | 0.49 (0.45,0.53) | 0 |
| Barbados | 29.37 (16.56,48.00) | 32.30 (18.71,53.10) | 52.45 (29.55,85.87) | 60.47 (34.95,99.26) | 0.47 (0.38,0.56) | 0 |
| Belarus | 3916.34 (2263.68,6447.13) | 3625.12 (2063.24,5953.61) | 185.55 (107.03,305.99) | 200.63 (113.70,329.26) | 0.28 (0.23,0.33) | 0 |
| Belgium | 334.03 (187.85,545.77) | 357.64 (198.82,595.65) | 17.54 (9.82,28.71) | 19.28 (10.66,32.15) | 0.43 (0.02,0.83) | 0.039 |
| Belize | 18.56 (10.63,30.69) | 65.30 (38.73,105.19) | 59.88 (34.60,99.20) | 76.66 (45.63,123.39) | 0.84 (0.75,0.92) | 0 |
| Benin | 832.21 (473.49,1347.68) | 2338.21 (1324.69,3769.92) | 96.38 (55.11,156.29) | 101.63 (57.95,163.98) | 0.19 (0.17,0.21) | 0 |
| Bermuda | 8.06 (4.64,13.40) | 6.67 (3.90,11.00) | 56.71 (32.49,94.48) | 62.31 (36.16,102.58) | 0.30 (0.24,0.35) | 0 |
| Bhutan | 73.42 (41.72,119.30) | 141.03 (81.20,232.83) | 71.42 (40.86,116.17) | 86.59 (49.89,143.18) | 0.65 (0.62,0.69) | 0 |
| Bolivia (Plurinational State of) | 779.36 (446.97,1285.32) | 1823.83 (1069.59,2961.43) | 67.53 (38.92,111.32) | 78.22 (45.96,127.11) | 0.51 (0.50,0.52) | 0 |
| Bosnia and Herzegovina | 1457.65 (852.94,2369.66) | 1007.51 (584.62,1654.36) | 154.27 (90.21,250.94) | 179.85 (104.02,294.52) | 0.54 (0.45,0.63) | 0 |
| Botswana | 347.22 (199.21,560.13) | 830.18 (485.41,1349.43) | 141.54 (81.71,229.12) | 152.27 (88.91,247.80) | 0.30 (0.22,0.39) | 0 |
| Brazil | 23872.27 (13613.61,39562.69) | 13780.76 (8021.50,22195.14) | 80.18 (45.89,133.10) | 30.09 (17.44,48.44) | -3.34 (-3.49,-3.20) | 0 |
| Brunei Darussalam | 44.60 (25.16,73.34) | 64.82 (36.63,107.42) | 78.43 (44.29,129.43) | 67.62 (38.12,112.27) | -0.46 (-0.62,-0.31) | 0 |
| Bulgaria | 2417.83 (1413.68,3996.94) | 2011.24 (1182.91,3238.64) | 154.79 (90.43,255.11) | 178.87 (104.52,287.90) | 0.50 (0.47,0.53) | 0 |
| Burkina Faso | 1535.01 (875.90,2469.63) | 4254.40 (2418.10,6891.55) | 95.63 (54.87,153.93) | 102.02 (58.30,165.13) | 0.25 (0.21,0.29) | 0 |
| Burundi | 879.36 (507.11,1429.01) | 2012.25 (1147.74,3282.70) | 88.96 (51.62,144.73) | 93.11 (53.47,152.22) | 0.16 (0.14,0.18) | 0 |
| Cabo Verde | 59.63 (33.86,96.45) | 122.62 (70.12,199.38) | 96.99 (55.43,157.08) | 103.01 (58.96,168.02) | 0.22 (0.17,0.27) | 0 |
| Cambodia | 419.43 (228.46,714.37) | 777.67 (422.48,1323.81) | 21.59 (11.84,36.89) | 22.15 (12.03,37.80) | 0.09 (0.07,0.11) | 0 |
| Cameroon | 1746.20 (1006.23,2852.19) | 5854.43 (3311.16,9546.21) | 96.40 (55.97,157.88) | 102.44 (58.28,167.48) | 0.23 (0.19,0.27) | 0 |
| Canada | 8729.62 (5034.91,14495.14) | 8588.75 (4888.85,14574.28) | 144.01 (82.77,239.60) | 136.02 (77.23,230.24) | -0.12 (-0.35,0.11) | 0.321 |
| Central African Republic | 467.55 (269.63,754.93) | 1013.50 (579.50,1632.97) | 94.83 (55.09,153.44) | 100.53 (57.88,161.66) | 0.22 (0.13,0.31) | 0 |
| Chad | 981.16 (562.79,1613.27) | 2732.62 (1552.23,4485.67) | 95.76 (55.20,157.53) | 101.44 (58.27,167.04) | 0.22 (0.13,0.30) | 0 |
| Chile | 1728.54 (970.97,2785.25) | 1975.29 (1122.49,3208.60) | 61.50 (34.63,99.14) | 56.27 (31.87,91.59) | -0.32 (-0.41,-0.24) | 0 |
| China | 106638.58 (59201.27,181108.89) | 83305.45 (47804.21,136859.94) | 42.11 (23.47,71.37) | 30.80 (17.52,50.90) | -1.10 (-1.22,-0.98) | 0 |
| Colombia | 2816.79 (1526.36,4712.74) | 4580.08 (2562.69,7642.81) | 42.62 (23.13,71.54) | 47.26 (26.43,78.83) | 0.36 (0.33,0.39) | 0 |
| Comoros | 70.36 (39.62,115.65) | 131.28 (75.59,212.76) | 89.71 (50.82,147.77) | 94.07 (54.41,152.62) | 0.18 (0.12,0.24) | 0 |
| Congo | 416.84 (236.03,674.79) | 1088.32 (634.37,1775.57) | 95.81 (54.76,155.19) | 102.23 (59.67,166.77) | 0.23 (0.19,0.28) | 0 |
| Cook Islands | 3.47 (1.98,5.63) | 3.32 (1.88,5.44) | 98.60 (56.61,160.14) | 107.12 (60.78,175.52) | 0.28 (0.23,0.32) | 0 |
| Costa Rica | 287.13 (161.23,478.97) | 541.34 (305.59,911.92) | 47.61 (26.84,79.76) | 52.74 (29.71,88.93) | 0.30 (0.20,0.40) | 0 |
| Coted'Ivoire | 2020.06 (1150.10,3269.00) | 5163.47 (2952.79,8395.67) | 95.85 (55.01,155.64) | 101.51 (58.17,165.40) | 0.22 (0.14,0.30) | 0 |
| Croatia | 1706.85 (975.98,2828.47) | 1369.82 (812.49,2163.98) | 177.27 (101.24,293.13) | 193.03 (113.97,304.19) | 0.30 (0.25,0.36) | 0 |
| Cuba | 1119.45 (635.17,1837.78) | 1006.06 (562.45,1683.58) | 50.20 (28.65,82.44) | 55.03 (30.67,92.33) | 0.31 (0.30,0.32) | 0 |
| Cyprus | 17.73 (9.80,29.82) | 32.75 (18.04,54.03) | 11.64 (6.42,19.60) | 11.61 (6.31,19.27) | -0.01 (-0.04,0.02) | 0.606 |
| Czechia | 2730.06 (1602.36,4471.21) | 3119.36 (1806.95,5205.41) | 140.80 (82.21,229.10) | 178.91 (103.21,297.26) | 0.83 (0.79,0.87) | 0 |
| Democratic People's Republic of Korea | 837.04 (455.64,1430.19) | 1037.75 (565.98,1763.47) | 20.89 (11.44,35.74) | 21.33 (11.61,36.27) | 0.07 (0.06,0.08) | 0 |
| Democratic Republic of the Congo | 6291.06 (3561.38,10187.20) | 16051.31 (9103.79,25933.08) | 95.29 (54.33,154.34) | 101.39 (57.98,163.96) | 0.22 (0.18,0.27) | 0 |
| Denmark | 72.38 (40.35,117.78) | 76.03 (41.24,125.30) | 7.56 (4.21,12.30) | 8.46 (4.58,13.94) | 0.39 (0.10,0.68) | 0.009 |
| Djibouti | 72.51 (41.67,117.76) | 240.30 (136.60,390.02) | 90.31 (52.36,146.63) | 94.43 (53.54,153.48) | 0.16 (0.14,0.19) | 0 |
| Dominica | 6.41 (3.62,10.52) | 7.23 (4.16,11.84) | 51.35 (29.30,84.25) | 57.40 (33.03,94.14) | 0.37 (0.35,0.40) | 0 |
| Dominican Republic | 699.56 (393.48,1154.87) | 1206.57 (683.08,2036.38) | 49.00 (27.87,80.89) | 55.64 (31.55,94.08) | 0.44 (0.43,0.45) | 0 |
| Ecuador | 1015.14 (590.70,1622.70) | 2629.46 (1601.56,4083.29) | 53.17 (31.09,85.07) | 75.22 (45.86,116.84) | 1.22 (1.15,1.28) | 0 |
| Egypt | 13176.70 (7597.31,21458.82) | 29754.72 (17186.49,48196.91) | 132.65 (76.81,215.84) | 151.35 (87.49,245.27) | 0.46 (0.43,0.50) | 0 |
| El Salvador | 406.35 (223.32,684.76) | 603.90 (337.65,1003.80) | 43.01 (23.85,72.65) | 46.83 (26.22,77.86) | 0.29 (0.25,0.33) | 0 |
| Equatorial Guinea | 71.66 (41.05,115.40) | 273.01 (156.01,442.53) | 93.31 (53.72,150.02) | 104.06 (59.93,168.84) | 0.39 (0.34,0.44) | 0 |
| Eritrea | 460.40 (264.84,741.19) | 1212.21 (685.95,2005.04) | 87.59 (50.67,141.10) | 93.96 (53.46,156.04) | 0.25 (0.23,0.28) | 0 |
| Estonia | 565.49 (327.30,907.37) | 456.06 (268.07,741.69) | 185.84 (107.27,298.22) | 202.99 (118.70,330.05) | 0.32 (0.28,0.36) | 0 |
| Eswatini | 204.31 (116.42,326.87) | 368.37 (209.28,592.58) | 140.31 (80.57,224.35) | 148.58 (84.59,239.85) | 0.21 (0.13,0.28) | 0 |
| Ethiopia | 8460.75 (4854.68,13703.81) | 20177.10 (11483.04,32667.30) | 94.13 (54.38,152.36) | 98.62 (56.57,159.81) | 0.21 (0.09,0.32) | 0 |
| Fiji | 136.23 (76.40,229.13) | 168.60 (95.15,286.20) | 89.73 (50.44,151.50) | 95.14 (53.64,161.30) | 0.20 (0.18,0.23) | 0 |
| Finland | 144.34 (81.31,235.15) | 131.22 (73.04,220.05) | 15.00 (8.40,24.48) | 15.12 (8.37,25.37) | 0.05 (-0.01,0.12) | 0.083 |
| France | 1515.40 (837.64,2485.99) | 1382.15 (755.48,2327.98) | 13.36 (7.37,21.94) | 13.21 (7.19,22.22) | -0.02 (-0.10,0.05) | 0.57 |
| Gabon | 162.75 (93.18,264.31) | 383.59 (219.28,620.46) | 95.73 (55.27,155.76) | 102.91 (59.05,166.61) | 0.26 (0.23,0.29) | 0 |
| Gambia | 170.95 (97.26,273.88) | 455.36 (261.66,735.39) | 96.17 (54.98,154.74) | 101.82 (58.82,164.52) | 0.21 (0.19,0.24) | 0 |
| Georgia | 1137.17 (664.49,1851.79) | 553.20 (328.09,872.35) | 102.01 (59.56,166.54) | 86.07 (50.68,135.71) | -0.58 (-0.63,-0.53) | 0 |
| Germany | 1971.39 (1094.34,3274.55) | 1695.89 (941.30,2792.55) | 12.95 (7.16,21.58) | 12.85 (7.08,21.21) | 0.06 (-0.03,0.14) | 0.172 |
| Ghana | 2649.52 (1519.43,4325.19) | 6930.15 (3997.99,11309.35) | 96.33 (55.63,157.81) | 102.05 (59.09,166.82) | 0.23 (0.20,0.26) | 0 |
| Greece | 197.13 (105.36,326.55) | 176.21 (97.70,300.05) | 10.39 (5.55,17.23) | 10.94 (5.99,18.56) | 0.18 (0.15,0.21) | 0 |
| Greenland | 16.79 (9.67,27.54) | 13.83 (7.88,23.03) | 140.59 (81.08,232.17) | 134.37 (76.56,223.96) | 0.00 (-0.50,0.50) | 0.997 |
| Grenada | 8.15 (4.60,13.44) | 12.16 (7.07,19.75) | 53.82 (30.55,88.92) | 65.52 (38.16,106.50) | 0.67 (0.63,0.71) | 0 |
| Guam | 40.04 (23.80,63.40) | 38.54 (22.63,62.45) | 142.56 (84.87,225.87) | 138.60 (81.50,224.39) | -0.12 (-0.24,-0.01) | 0.036 |
| Guatemala | 596.18 (336.03,998.35) | 1752.29 (996.48,2998.13) | 44.55 (25.29,74.57) | 46.21 (26.37,79.27) | 0.12 (0.10,0.15) | 0 |
| Guinea | 1049.82 (599.27,1712.82) | 2459.35 (1403.84,3991.24) | 95.48 (54.78,156.11) | 101.65 (58.24,165.22) | 0.24 (0.18,0.30) | 0 |
| Guinea-Bissau | 175.57 (100.10,284.15) | 402.99 (228.42,649.51) | 95.27 (54.66,154.09) | 102.06 (58.14,164.97) | 0.25 (0.21,0.28) | 0 |
| Guyana | 81.04 (46.24,133.94) | 95.56 (55.35,157.97) | 52.06 (29.87,86.46) | 63.02 (36.66,104.23) | 0.65 (0.63,0.67) | 0 |
| Haiti | 587.51 (329.79,983.13) | 1514.44 (873.81,2532.15) | 49.16 (27.74,82.39) | 55.17 (31.86,92.38) | 0.39 (0.37,0.42) | 0 |
| Honduras | 361.60 (201.41,596.41) | 1002.32 (574.57,1698.38) | 46.65 (26.23,76.93) | 49.74 (28.65,84.38) | 0.20 (0.15,0.26) | 0 |
| Hungary | 3106.21 (1800.53,5136.13) | 2844.65 (1664.65,4699.24) | 154.36 (88.98,254.36) | 180.03 (104.86,296.09) | 0.55 (0.48,0.62) | 0 |
| Iceland | 11.22 (6.28,18.76) | 14.65 (8.22,24.01) | 21.85 (12.22,36.59) | 24.45 (13.70,40.12) | 0.48 (0.34,0.62) | 0 |
| India | 157354.45 (91786.05,253783.20) | 363996.16 (213964.05,584571.41) | 102.04 (59.78,164.61) | 128.42 (75.58,206.29) | 0.83 (0.69,0.97) | 0 |
| Indonesia | 9918.99 (5470.53,16525.05) | 14936.98 (8300.77,24978.91) | 26.73 (14.83,44.70) | 28.06 (15.57,46.83) | 0.17 (0.14,0.21) | 0 |
| Iran (Islamic Republic of) | 13379.97 (7724.63,21585.44) | 29328.36 (17081.52,47640.71) | 133.85 (77.71,216.55) | 147.78 (85.54,239.67) | 0.37 (0.32,0.42) | 0 |
| Iraq | 3780.52 (2137.21,6158.68) | 12350.40 (7249.79,19975.65) | 132.59 (75.50,215.81) | 147.04 (86.80,238.55) | 0.36 (0.33,0.40) | 0 |
| Ireland | 79.70 (44.26,132.60) | 110.61 (61.78,187.23) | 11.88 (6.61,19.75) | 12.44 (6.89,21.01) | 0.15 (-0.17,0.47) | 0.347 |
| Israel | 66.02 (36.35,109.81) | 118.62 (64.66,201.35) | 6.93 (3.82,11.52) | 7.32 (3.98,12.41) | 0.20 (0.14,0.26) | 0 |
| Italy | 2660.25 (1498.34,4384.40) | 1854.40 (1077.37,2973.47) | 25.07 (14.11,41.33) | 21.91 (12.66,35.15) | -0.43 (-0.48,-0.38) | 0 |
| Jamaica | 231.11 (128.35,382.08) | 330.30 (189.60,546.41) | 51.22 (28.55,85.00) | 55.52 (31.87,91.98) | 0.27 (0.26,0.29) | 0 |
| Japan | 24053.02 (13684.34,39875.20) | 14864.60 (8705.98,24048.85) | 107.56 (61.18,177.53) | 88.01 (51.29,141.98) | -0.68 (-0.76,-0.61) | 0 |
| Jordan | 832.12 (484.66,1333.53) | 3295.47 (1877.79,5282.71) | 139.11 (81.89,222.49) | 152.16 (86.94,243.59) | 0.32 (0.29,0.35) | 0 |
| Kazakhstan | 3471.73 (2043.70,5572.78) | 4177.47 (2417.68,6853.35) | 101.88 (59.95,163.96) | 111.13 (64.08,182.86) | 0.31 (0.28,0.33) | 0 |
| Kenya | 3837.66 (2190.81,6197.08) | 10379.46 (5984.64,16808.27) | 98.07 (56.60,158.93) | 101.71 (58.95,165.05) | 0.14 (0.10,0.18) | 0 |
| Kiribati | 12.91 (7.25,21.62) | 22.59 (12.76,37.66) | 88.14 (49.65,148.00) | 92.91 (52.55,155.11) | 0.19 (-0.01,0.39) | 0.061 |
| Kuwait | 485.04 (279.67,779.14) | 1825.33 (1053.78,3006.42) | 135.56 (78.19,218.91) | 149.36 (85.65,245.42) | 0.34 (0.32,0.35) | 0 |
| Kyrgyzstan | 844.55 (485.81,1381.09) | 1434.33 (819.87,2348.45) | 98.58 (56.86,162.04) | 109.39 (62.53,179.66) | 0.38 (0.33,0.42) | 0 |
| Lao People's Democratic Republic | 160.56 (87.21,270.12) | 347.05 (192.11,580.01) | 21.71 (11.90,36.58) | 23.03 (12.79,38.60) | 0.21 (0.19,0.22) | 0 |
| Latvia | 1071.90 (612.43,1744.96) | 738.73 (427.34,1205.33) | 212.58 (121.22,346.52) | 231.98 (133.63,378.97) | 0.37 (0.24,0.49) | 0 |
| Lebanon | 759.80 (432.58,1223.52) | 1661.65 (969.65,2670.61) | 133.02 (75.92,214.43) | 148.15 (85.99,239.02) | 0.38 (0.34,0.42) | 0 |
| Lesotho | 440.52 (254.91,712.95) | 661.68 (382.98,1068.15) | 138.60 (80.70,224.91) | 148.80 (86.34,240.64) | 0.26 (0.19,0.32) | 0 |
| Liberia | 331.90 (190.48,541.55) | 975.47 (556.59,1585.05) | 96.31 (55.49,156.94) | 102.63 (58.87,166.58) | 0.24 (0.19,0.29) | 0 |
| Libya | 860.16 (495.32,1386.19) | 2233.86 (1299.71,3650.63) | 134.27 (78.06,216.67) | 146.01 (84.72,238.33) | 0.29 (0.27,0.32) | 0 |
| Lithuania | 1147.98 (670.90,1864.92) | 771.06 (444.25,1275.56) | 160.83 (93.86,262.03) | 173.43 (99.64,287.28) | 0.25 (0.19,0.30) | 0 |
| Luxembourg | 10.14 (5.59,16.81) | 15.28 (8.54,25.70) | 13.01 (7.13,21.63) | 13.20 (7.31,22.22) | 0.06 (-0.08,0.20) | 0.402 |
| Madagascar | 1877.35 (1083.55,3044.70) | 4768.28 (2741.93,7777.64) | 91.14 (53.03,147.79) | 94.65 (54.65,154.63) | 0.14 (0.10,0.18) | 0 |
| Malawi | 1491.74 (847.77,2408.29) | 3339.24 (1886.01,5505.12) | 89.58 (51.31,144.73) | 93.77 (53.34,154.64) | 0.18 (0.13,0.23) | 0 |
| Malaysia | 803.95 (439.39,1362.65) | 1581.37 (873.98,2644.15) | 22.68 (12.43,38.51) | 24.28 (13.41,40.68) | 0.23 (0.19,0.27) | 0 |
| Maldives | 12.98 (7.39,21.00) | 44.10 (24.97,74.63) | 40.04 (23.11,64.57) | 44.96 (25.35,76.16) | 0.38 (0.30,0.47) | 0 |
| Mali | 1404.91 (794.47,2299.37) | 3869.97 (2223.19,6259.48) | 95.56 (54.42,156.88) | 102.07 (59.22,165.63) | 0.25 (0.21,0.29) | 0 |
| Malta | 9.65 (5.39,16.06) | 10.27 (5.71,17.00) | 13.37 (7.41,22.26) | 13.92 (7.67,23.07) | 0.18 (0.12,0.24) | 0 |
| Marshall Islands | 7.96 (4.59,12.83) | 12.33 (7.06,20.07) | 102.27 (59.23,165.09) | 107.14 (61.38,174.14) | 0.15 (0.09,0.20) | 0 |
| Mauritania | 352.36 (199.46,568.88) | 784.13 (446.63,1263.74) | 96.53 (54.88,155.85) | 102.66 (58.80,165.48) | 0.22 (0.19,0.24) | 0 |
| Mauritius | 58.28 (32.13,99.73) | 69.62 (39.31,116.18) | 24.01 (13.24,41.19) | 28.74 (16.16,47.94) | 0.59 (0.54,0.64) | 0 |
| Mexico | 11204.26 (6379.88,18710.67) | 11768.16 (6942.36,18819.57) | 69.53 (39.95,116.44) | 45.90 (27.07,73.40) | -1.42 (-1.57,-1.27) | 0 |
| Micronesia (Federated States of) | 18.54 (10.73,30.05) | 21.13 (11.99,34.42) | 102.02 (59.27,165.30) | 110.22 (62.76,179.52) | 0.25 (0.20,0.30) | 0 |
| Monaco | 0.69 (0.39,1.13) | 0.69 (0.39,1.15) | 13.67 (7.64,22.59) | 13.71 (7.60,22.66) | 0.02 (0.00,0.04) | 0.021 |
| Mongolia | 398.76 (229.89,640.43) | 820.14 (481.09,1323.55) | 102.98 (59.91,165.64) | 110.96 (64.81,179.84) | 0.27 (0.23,0.31) | 0 |
| Montenegro | 191.65 (110.39,314.30) | 202.14 (117.41,332.02) | 155.96 (89.79,255.74) | 180.41 (104.51,295.64) | 0.51 (0.48,0.54) | 0 |
| Morocco | 6376.95 (3688.84,10225.04) | 10694.27 (6258.97,17260.59) | 132.45 (76.89,212.64) | 144.47 (84.49,233.20) | 0.31 (0.25,0.36) | 0 |
| Mozambique | 2064.94 (1190.04,3368.60) | 4944.63 (2782.43,7999.14) | 88.33 (51.12,143.93) | 93.42 (53.05,151.53) | 0.20 (0.16,0.25) | 0 |
| Myanmar | 1758.79 (955.26,2987.73) | 2681.95 (1477.45,4473.09) | 21.92 (11.98,37.23) | 23.57 (12.98,39.31) | 0.26 (0.23,0.29) | 0 |
| Namibia | 358.22 (206.28,588.81) | 750.04 (431.60,1228.75) | 140.50 (81.29,232.12) | 150.88 (87.10,247.67) | 0.26 (0.20,0.32) | 0 |
| Nauru | 1.91 (1.09,3.11) | 2.23 (1.27,3.68) | 98.75 (56.46,160.93) | 102.79 (58.84,170.06) | 0.13 (0.07,0.19) | 0 |
| Nepal | 2129.51 (1215.51,3448.16) | 4066.02 (2365.91,6368.97) | 62.43 (35.89,101.09) | 61.02 (35.62,95.67) | -0.12 (-0.24,0.01) | 0.065 |
| Netherlands | 400.85 (224.74,666.80) | 359.17 (200.48,600.57) | 13.18 (7.36,21.95) | 13.37 (7.45,22.36) | 0.07 (0.00,0.14) | 0.053 |
| New Zealand | 558.82 (311.94,915.09) | 353.55 (212.12,554.78) | 79.96 (44.60,131.06) | 47.82 (28.61,74.96) | -1.79 (-1.87,-1.70) | 0 |
| Nicaragua | 277.25 (153.45,468.55) | 614.86 (339.32,1023.16) | 42.08 (23.50,71.08) | 45.18 (24.95,75.26) | 0.25 (0.22,0.27) | 0 |
| Niger | 1248.23 (712.92,2017.70) | 3660.48 (2087.30,5964.20) | 95.58 (55.05,155.32) | 101.98 (58.70,166.94) | 0.25 (0.21,0.30) | 0 |
| Nigeria | 16438.46 (9413.16,26525.68) | 45146.93 (25746.84,72910.08) | 106.35 (61.48,172.02) | 111.60 (64.08,180.56) | 0.20 (0.12,0.28) | 0 |
| Niue | 0.37 (0.21,0.60) | 0.29 (0.17,0.48) | 99.50 (56.66,162.24) | 106.22 (60.92,173.20) | 0.21 (0.10,0.32) | 0 |
| North Macedonia | 623.30 (360.84,1022.99) | 748.80 (430.04,1263.05) | 155.18 (89.80,254.60) | 179.98 (102.98,302.65) | 0.52 (0.49,0.56) | 0 |
| Northern Mariana Islands | 19.57 (11.42,30.85) | 7.47 (4.32,12.05) | 169.75 (99.19,267.59) | 142.01 (83.04,227.10) | -0.63 (-0.75,-0.52) | 0 |
| Norway | 551.60 (314.68,906.44) | 650.83 (373.17,1064.37) | 69.35 (39.51,113.92) | 73.01 (41.75,119.43) | 0.21 (0.08,0.35) | 0.002 |
| Oman | 357.70 (205.09,574.47) | 1233.52 (718.26,1986.11) | 132.36 (76.14,212.97) | 146.99 (85.32,237.59) | 0.37 (0.32,0.42) | 0 |
| Pakistan | 13304.52 (7547.19,21968.17) | 34020.21 (19481.12,55972.07) | 77.79 (44.60,128.55) | 82.68 (47.61,136.35) | 0.26 (0.14,0.39) | 0 |
| Palau | 3.49 (2.00,5.71) | 3.01 (1.73,4.96) | 107.06 (61.53,175.22) | 112.21 (64.48,184.12) | 0.14 (0.09,0.18) | 0 |
| Palestine | 482.83 (280.52,767.85) | 1498.60 (874.04,2419.78) | 152.94 (89.65,243.49) | 161.28 (94.57,260.92) | 0.18 (0.17,0.19) | 0 |
| Panama | 203.02 (113.24,341.49) | 395.85 (224.30,661.62) | 44.66 (25.01,75.31) | 50.75 (28.77,84.79) | 0.44 (0.38,0.50) | 0 |
| Papua New Guinea | 830.27 (474.57,1337.74) | 2510.39 (1473.98,3971.58) | 113.02 (64.81,182.09) | 129.69 (76.24,205.24) | 0.46 (0.36,0.56) | 0 |
| Paraguay | 258.56 (144.56,431.00) | 542.11 (301.82,906.57) | 35.95 (20.22,60.01) | 37.95 (21.15,63.62) | 0.19 (0.17,0.20) | 0 |
| Peru | 3037.54 (1761.18,4898.13) | 6190.91 (3653.87,10016.49) | 75.10 (43.92,121.18) | 89.74 (52.91,145.24) | 0.57 (0.49,0.66) | 0 |
| Philippines | 3214.38 (1751.50,5368.54) | 6075.32 (3332.71,10207.01) | 26.68 (14.65,44.66) | 27.81 (15.31,46.77) | 0.15 (0.08,0.22) | 0 |
| Poland | 14842.83 (8776.93,23960.10) | 12331.46 (7875.78,18144.01) | 188.25 (110.83,303.10) | 164.92 (104.95,242.51) | -0.41 (-0.63,-0.19) | 0 |
| Portugal | 224.39 (122.99,377.25) | 199.36 (111.34,334.95) | 11.84 (6.49,19.89) | 12.01 (6.66,20.13) | 0.05 (0.03,0.08) | 0 |
| Puerto Rico | 402.67 (229.73,676.86) | 400.91 (233.25,653.28) | 55.65 (31.77,93.53) | 66.62 (38.67,108.40) | 0.58 (0.49,0.66) | 0 |
| Qatar | 95.17 (54.91,157.17) | 627.97 (367.70,999.69) | 134.52 (77.34,222.72) | 147.84 (85.84,236.22) | 0.34 (0.28,0.39) | 0 |
| Republic of Korea | 2981.15 (1663.44,4849.26) | 2463.33 (1353.34,4097.54) | 29.25 (16.35,47.64) | 28.56 (15.59,47.41) | -0.08 (-0.53,0.36) | 0.717 |
| Republic of Moldova | 1789.66 (1042.01,2911.85) | 1508.50 (884.31,2465.87) | 185.99 (108.04,302.57) | 199.07 (116.04,325.18) | 0.25 (0.19,0.30) | 0 |
| Romania | 7068.45 (4182.34,11464.71) | 5595.35 (3264.21,9255.05) | 160.32 (94.57,259.33) | 180.91 (105.03,298.12) | 0.42 (0.38,0.46) | 0 |
| Russian Federation | 63365.10 (37035.87,102760.05) | 60969.89 (35841.89,99693.53) | 199.83 (116.41,324.06) | 213.28 (124.52,347.83) | 0.24 (0.19,0.28) | 0 |
| Rwanda | 1139.70 (660.83,1857.60) | 2455.57 (1413.83,3987.05) | 89.47 (52.14,146.28) | 94.77 (54.78,153.91) | 0.21 (0.18,0.24) | 0 |
| Saint Kitts and Nevis | 4.53 (2.59,7.47) | 7.28 (4.16,11.99) | 55.09 (31.61,91.14) | 61.09 (34.80,100.71) | 0.34 (0.30,0.38) | 0 |
| Saint Lucia | 13.62 (7.81,22.55) | 22.25 (13.07,36.00) | 53.76 (31.06,89.19) | 63.67 (37.30,103.01) | 0.58 (0.52,0.64) | 0 |
| Saint Vincent and the Grenadines | 9.96 (5.57,16.52) | 11.86 (6.80,19.56) | 50.22 (28.31,83.57) | 56.46 (32.32,92.94) | 0.41 (0.39,0.42) | 0 |
| Samoa | 30.28 (17.33,48.69) | 42.20 (24.52,68.90) | 109.51 (63.47,175.80) | 112.67 (65.91,183.64) | 0.09 (0.06,0.12) | 0 |
| San Marino | 0.57 (0.31,0.95) | 0.73 (0.40,1.22) | 13.15 (7.23,21.86) | 12.98 (7.09,21.78) | -0.03 (-0.15,0.09) | 0.676 |
| Sao Tome and Principe | 18.70 (10.82,30.00) | 42.14 (24.21,68.21) | 96.99 (56.63,155.77) | 103.50 (59.77,167.53) | 0.23 (0.19,0.27) | 0 |
| Saudi Arabia | 3655.28 (2153.15,5829.01) | 15865.10 (9413.77,24659.85) | 147.92 (87.73,236.23) | 189.42 (111.91,295.20) | 0.85 (0.82,0.87) | 0 |
| Senegal | 1206.54 (690.25,1973.09) | 2905.60 (1646.22,4709.14) | 92.11 (53.15,151.49) | 101.96 (58.11,165.15) | 0.38 (0.29,0.47) | 0 |
| Serbia | 2322.17 (1351.04,3771.38) | 2293.30 (1332.52,3722.97) | 129.83 (75.36,210.84) | 152.25 (88.22,246.55) | 0.56 (0.52,0.60) | 0 |
| Seychelles | 3.11 (1.71,5.22) | 4.08 (2.23,6.86) | 22.11 (12.22,37.37) | 22.71 (12.38,38.17) | 0.10 (0.08,0.12) | 0 |
| Sierra Leone | 650.05 (373.30,1054.48) | 1683.13 (959.21,2694.60) | 95.66 (55.24,155.29) | 102.21 (58.51,164.07) | 0.24 (0.20,0.28) | 0 |
| Singapore | 1366.52 (788.04,2233.45) | 1779.77 (1016.86,2934.15) | 181.90 (104.56,297.94) | 157.13 (89.04,259.28) | -0.42 (-0.66,-0.17) | 0.001 |
| Slovakia | 1663.23 (960.33,2736.40) | 1869.54 (1088.47,3025.50) | 154.62 (89.14,253.53) | 182.65 (105.89,295.14) | 0.57 (0.47,0.66) | 0 |
| Slovenia | 705.99 (408.78,1168.20) | 692.87 (408.35,1166.32) | 175.59 (101.44,290.01) | 205.16 (120.22,342.89) | 0.53 (0.46,0.59) | 0 |
| Solomon Islands | 50.27 (28.36,83.57) | 119.94 (68.32,196.33) | 89.85 (51.20,148.89) | 95.80 (54.77,156.67) | 0.23 (0.19,0.26) | 0 |
| Somalia | 1073.67 (613.14,1774.86) | 3128.46 (1761.74,5099.55) | 89.50 (51.21,147.38) | 92.70 (52.79,150.84) | 0.13 (0.11,0.15) | 0 |
| South Africa | 12302.91 (7168.83,19633.30) | 19608.34 (11453.47,31357.85) | 162.27 (95.09,259.45) | 161.21 (94.02,258.46) | 0.04 (-0.01,0.09) | 0.123 |
| South Sudan | 864.60 (493.77,1408.79) | 1522.83 (869.81,2551.94) | 89.71 (51.78,146.73) | 93.07 (53.46,155.69) | 0.13 (0.10,0.16) | 0 |
| Spain | 841.57 (471.87,1385.80) | 981.79 (547.78,1626.88) | 11.67 (6.56,19.21) | 13.65 (7.55,22.61) | 0.56 (0.49,0.63) | 0 |
| Sri Lanka | 819.21 (455.27,1383.13) | 998.60 (546.08,1731.68) | 22.85 (12.74,38.63) | 23.45 (12.80,40.50) | 0.08 (0.03,0.13) | 0.001 |
| Sudan | 4619.74 (2646.07,7470.02) | 11940.50 (6849.30,19265.94) | 130.91 (75.54,211.83) | 145.02 (83.41,234.37) | 0.37 (0.32,0.41) | 0 |
| Suriname | 37.10 (21.50,59.99) | 68.53 (39.09,112.80) | 52.81 (30.87,85.52) | 62.48 (35.61,102.84) | 0.56 (0.51,0.61) | 0 |
| Sweden | 279.33 (154.35,468.50) | 272.00 (151.70,453.02) | 18.86 (10.39,31.63) | 16.70 (9.27,27.89) | -0.42 (-0.52,-0.32) | 0 |
| Switzerland | 174.16 (95.83,291.08) | 190.01 (106.10,313.20) | 12.95 (7.10,21.70) | 12.70 (7.04,21.01) | 0.01 (-0.10,0.12) | 0.912 |
| Syrian Arab Republic | 2713.73 (1553.15,4373.42) | 4083.63 (2351.04,6732.61) | 133.12 (76.93,214.48) | 147.31 (85.21,239.84) | 0.36 (0.32,0.41) | 0 |
| Taiwan (Province of China) | 837.77 (459.15,1402.59) | 1232.33 (718.24,2008.58) | 18.27 (10.00,30.63) | 27.81 (16.04,45.15) | 1.50 (1.41,1.59) | 0 |
| Tajikistan | 1058.94 (621.50,1699.59) | 2264.87 (1312.25,3658.50) | 113.44 (67.10,182.51) | 117.30 (68.14,190.29) | 0.12 (0.09,0.15) | 0 |
| Thailand | 2765.32 (1499.13,4632.44) | 2939.78 (1624.60,4960.89) | 21.99 (11.94,36.94) | 22.54 (12.38,37.91) | 0.09 (0.06,0.11) | 0 |
| Timor-Leste | 31.72 (17.48,53.49) | 53.92 (29.49,90.78) | 21.77 (12.04,36.82) | 22.65 (12.53,38.36) | 0.15 (0.10,0.20) | 0 |
| Togo | 632.87 (361.43,1037.59) | 1640.55 (945.65,2658.25) | 96.43 (55.43,158.66) | 101.89 (58.86,165.31) | 0.22 (0.16,0.27) | 0 |
| Tokelau | 0.26 (0.15,0.42) | 0.23 (0.13,0.37) | 93.53 (53.39,151.89) | 102.75 (58.92,169.69) | 0.31 (0.24,0.38) | 0 |
| Tonga | 15.61 (8.76,25.75) | 19.28 (11.21,31.57) | 95.21 (53.83,156.56) | 102.40 (59.70,167.48) | 0.26 (0.24,0.28) | 0 |
| Trinidad and Tobago | 131.32 (75.36,215.87) | 175.23 (102.08,291.43) | 54.50 (31.33,89.79) | 61.90 (35.82,102.73) | 0.43 (0.39,0.47) | 0 |
| Tunisia | 2077.68 (1191.70,3393.87) | 3604.43 (2097.30,5803.55) | 132.79 (76.54,217.57) | 146.80 (85.06,235.89) | 0.36 (0.32,0.39) | 0 |
| Turkey | 16059.69 (9300.58,25955.21) | 27488.09 (15835.92,45095.35) | 143.34 (83.38,231.68) | 159.54 (91.77,261.19) | 0.36 (0.32,0.40) | 0 |
| Turkmenistan | 732.99 (423.64,1164.48) | 1050.43 (604.52,1685.38) | 102.62 (59.66,163.52) | 109.41 (62.92,175.90) | 0.23 (0.20,0.26) | 0 |
| Tuvalu | 1.78 (1.02,2.90) | 2.13 (1.21,3.51) | 93.20 (53.15,151.79) | 100.76 (57.58,165.87) | 0.27 (0.24,0.29) | 0 |
| Uganda | 2575.79 (1465.33,4170.05) | 6954.12 (4003.18,11307.64) | 89.51 (51.32,145.56) | 94.47 (54.95,154.78) | 0.19 (0.14,0.23) | 0 |
| Ukraine | 21883.69 (12940.89,35310.49) | 17497.93 (9983.50,28904.41) | 213.20 (125.75,344.06) | 203.97 (116.08,336.32) | -0.14 (-0.23,-0.05) | 0.002 |
| United Arab Emirates | 407.72 (235.81,653.69) | 2479.88 (1469.76,4031.40) | 134.45 (77.75,217.16) | 147.96 (86.35,235.77) | 0.34 (0.29,0.39) | 0 |
| United Kingdom | 1412.69 (795.21,2298.32) | 1840.05 (1059.22,2969.77) | 13.25 (7.45,21.59) | 15.80 (9.03,25.56) | 0.62 (0.55,0.69) | 0 |
| United Republic of Tanzania | 4047.28 (2314.92,6521.44) | 9958.48 (5682.61,16172.10) | 89.29 (51.50,144.10) | 93.77 (53.81,152.29) | 0.18 (0.13,0.23) | 0 |
| United States of America | 107155.42 (62636.41,175136.03) | 97417.97 (59440.91,150632.26) | 195.71 (114.11,319.98) | 169.27 (103.17,261.45) | -0.45 (-0.60,-0.30) | 0 |
| United States Virgin Islands | 11.63 (6.66,19.18) | 10.90 (6.29,18.24) | 54.55 (31.19,89.78) | 64.02 (36.81,106.81) | 0.52 (0.40,0.64) | 0 |
| Uruguay | 351.11 (199.90,587.73) | 358.04 (202.53,584.61) | 61.51 (35.02,102.99) | 57.33 (32.38,93.58) | -0.20 (-0.70,0.30) | 0.429 |
| Uzbekistan | 5449.33 (3187.91,8722.46) | 8526.90 (4987.36,13920.72) | 138.44 (81.35,222.75) | 119.58 (69.94,195.58) | -0.50 (-0.57,-0.42) | 0 |
| Vanuatu | 28.14 (16.24,45.58) | 60.48 (34.79,98.97) | 102.14 (59.26,165.62) | 106.17 (61.34,173.52) | 0.11 (0.02,0.21) | 0.02 |
| Venezuela (Bolivarian Republic of) | 1633.62 (916.29,2783.91) | 2588.17 (1458.47,4377.76) | 43.47 (24.49,74.25) | 44.96 (25.29,75.92) | 0.11 (0.07,0.15) | 0 |
| Viet Nam | 2992.84 (1615.79,5038.50) | 4633.58 (2570.19,7690.33) | 21.72 (11.80,36.72) | 22.48 (12.40,37.38) | 0.12 (0.11,0.14) | 0 |
| Yemen | 2858.91 (1643.13,4670.31) | 8785.05 (5134.21,14296.32) | 132.14 (76.17,216.07) | 141.95 (83.12,231.33) | 0.26 (0.23,0.29) | 0 |
| Zambia | 1196.45 (676.42,1942.01) | 3295.09 (1891.37,5338.70) | 89.21 (51.03,145.58) | 93.31 (53.87,151.44) | 0.16 (0.13,0.20) | 0 |
| Zimbabwe | 2605.62 (1482.38,4219.82) | 4612.76 (2647.31,7533.00) | 143.96 (82.44,233.26) | 150.71 (86.89,246.32) | 0.16 (0.06,0.27) | 0.002 |

NOTE:ASIR,age-standardised incidence rate;APPC,average annual percentage change;

| **Table 8.DALYs of interstitial lung disease and pulmonary sarcoidosis in adolescents and young adultsr in 1990 and 2019 for female and all nation, with AAPC from 2009 and 2019** | | | | | | |
| --- | --- | --- | --- | --- | --- | --- |
| location | Numbers in 1990 | Numbers in 2019 | Age-standardized rates in 1990   (95% CI) | Age standardized rates in 2019   (95% CI) | AAPC, %   (95% CI) | P |
| Afghanistan | 78.10 (34.57,153.05) | 332.08 (151.35,664.35) | 4.34 (1.97,8.28) | 5.14 (2.38,10.21) | 0.56 (0.36,0.77) | 0 |
| Albania | 53.84 (32.23,83.13) | 29.76 (16.56,50.82) | 8.18 (4.90,12.59) | 6.40 (3.56,10.92) | -0.85 (-1.07,-0.64) | 0 |
| Algeria | 248.28 (107.11,505.85) | 638.26 (298.68,1248.45) | 5.56 (2.42,11.24) | 7.11 (3.30,13.97) | 0.88 (0.75,1.00) | 0 |
| American Samoa | 2.52 (1.25,4.62) | 2.10 (1.02,3.77) | 26.70 (13.33,48.86) | 20.96 (10.34,37.36) | -0.79 (-1.05,-0.53) | 0 |
| Andorra | 0.72 (0.33,1.34) | 1.06 (0.51,1.87) | 5.91 (2.65,11.06) | 7.67 (3.63,13.64) | 0.92 (0.73,1.11) | 0 |
| Angola | 90.45 (30.09,210.10) | 264.21 (120.57,533.26) | 5.08 (1.68,11.79) | 4.92 (2.25,9.92) | -0.12 (-0.27,0.03) | 0.103 |
| Antigua and Barbuda | 0.49 (0.29,0.87) | 0.93 (0.55,1.43) | 3.82 (2.24,6.70) | 5.07 (3.02,7.84) | 0.92 (0.76,1.09) | 0 |
| Argentina | 910.77 (480.77,1388.83) | 1162.72 (649.78,1797.31) | 14.92 (7.87,22.73) | 13.03 (7.27,20.17) | -0.46 (-0.84,-0.09) | 0.015 |
| Armenia | 69.39 (38.30,130.89) | 56.17 (33.98,112.58) | 9.48 (5.21,17.83) | 8.78 (5.24,17.86) | -0.32 (-0.57,-0.07) | 0.011 |
| Australia | 99.53 (60.88,163.89) | 235.68 (122.84,385.58) | 2.84 (1.74,4.68) | 5.21 (2.71,8.52) | 2.12 (1.86,2.38) | 0 |
| Austria | 60.40 (38.61,91.84) | 73.90 (43.67,112.64) | 4.06 (2.59,6.18) | 5.07 (2.96,7.77) | 0.75 (0.40,1.10) | 0 |
| Azerbaijan | 341.29 (178.25,586.55) | 332.05 (166.55,613.69) | 21.83 (11.39,37.50) | 15.12 (7.54,28.12) | -1.32 (-1.60,-1.03) | 0 |
| Bahamas | 8.04 (4.54,13.00) | 16.05 (7.59,27.17) | 14.11 (7.98,22.75) | 20.54 (9.71,34.78) | 1.20 (0.90,1.50) | 0 |
| Bahrain | 10.35 (4.98,20.35) | 34.18 (17.94,60.15) | 10.93 (5.33,21.29) | 12.83 (6.61,22.96) | 0.47 (-0.03,0.97) | 0.066 |
| Bangladesh | 1341.53 (640.34,2600.59) | 2672.13 (1220.11,5988.44) | 7.34 (3.51,14.13) | 7.85 (3.58,17.57) | 0.16 (-0.26,0.59) | 0.453 |
| Barbados | 3.95 (2.27,6.71) | 5.80 (3.28,9.54) | 7.06 (4.05,11.96) | 11.02 (6.19,18.17) | 1.45 (0.97,1.93) | 0 |
| Belarus | 174.94 (94.18,284.35) | 96.61 (42.04,209.86) | 8.40 (4.53,13.66) | 5.52 (2.47,12.23) | -1.43 (-1.55,-1.30) | 0 |
| Belgium | 69.86 (42.64,111.03) | 92.48 (53.27,146.74) | 3.65 (2.21,5.86) | 4.98 (2.86,7.97) | 1.12 (0.78,1.46) | 0 |
| Belize | 4.49 (2.28,8.12) | 26.00 (13.86,41.59) | 14.10 (7.16,25.59) | 30.02 (16.07,47.91) | 2.65 (2.25,3.06) | 0 |
| Benin | 30.37 (15.05,56.31) | 82.79 (42.76,156.05) | 3.52 (1.74,6.50) | 3.58 (1.86,6.73) | 0.06 (-0.05,0.18) | 0.285 |
| Bermuda | 1.75 (0.98,3.04) | 1.49 (0.91,2.38) | 12.40 (6.89,21.53) | 14.52 (8.82,23.38) | 0.57 (0.47,0.67) | 0 |
| Bhutan | 7.75 (3.20,16.79) | 16.49 (7.08,31.74) | 7.71 (3.19,16.35) | 10.13 (4.36,19.49) | 0.95 (0.83,1.08) | 0 |
| Bolivia (Plurinational State of) | 213.43 (87.96,434.43) | 456.84 (195.51,832.46) | 17.84 (7.45,36.21) | 19.36 (8.29,35.21) | 0.30 (0.19,0.41) | 0 |
| Bosnia and Herzegovina | 55.90 (31.02,96.34) | 27.90 (14.83,49.49) | 5.95 (3.29,10.26) | 5.07 (2.69,9.00) | -0.52 (-0.72,-0.32) | 0 |
| Botswana | 26.05 (9.69,66.69) | 48.90 (23.01,95.65) | 10.82 (4.02,27.85) | 8.92 (4.20,17.44) | -0.65 (-0.78,-0.53) | 0 |
| Brazil | 2741.19 (1863.85,3813.10) | 4120.33 (2651.90,5825.57) | 8.96 (6.10,12.45) | 9.20 (5.89,12.99) | 0.07 (-0.32,0.47) | 0.711 |
| Brunei Darussalam | 8.95 (4.26,16.13) | 10.52 (5.59,18.62) | 15.80 (7.53,28.44) | 11.03 (5.82,19.65) | -1.23 (-1.35,-1.10) | 0 |
| Bulgaria | 80.13 (48.47,124.46) | 59.66 (35.68,94.39) | 5.14 (3.10,8.01) | 5.40 (3.23,8.57) | 0.21 (0.09,0.34) | 0.001 |
| Burkina Faso | 44.89 (23.83,79.05) | 126.75 (67.18,226.14) | 2.83 (1.51,4.98) | 3.04 (1.62,5.39) | 0.27 (0.03,0.52) | 0.028 |
| Burundi | 57.61 (16.80,151.74) | 119.23 (47.37,300.11) | 5.85 (1.70,15.32) | 5.52 (2.19,13.91) | -0.21 (-0.44,0.03) | 0.081 |
| Cabo Verde | 2.97 (1.47,5.50) | 3.91 (2.20,6.31) | 4.70 (2.35,8.60) | 3.26 (1.83,5.27) | -1.25 (-1.42,-1.08) | 0 |
| Cambodia | 20.73 (9.81,40.49) | 51.20 (22.43,99.84) | 1.10 (0.52,2.17) | 1.47 (0.64,2.87) | 1.01 (0.95,1.07) | 0 |
| Cameroon | 79.95 (40.83,151.69) | 236.48 (125.60,418.19) | 4.44 (2.27,8.39) | 4.12 (2.20,7.26) | -0.26 (-0.41,-0.11) | 0.001 |
| Canada | 523.66 (297.92,880.67) | 659.34 (397.94,1031.67) | 8.72 (4.99,14.60) | 10.73 (6.48,16.73) | 0.74 (0.38,1.10) | 0 |
| Central African Republic | 23.56 (7.75,59.21) | 39.81 (17.27,83.17) | 4.88 (1.59,12.39) | 3.98 (1.72,8.31) | -0.75 (-1.25,-0.25) | 0.003 |
| Chad | 38.27 (18.60,75.37) | 91.65 (49.15,166.19) | 3.78 (1.84,7.42) | 3.42 (1.84,6.16) | -0.29 (-0.40,-0.18) | 0 |
| Chile | 246.46 (125.84,518.62) | 369.36 (228.23,627.02) | 8.74 (4.49,18.31) | 10.65 (6.56,18.04) | 0.73 (0.59,0.87) | 0 |
| China | 6949.69 (4561.32,12143.90) | 5568.53 (3654.83,8315.57) | 2.72 (1.79,4.76) | 2.10 (1.38,3.14) | -0.93 (-1.12,-0.73) | 0 |
| Colombia | 208.24 (121.81,382.32) | 1103.40 (353.94,2211.71) | 3.03 (1.78,5.54) | 11.38 (3.65,22.80) | 4.85 (4.20,5.52) | 0 |
| Comoros | 3.67 (1.04,9.47) | 6.91 (2.89,14.77) | 4.70 (1.35,11.98) | 4.96 (2.08,10.54) | -0.24 (-2.27,1.84) | 0.822 |
| Congo | 19.59 (6.54,46.53) | 49.53 (21.45,98.98) | 4.58 (1.52,10.78) | 4.66 (2.02,9.30) | 0.06 (-0.07,0.18) | 0.377 |
| Cook Islands | 1.58 (0.74,2.88) | 0.85 (0.29,1.56) | 45.28 (21.36,81.93) | 27.16 (9.11,49.79) | -1.78 (-1.96,-1.61) | 0 |
| Costa Rica | 84.64 (47.96,140.78) | 171.02 (94.11,296.75) | 13.35 (7.56,22.27) | 16.97 (9.36,29.40) | 0.85 (0.06,1.65) | 0.036 |
| Coted'Ivoire | 70.50 (35.59,128.59) | 157.52 (84.78,274.54) | 3.34 (1.69,6.05) | 3.08 (1.66,5.36) | -0.27 (-0.35,-0.18) | 0 |
| Croatia | 49.25 (29.45,77.95) | 39.69 (20.40,65.14) | 5.16 (3.07,8.17) | 5.72 (3.00,9.32) | 0.35 (0.07,0.62) | 0.014 |
| Cuba | 106.45 (52.06,179.05) | 94.66 (49.67,157.77) | 4.72 (2.33,7.85) | 5.19 (2.71,8.69) | 0.35 (0.00,0.69) | 0.048 |
| Cyprus | 8.28 (3.07,18.88) | 12.11 (5.10,25.29) | 5.49 (2.03,12.51) | 4.50 (1.86,9.43) | -0.70 (-1.17,-0.24) | 0.003 |
| Czechia | 112.64 (70.54,172.79) | 136.85 (72.23,219.59) | 5.78 (3.60,8.90) | 8.05 (4.20,13.00) | 1.16 (0.94,1.38) | 0 |
| Democratic People's Republic of Korea | 119.31 (49.48,256.16) | 123.79 (55.15,252.85) | 2.92 (1.22,6.23) | 2.55 (1.13,5.22) | -0.48 (-0.58,-0.38) | 0 |
| Democratic Republic of the Congo | 373.08 (118.56,916.02) | 875.56 (354.09,1985.48) | 5.74 (1.81,14.05) | 5.59 (2.26,12.64) | -0.11 (-0.44,0.21) | 0.491 |
| Denmark | 56.10 (32.12,98.00) | 92.27 (52.53,144.30) | 6.01 (3.41,10.49) | 10.49 (5.94,16.42) | 1.92 (1.59,2.24) | 0 |
| Djibouti | 3.81 (1.33,9.24) | 10.91 (4.61,23.16) | 4.80 (1.66,11.62) | 4.28 (1.80,9.11) | -0.41 (-0.64,-0.18) | 0 |
| Dominica | 0.76 (0.40,1.36) | 1.17 (0.54,2.18) | 5.97 (3.17,10.55) | 9.28 (4.29,17.28) | 1.56 (1.23,1.89) | 0 |
| Dominican Republic | 77.74 (40.26,137.58) | 192.98 (73.51,418.81) | 5.47 (2.86,9.59) | 8.89 (3.38,19.33) | 1.69 (1.30,2.09) | 0 |
| Ecuador | 245.86 (131.08,427.01) | 546.53 (291.22,906.97) | 12.35 (6.63,21.35) | 15.45 (8.24,25.60) | 0.64 (0.31,0.96) | 0 |
| Egypt | 633.46 (284.67,1308.05) | 1685.66 (746.64,3475.85) | 6.28 (2.86,12.86) | 8.55 (3.80,17.62) | 1.11 (0.89,1.32) | 0 |
| El Salvador | 63.57 (29.69,127.07) | 109.04 (55.78,191.05) | 6.35 (3.03,12.56) | 8.25 (4.23,14.46) | 0.89 (0.57,1.22) | 0 |
| Equatorial Guinea | 3.44 (1.19,8.02) | 14.00 (5.60,32.88) | 4.53 (1.56,10.50) | 5.38 (2.15,12.67) | 0.61 (0.13,1.08) | 0.012 |
| Eritrea | 20.99 (7.64,49.86) | 56.77 (25.65,116.42) | 4.06 (1.47,9.63) | 4.46 (2.01,9.15) | 0.34 (0.20,0.48) | 0 |
| Estonia | 33.25 (17.45,53.68) | 21.11 (11.29,34.31) | 11.40 (5.85,18.59) | 10.00 (5.18,16.48) | -0.31 (-0.80,0.18) | 0.217 |
| Eswatini | 13.23 (5.17,33.43) | 18.05 (8.60,35.64) | 9.22 (3.61,23.18) | 7.27 (3.47,14.33) | -0.83 (-1.03,-0.63) | 0 |
| Ethiopia | 311.05 (128.78,604.07) | 888.36 (440.40,1561.86) | 3.51 (1.44,6.83) | 4.39 (2.17,7.71) | 0.79 (0.68,0.89) | 0 |
| Fiji | 22.44 (11.44,41.34) | 24.86 (13.06,43.71) | 14.46 (7.38,26.58) | 14.07 (7.38,24.74) | -0.08 (-0.25,0.09) | 0.362 |
| Finland | 34.08 (18.29,62.41) | 38.59 (22.92,63.38) | 3.55 (1.86,6.65) | 4.48 (2.61,7.49) | 0.79 (0.58,0.99) | 0 |
| France | 492.87 (266.80,733.39) | 468.92 (282.88,719.18) | 4.37 (2.35,6.52) | 4.55 (2.72,7.00) | 0.18 (0.02,0.35) | 0.024 |
| Gabon | 8.43 (2.88,18.44) | 17.54 (8.02,34.72) | 5.07 (1.73,11.06) | 4.73 (2.16,9.35) | -0.26 (-0.72,0.21) | 0.284 |
| Gambia | 6.06 (3.01,11.77) | 16.14 (8.62,30.73) | 3.41 (1.70,6.58) | 3.60 (1.93,6.84) | 0.24 (-0.28,0.77) | 0.363 |
| Georgia | 89.46 (52.62,135.73) | 53.99 (29.69,86.42) | 8.00 (4.70,12.15) | 8.37 (4.58,13.44) | 0.28 (-0.33,0.89) | 0.374 |
| Germany | 837.39 (439.35,1297.23) | 638.22 (391.81,985.05) | 5.52 (2.91,8.61) | 4.91 (3.00,7.71) | -0.44 (-0.81,-0.07) | 0.021 |
| Ghana | 100.53 (56.29,164.60) | 281.68 (152.12,480.13) | 3.67 (2.06,5.99) | 4.12 (2.23,7.00) | 0.38 (0.14,0.62) | 0.002 |
| Greece | 38.02 (21.71,70.94) | 53.27 (31.79,82.07) | 2.01 (1.14,3.75) | 3.44 (2.03,5.33) | 1.85 (1.44,2.27) | 0 |
| Greenland | 1.30 (0.47,3.21) | 1.55 (0.61,3.56) | 10.91 (3.89,27.12) | 15.02 (5.88,34.65) | 1.21 (0.54,1.87) | 0 |
| Grenada | 1.44 (0.80,2.44) | 2.94 (1.49,4.87) | 9.42 (5.29,15.90) | 15.69 (7.96,25.93) | 1.81 (1.59,2.03) | 0 |
| Guam | 19.87 (11.33,32.03) | 18.21 (10.46,29.13) | 70.24 (40.06,113.00) | 65.04 (37.42,104.01) | -0.37 (-0.66,-0.08) | 0.012 |
| Guatemala | 172.11 (81.66,311.26) | 456.74 (245.42,798.86) | 12.06 (5.84,21.75) | 11.62 (6.26,20.27) | -0.17 (-1.27,0.95) | 0.769 |
| Guinea | 37.15 (18.78,68.32) | 82.02 (43.87,147.72) | 3.39 (1.72,6.21) | 3.38 (1.81,6.07) | 0.01 (-0.08,0.09) | 0.886 |
| Guinea-Bissau | 6.29 (3.14,11.82) | 13.68 (7.40,24.66) | 3.45 (1.72,6.47) | 3.47 (1.88,6.25) | 0.04 (-0.06,0.14) | 0.452 |
| Guyana | 11.37 (6.40,18.73) | 26.32 (12.37,47.31) | 7.26 (4.12,11.90) | 17.12 (8.09,30.68) | 3.07 (2.69,3.45) | 0 |
| Haiti | 89.21 (37.44,208.12) | 351.10 (145.69,780.03) | 7.50 (3.15,17.54) | 12.76 (5.29,28.31) | 1.89 (1.44,2.34) | 0 |
| Honduras | 142.89 (58.66,286.20) | 292.26 (105.67,634.77) | 17.59 (7.24,35.04) | 14.12 (5.11,30.60) | -0.78 (-1.40,-0.16) | 0.013 |
| Hungary | 160.34 (94.69,238.63) | 126.24 (77.59,192.80) | 7.96 (4.73,11.91) | 8.04 (4.92,12.35) | 0.06 (-0.18,0.30) | 0.605 |
| Iceland | 2.19 (1.31,3.68) | 4.54 (2.41,7.27) | 4.27 (2.55,7.18) | 7.62 (4.01,12.26) | 2.06 (1.64,2.48) | 0 |
| India | 15051.83 (8726.28,23004.56) | 34424.14 (21669.17,50579.03) | 9.87 (5.73,15.01) | 12.17 (7.66,17.86) | 0.80 (0.38,1.21) | 0 |
| Indonesia | 832.63 (488.07,1496.34) | 1518.41 (879.66,2988.08) | 2.30 (1.34,4.17) | 2.83 (1.65,5.56) | 0.71 (0.62,0.79) | 0 |
| Iran (Islamic Republic of) | 372.03 (223.83,589.30) | 962.54 (533.39,1437.61) | 3.70 (2.24,5.83) | 4.98 (2.73,7.42) | 1.06 (0.80,1.32) | 0 |
| Iraq | 177.80 (88.46,347.69) | 580.11 (273.45,1087.32) | 5.94 (3.02,11.30) | 6.74 (3.25,12.45) | 0.51 (0.34,0.68) | 0 |
| Ireland | 36.96 (20.69,66.95) | 78.88 (43.57,123.84) | 5.47 (3.06,9.94) | 9.04 (4.93,14.31) | 1.70 (1.46,1.94) | 0 |
| Israel | 42.67 (22.09,79.65) | 74.51 (41.63,126.81) | 4.46 (2.31,8.33) | 4.63 (2.58,7.86) | 0.07 (-0.15,0.30) | 0.517 |
| Italy | 376.25 (234.62,598.01) | 467.88 (250.24,661.86) | 3.55 (2.21,5.64) | 5.57 (2.92,7.90) | 1.61 (1.27,1.96) | 0 |
| Jamaica | 26.27 (13.22,47.19) | 50.73 (24.33,90.90) | 5.72 (2.90,10.19) | 8.38 (4.04,14.98) | 1.37 (0.52,2.24) | 0.002 |
| Japan | 1637.45 (1111.63,2499.82) | 1154.05 (801.24,1979.61) | 7.24 (4.92,11.06) | 6.71 (4.67,11.62) | -0.28 (-0.46,-0.09) | 0.004 |
| Jordan | 81.95 (41.92,145.36) | 272.64 (159.74,437.46) | 12.71 (6.59,22.15) | 12.34 (7.24,19.73) | -0.09 (-0.39,0.21) | 0.545 |
| Kazakhstan | 392.42 (196.59,652.34) | 387.33 (182.21,711.14) | 11.58 (5.80,19.25) | 10.26 (4.87,18.77) | -0.41 (-0.84,0.02) | 0.063 |
| Kenya | 213.64 (74.99,458.15) | 606.67 (263.08,1195.18) | 5.68 (1.96,12.38) | 6.02 (2.60,11.90) | 0.21 (0.05,0.37) | 0.012 |
| Kiribati | 1.46 (0.71,2.70) | 2.66 (1.25,4.99) | 9.70 (4.77,17.83) | 10.85 (5.12,20.28) | 0.37 (0.23,0.52) | 0 |
| Kuwait | 20.80 (12.81,31.75) | 69.93 (42.63,114.28) | 5.71 (3.52,8.71) | 5.76 (3.51,9.44) | -0.06 (-0.37,0.26) | 0.733 |
| Kyrgyzstan | 49.90 (24.79,86.94) | 55.43 (29.49,110.90) | 5.68 (2.88,9.78) | 4.25 (2.26,8.48) | -1.06 (-1.45,-0.67) | 0 |
| Lao People's Democratic Republic | 14.26 (6.04,30.64) | 45.41 (18.05,95.73) | 2.02 (0.85,4.38) | 3.06 (1.21,6.47) | 1.48 (1.41,1.55) | 0 |
| Latvia | 55.17 (27.41,88.67) | 22.41 (11.16,41.41) | 11.06 (5.50,17.88) | 7.24 (3.63,13.53) | -1.38 (-1.83,-0.93) | 0 |
| Lebanon | 33.66 (14.93,68.25) | 72.55 (35.65,137.90) | 5.82 (2.60,11.74) | 6.68 (3.24,12.87) | 0.49 (0.41,0.56) | 0 |
| Lesotho | 33.36 (12.67,91.63) | 33.61 (15.74,66.13) | 10.65 (4.04,29.25) | 7.58 (3.55,14.91) | -1.14 (-1.45,-0.84) | 0 |
| Liberia | 9.00 (4.90,15.44) | 28.91 (15.40,53.35) | 2.63 (1.44,4.51) | 3.03 (1.62,5.57) | 0.50 (0.36,0.63) | 0 |
| Libya | 36.93 (16.12,76.38) | 102.39 (45.73,210.74) | 5.60 (2.48,11.45) | 6.74 (3.01,13.90) | 0.65 (0.50,0.80) | 0 |
| Lithuania | 45.04 (25.00,71.27) | 24.32 (14.30,39.64) | 6.41 (3.53,10.17) | 5.54 (3.25,9.05) | -0.59 (-1.08,-0.09) | 0.02 |
| Luxembourg | 2.30 (1.41,3.65) | 6.31 (3.37,10.23) | 2.98 (1.82,4.74) | 5.61 (2.95,9.15) | 2.20 (2.08,2.33) | 0 |
| Madagascar | 220.34 (57.55,585.21) | 475.00 (162.51,1205.88) | 10.59 (2.80,27.70) | 9.35 (3.22,23.51) | -0.45 (-0.69,-0.20) | 0 |
| Malawi | 64.74 (25.04,138.21) | 151.65 (69.91,302.85) | 3.96 (1.52,8.42) | 4.27 (1.97,8.48) | 0.27 (0.10,0.43) | 0.002 |
| Malaysia | 136.70 (63.87,248.51) | 322.90 (158.58,590.81) | 3.92 (1.83,7.10) | 4.96 (2.44,9.07) | 0.85 (0.61,1.09) | 0 |
| Maldives | 8.12 (3.18,19.35) | 23.23 (12.13,39.49) | 24.15 (9.48,57.56) | 24.09 (12.47,41.31) | 0.01 (-0.12,0.14) | 0.911 |
| Mali | 67.49 (26.87,141.37) | 174.31 (71.68,413.60) | 4.62 (1.84,9.66) | 4.61 (1.90,10.84) | -0.01 (-0.25,0.23) | 0.936 |
| Malta | 3.84 (2.26,6.36) | 6.88 (3.87,10.38) | 5.44 (3.18,9.03) | 9.86 (5.45,15.01) | 2.15 (1.89,2.40) | 0 |
| Marshall Islands | 3.56 (1.43,7.36) | 5.09 (1.92,10.41) | 45.05 (18.42,92.24) | 44.09 (16.61,90.17) | -0.07 (-0.12,-0.02) | 0.006 |
| Mauritania | 11.43 (5.94,20.18) | 25.05 (13.73,42.93) | 3.13 (1.63,5.51) | 3.27 (1.80,5.58) | 0.16 (0.05,0.26) | 0.003 |
| Mauritius | 13.12 (7.18,24.12) | 26.99 (13.05,46.27) | 5.49 (3.00,10.10) | 10.80 (5.21,18.53) | 2.35 (1.48,3.23) | 0 |
| Mexico | 2222.72 (1532.62,3244.25) | 3731.73 (2380.85,5463.43) | 13.38 (9.14,19.19) | 14.56 (9.29,21.32) | 0.35 (0.15,0.55) | 0 |
| Micronesia (Federated States of) | 6.83 (2.92,14.06) | 8.17 (2.37,17.48) | 36.32 (15.79,74.08) | 41.31 (11.42,88.93) | 0.45 (0.40,0.50) | 0 |
| Monaco | 0.51 (0.27,0.84) | 0.55 (0.28,0.98) | 10.50 (5.58,17.74) | 11.33 (5.65,20.23) | 0.26 (0.14,0.39) | 0 |
| Mongolia | 51.66 (23.39,105.20) | 65.11 (31.32,133.68) | 13.25 (6.15,26.40) | 8.92 (4.24,18.38) | -1.37 (-1.58,-1.15) | 0 |
| Montenegro | 4.94 (2.90,8.12) | 4.67 (2.41,8.33) | 4.03 (2.36,6.62) | 4.22 (2.21,7.43) | 0.27 (0.02,0.51) | 0.031 |
| Morocco | 208.66 (92.97,405.81) | 395.69 (186.40,780.52) | 4.29 (1.93,8.30) | 5.36 (2.52,10.58) | 0.78 (0.61,0.94) | 0 |
| Mozambique | 71.37 (31.53,144.25) | 193.04 (90.91,378.46) | 3.06 (1.35,6.16) | 3.61 (1.71,6.99) | 0.58 (0.42,0.74) | 0 |
| Myanmar | 247.16 (95.46,558.18) | 405.91 (160.88,879.86) | 3.16 (1.22,7.15) | 3.56 (1.41,7.72) | 0.36 (0.07,0.66) | 0.015 |
| Namibia | 21.67 (8.27,55.38) | 40.44 (18.88,84.10) | 8.68 (3.29,22.31) | 8.13 (3.80,16.94) | -0.22 (-0.37,-0.07) | 0.004 |
| Nauru | 0.76 (0.32,1.56) | 0.85 (0.31,1.73) | 38.45 (16.22,79.38) | 38.81 (14.20,78.79) | 0.03 (-0.08,0.15) | 0.599 |
| Nepal | 306.24 (125.81,612.55) | 938.42 (386.88,1988.05) | 9.33 (3.82,18.62) | 14.41 (5.94,30.36) | 1.52 (1.32,1.72) | 0 |
| Netherlands | 88.56 (52.08,162.27) | 138.91 (67.10,227.93) | 2.92 (1.71,5.36) | 5.20 (2.51,8.53) | 2.04 (1.69,2.40) | 0 |
| New Zealand | 25.47 (16.63,38.74) | 39.90 (22.32,60.44) | 3.65 (2.38,5.55) | 5.36 (3.00,8.13) | 1.28 (0.62,1.94) | 0 |
| Nicaragua | 22.91 (12.35,40.28) | 79.99 (38.07,141.58) | 3.32 (1.82,5.74) | 5.82 (2.77,10.31) | 1.96 (1.33,2.61) | 0 |
| Niger | 53.77 (26.00,104.62) | 151.94 (73.50,312.49) | 4.17 (2.01,8.05) | 4.25 (2.07,8.65) | 0.17 (-0.03,0.38) | 0.099 |
| Nigeria | 496.04 (276.82,777.41) | 1324.56 (776.02,2094.15) | 3.27 (1.82,5.09) | 3.29 (1.94,5.18) | 0.05 (-0.10,0.19) | 0.518 |
| Niue | 0.18 (0.08,0.34) | 0.10 (0.04,0.20) | 47.98 (21.50,91.84) | 37.16 (14.07,74.12) | -0.89 (-1.00,-0.78) | 0 |
| North Macedonia | 18.43 (10.50,30.05) | 18.95 (9.36,33.45) | 4.59 (2.61,7.48) | 4.58 (2.28,8.06) | 0.01 (-0.10,0.12) | 0.835 |
| Northern Mariana Islands | 9.91 (5.06,17.28) | 2.99 (1.52,5.19) | 85.87 (43.84,149.53) | 54.27 (28.30,93.34) | -1.58 (-1.75,-1.41) | 0 |
| Norway | 46.45 (29.88,75.36) | 73.69 (47.42,101.34) | 5.85 (3.76,9.49) | 8.40 (5.35,11.51) | 1.26 (0.95,1.58) | 0 |
| Oman | 24.49 (8.64,56.83) | 68.83 (26.67,152.48) | 8.94 (3.18,20.81) | 8.56 (3.30,18.89) | -0.09 (-0.37,0.19) | 0.537 |
| Pakistan | 906.14 (483.77,1761.76) | 2740.79 (1471.45,4772.57) | 5.45 (2.93,10.49) | 6.78 (3.63,11.80) | 0.75 (0.56,0.95) | 0 |
| Palau | 1.20 (0.57,2.34) | 0.85 (0.40,1.62) | 37.08 (17.64,71.97) | 31.34 (14.67,59.78) | -0.59 (-0.64,-0.53) | 0 |
| Palestine | 76.87 (34.43,155.45) | 184.50 (100.48,299.99) | 23.04 (10.42,46.16) | 19.53 (10.67,31.66) | -0.51 (-0.63,-0.39) | 0 |
| Panama | 40.05 (22.67,66.52) | 109.51 (53.02,189.62) | 8.41 (4.81,13.88) | 13.94 (6.75,24.12) | 1.75 (0.95,2.56) | 0 |
| Papua New Guinea | 251.81 (103.35,558.60) | 715.31 (318.45,1509.89) | 33.81 (13.99,74.04) | 36.80 (16.47,77.41) | 0.27 (0.13,0.42) | 0 |
| Paraguay | 48.96 (24.81,88.88) | 115.94 (54.01,211.33) | 6.62 (3.38,11.93) | 8.07 (3.76,14.69) | 0.63 (0.43,0.83) | 0 |
| Peru | 1101.52 (548.68,1931.51) | 1650.26 (880.85,2740.64) | 25.69 (12.93,44.76) | 24.15 (12.87,40.15) | -0.17 (-0.45,0.11) | 0.242 |
| Philippines | 171.40 (102.39,244.17) | 357.40 (195.64,526.62) | 1.45 (0.88,2.06) | 1.64 (0.90,2.42) | 0.38 (0.13,0.63) | 0.003 |
| Poland | 603.45 (365.55,840.37) | 489.56 (332.30,699.49) | 7.78 (4.68,10.80) | 6.90 (4.63,9.83) | -0.41 (-0.51,-0.31) | 0 |
| Portugal | 98.36 (57.62,159.42) | 132.05 (61.70,217.13) | 5.18 (3.03,8.40) | 8.18 (3.76,13.56) | 1.60 (1.22,1.99) | 0 |
| Puerto Rico | 79.96 (44.32,145.63) | 105.78 (56.23,182.39) | 11.07 (6.14,20.14) | 17.68 (9.36,30.65) | 1.69 (1.50,1.87) | 0 |
| Qatar | 3.61 (1.89,6.39) | 20.65 (10.73,38.10) | 5.24 (2.73,9.32) | 5.08 (2.62,9.33) | -0.09 (-0.32,0.14) | 0.445 |
| Republic of Korea | 604.52 (348.19,1006.05) | 459.82 (278.02,786.20) | 5.93 (3.42,9.87) | 5.30 (3.19,9.13) | -0.39 (-0.58,-0.20) | 0 |
| Republic of Moldova | 53.21 (28.45,91.21) | 44.32 (22.83,82.25) | 5.59 (3.01,9.57) | 5.99 (3.13,11.21) | 0.20 (-0.17,0.56) | 0.296 |
| Romania | 804.28 (451.00,1202.68) | 264.74 (150.04,519.84) | 18.46 (10.30,27.70) | 8.90 (5.05,17.57) | -2.45 (-2.63,-2.26) | 0 |
| Russian Federation | 1667.03 (1070.62,2557.04) | 1535.30 (963.54,2542.82) | 5.32 (3.42,8.13) | 5.54 (3.49,9.24) | 0.17 (0.06,0.28) | 0.003 |
| Rwanda | 76.76 (23.14,192.94) | 181.36 (70.76,464.99) | 6.04 (1.82,15.07) | 7.00 (2.74,17.92) | 0.51 (0.33,0.69) | 0 |
| Saint Kitts and Nevis | 1.14 (0.58,2.03) | 1.38 (0.33,3.13) | 13.78 (7.01,24.49) | 11.74 (2.89,26.38) | -0.68 (-0.86,-0.50) | 0 |
| Saint Lucia | 2.62 (1.52,4.34) | 6.19 (3.48,9.72) | 10.25 (5.96,16.80) | 17.86 (10.05,28.13) | 1.93 (1.12,2.76) | 0 |
| Saint Vincent and the Grenadines | 0.61 (0.28,1.29) | 1.66 (0.84,2.77) | 3.02 (1.37,6.37) | 7.95 (4.02,13.28) | 3.49 (2.73,4.27) | 0 |
| Samoa | 15.37 (6.46,33.34) | 17.35 (6.39,34.28) | 55.41 (23.40,119.55) | 45.56 (16.67,89.80) | -0.67 (-0.75,-0.59) | 0 |
| San Marino | 0.09 (0.05,0.17) | 0.15 (0.06,0.30) | 2.16 (1.08,3.85) | 2.69 (1.14,5.41) | 0.76 (0.66,0.86) | 0 |
| Sao Tome and Principe | 1.04 (0.46,2.06) | 2.70 (1.36,4.98) | 5.54 (2.47,10.81) | 6.63 (3.36,12.18) | 0.56 (0.27,0.86) | 0.001 |
| Saudi Arabia | 555.85 (251.96,1109.66) | 1635.56 (783.18,2897.55) | 22.27 (10.23,44.00) | 20.14 (9.63,35.84) | -0.34 (-0.68,0.02) | 0.061 |
| Senegal | 46.89 (23.77,88.35) | 105.69 (56.04,192.00) | 3.60 (1.83,6.75) | 3.68 (1.96,6.64) | 0.12 (-0.24,0.48) | 0.531 |
| Serbia | 93.33 (53.01,153.32) | 76.96 (46.20,123.79) | 5.20 (2.95,8.55) | 5.11 (3.05,8.25) | -0.08 (-0.32,0.16) | 0.517 |
| Seychelles | 0.34 (0.15,0.68) | 0.46 (0.20,0.93) | 2.55 (1.13,5.13) | 2.51 (1.10,5.12) | -0.06 (-0.25,0.13) | 0.517 |
| Sierra Leone | 20.78 (10.79,37.97) | 53.70 (28.83,97.04) | 3.07 (1.59,5.60) | 3.25 (1.75,5.82) | 0.21 (0.06,0.35) | 0.005 |
| Singapore | 55.45 (27.70,95.74) | 79.76 (38.46,142.11) | 7.47 (3.72,12.91) | 6.97 (3.46,12.07) | -0.15 (-0.36,0.07) | 0.177 |
| Slovakia | 79.54 (47.65,122.81) | 91.79 (44.91,157.02) | 7.44 (4.45,11.50) | 9.27 (4.52,15.95) | 0.75 (0.53,0.97) | 0 |
| Slovenia | 19.46 (10.37,33.35) | 18.70 (8.95,32.84) | 4.89 (2.61,8.38) | 5.78 (2.84,10.06) | 0.59 (0.35,0.83) | 0 |
| Solomon Islands | 10.11 (3.55,33.79) | 27.50 (9.99,90.41) | 17.24 (6.16,58.08) | 21.59 (7.85,71.75) | 0.77 (0.62,0.92) | 0 |
| Somalia | 60.09 (18.18,175.32) | 141.36 (57.28,359.74) | 5.02 (1.51,14.67) | 4.20 (1.70,10.63) | -0.60 (-0.78,-0.42) | 0 |
| South Africa | 1319.36 (617.80,2048.22) | 1345.64 (641.62,2734.55) | 17.53 (8.19,27.17) | 10.92 (5.23,22.13) | -1.62 (-2.20,-1.04) | 0 |
| South Sudan | 47.01 (14.73,121.59) | 66.27 (28.49,150.29) | 4.97 (1.55,12.75) | 4.07 (1.75,9.17) | -0.68 (-1.02,-0.33) | 0 |
| Spain | 555.72 (349.38,864.07) | 560.74 (334.02,837.06) | 7.67 (4.83,11.93) | 8.23 (4.78,12.39) | 0.20 (-0.21,0.61) | 0.345 |
| Sri Lanka | 162.39 (71.88,315.32) | 157.05 (71.49,302.92) | 4.60 (2.04,8.93) | 3.62 (1.64,7.00) | -0.92 (-1.35,-0.49) | 0 |
| Sudan | 174.96 (79.23,331.68) | 560.98 (252.36,1144.17) | 4.88 (2.24,9.18) | 6.72 (3.05,13.66) | 1.16 (1.11,1.22) | 0 |
| Suriname | 6.88 (3.34,12.33) | 18.65 (8.48,35.77) | 9.73 (4.77,17.29) | 17.04 (7.73,32.74) | 2.03 (1.39,2.67) | 0 |
| Sweden | 58.16 (36.64,91.70) | 88.15 (53.69,128.82) | 3.89 (2.44,6.17) | 5.42 (3.29,7.99) | 1.12 (0.63,1.61) | 0 |
| Switzerland | 59.31 (33.73,96.79) | 68.69 (40.82,108.47) | 4.40 (2.49,7.24) | 4.65 (2.72,7.43) | 0.32 (-0.33,0.97) | 0.339 |
| Syrian Arab Republic | 117.68 (49.55,250.04) | 249.75 (109.59,531.49) | 5.46 (2.35,11.51) | 8.21 (3.72,17.29) | 1.43 (1.27,1.59) | 0 |
| Taiwan (Province of China) | 72.88 (44.57,120.21) | 114.67 (60.42,192.39) | 1.58 (0.97,2.60) | 2.68 (1.40,4.53) | 1.82 (1.55,2.09) | 0 |
| Tajikistan | 161.94 (73.08,323.57) | 295.79 (139.57,589.23) | 17.68 (8.11,34.85) | 15.33 (7.24,30.44) | -0.48 (-0.73,-0.24) | 0 |
| Thailand | 192.68 (90.76,379.89) | 293.07 (130.44,576.83) | 1.55 (0.73,3.05) | 2.26 (0.98,4.55) | 1.26 (0.86,1.66) | 0 |
| Timor-Leste | 2.70 (1.12,5.64) | 6.22 (1.63,13.42) | 1.91 (0.79,4.02) | 2.69 (0.68,5.90) | 1.27 (0.61,1.93) | 0 |
| Togo | 26.07 (13.00,50.49) | 61.08 (31.89,113.51) | 3.98 (1.99,7.69) | 3.78 (1.98,7.01) | -0.15 (-0.35,0.04) | 0.126 |
| Tokelau | 0.14 (0.06,0.28) | 0.10 (0.04,0.19) | 49.42 (20.97,101.91) | 43.01 (17.92,84.23) | -0.48 (-0.51,-0.45) | 0 |
| Tonga | 4.00 (1.97,7.24) | 4.41 (1.98,8.11) | 24.29 (12.11,43.66) | 23.16 (10.45,42.52) | -0.16 (-0.24,-0.08) | 0 |
| Trinidad and Tobago | 31.24 (15.48,56.38) | 43.48 (20.41,80.04) | 12.75 (6.32,23.11) | 15.60 (7.28,28.86) | 0.59 (0.12,1.06) | 0.015 |
| Tunisia | 91.12 (41.03,193.77) | 166.96 (77.39,334.70) | 5.77 (2.62,12.19) | 6.87 (3.17,13.83) | 0.61 (0.51,0.72) | 0 |
| Turkey | 1090.88 (553.36,2030.96) | 1386.35 (762.43,2302.89) | 9.34 (4.82,17.11) | 8.14 (4.45,13.56) | -0.47 (-0.71,-0.23) | 0 |
| Turkmenistan | 98.68 (50.25,177.36) | 73.83 (36.63,151.72) | 13.71 (7.04,24.54) | 7.72 (3.82,15.87) | -1.90 (-2.38,-1.42) | 0 |
| Tuvalu | 0.87 (0.38,1.90) | 0.92 (0.39,1.81) | 45.38 (19.91,99.25) | 42.64 (18.07,83.81) | -0.22 (-0.37,-0.07) | 0.003 |
| Uganda | 163.83 (47.48,461.45) | 429.47 (171.98,979.59) | 5.79 (1.67,16.10) | 5.83 (2.34,13.14) | -0.02 (-0.45,0.42) | 0.933 |
| Ukraine | 1450.37 (811.48,2342.36) | 723.66 (373.36,1431.75) | 14.42 (8.07,23.43) | 8.80 (4.53,18.18) | -1.66 (-2.26,-1.05) | 0 |
| United Arab Emirates | 25.18 (9.91,58.13) | 186.03 (74.26,405.45) | 8.55 (3.33,19.82) | 10.78 (4.28,23.44) | 0.80 (0.44,1.17) | 0 |
| United Kingdom | 525.87 (349.91,936.54) | 1051.23 (622.47,1326.40) | 4.94 (3.28,8.81) | 9.08 (5.38,11.44) | 2.07 (1.82,2.31) | 0 |
| United Republic of Tanzania | 173.17 (69.98,365.12) | 480.28 (215.67,988.87) | 3.88 (1.57,8.15) | 4.53 (2.04,9.30) | 0.55 (0.28,0.82) | 0 |
| United States of America | 9245.26 (6641.77,12118.79) | 7780.20 (5995.82,10797.00) | 16.92 (12.15,22.22) | 13.60 (10.47,18.89) | -0.73 (-0.87,-0.59) | 0 |
| United States Virgin Islands | 2.56 (1.23,4.80) | 2.00 (1.01,3.54) | 12.09 (5.78,22.70) | 11.95 (5.98,21.25) | -0.04 (-0.18,0.10) | 0.585 |
| Uruguay | 38.95 (20.32,73.68) | 76.49 (34.08,133.58) | 6.82 (3.56,12.90) | 12.30 (5.47,21.49) | 2.23 (1.86,2.60) | 0 |
| Uzbekistan | 1077.37 (465.99,1934.83) | 1194.17 (684.04,1921.55) | 26.68 (11.57,47.83) | 16.65 (9.56,26.81) | -1.58 (-1.81,-1.36) | 0 |
| Vanuatu | 8.90 (3.56,20.28) | 24.87 (10.10,51.66) | 31.75 (12.81,71.60) | 43.30 (17.65,89.70) | 1.04 (0.71,1.36) | 0 |
| Venezuela (Bolivarian Republic of) | 262.32 (144.35,462.24) | 535.68 (276.50,938.09) | 6.70 (3.70,11.81) | 9.66 (4.98,16.92) | 1.34 (0.99,1.69) | 0 |
| Viet Nam | 213.62 (97.12,438.05) | 358.06 (153.90,713.14) | 1.62 (0.73,3.35) | 1.70 (0.73,3.37) | 0.16 (0.10,0.22) | 0 |
| Yemen | 87.63 (40.35,170.54) | 364.85 (170.93,755.46) | 4.09 (1.89,7.93) | 5.88 (2.77,12.16) | 1.33 (1.18,1.47) | 0 |
| Zambia | 62.70 (22.84,145.71) | 199.99 (86.70,411.22) | 4.78 (1.73,11.06) | 5.73 (2.48,11.75) | 0.65 (0.47,0.83) | 0 |
| Zimbabwe | 67.93 (38.65,110.87) | 116.87 (64.28,193.72) | 3.78 (2.15,6.18) | 3.83 (2.11,6.34) | 0.07 (-0.23,0.38) | 0.649 |

NOTE:APPC,average annual percentage change;

| **Table 9.ASMR of interstitial lung disease and pulmonary sarcoidosis in adolescents and young adultsr in 1990 and 2019 for female and all nation, with AAPC from 2009 and 2019** | | | | | | |
| --- | --- | --- | --- | --- | --- | --- |
| location | Numbers in 1990 | Numbers in 2019 | Age-standardized rates in 1990   (95% CI) | Age standardized rates in 2019   (95% CI) | AAPC, %   (95% CI) | P |
| Afghanistan | 0.73 (0.16,1.87) | 3.22 (0.84,8.53) | 0.04 (0.01,0.10) | 0.05 (0.01,0.13) | 0.73 (0.37,1.08) | 0 |
| Albania | 0.65 (0.33,1.10) | 0.28 (0.12,0.61) | 0.10 (0.05,0.17) | 0.06 (0.03,0.13) | -1.75 (-2.03,-1.46) | 0 |
| Algeria | 2.64 (0.65,6.98) | 7.46 (2.32,17.88) | 0.06 (0.01,0.16) | 0.08 (0.03,0.20) | 1.14 (0.96,1.32) | 0 |
| American Samoa | 0.04 (0.02,0.07) | 0.03 (0.01,0.06) | 0.42 (0.20,0.80) | 0.32 (0.14,0.59) | -0.92 (-1.20,-0.63) | 0 |
| Andorra | 0.01 (0.00,0.02) | 0.02 (0.01,0.03) | 0.09 (0.03,0.17) | 0.12 (0.05,0.22) | 1.02 (0.81,1.23) | 0 |
| Angola | 1.11 (0.16,3.11) | 3.05 (0.85,7.50) | 0.06 (0.01,0.18) | 0.06 (0.02,0.14) | -0.38 (-0.59,-0.18) | 0 |
| Antigua and Barbuda | 0.01 (0.00,0.01) | 0.01 (0.01,0.02) | 0.05 (0.03,0.10) | 0.07 (0.04,0.12) | 1.05 (0.86,1.24) | 0 |
| Argentina | 14.24 (6.94,22.33) | 18.05 (9.41,28.56) | 0.23 (0.11,0.37) | 0.20 (0.10,0.32) | -0.50 (-0.92,-0.08) | 0.02 |
| Armenia | 1.00 (0.48,2.05) | 0.80 (0.43,1.78) | 0.14 (0.07,0.28) | 0.12 (0.07,0.28) | -0.45 (-0.77,-0.13) | 0.005 |
| Australia | 1.13 (0.60,2.16) | 3.37 (1.46,5.89) | 0.03 (0.02,0.06) | 0.07 (0.03,0.13) | 2.89 (2.53,3.26) | 0 |
| Austria | 0.53 (0.29,0.87) | 0.87 (0.43,1.45) | 0.04 (0.02,0.06) | 0.06 (0.03,0.10) | 1.82 (1.47,2.17) | 0 |
| Azerbaijan | 5.27 (2.61,9.33) | 4.98 (2.24,9.80) | 0.34 (0.17,0.60) | 0.23 (0.10,0.44) | -1.49 (-1.82,-1.16) | 0 |
| Bahamas | 0.13 (0.07,0.22) | 0.26 (0.12,0.46) | 0.23 (0.12,0.38) | 0.34 (0.15,0.58) | 1.38 (1.28,1.49) | 0 |
| Bahrain | 0.14 (0.05,0.31) | 0.48 (0.22,0.93) | 0.15 (0.06,0.32) | 0.18 (0.08,0.34) | 0.49 (-0.13,1.12) | 0.124 |
| Bangladesh | 19.35 (7.81,40.09) | 38.46 (14.06,95.89) | 0.11 (0.04,0.22) | 0.11 (0.04,0.28) | 0.09 (-0.41,0.58) | 0.736 |
| Barbados | 0.06 (0.03,0.11) | 0.09 (0.05,0.16) | 0.11 (0.06,0.19) | 0.17 (0.09,0.29) | 1.56 (1.02,2.10) | 0 |
| Belarus | 1.92 (0.90,3.44) | 0.63 (0.22,2.36) | 0.09 (0.04,0.17) | 0.04 (0.01,0.14) | -3.09 (-3.64,-2.55) | 0 |
| Belgium | 0.93 (0.49,1.56) | 1.30 (0.66,2.16) | 0.05 (0.03,0.08) | 0.07 (0.04,0.12) | 1.31 (1.07,1.54) | 0 |
| Belize | 0.07 (0.03,0.13) | 0.42 (0.22,0.69) | 0.23 (0.11,0.43) | 0.49 (0.26,0.80) | 2.89 (2.55,3.23) | 0 |
| Benin | 0.30 (0.10,0.70) | 0.79 (0.28,1.92) | 0.04 (0.01,0.08) | 0.03 (0.01,0.08) | -0.03 (-0.12,0.07) | 0.558 |
| Bermuda | 0.03 (0.01,0.05) | 0.02 (0.01,0.04) | 0.20 (0.10,0.35) | 0.23 (0.13,0.38) | 0.57 (0.46,0.68) | 0 |
| Bhutan | 0.12 (0.04,0.27) | 0.25 (0.09,0.52) | 0.12 (0.04,0.27) | 0.16 (0.06,0.32) | 0.99 (0.85,1.13) | 0 |
| Bolivia (Plurinational State of) | 3.37 (1.29,7.05) | 7.31 (2.89,13.63) | 0.29 (0.11,0.60) | 0.31 (0.12,0.58) | 0.31 (0.19,0.43) | 0 |
| Bosnia and Herzegovina | 0.56 (0.23,1.20) | 0.20 (0.07,0.54) | 0.06 (0.02,0.13) | 0.04 (0.01,0.10) | -1.60 (-2.06,-1.14) | 0 |
| Botswana | 0.36 (0.09,1.06) | 0.63 (0.21,1.45) | 0.15 (0.04,0.45) | 0.12 (0.04,0.26) | -0.97 (-1.21,-0.72) | 0 |
| Brazil | 39.37 (24.67,57.11) | 66.90 (42.08,96.58) | 0.13 (0.08,0.19) | 0.15 (0.09,0.21) | 0.47 (0.22,0.72) | 0 |
| Brunei Darussalam | 0.12 (0.05,0.24) | 0.13 (0.06,0.26) | 0.22 (0.09,0.43) | 0.14 (0.06,0.28) | -1.55 (-1.68,-1.42) | 0 |
| Bulgaria | 0.73 (0.36,1.26) | 0.48 (0.24,0.88) | 0.05 (0.02,0.08) | 0.04 (0.02,0.08) | -0.14 (-0.42,0.14) | 0.319 |
| Burkina Faso | 0.36 (0.12,0.85) | 1.04 (0.37,2.47) | 0.02 (0.01,0.05) | 0.03 (0.01,0.06) | 0.26 (-0.09,0.61) | 0.143 |
| Burundi | 0.75 (0.10,2.36) | 1.51 (0.39,4.51) | 0.08 (0.01,0.24) | 0.07 (0.02,0.21) | -0.34 (-0.67,0.00) | 0.048 |
| Cabo Verde | 0.03 (0.01,0.07) | 0.03 (0.01,0.07) | 0.05 (0.02,0.12) | 0.03 (0.01,0.05) | -2.26 (-2.52,-2.01) | 0 |
| Cambodia | 0.25 (0.08,0.58) | 0.69 (0.22,1.55) | 0.01 (0.00,0.03) | 0.02 (0.01,0.04) | 1.34 (1.25,1.42) | 0 |
| Cameroon | 0.90 (0.32,2.08) | 2.48 (0.91,5.33) | 0.05 (0.02,0.12) | 0.04 (0.02,0.09) | -0.51 (-0.70,-0.33) | 0 |
| Canada | 5.79 (2.86,11.73) | 8.08 (4.59,13.50) | 0.10 (0.05,0.19) | 0.13 (0.07,0.22) | 1.11 (0.58,1.63) | 0 |
| Central African Republic | 0.28 (0.04,0.86) | 0.42 (0.09,1.15) | 0.06 (0.01,0.18) | 0.04 (0.01,0.12) | -1.33 (-1.63,-1.03) | 0 |
| Chad | 0.40 (0.12,1.00) | 0.84 (0.31,1.94) | 0.04 (0.01,0.10) | 0.03 (0.01,0.07) | -0.72 (-0.93,-0.51) | 0 |
| Chile | 3.63 (1.66,8.24) | 5.60 (3.25,9.93) | 0.13 (0.06,0.29) | 0.16 (0.09,0.28) | 0.82 (0.68,0.97) | 0 |
| China | 83.42 (49.11,169.77) | 72.37 (41.26,116.53) | 0.03 (0.02,0.07) | 0.03 (0.02,0.04) | -0.60 (-1.00,-0.19) | 0.004 |
| Colombia | 2.78 (1.46,5.67) | 17.56 (4.86,36.30) | 0.04 (0.02,0.08) | 0.18 (0.05,0.37) | 5.51 (4.79,6.23) | 0 |
| Comoros | 0.04 (0.00,0.14) | 0.08 (0.02,0.22) | 0.06 (0.00,0.18) | 0.06 (0.01,0.15) | -0.43 (-3.28,2.50) | 0.77 |
| Congo | 0.23 (0.03,0.67) | 0.57 (0.14,1.43) | 0.05 (0.01,0.16) | 0.05 (0.01,0.13) | -0.05 (-0.23,0.12) | 0.567 |
| Cook Islands | 0.03 (0.01,0.05) | 0.01 (0.00,0.03) | 0.75 (0.34,1.37) | 0.42 (0.11,0.81) | -2.00 (-2.19,-1.82) | 0 |
| Costa Rica | 1.33 (0.72,2.28) | 2.75 (1.45,4.90) | 0.21 (0.11,0.36) | 0.27 (0.14,0.48) | 0.89 (0.02,1.76) | 0.044 |
| Coted'Ivoire | 0.66 (0.21,1.56) | 1.33 (0.48,3.02) | 0.03 (0.01,0.07) | 0.03 (0.01,0.06) | -0.73 (-1.05,-0.40) | 0 |
| Croatia | 0.37 (0.18,0.62) | 0.32 (0.16,0.57) | 0.04 (0.02,0.07) | 0.05 (0.02,0.08) | 0.63 (-0.16,1.42) | 0.12 |
| Cuba | 1.52 (0.64,2.72) | 1.36 (0.62,2.42) | 0.07 (0.03,0.12) | 0.07 (0.03,0.13) | 0.32 (-0.09,0.74) | 0.129 |
| Cyprus | 0.12 (0.04,0.31) | 0.18 (0.06,0.41) | 0.08 (0.02,0.20) | 0.07 (0.02,0.15) | -0.80 (-1.29,-0.30) | 0.002 |
| Czechia | 1.25 (0.69,2.12) | 1.53 (0.60,2.81) | 0.06 (0.03,0.11) | 0.09 (0.03,0.17) | 1.25 (0.80,1.70) | 0 |
| Democratic People's Republic of Korea | 1.79 (0.64,4.07) | 1.84 (0.68,4.00) | 0.04 (0.02,0.10) | 0.04 (0.01,0.08) | -0.57 (-0.68,-0.46) | 0 |
| Democratic Republic of the Congo | 4.73 (0.72,13.87) | 10.74 (2.63,29.53) | 0.07 (0.01,0.22) | 0.07 (0.02,0.19) | -0.26 (-0.71,0.19) | 0.26 |
| Denmark | 0.83 (0.44,1.53) | 1.40 (0.77,2.25) | 0.09 (0.05,0.16) | 0.16 (0.09,0.26) | 2.00 (1.64,2.37) | 0 |
| Djibouti | 0.05 (0.01,0.14) | 0.13 (0.03,0.34) | 0.06 (0.01,0.18) | 0.05 (0.01,0.13) | -0.70 (-1.03,-0.37) | 0 |
| Dominica | 0.01 (0.01,0.02) | 0.02 (0.01,0.04) | 0.09 (0.04,0.17) | 0.14 (0.06,0.28) | 1.69 (1.39,1.99) | 0 |
| Dominican Republic | 1.15 (0.52,2.15) | 3.05 (0.98,7.02) | 0.08 (0.04,0.15) | 0.14 (0.05,0.33) | 1.88 (1.42,2.34) | 0 |
| Ecuador | 3.84 (1.96,6.86) | 8.62 (4.34,14.75) | 0.20 (0.10,0.35) | 0.24 (0.12,0.42) | 0.62 (0.26,0.97) | 0.001 |
| Egypt | 7.29 (2.10,18.51) | 20.89 (6.19,50.37) | 0.07 (0.02,0.18) | 0.11 (0.03,0.26) | 1.35 (0.93,1.77) | 0 |
| El Salvador | 0.96 (0.40,2.00) | 1.70 (0.81,3.12) | 0.10 (0.04,0.20) | 0.13 (0.06,0.24) | 0.97 (0.61,1.33) | 0 |
| Equatorial Guinea | 0.04 (0.01,0.12) | 0.17 (0.04,0.49) | 0.05 (0.01,0.15) | 0.07 (0.02,0.19) | 0.65 (0.03,1.27) | 0.04 |
| Eritrea | 0.24 (0.04,0.73) | 0.66 (0.19,1.67) | 0.05 (0.01,0.14) | 0.05 (0.02,0.13) | 0.40 (0.20,0.60) | 0 |
| Estonia | 0.40 (0.19,0.69) | 0.23 (0.11,0.41) | 0.14 (0.06,0.24) | 0.11 (0.05,0.20) | -0.59 (-1.28,0.11) | 0.1 |
| Eswatini | 0.17 (0.04,0.51) | 0.21 (0.07,0.51) | 0.12 (0.03,0.36) | 0.09 (0.03,0.20) | -1.23 (-1.51,-0.95) | 0 |
| Ethiopia | 3.11 (0.58,7.81) | 9.85 (3.36,20.41) | 0.04 (0.01,0.09) | 0.05 (0.02,0.10) | 1.13 (0.92,1.34) | 0 |
| Fiji | 0.32 (0.15,0.63) | 0.35 (0.16,0.66) | 0.21 (0.10,0.41) | 0.20 (0.09,0.38) | -0.16 (-0.34,0.02) | 0.089 |
| Finland | 0.42 (0.19,0.89) | 0.51 (0.27,0.88) | 0.04 (0.02,0.09) | 0.06 (0.03,0.10) | 1.01 (0.75,1.27) | 0 |
| France | 6.84 (3.11,10.56) | 6.49 (3.56,10.67) | 0.06 (0.03,0.09) | 0.06 (0.03,0.10) | 0.14 (-0.23,0.52) | 0.463 |
| Gabon | 0.10 (0.02,0.27) | 0.20 (0.06,0.50) | 0.06 (0.01,0.17) | 0.05 (0.02,0.14) | -0.52 (-1.18,0.14) | 0.124 |
| Gambia | 0.06 (0.02,0.15) | 0.15 (0.06,0.37) | 0.03 (0.01,0.08) | 0.04 (0.01,0.08) | 0.19 (-0.70,1.08) | 0.681 |
| Georgia | 1.26 (0.65,2.02) | 0.81 (0.39,1.36) | 0.11 (0.06,0.18) | 0.12 (0.06,0.21) | 0.54 (-0.08,1.17) | 0.086 |
| Germany | 12.39 (5.70,19.96) | 9.21 (5.23,14.75) | 0.08 (0.04,0.13) | 0.07 (0.04,0.11) | -0.52 (-0.74,-0.30) | 0 |
| Ghana | 1.02 (0.43,1.99) | 2.97 (1.16,6.06) | 0.04 (0.02,0.07) | 0.04 (0.02,0.09) | 0.47 (0.10,0.85) | 0.013 |
| Greece | 0.47 (0.23,1.00) | 0.74 (0.39,1.23) | 0.02 (0.01,0.05) | 0.05 (0.03,0.08) | 2.28 (1.77,2.79) | 0 |
| Greenland | 0.02 (0.00,0.05) | 0.02 (0.01,0.06) | 0.14 (0.03,0.42) | 0.21 (0.06,0.55) | 1.45 (0.74,2.15) | 0 |
| Grenada | 0.02 (0.01,0.04) | 0.05 (0.02,0.08) | 0.15 (0.08,0.26) | 0.25 (0.12,0.43) | 1.85 (1.53,2.18) | 0 |
| Guam | 0.31 (0.17,0.52) | 0.29 (0.16,0.48) | 1.11 (0.61,1.83) | 1.05 (0.58,1.71) | -0.19 (-0.59,0.21) | 0.355 |
| Guatemala | 2.70 (1.23,5.00) | 7.19 (3.69,12.90) | 0.19 (0.09,0.36) | 0.18 (0.09,0.33) | -0.19 (-1.37,1.01) | 0.756 |
| Guinea | 0.36 (0.11,0.84) | 0.75 (0.27,1.76) | 0.03 (0.01,0.08) | 0.03 (0.01,0.07) | -0.22 (-0.37,-0.06) | 0.006 |
| Guinea-Bissau | 0.06 (0.02,0.15) | 0.13 (0.05,0.30) | 0.03 (0.01,0.08) | 0.03 (0.01,0.08) | -0.16 (-0.38,0.06) | 0.164 |
| Guyana | 0.17 (0.09,0.30) | 0.43 (0.19,0.78) | 0.11 (0.06,0.19) | 0.28 (0.12,0.51) | 3.26 (2.85,3.67) | 0 |
| Haiti | 1.39 (0.50,3.43) | 5.70 (2.17,13.18) | 0.12 (0.04,0.29) | 0.21 (0.08,0.48) | 2.00 (1.53,2.48) | 0 |
| Honduras | 2.31 (0.90,4.67) | 4.74 (1.53,10.52) | 0.29 (0.11,0.58) | 0.23 (0.07,0.51) | -0.80 (-1.42,-0.16) | 0.014 |
| Hungary | 1.98 (0.94,3.24) | 1.43 (0.73,2.42) | 0.10 (0.05,0.16) | 0.09 (0.05,0.15) | -0.27 (-0.63,0.10) | 0.152 |
| Iceland | 0.03 (0.02,0.05) | 0.07 (0.03,0.11) | 0.06 (0.03,0.11) | 0.11 (0.05,0.19) | 2.35 (1.95,2.75) | 0 |
| India | 221.93 (115.43,356.01) | 506.47 (291.22,777.79) | 0.15 (0.08,0.23) | 0.18 (0.10,0.28) | 0.73 (0.14,1.33) | 0.015 |
| Indonesia | 11.67 (6.24,23.00) | 22.44 (11.75,48.01) | 0.03 (0.02,0.07) | 0.04 (0.02,0.09) | 0.78 (0.68,0.87) | 0 |
| Iran (Islamic Republic of) | 2.75 (1.29,5.17) | 8.80 (3.58,13.11) | 0.03 (0.01,0.05) | 0.05 (0.02,0.07) | 1.73 (1.18,2.29) | 0 |
| Iraq | 1.95 (0.69,4.54) | 6.48 (2.03,14.65) | 0.07 (0.02,0.15) | 0.08 (0.02,0.17) | 0.60 (0.35,0.84) | 0 |
| Ireland | 0.55 (0.29,1.06) | 1.25 (0.65,2.01) | 0.08 (0.04,0.16) | 0.14 (0.07,0.23) | 1.85 (1.17,2.53) | 0 |
| Israel | 0.63 (0.29,1.24) | 1.08 (0.57,1.97) | 0.07 (0.03,0.13) | 0.07 (0.04,0.12) | 0.03 (-0.23,0.28) | 0.822 |
| Italy | 2.97 (2.05,5.56) | 5.75 (2.46,8.10) | 0.03 (0.02,0.05) | 0.07 (0.03,0.10) | 3.12 (2.82,3.43) | 0 |
| Jamaica | 0.39 (0.17,0.74) | 0.78 (0.34,1.47) | 0.09 (0.04,0.16) | 0.13 (0.06,0.24) | 1.49 (0.54,2.46) | 0.002 |
| Japan | 16.13 (10.79,27.46) | 12.08 (8.76,25.55) | 0.07 (0.05,0.12) | 0.07 (0.05,0.15) | -0.05 (-0.49,0.39) | 0.825 |
| Jordan | 1.13 (0.49,2.13) | 3.72 (1.93,6.35) | 0.18 (0.08,0.33) | 0.17 (0.09,0.29) | -0.19 (-0.56,0.18) | 0.32 |
| Kazakhstan | 5.93 (2.57,10.47) | 5.74 (2.19,11.48) | 0.18 (0.08,0.31) | 0.15 (0.06,0.30) | -0.50 (-1.01,0.00) | 0.05 |
| Kenya | 2.63 (0.50,6.69) | 7.67 (2.30,17.60) | 0.07 (0.01,0.19) | 0.08 (0.02,0.18) | 0.21 (-0.01,0.42) | 0.062 |
| Kiribati | 0.02 (0.01,0.04) | 0.04 (0.01,0.07) | 0.13 (0.05,0.26) | 0.15 (0.05,0.30) | 0.36 (0.22,0.51) | 0 |
| Kuwait | 0.23 (0.12,0.38) | 0.74 (0.39,1.40) | 0.06 (0.03,0.10) | 0.06 (0.03,0.11) | -0.30 (-0.82,0.22) | 0.26 |
| Kyrgyzstan | 0.60 (0.24,1.14) | 0.56 (0.22,1.50) | 0.07 (0.03,0.13) | 0.04 (0.02,0.12) | -1.66 (-2.21,-1.11) | 0 |
| Lao People's Democratic Republic | 0.21 (0.07,0.50) | 0.70 (0.22,1.58) | 0.03 (0.01,0.07) | 0.05 (0.02,0.11) | 1.64 (1.56,1.71) | 0 |
| Latvia | 0.63 (0.24,1.11) | 0.16 (0.07,0.38) | 0.13 (0.05,0.22) | 0.05 (0.02,0.13) | -2.84 (-3.27,-2.41) | 0 |
| Lebanon | 0.37 (0.10,0.94) | 0.81 (0.26,1.89) | 0.06 (0.02,0.16) | 0.07 (0.02,0.18) | 0.50 (0.40,0.61) | 0 |
| Lesotho | 0.46 (0.12,1.45) | 0.41 (0.13,0.95) | 0.15 (0.04,0.47) | 0.09 (0.03,0.22) | -1.60 (-2.09,-1.11) | 0 |
| Liberia | 0.07 (0.02,0.15) | 0.24 (0.08,0.58) | 0.02 (0.01,0.04) | 0.02 (0.01,0.06) | 0.77 (0.37,1.17) | 0 |
| Libya | 0.39 (0.10,1.03) | 1.17 (0.33,3.01) | 0.06 (0.02,0.16) | 0.08 (0.02,0.20) | 0.80 (0.59,1.02) | 0 |
| Lithuania | 0.45 (0.19,0.79) | 0.20 (0.09,0.40) | 0.06 (0.03,0.11) | 0.05 (0.02,0.09) | -1.08 (-1.57,-0.58) | 0 |
| Luxembourg | 0.03 (0.02,0.05) | 0.09 (0.04,0.16) | 0.04 (0.02,0.07) | 0.08 (0.04,0.14) | 2.69 (2.51,2.88) | 0 |
| Madagascar | 3.23 (0.55,9.22) | 6.81 (1.72,19.06) | 0.16 (0.03,0.45) | 0.14 (0.03,0.38) | -0.54 (-0.82,-0.26) | 0 |
| Malawi | 0.72 (0.14,1.95) | 1.72 (0.49,4.18) | 0.05 (0.01,0.12) | 0.05 (0.01,0.12) | 0.29 (0.05,0.53) | 0.019 |
| Malaysia | 2.17 (0.90,4.09) | 5.23 (2.39,9.88) | 0.06 (0.03,0.12) | 0.08 (0.04,0.15) | 0.90 (0.64,1.16) | 0 |
| Maldives | 0.13 (0.05,0.32) | 0.40 (0.20,0.68) | 0.41 (0.15,1.00) | 0.41 (0.21,0.70) | -0.03 (-0.22,0.16) | 0.791 |
| Mali | 0.79 (0.17,2.04) | 1.94 (0.47,5.77) | 0.05 (0.01,0.14) | 0.05 (0.01,0.15) | -0.14 (-0.42,0.14) | 0.32 |
| Malta | 0.06 (0.03,0.10) | 0.11 (0.06,0.16) | 0.08 (0.04,0.14) | 0.15 (0.08,0.23) | 2.33 (2.07,2.59) | 0 |
| Marshall Islands | 0.06 (0.02,0.12) | 0.08 (0.03,0.17) | 0.73 (0.28,1.52) | 0.70 (0.24,1.49) | -0.12 (-0.18,-0.07) | 0 |
| Mauritania | 0.10 (0.03,0.24) | 0.22 (0.08,0.47) | 0.03 (0.01,0.07) | 0.03 (0.01,0.06) | 0.16 (0.03,0.28) | 0.016 |
| Mauritius | 0.22 (0.11,0.42) | 0.47 (0.22,0.82) | 0.09 (0.05,0.18) | 0.18 (0.09,0.33) | 2.49 (1.71,3.28) | 0 |
| Mexico | 34.59 (23.00,51.48) | 61.25 (37.67,90.76) | 0.21 (0.14,0.31) | 0.24 (0.15,0.35) | 0.47 (0.26,0.68) | 0 |
| Micronesia (Federated States of) | 0.11 (0.04,0.22) | 0.13 (0.03,0.28) | 0.57 (0.23,1.21) | 0.65 (0.14,1.47) | 0.44 (0.39,0.50) | 0 |
| Monaco | 0.01 (0.00,0.01) | 0.01 (0.00,0.02) | 0.16 (0.08,0.28) | 0.18 (0.08,0.32) | 0.29 (0.15,0.42) | 0 |
| Mongolia | 0.77 (0.31,1.66) | 0.93 (0.36,2.13) | 0.20 (0.08,0.43) | 0.13 (0.05,0.29) | -1.63 (-1.78,-1.49) | 0 |
| Montenegro | 0.03 (0.02,0.06) | 0.03 (0.01,0.05) | 0.03 (0.01,0.05) | 0.02 (0.01,0.04) | -0.25 (-0.68,0.18) | 0.257 |
| Morocco | 1.87 (0.39,5.01) | 3.98 (1.10,10.41) | 0.04 (0.01,0.10) | 0.05 (0.01,0.14) | 1.14 (1.02,1.26) | 0 |
| Mozambique | 0.69 (0.15,1.84) | 1.98 (0.55,4.92) | 0.03 (0.01,0.08) | 0.04 (0.01,0.09) | 0.79 (0.60,0.99) | 0 |
| Myanmar | 3.84 (1.22,9.30) | 6.43 (2.15,14.80) | 0.05 (0.02,0.12) | 0.06 (0.02,0.13) | 0.41 (0.20,0.62) | 0 |
| Namibia | 0.28 (0.06,0.83) | 0.50 (0.17,1.23) | 0.11 (0.03,0.34) | 0.10 (0.03,0.25) | -0.42 (-0.79,-0.04) | 0.029 |
| Nauru | 0.01 (0.00,0.03) | 0.01 (0.00,0.03) | 0.61 (0.24,1.31) | 0.62 (0.20,1.28) | 0.02 (-0.11,0.15) | 0.815 |
| Nepal | 4.92 (1.68,10.25) | 15.81 (5.89,34.38) | 0.15 (0.05,0.32) | 0.25 (0.09,0.53) | 1.68 (1.61,1.75) | 0 |
| Netherlands | 1.12 (0.59,2.28) | 2.00 (0.79,3.52) | 0.04 (0.02,0.08) | 0.07 (0.03,0.13) | 2.50 (2.07,2.94) | 0 |
| New Zealand | 0.29 (0.17,0.49) | 0.61 (0.31,0.97) | 0.04 (0.02,0.07) | 0.08 (0.04,0.13) | 2.22 (1.41,3.04) | 0 |
| Nicaragua | 0.31 (0.15,0.60) | 1.22 (0.51,2.30) | 0.05 (0.02,0.09) | 0.09 (0.04,0.17) | 2.19 (1.75,2.63) | 0 |
| Niger | 0.59 (0.19,1.43) | 1.61 (0.53,4.15) | 0.05 (0.01,0.11) | 0.05 (0.02,0.12) | -0.05 (-0.37,0.26) | 0.736 |
| Nigeria | 4.10 (1.54,7.33) | 10.53 (4.75,19.72) | 0.03 (0.01,0.05) | 0.03 (0.01,0.05) | -0.21 (-0.40,-0.03) | 0.023 |
| Niue | 0.00 (0.00,0.01) | 0.00 (0.00,0.00) | 0.79 (0.33,1.52) | 0.59 (0.20,1.22) | -0.99 (-1.11,-0.87) | 0 |
| North Macedonia | 0.15 (0.06,0.31) | 0.12 (0.05,0.25) | 0.04 (0.02,0.08) | 0.03 (0.01,0.06) | -0.86 (-1.22,-0.50) | 0 |
| Northern Mariana Islands | 0.16 (0.08,0.29) | 0.05 (0.02,0.08) | 1.38 (0.65,2.47) | 0.86 (0.42,1.53) | -1.62 (-1.79,-1.44) | 0 |
| Norway | 0.46 (0.29,0.86) | 0.86 (0.50,1.18) | 0.06 (0.04,0.11) | 0.10 (0.06,0.13) | 1.91 (1.14,2.68) | 0 |
| Oman | 0.32 (0.07,0.87) | 0.85 (0.18,2.27) | 0.12 (0.03,0.32) | 0.11 (0.02,0.28) | -0.31 (-0.67,0.05) | 0.092 |
| Pakistan | 12.24 (5.41,26.72) | 38.82 (17.95,74.02) | 0.08 (0.03,0.16) | 0.10 (0.05,0.19) | 0.90 (0.64,1.16) | 0 |
| Palau | 0.02 (0.01,0.04) | 0.01 (0.01,0.03) | 0.60 (0.27,1.19) | 0.49 (0.21,0.97) | -0.70 (-0.74,-0.65) | 0 |
| Palestine | 1.15 (0.45,2.45) | 2.73 (1.33,4.63) | 0.35 (0.14,0.75) | 0.29 (0.14,0.50) | -0.56 (-0.70,-0.42) | 0 |
| Panama | 0.62 (0.33,1.06) | 1.75 (0.79,3.10) | 0.13 (0.07,0.22) | 0.22 (0.10,0.40) | 1.84 (1.06,2.62) | 0 |
| Papua New Guinea | 3.89 (1.42,8.92) | 10.95 (4.31,24.34) | 0.53 (0.20,1.21) | 0.57 (0.22,1.26) | 0.20 (0.06,0.35) | 0.006 |
| Paraguay | 0.76 (0.36,1.42) | 1.84 (0.79,3.46) | 0.10 (0.05,0.19) | 0.13 (0.05,0.24) | 0.69 (0.47,0.91) | 0 |
| Peru | 17.43 (8.38,30.96) | 26.22 (13.22,44.59) | 0.41 (0.20,0.73) | 0.38 (0.19,0.65) | -0.21 (-0.53,0.11) | 0.193 |
| Philippines | 2.07 (1.07,3.04) | 4.51 (2.06,7.18) | 0.02 (0.01,0.03) | 0.02 (0.01,0.03) | 0.45 (0.10,0.80) | 0.012 |
| Poland | 6.46 (3.14,8.63) | 5.29 (3.14,8.52) | 0.08 (0.04,0.11) | 0.07 (0.04,0.12) | -0.35 (-0.55,-0.15) | 0.001 |
| Portugal | 1.47 (0.77,2.51) | 2.06 (0.88,3.51) | 0.08 (0.04,0.13) | 0.13 (0.05,0.22) | 1.73 (1.29,2.18) | 0 |
| Puerto Rico | 1.29 (0.68,2.42) | 1.75 (0.89,3.10) | 0.18 (0.09,0.34) | 0.29 (0.15,0.52) | 1.73 (1.54,1.92) | 0 |
| Qatar | 0.04 (0.01,0.08) | 0.19 (0.06,0.47) | 0.05 (0.02,0.12) | 0.05 (0.02,0.11) | -0.44 (-0.84,-0.04) | 0.031 |
| Republic of Korea | 7.32 (3.70,13.32) | 5.21 (2.76,10.40) | 0.07 (0.04,0.13) | 0.06 (0.03,0.12) | -0.63 (-0.84,-0.42) | 0 |
| Republic of Moldova | 0.41 (0.22,0.78) | 0.34 (0.16,0.78) | 0.04 (0.02,0.08) | 0.05 (0.02,0.11) | 0.16 (-0.23,0.54) | 0.433 |
| Romania | 11.82 (6.23,18.47) | 3.03 (1.42,7.39) | 0.27 (0.14,0.42) | 0.10 (0.05,0.25) | -3.28 (-3.51,-3.04) | 0 |
| Russian Federation | 12.05 (6.93,18.34) | 10.39 (6.31,22.56) | 0.04 (0.02,0.06) | 0.04 (0.02,0.08) | -0.04 (-0.22,0.14) | 0.649 |
| Rwanda | 1.01 (0.14,2.96) | 2.46 (0.66,7.32) | 0.08 (0.01,0.24) | 0.10 (0.03,0.29) | 0.56 (0.30,0.82) | 0 |
| Saint Kitts and Nevis | 0.02 (0.01,0.03) | 0.02 (0.00,0.05) | 0.23 (0.11,0.41) | 0.19 (0.03,0.44) | -0.70 (-1.11,-0.28) | 0.001 |
| Saint Lucia | 0.04 (0.02,0.07) | 0.10 (0.05,0.16) | 0.16 (0.09,0.28) | 0.29 (0.16,0.47) | 2.02 (1.16,2.89) | 0 |
| Saint Vincent and the Grenadines | 0.01 (0.00,0.02) | 0.03 (0.01,0.04) | 0.04 (0.01,0.10) | 0.12 (0.06,0.21) | 4.12 (3.62,4.62) | 0 |
| Samoa | 0.25 (0.10,0.55) | 0.27 (0.09,0.56) | 0.91 (0.36,2.01) | 0.73 (0.23,1.48) | -0.76 (-0.83,-0.69) | 0 |
| San Marino | 0.00 (0.00,0.00) | 0.00 (0.00,0.00) | 0.02 (0.01,0.05) | 0.03 (0.01,0.08) | 1.07 (0.87,1.26) | 0 |
| Sao Tome and Principe | 0.01 (0.00,0.03) | 0.03 (0.01,0.07) | 0.07 (0.02,0.16) | 0.09 (0.03,0.18) | 0.59 (0.21,0.97) | 0.004 |
| Saudi Arabia | 8.49 (3.39,17.74) | 24.29 (9.78,46.29) | 0.35 (0.14,0.72) | 0.30 (0.12,0.56) | -0.58 (-1.07,-0.09) | 0.02 |
| Senegal | 0.48 (0.16,1.11) | 1.03 (0.38,2.34) | 0.04 (0.01,0.09) | 0.04 (0.01,0.08) | -0.12 (-0.78,0.54) | 0.716 |
| Serbia | 1.04 (0.46,1.95) | 0.73 (0.34,1.34) | 0.06 (0.03,0.11) | 0.05 (0.02,0.09) | -0.64 (-0.97,-0.30) | 0 |
| Seychelles | 0.01 (0.00,0.01) | 0.01 (0.00,0.02) | 0.04 (0.01,0.08) | 0.04 (0.01,0.08) | -0.11 (-0.32,0.11) | 0.33 |
| Sierra Leone | 0.18 (0.06,0.45) | 0.47 (0.17,1.14) | 0.03 (0.01,0.07) | 0.03 (0.01,0.07) | 0.13 (-0.09,0.34) | 0.256 |
| Singapore | 0.40 (0.14,0.70) | 0.51 (0.21,0.92) | 0.05 (0.02,0.09) | 0.05 (0.02,0.08) | -0.55 (-1.00,-0.10) | 0.017 |
| Slovakia | 0.93 (0.46,1.59) | 1.10 (0.41,2.09) | 0.09 (0.04,0.15) | 0.11 (0.04,0.21) | 0.85 (0.61,1.09) | 0 |
| Slovenia | 0.13 (0.05,0.32) | 0.13 (0.06,0.26) | 0.03 (0.01,0.08) | 0.04 (0.02,0.08) | 0.65 (0.19,1.11) | 0.006 |
| Solomon Islands | 0.15 (0.04,0.54) | 0.42 (0.12,1.49) | 0.26 (0.08,0.96) | 0.33 (0.10,1.19) | 0.82 (0.66,0.97) | 0 |
| Somalia | 0.76 (0.10,2.75) | 1.61 (0.35,5.31) | 0.06 (0.01,0.23) | 0.05 (0.01,0.16) | -0.90 (-1.03,-0.78) | 0 |
| South Africa | 19.39 (7.68,31.61) | 18.17 (6.58,42.72) | 0.26 (0.10,0.43) | 0.15 (0.05,0.34) | -2.00 (-2.68,-1.30) | 0 |
| South Sudan | 0.58 (0.09,1.84) | 0.74 (0.18,2.15) | 0.06 (0.01,0.20) | 0.05 (0.01,0.13) | -1.05 (-1.55,-0.55) | 0 |
| Spain | 8.43 (5.08,13.68) | 8.46 (4.73,12.92) | 0.12 (0.07,0.19) | 0.12 (0.07,0.19) | 0.16 (-0.32,0.65) | 0.507 |
| Sri Lanka | 2.65 (1.06,5.36) | 2.53 (1.03,5.11) | 0.08 (0.03,0.15) | 0.06 (0.02,0.12) | -0.95 (-1.48,-0.43) | 0 |
| Sudan | 1.75 (0.46,4.21) | 6.36 (1.72,15.92) | 0.05 (0.01,0.12) | 0.08 (0.02,0.19) | 1.62 (1.57,1.66) | 0 |
| Suriname | 0.11 (0.05,0.20) | 0.30 (0.13,0.60) | 0.16 (0.07,0.29) | 0.28 (0.12,0.54) | 2.10 (1.42,2.78) | 0 |
| Sweden | 0.77 (0.43,1.28) | 1.29 (0.72,1.95) | 0.05 (0.03,0.09) | 0.08 (0.04,0.12) | 1.61 (1.01,2.23) | 0 |
| Switzerland | 0.85 (0.44,1.48) | 0.99 (0.53,1.66) | 0.06 (0.03,0.11) | 0.07 (0.03,0.11) | 0.18 (-0.39,0.75) | 0.542 |
| Syrian Arab Republic | 1.24 (0.31,3.38) | 3.05 (0.92,7.65) | 0.06 (0.01,0.16) | 0.10 (0.03,0.25) | 1.88 (1.66,2.09) | 0 |
| Taiwan (Province of China) | 1.03 (0.58,1.84) | 1.61 (0.70,2.93) | 0.02 (0.01,0.04) | 0.04 (0.02,0.07) | 1.77 (1.57,1.96) | 0 |
| Tajikistan | 2.53 (1.02,5.32) | 4.57 (1.91,9.71) | 0.28 (0.12,0.58) | 0.24 (0.10,0.51) | -0.56 (-0.83,-0.28) | 0 |
| Thailand | 2.58 (0.97,5.77) | 4.32 (1.54,9.10) | 0.02 (0.01,0.05) | 0.03 (0.01,0.07) | 1.45 (0.97,1.93) | 0 |
| Timor-Leste | 0.04 (0.01,0.09) | 0.09 (0.01,0.22) | 0.03 (0.01,0.06) | 0.04 (0.01,0.10) | 1.42 (0.69,2.16) | 0 |
| Togo | 0.28 (0.09,0.67) | 0.61 (0.22,1.44) | 0.04 (0.01,0.10) | 0.04 (0.01,0.09) | -0.46 (-0.85,-0.07) | 0.022 |
| Tokelau | 0.00 (0.00,0.00) | 0.00 (0.00,0.00) | 0.82 (0.33,1.71) | 0.69 (0.26,1.39) | -0.57 (-0.61,-0.54) | 0 |
| Tonga | 0.06 (0.03,0.12) | 0.07 (0.03,0.13) | 0.38 (0.18,0.72) | 0.36 (0.14,0.69) | -0.24 (-0.34,-0.14) | 0 |
| Trinidad and Tobago | 0.50 (0.23,0.94) | 0.71 (0.31,1.35) | 0.21 (0.09,0.39) | 0.25 (0.11,0.48) | 0.57 (0.07,1.08) | 0.026 |
| Tunisia | 1.00 (0.27,2.74) | 1.94 (0.60,4.79) | 0.06 (0.02,0.18) | 0.08 (0.02,0.20) | 0.73 (0.60,0.86) | 0 |
| Turkey | 13.72 (5.64,28.66) | 16.32 (7.08,30.23) | 0.12 (0.05,0.24) | 0.10 (0.04,0.18) | -0.71 (-1.08,-0.33) | 0 |
| Turkmenistan | 1.49 (0.67,2.80) | 0.99 (0.40,2.33) | 0.21 (0.09,0.39) | 0.10 (0.04,0.24) | -2.35 (-2.91,-1.78) | 0 |
| Tuvalu | 0.01 (0.01,0.03) | 0.01 (0.01,0.03) | 0.74 (0.31,1.65) | 0.69 (0.27,1.38) | -0.28 (-0.43,-0.12) | 0 |
| Uganda | 2.11 (0.27,7.01) | 5.47 (1.42,14.67) | 0.08 (0.01,0.25) | 0.08 (0.02,0.20) | -0.11 (-0.38,0.15) | 0.409 |
| Ukraine | 19.23 (8.94,33.45) | 8.01 (3.35,18.48) | 0.19 (0.09,0.33) | 0.10 (0.04,0.24) | -2.28 (-3.17,-1.37) | 0 |
| United Arab Emirates | 0.33 (0.08,0.90) | 2.71 (0.76,6.66) | 0.11 (0.03,0.31) | 0.15 (0.04,0.36) | 0.91 (0.41,1.42) | 0 |
| United Kingdom | 7.20 (4.89,14.00) | 15.84 (8.62,19.78) | 0.07 (0.05,0.13) | 0.14 (0.07,0.17) | 2.49 (2.23,2.75) | 0 |
| United Republic of Tanzania | 1.90 (0.41,4.94) | 5.60 (1.62,13.57) | 0.04 (0.01,0.11) | 0.05 (0.02,0.13) | 0.70 (0.26,1.13) | 0.002 |
| United States of America | 127.89 (85.63,170.92) | 106.08 (77.69,157.49) | 0.23 (0.16,0.31) | 0.18 (0.13,0.27) | -0.79 (-0.90,-0.68) | 0 |
| United States Virgin Islands | 0.04 (0.02,0.08) | 0.03 (0.01,0.06) | 0.20 (0.08,0.38) | 0.19 (0.09,0.35) | -0.12 (-0.27,0.03) | 0.125 |
| Uruguay | 0.56 (0.25,1.15) | 1.18 (0.45,2.15) | 0.10 (0.04,0.20) | 0.19 (0.07,0.34) | 2.51 (2.08,2.94) | 0 |
| Uzbekistan | 16.75 (6.29,31.39) | 18.36 (9.53,30.68) | 0.42 (0.16,0.79) | 0.26 (0.13,0.43) | -1.66 (-1.90,-1.41) | 0 |
| Vanuatu | 0.14 (0.05,0.32) | 0.39 (0.15,0.84) | 0.50 (0.18,1.16) | 0.70 (0.26,1.48) | 1.12 (0.78,1.46) | 0 |
| Venezuela (Bolivarian Republic of) | 3.95 (2.03,7.26) | 8.32 (3.99,15.08) | 0.10 (0.05,0.19) | 0.15 (0.07,0.27) | 1.41 (1.04,1.78) | 0 |
| Viet Nam | 2.94 (1.01,6.74) | 5.12 (1.63,11.49) | 0.02 (0.01,0.05) | 0.02 (0.01,0.05) | 0.14 (0.05,0.22) | 0.001 |
| Yemen | 0.76 (0.17,2.07) | 3.94 (1.14,10.37) | 0.04 (0.01,0.10) | 0.06 (0.02,0.17) | 2.06 (1.83,2.30) | 0 |
| Zambia | 0.75 (0.15,2.14) | 2.56 (0.76,6.11) | 0.06 (0.01,0.17) | 0.08 (0.02,0.18) | 0.79 (0.58,1.01) | 0 |
| Zimbabwe | 0.47 (0.20,0.94) | 0.79 (0.26,1.74) | 0.03 (0.01,0.05) | 0.03 (0.01,0.06) | -0.09 (-0.60,0.43) | 0.738 |

NOTE:;ASMR,age-standardised mortality rate ;APPC,average annual percentage change;

| **Table 10.ASIR of interstitial lung disease and pulmonary sarcoidosis in adolescents and young adultsr in 1990 and 2019 for male and all nation, with AAPC from 2009 and 2019** | | | | | | |
| --- | --- | --- | --- | --- | --- | --- |
| location | Numbers in 1990 | Numbers in 2019 | Age-standardized rates in 1990   (95% CI) | Age standardized rates in 2019   (95% CI) | AAPC, %   (95% CI) | P |
| Afghanistan | 1612.57 (918.40,2615.94) | 8298.10 (4779.60,13296.65) | 111.48 (64.50,179.18) | 123.96 (72.20,198.39) | 0.38 (0.33,0.42) | 0 |
| Albania | 1287.27 (751.38,2077.98) | 1112.01 (638.72,1818.84) | 189.00 (110.65,305.66) | 226.10 (130.05,371.13) | 0.66 (0.53,0.79) | 0 |
| Algeria | 5229.36 (2968.94,8489.47) | 11559.95 (6745.94,18498.29) | 113.15 (64.83,184.28) | 126.39 (73.50,202.60) | 0.39 (0.35,0.43) | 0 |
| American Samoa | 14.42 (8.20,24.04) | 15.05 (8.60,24.88) | 150.95 (85.88,252.14) | 155.63 (89.31,257.21) | 0.10 (0.00,0.20) | 0.058 |
| Andorra | 2.59 (1.43,4.29) | 2.51 (1.41,4.16) | 17.80 (9.78,29.60) | 17.28 (9.57,28.64) | -0.09 (-0.22,0.04) | 0.172 |
| Angola | 1619.74 (922.40,2657.40) | 4720.27 (2710.56,7666.12) | 87.07 (49.90,143.08) | 96.43 (55.82,156.84) | 0.36 (0.32,0.40) | 0 |
| Antigua and Barbuda | 5.84 (3.27,9.63) | 9.17 (5.18,15.16) | 48.95 (27.54,80.85) | 53.07 (29.94,87.66) | 0.29 (0.23,0.35) | 0 |
| Argentina | 3784.36 (2170.38,6213.66) | 5787.89 (3278.42,9291.69) | 63.58 (36.51,104.33) | 66.12 (37.42,106.12) | 0.15 (0.09,0.22) | 0 |
| Armenia | 751.02 (439.28,1204.96) | 693.52 (400.35,1119.15) | 105.69 (61.80,170.44) | 113.34 (65.04,183.38) | 0.26 (0.21,0.32) | 0 |
| Australia | 2553.54 (1478.91,4202.24) | 3366.17 (1934.99,5421.26) | 73.48 (42.48,120.90) | 76.16 (43.65,122.86) | 0.12 (0.11,0.14) | 0 |
| Austria | 461.95 (269.27,734.89) | 381.34 (219.95,626.22) | 29.59 (17.24,47.15) | 24.46 (14.04,40.23) | -0.54 (-1.07,-0.01) | 0.046 |
| Azerbaijan | 1455.77 (835.14,2358.63) | 2567.84 (1500.47,4144.14) | 99.66 (57.30,162.47) | 110.53 (64.44,179.14) | 0.37 (0.33,0.41) | 0 |
| Bahamas | 28.02 (15.78,46.03) | 43.24 (25.19,69.64) | 51.49 (29.09,85.06) | 58.40 (34.01,94.04) | 0.43 (0.36,0.50) | 0 |
| Bahrain | 198.31 (114.88,316.70) | 645.57 (379.00,1057.62) | 113.58 (65.27,182.77) | 128.00 (73.95,206.14) | 0.42 (0.38,0.47) | 0 |
| Bangladesh | 15451.99 (9003.84,25075.64) | 30574.68 (17761.19,49092.03) | 80.36 (47.08,130.26) | 96.43 (56.11,154.88) | 0.63 (0.62,0.64) | 0 |
| Barbados | 27.00 (15.23,45.07) | 28.01 (15.97,46.05) | 50.45 (28.46,84.41) | 55.38 (31.54,90.94) | 0.32 (0.31,0.34) | 0 |
| Belarus | 4141.26 (2406.99,6694.75) | 3862.62 (2266.77,6269.00) | 197.20 (114.30,319.61) | 213.33 (124.45,346.71) | 0.29 (0.24,0.34) | 0 |
| Belgium | 571.20 (323.16,942.04) | 520.32 (288.26,857.13) | 28.84 (16.27,47.63) | 27.75 (15.30,45.76) | 0.00 (-0.30,0.30) | 0.996 |
| Belize | 23.67 (13.77,37.53) | 102.55 (61.29,161.33) | 73.08 (42.71,116.01) | 125.92 (75.44,198.10) | 1.86 (1.77,1.95) | 0 |
| Benin | 628.38 (358.87,1015.34) | 2031.32 (1156.68,3293.39) | 88.45 (50.78,143.06) | 95.21 (54.63,154.18) | 0.26 (0.22,0.31) | 0 |
| Bermuda | 8.83 (5.14,14.58) | 7.45 (4.41,11.99) | 64.40 (37.33,106.52) | 72.68 (42.76,116.77) | 0.42 (0.29,0.56) | 0 |
| Bhutan | 93.22 (53.25,153.92) | 178.72 (103.48,287.84) | 78.42 (45.32,129.71) | 96.70 (55.94,156.07) | 0.73 (0.68,0.78) | 0 |
| Bolivia (Plurinational State of) | 855.12 (499.90,1384.09) | 2121.90 (1251.03,3446.13) | 78.20 (46.05,126.54) | 90.74 (53.61,147.52) | 0.51 (0.50,0.52) | 0 |
| Bosnia and Herzegovina | 1949.55 (1126.25,3106.02) | 1276.18 (751.53,2078.94) | 191.96 (110.84,306.11) | 225.18 (132.19,365.75) | 0.59 (0.48,0.71) | 0 |
| Botswana | 285.62 (165.15,453.49) | 783.39 (458.74,1261.52) | 134.09 (78.31,212.72) | 146.13 (85.46,235.41) | 0.34 (0.27,0.41) | 0 |
| Brazil | 22000.72 (12722.38,35672.75) | 13122.93 (7680.91,20979.84) | 76.26 (44.29,123.86) | 29.57 (17.24,47.25) | -3.21 (-3.36,-3.06) | 0 |
| Brunei Darussalam | 64.89 (36.86,106.49) | 107.20 (60.31,176.84) | 96.79 (54.95,159.45) | 94.60 (53.06,156.28) | -0.03 (-0.15,0.09) | 0.595 |
| Bulgaria | 3035.64 (1748.38,4949.70) | 2747.46 (1603.95,4457.92) | 194.04 (111.55,315.61) | 227.44 (132.29,368.79) | 0.59 (0.43,0.75) | 0 |
| Burkina Faso | 1136.65 (647.99,1877.85) | 3515.97 (2001.64,5688.82) | 88.86 (51.18,146.67) | 95.88 (54.97,154.75) | 0.27 (0.23,0.31) | 0 |
| Burundi | 725.86 (417.05,1163.83) | 1724.51 (992.20,2774.02) | 77.81 (45.03,125.00) | 81.87 (47.30,131.72) | 0.19 (0.13,0.26) | 0 |
| Cabo Verde | 46.89 (26.26,76.59) | 126.91 (72.29,205.48) | 89.12 (50.43,146.01) | 96.72 (55.04,156.98) | 0.29 (0.23,0.35) | 0 |
| Cambodia | 372.09 (200.84,619.41) | 857.29 (474.27,1443.64) | 23.17 (12.68,38.80) | 24.36 (13.51,41.15) | 0.20 (0.12,0.27) | 0 |
| Cameroon | 1452.72 (824.51,2339.81) | 5181.38 (2960.56,8296.36) | 88.60 (50.58,142.60) | 94.45 (54.28,151.35) | 0.23 (0.19,0.27) | 0 |
| Canada | 7975.03 (4514.02,13299.67) | 7991.01 (4579.55,13313.53) | 132.68 (74.92,221.59) | 128.32 (73.38,213.56) | -0.20 (-0.68,0.29) | 0.434 |
| Central African Republic | 407.19 (232.58,653.74) | 869.68 (494.71,1410.89) | 86.83 (49.91,139.92) | 94.07 (53.88,152.46) | 0.31 (0.25,0.37) | 0 |
| Chad | 785.49 (446.64,1279.84) | 2283.29 (1300.89,3675.59) | 88.08 (50.51,143.59) | 94.51 (54.32,151.80) | 0.27 (0.18,0.36) | 0 |
| Chile | 1744.85 (1002.76,2817.88) | 2357.97 (1347.63,3926.74) | 64.62 (37.26,104.51) | 66.62 (37.98,111.13) | 0.09 (0.05,0.12) | 0 |
| China | 120512.27 (67286.75,204300.36) | 89734.85 (52196.98,146001.40) | 44.61 (25.00,75.50) | 31.72 (18.32,51.84) | -1.14 (-1.32,-0.95) | 0 |
| Colombia | 3867.46 (2224.29,6369.33) | 6134.96 (3531.84,9988.11) | 60.96 (35.20,100.74) | 64.83 (37.34,105.56) | 0.21 (0.18,0.25) | 0 |
| Comoros | 57.81 (32.90,94.99) | 117.55 (67.75,191.96) | 78.38 (45.11,128.84) | 82.16 (47.52,134.21) | 0.17 (0.13,0.20) | 0 |
| Congo | 368.98 (211.57,599.58) | 956.51 (548.23,1545.53) | 87.86 (50.69,143.08) | 95.81 (55.04,154.62) | 0.32 (0.27,0.37) | 0 |
| Cook Islands | 5.76 (3.25,9.53) | 4.48 (2.58,7.30) | 152.51 (86.08,252.06) | 165.73 (95.44,269.69) | 0.28 (0.23,0.34) | 0 |
| Costa Rica | 385.45 (223.15,640.17) | 672.83 (394.63,1097.03) | 64.33 (37.30,107.29) | 70.86 (41.50,115.70) | 0.32 (0.26,0.38) | 0 |
| Coted'Ivoire | 1983.40 (1134.07,3209.69) | 4944.99 (2845.27,8043.14) | 89.12 (51.22,144.45) | 94.47 (54.54,153.52) | 0.21 (0.17,0.26) | 0 |
| Croatia | 2262.28 (1303.17,3676.13) | 1927.71 (1140.86,3088.66) | 225.98 (129.86,366.94) | 260.22 (153.33,416.37) | 0.52 (0.43,0.60) | 0 |
| Cuba | 1128.81 (629.52,1843.50) | 1039.93 (583.86,1712.81) | 49.78 (27.97,81.34) | 54.16 (30.35,89.52) | 0.30 (0.27,0.33) | 0 |
| Cyprus | 24.53 (13.59,40.73) | 42.40 (23.47,69.10) | 15.33 (8.48,25.49) | 14.80 (8.13,24.26) | -0.09 (-0.17,-0.01) | 0.031 |
| Czechia | 3247.81 (1913.22,5333.90) | 3869.71 (2287.83,6356.27) | 163.86 (96.29,267.58) | 208.92 (122.67,342.24) | 0.88 (0.75,1.00) | 0 |
| Democratic People's Republic of Korea | 842.21 (464.83,1430.09) | 1259.17 (702.69,2136.66) | 22.31 (12.35,38.04) | 22.57 (12.55,38.35) | 0.03 (0.00,0.07) | 0.083 |
| Democratic Republic of the Congo | 5740.46 (3273.78,9310.93) | 15605.20 (8869.05,25243.67) | 88.73 (51.06,143.96) | 95.95 (54.92,154.79) | 0.29 (0.22,0.37) | 0 |
| Denmark | 89.48 (49.79,147.23) | 87.76 (48.32,145.66) | 8.89 (4.94,14.62) | 9.38 (5.16,15.58) | 0.17 (-0.18,0.53) | 0.342 |
| Djibouti | 78.52 (44.38,127.69) | 215.46 (125.22,343.43) | 79.47 (45.39,129.11) | 82.77 (48.13,131.98) | 0.15 (0.13,0.17) | 0 |
| Dominica | 6.96 (3.95,11.39) | 7.53 (4.33,12.31) | 50.17 (28.78,82.16) | 57.54 (33.09,94.16) | 0.48 (0.45,0.51) | 0 |
| Dominican Republic | 643.64 (356.92,1072.52) | 1181.66 (683.48,1924.03) | 48.33 (27.06,80.67) | 52.64 (30.46,85.79) | 0.32 (0.22,0.41) | 0 |
| Ecuador | 1247.84 (719.02,2025.66) | 2908.58 (1810.39,4328.06) | 68.38 (39.63,111.36) | 84.73 (52.79,126.01) | 0.76 (0.69,0.83) | 0 |
| Egypt | 11754.61 (6743.28,19201.54) | 26309.11 (15178.32,42329.91) | 112.66 (64.96,183.98) | 126.84 (73.24,204.01) | 0.42 (0.37,0.46) | 0 |
| El Salvador | 533.77 (306.74,881.53) | 726.04 (411.42,1199.63) | 61.21 (35.49,101.36) | 64.94 (37.00,107.56) | 0.21 (0.18,0.24) | 0 |
| Equatorial Guinea | 52.60 (29.68,85.23) | 317.13 (181.58,516.81) | 84.51 (48.09,136.28) | 98.61 (57.13,161.26) | 0.54 (0.51,0.57) | 0 |
| Eritrea | 401.22 (226.45,652.48) | 1108.44 (629.20,1816.64) | 76.89 (43.95,125.03) | 81.70 (46.59,133.95) | 0.22 (0.18,0.26) | 0 |
| Estonia | 626.10 (369.99,1002.86) | 508.27 (297.71,808.00) | 208.28 (122.84,333.92) | 212.89 (124.12,340.03) | 0.10 (0.03,0.16) | 0.004 |
| Eswatini | 157.09 (89.64,252.50) | 331.18 (195.21,534.96) | 132.86 (76.26,213.26) | 143.77 (85.23,232.23) | 0.29 (0.25,0.33) | 0 |
| Ethiopia | 6558.05 (3742.08,10607.79) | 17123.47 (9680.93,27801.72) | 80.54 (46.40,130.22) | 86.04 (49.16,140.01) | 0.25 (0.20,0.29) | 0 |
| Fiji | 216.16 (123.10,358.29) | 272.05 (155.12,442.80) | 138.15 (78.84,229.12) | 146.92 (83.72,238.93) | 0.21 (0.17,0.25) | 0 |
| Finland | 202.09 (113.88,335.05) | 178.99 (98.96,300.33) | 20.08 (11.23,33.34) | 19.37 (10.65,32.51) | -0.08 (-0.24,0.09) | 0.372 |
| France | 2006.84 (1099.31,3374.66) | 1749.73 (968.09,2912.54) | 17.61 (9.62,29.60) | 17.03 (9.39,28.32) | -0.07 (-0.22,0.09) | 0.393 |
| Gabon | 162.98 (93.85,263.17) | 325.76 (186.22,525.54) | 88.62 (51.30,143.15) | 96.24 (55.14,155.43) | 0.30 (0.25,0.35) | 0 |
| Gambia | 146.92 (83.52,236.86) | 388.92 (220.46,628.99) | 88.76 (50.82,143.63) | 94.37 (53.87,153.02) | 0.22 (0.18,0.26) | 0 |
| Georgia | 1093.31 (634.05,1785.29) | 568.43 (344.88,875.25) | 103.76 (60.16,169.72) | 87.35 (52.80,134.76) | -0.56 (-0.66,-0.47) | 0 |
| Germany | 2806.61 (1568.03,4596.76) | 2462.66 (1369.23,4072.75) | 17.46 (9.72,28.69) | 17.08 (9.44,28.34) | 0.07 (-0.13,0.26) | 0.502 |
| Ghana | 2261.77 (1295.02,3699.83) | 5904.90 (3360.83,9482.85) | 88.53 (51.18,144.79) | 96.06 (54.95,154.31) | 0.30 (0.25,0.34) | 0 |
| Greece | 312.13 (162.86,538.42) | 255.23 (142.48,418.18) | 16.41 (8.55,28.28) | 15.76 (8.72,25.88) | -0.10 (-0.23,0.02) | 0.105 |
| Greenland | 19.35 (11.02,32.30) | 13.52 (7.62,22.45) | 128.90 (73.24,216.11) | 123.92 (69.70,206.04) | -0.20 (-0.66,0.25) | 0.378 |
| Grenada | 7.82 (4.41,12.66) | 11.62 (6.66,18.95) | 50.25 (28.48,81.24) | 57.83 (33.24,94.68) | 0.48 (0.45,0.51) | 0 |
| Guam | 77.30 (45.84,120.75) | 73.81 (43.98,115.18) | 226.26 (134.35,353.46) | 239.20 (142.37,373.06) | 0.17 (0.14,0.21) | 0 |
| Guatemala | 828.81 (483.53,1328.35) | 2281.75 (1288.37,3766.12) | 65.30 (38.39,104.59) | 64.73 (36.72,107.19) | -0.03 (-0.04,-0.02) | 0 |
| Guinea | 813.34 (462.15,1316.67) | 1886.84 (1065.49,3046.16) | 88.30 (50.42,142.76) | 94.58 (53.76,152.37) | 0.27 (0.21,0.32) | 0 |
| Guinea-Bissau | 138.59 (79.64,223.87) | 329.45 (189.38,528.05) | 88.21 (51.11,142.52) | 93.53 (54.07,149.93) | 0.21 (0.18,0.25) | 0 |
| Guyana | 76.74 (43.07,126.38) | 84.97 (48.11,138.33) | 49.92 (28.19,82.33) | 57.34 (32.67,93.29) | 0.48 (0.45,0.51) | 0 |
| Haiti | 519.39 (297.21,845.85) | 1302.19 (734.28,2132.12) | 48.30 (27.78,78.61) | 53.38 (30.20,87.54) | 0.36 (0.31,0.40) | 0 |
| Honduras | 477.94 (276.69,777.30) | 1181.01 (678.82,1908.51) | 63.95 (37.31,103.93) | 64.65 (37.40,104.57) | 0.04 (0.02,0.06) | 0 |
| Hungary | 3900.14 (2278.86,6345.16) | 3688.14 (2164.21,5974.89) | 192.41 (111.83,311.92) | 227.34 (132.84,368.16) | 0.61 (0.44,0.78) | 0 |
| Iceland | 21.19 (11.67,34.55) | 26.10 (14.79,43.49) | 39.38 (21.67,64.28) | 40.12 (22.66,66.84) | 0.11 (-0.01,0.23) | 0.084 |
| India | 181159.25 (106480.26,288879.65) | 369399.60 (216592.37,591032.22) | 109.60 (64.71,174.67) | 123.33 (72.43,197.35) | 0.42 (0.38,0.46) | 0 |
| Indonesia | 10356.26 (5695.94,17211.70) | 16621.81 (9269.40,27695.93) | 28.37 (15.69,47.22) | 30.51 (16.99,50.73) | 0.26 (0.22,0.30) | 0 |
| Iran (Islamic Republic of) | 13048.07 (7509.56,20978.04) | 28981.99 (17051.90,46782.17) | 125.78 (72.88,202.61) | 143.61 (83.88,231.63) | 0.48 (0.41,0.56) | 0 |
| Iraq | 3425.76 (1980.28,5442.46) | 11320.44 (6478.43,18389.75) | 113.00 (65.81,179.02) | 125.90 (72.47,205.10) | 0.38 (0.34,0.42) | 0 |
| Ireland | 106.41 (58.79,180.01) | 136.81 (76.94,231.22) | 15.83 (8.77,26.74) | 16.09 (9.01,27.12) | 0.03 (-0.22,0.29) | 0.81 |
| Israel | 91.05 (49.61,151.75) | 162.53 (88.10,269.96) | 9.83 (5.37,16.34) | 9.86 (5.34,16.36) | 0.02 (-0.01,0.06) | 0.184 |
| Italy | 3927.41 (2213.94,6475.21) | 3032.50 (1765.24,4820.72) | 36.56 (20.62,60.30) | 34.63 (20.06,55.07) | -0.13 (-0.27,0.01) | 0.078 |
| Jamaica | 214.04 (120.04,354.61) | 311.51 (177.62,514.09) | 49.60 (28.00,82.37) | 54.39 (31.11,89.90) | 0.32 (0.30,0.35) | 0 |
| Japan | 30351.69 (17314.43,50217.56) | 22662.76 (13295.87,36917.58) | 132.91 (75.79,218.95) | 127.18 (74.38,206.33) | -0.12 (-0.16,-0.08) | 0 |
| Jordan | 835.08 (486.43,1344.54) | 3518.60 (2077.06,5631.54) | 119.62 (70.39,193.69) | 132.36 (78.27,211.89) | 0.36 (0.33,0.38) | 0 |
| Kazakhstan | 3413.14 (2013.43,5462.49) | 4139.27 (2412.98,6701.39) | 100.71 (59.40,161.70) | 112.11 (65.08,182.19) | 0.38 (0.29,0.47) | 0 |
| Kenya | 3274.57 (1874.28,5272.26) | 8750.20 (5024.89,14100.16) | 85.65 (49.56,138.18) | 88.19 (50.98,142.24) | 0.12 (0.07,0.16) | 0 |
| Kiribati | 22.03 (12.73,35.94) | 37.32 (21.14,59.89) | 158.03 (91.48,257.37) | 161.56 (91.53,259.07) | 0.10 (-0.17,0.38) | 0.476 |
| Kuwait | 642.51 (378.31,1037.71) | 1660.60 (983.21,2685.17) | 115.13 (67.43,186.74) | 127.90 (74.88,206.94) | 0.37 (0.34,0.41) | 0 |
| Kyrgyzstan | 847.36 (488.99,1373.17) | 1450.00 (831.46,2355.59) | 99.57 (57.66,162.04) | 109.80 (62.93,179.07) | 0.36 (0.33,0.39) | 0 |
| Lao People's Democratic Republic | 159.02 (86.83,264.77) | 382.25 (208.76,634.78) | 23.19 (12.76,38.64) | 24.83 (13.59,41.34) | 0.23 (0.19,0.27) | 0 |
| Latvia | 1118.13 (651.18,1784.05) | 778.90 (452.66,1266.99) | 225.07 (130.92,359.65) | 237.83 (137.67,386.98) | 0.28 (0.14,0.43) | 0 |
| Lebanon | 615.08 (352.15,993.45) | 1407.80 (824.49,2258.80) | 114.03 (65.74,184.16) | 128.79 (75.04,207.67) | 0.43 (0.38,0.47) | 0 |
| Lesotho | 400.35 (230.38,652.07) | 670.24 (384.51,1065.25) | 132.38 (76.79,215.37) | 141.66 (81.38,225.19) | 0.25 (0.20,0.29) | 0 |
| Liberia | 263.80 (148.67,430.41) | 863.27 (491.80,1412.68) | 89.36 (50.51,145.10) | 95.37 (54.56,155.82) | 0.23 (0.20,0.27) | 0 |
| Libya | 916.06 (528.06,1474.54) | 2069.25 (1198.20,3347.41) | 113.24 (65.62,182.19) | 124.96 (72.15,201.91) | 0.35 (0.31,0.40) | 0 |
| Lithuania | 1268.25 (738.30,2018.16) | 860.27 (502.29,1391.75) | 178.74 (104.02,285.41) | 187.35 (109.13,303.90) | 0.15 (-0.15,0.45) | 0.331 |
| Luxembourg | 13.60 (7.56,23.01) | 19.73 (11.02,32.60) | 16.80 (9.28,28.51) | 16.53 (9.17,27.37) | 0.03 (-0.09,0.15) | 0.625 |
| Madagascar | 1658.73 (970.01,2670.91) | 4208.99 (2424.27,6845.23) | 81.50 (47.94,131.57) | 84.60 (49.08,137.71) | 0.14 (0.11,0.16) | 0 |
| Malawi | 1237.39 (696.10,1994.90) | 2679.05 (1514.65,4367.01) | 78.09 (44.26,125.99) | 82.01 (46.71,133.83) | 0.20 (0.13,0.26) | 0 |
| Malaysia | 854.14 (464.17,1423.83) | 1924.28 (1060.52,3213.89) | 23.98 (13.08,40.05) | 26.23 (14.42,43.92) | 0.29 (0.21,0.37) | 0 |
| Maldives | 18.88 (10.98,30.00) | 75.10 (43.39,124.77) | 55.25 (32.31,87.90) | 39.23 (22.47,65.40) | -1.24 (-1.42,-1.07) | 0 |
| Mali | 1125.26 (633.06,1817.35) | 3275.52 (1867.12,5296.32) | 88.10 (50.03,141.83) | 95.76 (55.05,154.62) | 0.31 (0.23,0.39) | 0 |
| Malta | 13.24 (7.42,21.89) | 14.87 (8.34,24.12) | 17.88 (9.98,29.63) | 18.76 (10.43,30.55) | 0.19 (0.10,0.27) | 0 |
| Marshall Islands | 14.71 (8.69,23.85) | 22.69 (13.20,36.15) | 181.30 (107.32,293.61) | 184.95 (107.66,294.55) | 0.06 (0.02,0.09) | 0.002 |
| Mauritania | 304.99 (174.82,492.05) | 668.89 (378.98,1081.98) | 88.80 (51.22,142.89) | 95.08 (54.24,153.42) | 0.25 (0.22,0.28) | 0 |
| Mauritius | 65.50 (36.23,108.60) | 74.65 (41.04,127.19) | 26.04 (14.40,43.27) | 30.27 (16.62,51.38) | 0.50 (0.45,0.56) | 0 |
| Mexico | 12670.01 (7250.38,20701.33) | 15991.69 (9525.36,25179.69) | 82.05 (47.36,134.22) | 65.79 (39.23,103.58) | -0.78 (-0.86,-0.70) | 0 |
| Micronesia (Federated States of) | 33.35 (19.42,53.32) | 36.99 (21.45,59.64) | 175.69 (102.51,280.24) | 180.41 (104.77,289.61) | 0.07 (0.03,0.11) | 0.001 |
| Monaco | 0.88 (0.49,1.48) | 0.81 (0.44,1.34) | 17.72 (9.76,29.70) | 16.88 (9.22,28.12) | -0.14 (-0.29,0.02) | 0.096 |
| Mongolia | 414.84 (239.09,658.64) | 841.58 (489.54,1353.67) | 105.95 (61.33,168.64) | 114.92 (66.54,185.54) | 0.29 (0.23,0.35) | 0 |
| Montenegro | 259.71 (149.65,421.71) | 264.36 (154.36,435.01) | 197.67 (113.87,321.05) | 229.41 (133.75,376.54) | 0.55 (0.43,0.66) | 0 |
| Morocco | 5374.53 (3074.17,8721.08) | 9232.76 (5350.76,14789.35) | 112.79 (64.91,183.35) | 125.35 (72.63,200.81) | 0.37 (0.31,0.44) | 0 |
| Mozambique | 1460.08 (834.24,2385.93) | 3834.23 (2184.53,6170.73) | 77.14 (44.38,125.96) | 82.04 (47.11,131.83) | 0.22 (0.16,0.28) | 0 |
| Myanmar | 1846.31 (1017.29,3119.30) | 2742.40 (1509.99,4611.28) | 23.47 (12.99,39.68) | 25.91 (14.28,43.55) | 0.34 (0.32,0.36) | 0 |
| Namibia | 322.66 (186.11,529.46) | 686.57 (398.89,1108.88) | 133.82 (77.95,220.30) | 144.70 (84.33,234.23) | 0.28 (0.23,0.33) | 0 |
| Nauru | 3.22 (1.86,5.29) | 3.68 (2.14,5.90) | 164.46 (95.17,270.68) | 166.72 (97.20,267.18) | 0.07 (-0.15,0.28) | 0.547 |
| Nepal | 2279.02 (1329.54,3677.08) | 3927.87 (2274.12,6309.90) | 70.88 (41.63,114.19) | 74.40 (43.52,119.29) | 0.15 (0.09,0.22) | 0 |
| Netherlands | 555.13 (307.80,918.67) | 466.31 (258.00,779.58) | 17.48 (9.67,28.98) | 17.02 (9.40,28.47) | -0.05 (-0.15,0.05) | 0.353 |
| New Zealand | 618.69 (352.18,1014.64) | 366.38 (223.03,565.98) | 89.77 (51.10,147.25) | 52.62 (32.03,81.25) | -1.86 (-2.02,-1.69) | 0 |
| Nicaragua | 371.50 (210.35,605.10) | 879.82 (500.45,1449.27) | 60.46 (34.55,98.66) | 65.24 (37.23,107.71) | 0.25 (0.21,0.30) | 0 |
| Niger | 1069.84 (611.47,1740.33) | 3309.43 (1881.21,5349.74) | 88.20 (50.72,143.46) | 94.85 (54.35,152.81) | 0.26 (0.23,0.29) | 0 |
| Nigeria | 15363.47 (8816.43,24797.24) | 36951.88 (21144.57,59607.44) | 96.67 (55.79,156.14) | 102.73 (59.27,165.80) | 0.22 (0.16,0.29) | 0 |
| Niue | 0.61 (0.36,1.00) | 0.47 (0.26,0.76) | 154.67 (90.30,252.81) | 165.74 (94.23,270.53) | 0.23 (0.18,0.28) | 0 |
| North Macedonia | 819.72 (477.48,1326.32) | 1032.00 (605.44,1690.39) | 194.61 (113.32,314.91) | 228.14 (133.41,373.59) | 0.58 (0.41,0.75) | 0 |
| Northern Mariana Islands | 23.90 (14.12,37.89) | 12.55 (7.20,20.09) | 190.75 (112.30,303.01) | 192.63 (110.64,308.33) | -0.01 (-0.08,0.06) | 0.739 |
| Norway | 893.44 (515.59,1450.44) | 1004.63 (576.96,1625.77) | 106.54 (61.41,172.92) | 105.74 (60.58,171.19) | 0.08 (-0.14,0.30) | 0.466 |
| Oman | 620.98 (365.43,993.00) | 2625.77 (1530.31,4185.47) | 114.24 (66.97,183.57) | 126.74 (73.25,203.74) | 0.37 (0.32,0.42) | 0 |
| Pakistan | 16955.28 (9827.48,27357.76) | 39570.61 (23113.06,63427.47) | 89.97 (52.65,144.99) | 94.21 (55.46,150.77) | 0.19 (0.12,0.25) | 0 |
| Palau | 10.18 (6.06,15.98) | 11.45 (6.90,18.00) | 288.23 (171.66,452.64) | 293.24 (176.54,461.38) | 0.04 (0.01,0.08) | 0.021 |
| Palestine | 397.11 (230.87,626.22) | 1255.99 (722.50,2046.13) | 117.50 (69.01,184.84) | 129.12 (74.55,211.76) | 0.33 (0.31,0.35) | 0 |
| Panama | 288.34 (165.53,473.53) | 523.01 (303.06,858.01) | 61.52 (35.45,101.17) | 65.65 (38.07,107.70) | 0.22 (0.20,0.25) | 0 |
| Papua New Guinea | 1408.98 (824.80,2218.64) | 4085.33 (2428.16,6481.88) | 177.68 (104.00,279.35) | 203.83 (121.17,323.06) | 0.45 (0.34,0.57) | 0 |
| Paraguay | 254.67 (141.52,424.71) | 524.03 (292.61,870.24) | 34.49 (19.25,57.63) | 35.20 (19.67,58.57) | 0.08 (0.01,0.15) | 0.034 |
| Peru | 3494.40 (2054.73,5589.87) | 7568.49 (4480.51,12104.96) | 89.93 (53.26,144.12) | 111.62 (66.10,178.59) | 0.74 (0.68,0.80) | 0 |
| Philippines | 3391.60 (1869.93,5671.44) | 7138.78 (3948.47,11904.00) | 27.94 (15.53,46.78) | 31.20 (17.31,52.05) | 0.39 (0.35,0.42) | 0 |
| Poland | 19650.21 (11648.70,31531.29) | 20358.06 (13008.24,29906.70) | 244.38 (144.27,391.77) | 263.07 (167.52,386.33) | 0.31 (0.17,0.45) | 0 |
| Portugal | 295.05 (162.89,496.11) | 266.74 (149.51,436.31) | 15.89 (8.79,26.70) | 16.65 (9.31,27.15) | 0.19 (0.13,0.25) | 0 |
| Puerto Rico | 366.38 (209.57,613.65) | 352.29 (203.47,566.09) | 55.63 (31.89,93.13) | 62.91 (36.28,100.94) | 0.45 (0.31,0.58) | 0 |
| Qatar | 227.22 (131.76,371.26) | 2028.89 (1173.92,3279.30) | 114.17 (65.64,187.55) | 128.06 (73.56,209.37) | 0.42 (0.37,0.47) | 0 |
| Republic of Korea | 3844.67 (2187.66,6227.04) | 4130.86 (2342.09,6847.40) | 35.86 (20.42,58.21) | 41.83 (23.59,69.18) | 0.54 (0.39,0.68) | 0 |
| Republic of Moldova | 1824.14 (1063.38,2916.11) | 1637.61 (945.78,2630.89) | 200.60 (116.62,321.07) | 211.37 (121.21,339.35) | 0.20 (0.13,0.27) | 0 |
| Romania | 9130.18 (5470.65,14632.51) | 7302.34 (4330.83,11792.85) | 203.50 (121.61,326.01) | 225.53 (133.17,364.02) | 0.39 (0.22,0.56) | 0 |
| Russian Federation | 65920.60 (38780.16,105825.06) | 61895.36 (36434.86,98884.45) | 207.49 (121.62,333.65) | 220.43 (129.06,352.64) | 0.23 (0.19,0.28) | 0 |
| Rwanda | 965.02 (547.71,1564.74) | 2097.80 (1187.35,3402.28) | 77.45 (44.17,125.63) | 83.34 (47.41,135.29) | 0.27 (0.24,0.30) | 0 |
| Saint Kitts and Nevis | 4.07 (2.33,6.66) | 6.47 (3.72,10.78) | 50.08 (28.73,82.01) | 54.61 (31.30,90.85) | 0.30 (0.27,0.32) | 0 |
| Saint Lucia | 12.99 (7.35,21.32) | 23.26 (13.48,37.80) | 53.64 (30.73,88.27) | 66.96 (38.73,108.69) | 0.77 (0.68,0.85) | 0 |
| Saint Vincent and the Grenadines | 10.32 (5.77,16.98) | 11.86 (6.79,19.71) | 49.61 (27.98,81.78) | 56.04 (32.08,92.96) | 0.43 (0.39,0.48) | 0 |
| Samoa | 52.41 (30.50,83.15) | 66.59 (38.21,109.31) | 168.06 (97.69,268.20) | 167.84 (96.67,275.25) | 0.00 (-0.15,0.15) | 1 |
| San Marino | 0.79 (0.44,1.31) | 0.84 (0.46,1.40) | 17.33 (9.62,28.82) | 16.69 (9.18,27.83) | -0.08 (-0.26,0.10) | 0.372 |
| Sao Tome and Principe | 16.23 (9.36,26.26) | 40.10 (23.07,65.99) | 89.06 (52.15,144.01) | 96.71 (55.77,159.25) | 0.31 (0.23,0.38) | 0 |
| Saudi Arabia | 4312.15 (2496.29,6889.73) | 16251.68 (9747.07,25820.23) | 114.60 (66.51,183.55) | 132.37 (78.69,210.42) | 0.50 (0.48,0.52) | 0 |
| Senegal | 1004.59 (568.26,1633.70) | 2635.76 (1510.60,4276.46) | 85.23 (48.69,138.84) | 95.54 (55.25,154.99) | 0.41 (0.37,0.44) | 0 |
| Serbia | 2937.42 (1720.30,4741.65) | 2955.11 (1726.32,4803.64) | 158.82 (92.80,256.49) | 188.79 (110.03,306.12) | 0.64 (0.45,0.82) | 0 |
| Seychelles | 3.59 (1.95,6.06) | 5.78 (3.15,9.90) | 23.88 (12.99,40.55) | 25.13 (13.59,42.99) | 0.18 (0.16,0.19) | 0 |
| Sierra Leone | 532.78 (302.22,856.89) | 1477.17 (844.03,2422.23) | 88.64 (50.53,142.52) | 94.87 (54.63,155.75) | 0.24 (0.21,0.28) | 0 |
| Singapore | 1700.10 (960.95,2814.46) | 2773.67 (1587.91,4654.55) | 216.47 (122.27,359.46) | 218.31 (124.48,363.22) | 0.08 (-0.11,0.27) | 0.391 |
| Slovakia | 2118.86 (1227.28,3415.31) | 2432.83 (1415.33,4029.84) | 191.92 (110.90,308.98) | 225.50 (130.68,372.63) | 0.59 (0.46,0.73) | 0 |
| Slovenia | 891.20 (518.40,1440.12) | 927.07 (547.05,1533.40) | 210.89 (122.33,340.08) | 250.88 (146.93,412.78) | 0.65 (0.48,0.82) | 0 |
| Solomon Islands | 85.62 (48.67,139.36) | 189.78 (108.20,306.32) | 146.63 (83.95,238.10) | 152.71 (87.12,245.90) | 0.14 (0.01,0.27) | 0.039 |
| Somalia | 930.63 (534.69,1521.89) | 2943.56 (1675.30,4765.99) | 78.15 (45.16,127.12) | 80.81 (46.41,130.41) | 0.13 (0.06,0.19) | 0 |
| South Africa | 11233.66 (6628.67,17825.92) | 18845.85 (11095.75,30019.54) | 155.53 (92.33,246.95) | 151.54 (89.06,241.95) | -0.05 (-0.17,0.07) | 0.426 |
| South Sudan | 859.28 (487.00,1386.32) | 1143.95 (645.91,1910.37) | 78.39 (44.86,126.63) | 81.05 (46.49,134.51) | 0.12 (0.08,0.17) | 0 |
| Spain | 1310.54 (739.16,2153.90) | 1443.87 (813.00,2427.48) | 17.87 (10.10,29.37) | 19.77 (11.06,33.14) | 0.40 (0.27,0.53) | 0 |
| Sri Lanka | 861.91 (472.00,1447.04) | 1012.84 (560.08,1704.35) | 23.96 (13.16,40.30) | 25.28 (13.96,42.39) | 0.18 (0.16,0.21) | 0 |
| Sudan | 3712.50 (2120.10,6060.32) | 9625.68 (5571.54,15427.93) | 111.75 (64.42,182.14) | 124.94 (72.76,200.17) | 0.39 (0.36,0.42) | 0 |
| Suriname | 37.93 (21.63,62.66) | 62.90 (35.65,103.27) | 50.99 (29.36,84.87) | 59.14 (33.54,96.98) | 0.51 (0.49,0.53) | 0 |
| Sweden | 380.77 (212.77,630.83) | 428.68 (240.59,705.74) | 24.51 (13.67,40.63) | 24.57 (13.74,40.54) | 0.01 (-0.23,0.26) | 0.915 |
| Switzerland | 253.12 (140.22,427.73) | 263.73 (149.28,434.79) | 17.58 (9.70,29.76) | 16.84 (9.46,27.85) | -0.05 (-0.43,0.33) | 0.79 |
| Syrian Arab Republic | 2425.72 (1381.57,3956.25) | 2801.12 (1608.23,4666.64) | 113.29 (65.22,184.49) | 127.38 (73.69,208.87) | 0.41 (0.39,0.43) | 0 |
| Taiwan (Province of China) | 932.95 (513.23,1616.65) | 1427.21 (841.55,2326.65) | 19.45 (10.69,33.75) | 31.64 (18.51,51.36) | 1.73 (1.66,1.80) | 0 |
| Tajikistan | 1195.83 (696.43,1890.75) | 2641.27 (1574.66,4205.09) | 131.34 (77.04,207.20) | 134.14 (80.00,214.55) | 0.07 (0.03,0.10) | 0 |
| Thailand | 2891.84 (1593.98,4869.35) | 3278.07 (1799.80,5431.03) | 23.58 (13.03,39.85) | 25.41 (13.87,42.03) | 0.25 (0.21,0.30) | 0 |
| Timor-Leste | 35.89 (19.68,60.96) | 57.64 (31.02,96.77) | 23.34 (12.86,39.82) | 24.78 (13.52,41.73) | 0.22 (0.18,0.25) | 0 |
| Togo | 516.80 (291.25,824.98) | 1343.54 (771.64,2164.69) | 89.34 (50.76,142.54) | 94.91 (54.90,152.98) | 0.22 (0.19,0.25) | 0 |
| Tokelau | 0.39 (0.22,0.64) | 0.39 (0.22,0.65) | 143.26 (82.01,232.87) | 158.32 (89.77,262.47) | 0.32 (0.18,0.46) | 0 |
| Tonga | 23.88 (13.67,39.02) | 28.87 (16.73,47.36) | 150.52 (86.63,246.22) | 165.45 (96.09,270.91) | 0.32 (0.19,0.45) | 0 |
| Trinidad and Tobago | 128.45 (73.05,210.36) | 169.01 (99.00,273.19) | 52.69 (30.06,86.48) | 58.86 (34.17,95.08) | 0.39 (0.35,0.43) | 0 |
| Tunisia | 1808.59 (1028.45,2939.64) | 2952.71 (1706.54,4815.89) | 113.81 (65.08,185.67) | 126.69 (72.83,205.74) | 0.38 (0.33,0.42) | 0 |
| Turkey | 13267.81 (7655.34,21468.21) | 22721.48 (13148.41,37031.01) | 112.37 (65.11,182.43) | 124.39 (71.82,202.71) | 0.34 (0.29,0.40) | 0 |
| Turkmenistan | 700.76 (405.04,1125.46) | 1225.96 (717.15,1958.46) | 100.48 (58.38,162.07) | 110.55 (64.59,177.19) | 0.34 (0.31,0.38) | 0 |
| Tuvalu | 2.38 (1.39,3.87) | 3.83 (2.20,6.32) | 145.04 (84.80,236.45) | 155.07 (89.35,255.99) | 0.22 (0.16,0.28) | 0 |
| Uganda | 2114.36 (1201.52,3402.12) | 5624.70 (3178.75,9032.34) | 77.30 (44.40,125.09) | 83.12 (47.50,133.55) | 0.27 (0.24,0.31) | 0 |
| Ukraine | 22906.53 (13668.66,35954.17) | 17338.40 (10300.68,27559.08) | 228.74 (136.05,359.24) | 206.33 (121.70,328.62) | -0.33 (-0.59,-0.06) | 0.016 |
| United Arab Emirates | 918.17 (543.56,1454.17) | 6355.67 (3809.65,10109.49) | 118.54 (69.40,188.59) | 133.67 (78.18,213.43) | 0.43 (0.40,0.45) | 0 |
| United Kingdom | 1497.74 (845.97,2441.21) | 2070.21 (1189.99,3350.58) | 14.13 (7.97,23.06) | 17.48 (9.98,28.37) | 0.75 (0.65,0.85) | 0 |
| United Republic of Tanzania | 3170.42 (1819.83,5174.33) | 7872.71 (4478.18,12998.67) | 78.04 (45.29,127.57) | 82.72 (47.45,136.77) | 0.24 (0.19,0.29) | 0 |
| United States of America | 92575.42 (54735.41,150933.27) | 93359.04 (57464.47,142645.43) | 169.66 (100.05,276.91) | 161.31 (99.17,246.39) | -0.19 (-0.43,0.05) | 0.117 |
| United States Virgin Islands | 10.20 (5.74,16.73) | 10.61 (6.12,17.14) | 55.11 (31.06,90.08) | 70.53 (40.66,113.61) | 0.87 (0.74,0.99) | 0 |
| Uruguay | 341.72 (196.72,561.59) | 387.87 (221.65,645.04) | 61.54 (35.46,101.21) | 64.32 (36.75,106.79) | 0.19 (0.04,0.34) | 0.014 |
| Uzbekistan | 4817.94 (2853.73,7639.07) | 8461.84 (4968.25,13507.44) | 122.91 (73.18,195.15) | 118.03 (69.30,188.94) | -0.14 (-0.25,-0.04) | 0.008 |
| Vanuatu | 43.37 (25.38,70.34) | 89.24 (52.02,141.83) | 159.65 (93.60,259.63) | 161.73 (94.52,257.07) | 0.02 (-0.15,0.18) | 0.831 |
| Venezuela (Bolivarian Republic of) | 2295.47 (1303.27,3811.62) | 3397.38 (1943.15,5557.28) | 61.84 (35.23,102.86) | 63.57 (36.28,103.70) | 0.10 (0.07,0.13) | 0 |
| Viet Nam | 2994.99 (1642.49,4989.60) | 5259.15 (2920.72,8835.23) | 23.47 (12.99,39.14) | 25.00 (13.81,42.10) | 0.22 (0.19,0.25) | 0 |
| Yemen | 2312.99 (1335.69,3784.50) | 7657.98 (4415.19,12490.07) | 112.10 (65.21,183.55) | 123.64 (71.59,201.67) | 0.35 (0.31,0.38) | 0 |
| Zambia | 985.67 (560.39,1601.28) | 2782.71 (1592.82,4454.74) | 77.88 (44.79,126.23) | 82.32 (47.39,131.48) | 0.21 (0.14,0.28) | 0 |
| Zimbabwe | 2275.04 (1308.56,3624.71) | 3913.85 (2249.94,6370.74) | 137.06 (79.43,218.15) | 143.72 (83.01,233.43) | 0.17 (0.13,0.21) | 0 |

NOTE:ASIRs,age-standardised incidence rates;APPC,average annual percentage change;

| **Table 11.DALYs of interstitial lung disease and pulmonary sarcoidosis in adolescents and young adultsr in 1990 and 2019 for male and all nation, with AAPC from 2009 and 2019** | | | | | | |
| --- | --- | --- | --- | --- | --- | --- |
| location | Numbers in 1990 | Numbers in 2019 | Age-standardized rates in 1990   (95% CI) | Age standardized rates in 2019   (95% CI) | AAPC, %   (95% CI) | P |
| Afghanistan | 86.98 (31.40,254.22) | 387.27 (172.84,827.71) | 5.70 (2.17,16.39) | 5.60 (2.55,11.89) | -0.05 (-0.26,0.16) | 0.638 |
| Albania | 58.48 (31.96,98.32) | 37.33 (20.51,63.17) | 8.60 (4.69,14.43) | 7.55 (4.13,12.81) | -0.49 (-0.87,-0.11) | 0.011 |
| Algeria | 249.45 (109.39,601.89) | 524.49 (256.24,956.36) | 5.25 (2.35,12.42) | 5.77 (2.80,10.58) | 0.34 (0.18,0.50) | 0 |
| American Samoa | 3.99 (1.95,7.45) | 3.78 (1.83,7.04) | 40.92 (20.04,75.80) | 37.70 (18.38,70.15) | -0.28 (-0.50,-0.06) | 0.014 |
| Andorra | 1.07 (0.47,2.06) | 1.62 (0.61,3.24) | 7.41 (3.22,14.27) | 11.02 (4.09,22.13) | 1.38 (1.27,1.50) | 0 |
| Angola | 118.60 (40.58,295.53) | 271.90 (125.19,532.24) | 6.38 (2.19,15.76) | 5.56 (2.58,10.83) | -0.54 (-0.91,-0.18) | 0.003 |
| Antigua and Barbuda | 0.21 (0.13,0.34) | 0.50 (0.29,0.79) | 1.77 (1.06,2.83) | 2.90 (1.67,4.60) | 1.92 (0.79,3.06) | 0.001 |
| Argentina | 670.12 (385.12,1067.08) | 1104.53 (558.95,1844.96) | 11.22 (6.46,17.84) | 12.64 (6.38,21.15) | 0.42 (-0.12,0.97) | 0.125 |
| Armenia | 66.74 (33.17,121.03) | 43.16 (23.42,71.26) | 9.34 (4.60,17.09) | 6.91 (3.70,11.60) | -0.98 (-1.81,-0.15) | 0.021 |
| Australia | 141.63 (82.82,234.95) | 341.81 (160.20,582.15) | 4.07 (2.39,6.74) | 7.75 (3.58,13.30) | 2.26 (1.45,3.07) | 0 |
| Austria | 77.81 (47.34,121.55) | 83.20 (46.13,133.62) | 5.10 (3.10,7.94) | 5.41 (2.96,8.76) | 0.22 (-0.19,0.64) | 0.29 |
| Azerbaijan | 87.30 (46.15,157.04) | 114.44 (54.55,212.90) | 6.04 (3.18,10.85) | 4.95 (2.36,9.24) | -0.71 (-1.04,-0.37) | 0 |
| Bahamas | 4.02 (2.25,6.89) | 10.77 (5.22,19.00) | 7.37 (4.12,12.62) | 14.57 (7.07,25.70) | 2.48 (2.02,2.95) | 0 |
| Bahrain | 11.59 (5.68,22.36) | 34.89 (19.69,58.78) | 6.84 (3.31,13.34) | 8.48 (4.67,14.54) | 0.79 (0.24,1.34) | 0.005 |
| Bangladesh | 3558.98 (1347.15,7419.57) | 4303.14 (1844.89,7920.58) | 18.16 (6.92,37.56) | 13.48 (5.79,24.81) | -1.01 (-1.60,-0.41) | 0.001 |
| Barbados | 2.27 (1.27,3.79) | 3.62 (2.05,5.95) | 4.23 (2.37,7.08) | 7.24 (4.09,11.92) | 1.77 (1.22,2.32) | 0 |
| Belarus | 184.83 (96.10,299.56) | 108.68 (49.39,193.43) | 8.82 (4.60,14.29) | 5.95 (2.74,10.54) | -1.33 (-1.71,-0.96) | 0 |
| Belgium | 101.26 (62.13,167.27) | 112.49 (60.23,178.72) | 5.10 (3.12,8.45) | 5.97 (3.18,9.51) | 0.59 (0.16,1.02) | 0.008 |
| Belize | 8.85 (4.44,15.06) | 66.24 (34.42,107.92) | 25.53 (12.68,43.57) | 78.68 (41.03,127.99) | 4.08 (3.57,4.59) | 0 |
| Benin | 29.10 (13.68,57.51) | 75.26 (41.36,128.51) | 4.12 (1.94,8.11) | 3.54 (1.95,6.00) | -0.51 (-0.59,-0.44) | 0 |
| Bermuda | 2.81 (1.49,4.68) | 2.52 (1.45,3.90) | 21.10 (11.15,35.13) | 26.58 (15.20,41.10) | 0.87 (0.60,1.14) | 0 |
| Bhutan | 12.37 (4.89,25.47) | 14.93 (6.31,29.83) | 10.43 (4.13,21.36) | 8.03 (3.40,16.06) | -0.92 (-1.01,-0.83) | 0 |
| Bolivia (Plurinational State of) | 289.11 (110.87,580.34) | 531.92 (207.83,1011.33) | 25.66 (10.01,51.28) | 22.39 (8.77,42.45) | -0.46 (-0.55,-0.38) | 0 |
| Bosnia and Herzegovina | 68.32 (32.38,120.02) | 35.03 (17.35,60.97) | 6.75 (3.19,11.88) | 6.14 (3.07,10.65) | -0.37 (-0.58,-0.17) | 0 |
| Botswana | 21.77 (8.57,47.48) | 46.70 (21.98,88.62) | 10.56 (4.11,23.21) | 8.63 (4.07,16.36) | -0.66 (-0.83,-0.48) | 0 |
| Brazil | 3489.12 (2389.75,4597.37) | 4694.70 (2982.72,6071.60) | 12.03 (8.24,15.87) | 10.61 (6.74,13.73) | -0.44 (-0.79,-0.08) | 0.015 |
| Brunei Darussalam | 16.05 (7.37,31.17) | 18.94 (10.90,30.65) | 24.21 (10.98,47.20) | 16.68 (9.58,27.01) | -1.26 (-1.81,-0.71) | 0 |
| Bulgaria | 96.16 (51.54,161.96) | 70.32 (35.05,122.24) | 6.20 (3.35,10.37) | 5.94 (3.02,10.25) | -0.02 (-0.22,0.19) | 0.86 |
| Burkina Faso | 29.49 (14.91,52.32) | 91.06 (50.78,152.45) | 2.35 (1.19,4.14) | 2.51 (1.40,4.17) | 0.25 (0.05,0.46) | 0.016 |
| Burundi | 89.87 (23.37,239.38) | 161.39 (63.09,356.98) | 9.36 (2.44,24.73) | 7.58 (2.98,16.73) | -0.75 (-1.08,-0.42) | 0 |
| Cabo Verde | 2.70 (1.39,4.83) | 3.39 (1.91,5.67) | 5.25 (2.73,9.35) | 2.58 (1.45,4.30) | -2.48 (-2.90,-2.05) | 0 |
| Cambodia | 38.56 (13.61,99.33) | 78.43 (30.62,172.06) | 2.20 (0.80,5.61) | 2.19 (0.86,4.79) | -0.02 (-0.10,0.07) | 0.688 |
| Cameroon | 77.80 (37.60,150.74) | 226.84 (123.14,403.47) | 4.78 (2.33,9.24) | 4.16 (2.26,7.36) | -0.48 (-0.71,-0.25) | 0 |
| Canada | 593.42 (350.63,964.01) | 676.14 (439.16,983.54) | 9.91 (5.84,16.14) | 11.00 (7.12,16.01) | 0.39 (0.09,0.69) | 0.011 |
| Central African Republic | 37.54 (10.13,114.55) | 67.94 (22.21,205.24) | 7.83 (2.09,24.06) | 7.22 (2.34,21.94) | -0.33 (-0.50,-0.16) | 0 |
| Chad | 34.96 (16.20,71.37) | 90.98 (48.71,165.47) | 3.95 (1.84,8.03) | 3.80 (2.04,6.87) | 0.01 (-0.15,0.17) | 0.926 |
| Chile | 301.82 (166.65,580.27) | 412.76 (258.36,616.31) | 11.34 (6.33,21.61) | 11.66 (7.29,17.42) | 0.11 (-0.22,0.44) | 0.52 |
| China | 8952.59 (6073.18,13435.01) | 7214.15 (5091.62,9859.36) | 3.26 (2.22,4.90) | 2.59 (1.83,3.56) | -0.76 (-1.08,-0.45) | 0 |
| Colombia | 258.85 (136.05,531.92) | 1069.22 (355.94,2155.34) | 3.98 (2.10,8.12) | 11.23 (3.74,22.63) | 3.68 (3.15,4.21) | 0 |
| Comoros | 5.26 (1.27,14.07) | 8.99 (3.55,19.06) | 7.10 (1.78,18.55) | 6.29 (2.49,13.32) | -0.41 (-2.50,1.73) | 0.705 |
| Congo | 38.12 (10.75,113.06) | 64.38 (29.20,130.16) | 9.13 (2.55,27.30) | 6.48 (2.94,13.09) | -1.20 (-1.79,-0.60) | 0 |
| Cook Islands | 1.67 (0.78,3.00) | 0.79 (0.39,1.37) | 42.44 (20.00,76.47) | 29.14 (14.47,50.75) | -1.31 (-1.44,-1.19) | 0 |
| Costa Rica | 62.82 (35.41,100.12) | 164.44 (92.14,268.75) | 10.21 (5.79,16.17) | 17.22 (9.62,28.23) | 2.01 (1.20,2.82) | 0 |
| Coted'Ivoire | 89.89 (41.53,180.57) | 173.97 (96.23,292.42) | 4.00 (1.86,7.97) | 3.34 (1.85,5.61) | -0.60 (-0.73,-0.46) | 0 |
| Croatia | 65.85 (33.92,111.14) | 52.90 (23.63,96.46) | 6.60 (3.44,11.07) | 7.18 (3.27,13.01) | 0.22 (-0.25,0.70) | 0.357 |
| Cuba | 104.67 (53.20,190.61) | 93.89 (46.34,165.70) | 4.51 (2.34,8.11) | 4.94 (2.41,8.75) | 0.32 (-0.24,0.88) | 0.262 |
| Cyprus | 8.21 (2.92,21.24) | 12.55 (4.71,29.71) | 5.17 (1.84,13.33) | 4.51 (1.64,10.88) | -0.39 (-0.66,-0.12) | 0.005 |
| Czechia | 111.51 (64.37,188.86) | 153.00 (68.95,264.89) | 5.57 (3.19,9.48) | 8.52 (3.92,14.70) | 1.50 (1.14,1.87) | 0 |
| Democratic People's Republic of Korea | 155.46 (59.78,359.62) | 195.93 (81.09,409.48) | 4.07 (1.57,9.39) | 3.55 (1.46,7.46) | -0.48 (-0.53,-0.43) | 0 |
| Democratic Republic of the Congo | 462.19 (164.30,1107.53) | 1191.96 (493.35,2675.54) | 7.27 (2.58,17.42) | 7.38 (3.05,16.49) | -0.01 (-0.19,0.18) | 0.952 |
| Denmark | 88.16 (44.99,152.12) | 88.24 (46.32,143.90) | 8.74 (4.44,15.15) | 9.38 (4.91,15.38) | 0.19 (-0.39,0.78) | 0.522 |
| Djibouti | 7.36 (2.32,17.45) | 15.26 (5.83,33.08) | 7.26 (2.30,17.00) | 5.85 (2.24,12.69) | -0.77 (-1.00,-0.53) | 0 |
| Dominica | 0.87 (0.41,1.60) | 1.33 (0.63,2.50) | 6.18 (2.92,11.33) | 10.13 (4.84,19.03) | 1.70 (1.52,1.89) | 0 |
| Dominican Republic | 50.58 (24.49,90.15) | 116.96 (54.58,239.22) | 3.72 (1.83,6.56) | 5.20 (2.43,10.64) | 1.24 (0.76,1.71) | 0 |
| Ecuador | 307.41 (151.49,554.89) | 880.49 (441.10,1490.60) | 16.27 (8.09,29.28) | 24.94 (12.57,42.13) | 1.31 (0.69,1.94) | 0 |
| Egypt | 727.10 (269.99,1744.36) | 1681.79 (726.70,3339.20) | 6.81 (2.59,16.29) | 8.09 (3.50,16.03) | 0.66 (0.53,0.79) | 0 |
| El Salvador | 92.60 (44.15,181.24) | 116.41 (56.59,214.19) | 10.40 (5.09,19.95) | 10.32 (5.01,18.97) | 0.02 (-0.85,0.90) | 0.969 |
| Equatorial Guinea | 5.05 (1.38,16.00) | 22.22 (9.98,45.01) | 8.01 (2.19,25.40) | 6.97 (3.13,14.13) | -0.50 (-0.82,-0.18) | 0.002 |
| Eritrea | 43.38 (11.04,127.28) | 107.80 (38.59,249.65) | 7.97 (2.03,23.37) | 7.87 (2.81,18.22) | -0.01 (-0.22,0.20) | 0.928 |
| Estonia | 40.24 (20.46,65.70) | 26.48 (14.31,42.84) | 13.36 (6.83,21.80) | 10.96 (5.97,17.73) | -0.88 (-2.09,0.33) | 0.154 |
| Eswatini | 9.67 (4.38,19.25) | 20.26 (9.81,38.71) | 8.58 (3.87,17.12) | 8.96 (4.31,17.22) | 0.13 (-0.02,0.29) | 0.084 |
| Ethiopia | 456.02 (150.93,954.29) | 989.71 (475.48,1692.08) | 5.58 (1.85,11.67) | 5.01 (2.39,8.55) | -0.36 (-0.55,-0.18) | 0 |
| Fiji | 40.37 (20.13,71.09) | 43.92 (23.03,74.80) | 25.02 (12.52,43.99) | 23.90 (12.53,40.72) | -0.14 (-0.33,0.06) | 0.163 |
| Finland | 56.24 (26.86,122.58) | 44.23 (24.84,73.79) | 5.48 (2.53,12.19) | 4.71 (2.59,8.01) | -0.52 (-0.74,-0.30) | 0 |
| France | 571.91 (373.81,941.30) | 503.38 (297.28,775.85) | 5.00 (3.26,8.23) | 4.88 (2.86,7.54) | -0.13 (-0.73,0.48) | 0.683 |
| Gabon | 14.39 (4.68,34.38) | 21.29 (9.39,45.33) | 7.88 (2.56,18.75) | 6.30 (2.78,13.38) | -0.84 (-1.10,-0.57) | 0 |
| Gambia | 6.26 (2.92,12.34) | 15.36 (8.30,26.63) | 3.75 (1.77,7.36) | 3.72 (2.01,6.44) | 0.04 (-0.69,0.78) | 0.916 |
| Georgia | 79.26 (45.42,127.79) | 75.57 (37.78,131.74) | 7.58 (4.33,12.24) | 11.81 (5.79,20.78) | 1.57 (0.13,3.02) | 0.032 |
| Germany | 1051.17 (597.26,1818.56) | 893.24 (538.20,1357.54) | 6.66 (3.77,11.53) | 6.26 (3.73,9.58) | -0.15 (-0.42,0.11) | 0.26 |
| Ghana | 126.93 (57.86,240.87) | 327.60 (154.32,597.95) | 5.02 (2.30,9.50) | 5.32 (2.51,9.69) | 0.19 (-0.17,0.55) | 0.305 |
| Greece | 45.42 (25.71,80.17) | 98.95 (40.85,182.36) | 2.38 (1.35,4.21) | 6.06 (2.48,11.19) | 3.39 (2.35,4.44) | 0 |
| Greenland | 1.57 (0.73,3.57) | 1.36 (0.67,2.51) | 10.53 (4.91,23.66) | 12.46 (6.13,23.11) | 0.65 (0.16,1.15) | 0.01 |
| Grenada | 0.97 (0.50,1.68) | 1.76 (0.91,3.09) | 6.26 (3.28,10.82) | 8.68 (4.52,15.12) | 0.96 (0.10,1.83) | 0.029 |
| Guam | 41.46 (24.20,65.86) | 39.54 (23.13,62.14) | 122.82 (71.83,194.93) | 128.89 (75.38,202.66) | 0.13 (-0.31,0.57) | 0.559 |
| Guatemala | 306.95 (160.15,601.55) | 572.46 (310.79,955.44) | 23.27 (12.17,46.08) | 15.48 (8.51,25.69) | -1.34 (-2.53,-0.13) | 0.03 |
| Guinea | 34.15 (16.52,67.62) | 72.63 (39.57,124.70) | 3.74 (1.81,7.40) | 3.65 (1.99,6.24) | -0.07 (-0.14,0.01) | 0.074 |
| Guinea-Bissau | 6.90 (2.91,14.74) | 13.67 (7.25,26.05) | 4.41 (1.87,9.39) | 3.88 (2.07,7.36) | -0.44 (-0.55,-0.33) | 0 |
| Guyana | 11.51 (5.83,20.63) | 20.48 (10.58,37.68) | 7.40 (3.75,13.19) | 13.73 (7.11,25.21) | 2.10 (1.48,2.73) | 0 |
| Haiti | 114.26 (31.81,283.68) | 342.45 (112.31,765.66) | 10.48 (2.96,25.82) | 14.00 (4.62,31.20) | 1.16 (0.83,1.49) | 0 |
| Honduras | 161.62 (67.68,325.13) | 256.81 (89.40,586.59) | 20.77 (8.83,41.38) | 13.42 (4.74,30.54) | -1.55 (-1.85,-1.25) | 0 |
| Hungary | 226.95 (132.62,352.23) | 143.13 (77.13,236.56) | 11.21 (6.61,17.34) | 9.01 (4.89,14.81) | -0.70 (-1.14,-0.26) | 0.002 |
| Iceland | 2.28 (1.31,3.97) | 4.28 (2.38,6.72) | 4.24 (2.45,7.41) | 6.58 (3.62,10.37) | 1.63 (1.35,1.91) | 0 |
| India | 23914.62 (12268.89,43288.64) | 44663.60 (25876.90,72855.00) | 14.57 (7.46,26.36) | 14.95 (8.65,24.38) | 0.18 (-0.27,0.63) | 0.433 |
| Indonesia | 1343.20 (678.23,2911.79) | 1984.80 (1085.20,4135.18) | 3.55 (1.80,7.68) | 3.70 (2.02,7.72) | 0.13 (-0.04,0.30) | 0.13 |
| Iran (Islamic Republic of) | 417.23 (258.46,661.33) | 1062.58 (584.00,1563.23) | 4.01 (2.48,6.34) | 5.31 (2.96,7.76) | 0.99 (0.85,1.13) | 0 |
| Iraq | 160.05 (82.32,296.65) | 536.67 (267.32,982.55) | 5.11 (2.67,9.35) | 5.85 (2.93,10.71) | 0.45 (0.29,0.62) | 0 |
| Ireland | 37.36 (19.65,68.19) | 77.37 (35.06,131.60) | 5.58 (2.94,10.14) | 9.37 (4.16,16.11) | 1.78 (1.28,2.29) | 0 |
| Israel | 53.48 (27.61,92.73) | 89.01 (49.66,143.30) | 5.66 (2.91,9.83) | 5.39 (3.01,8.69) | -0.18 (-0.64,0.27) | 0.434 |
| Italy | 471.85 (296.28,754.79) | 676.48 (295.82,997.75) | 4.40 (2.76,7.04) | 7.76 (3.30,11.50) | 1.95 (1.40,2.50) | 0 |
| Jamaica | 14.90 (7.24,27.21) | 45.82 (21.50,83.29) | 3.36 (1.66,6.05) | 7.94 (3.76,14.35) | 3.17 (1.19,5.19) | 0.002 |
| Japan | 2539.22 (1482.01,4385.81) | 1864.41 (1255.05,2723.76) | 11.09 (6.42,19.23) | 10.40 (6.97,15.27) | -0.19 (-0.37,-0.01) | 0.043 |
| Jordan | 110.11 (55.22,205.66) | 392.18 (209.24,655.58) | 14.89 (7.60,27.43) | 14.62 (7.82,24.39) | -0.08 (-0.38,0.23) | 0.615 |
| Kazakhstan | 234.13 (127.02,411.41) | 333.48 (163.02,632.38) | 6.88 (3.74,12.05) | 8.99 (4.40,17.04) | 0.83 (-0.37,2.04) | 0.176 |
| Kenya | 236.24 (100.63,396.67) | 644.60 (314.72,1026.62) | 6.22 (2.65,10.43) | 6.50 (3.16,10.33) | 0.14 (0.03,0.26) | 0.013 |
| Kiribati | 9.41 (3.58,19.88) | 14.73 (5.69,33.21) | 64.53 (24.52,135.62) | 63.01 (24.40,141.63) | -0.15 (-0.32,0.01) | 0.067 |
| Kuwait | 30.27 (18.60,46.70) | 74.70 (45.21,114.22) | 5.48 (3.34,8.54) | 6.17 (3.73,9.49) | 0.42 (-0.71,1.56) | 0.471 |
| Kyrgyzstan | 38.91 (22.31,61.86) | 46.60 (26.90,75.43) | 4.55 (2.62,7.22) | 3.54 (2.04,5.73) | -0.81 (-1.42,-0.18) | 0.011 |
| Lao People's Democratic Republic | 23.69 (7.11,71.60) | 54.52 (17.89,130.30) | 3.29 (1.00,9.84) | 3.46 (1.14,8.25) | 0.19 (0.07,0.30) | 0.002 |
| Latvia | 46.28 (26.65,74.53) | 22.02 (10.84,37.74) | 9.30 (5.36,15.00) | 6.72 (3.34,11.48) | -1.02 (-2.01,-0.01) | 0.047 |
| Lebanon | 25.55 (11.98,51.88) | 60.41 (29.43,109.70) | 4.64 (2.22,9.32) | 5.55 (2.69,10.10) | 0.64 (0.57,0.72) | 0 |
| Lesotho | 20.80 (9.18,44.44) | 31.49 (15.28,62.71) | 6.99 (3.08,14.94) | 6.67 (3.23,13.34) | -0.17 (-0.29,-0.05) | 0.005 |
| Liberia | 7.30 (3.84,12.41) | 22.61 (12.52,37.73) | 2.53 (1.33,4.33) | 2.52 (1.40,4.20) | 0.00 (-0.11,0.11) | 0.976 |
| Libya | 41.33 (17.87,96.05) | 99.07 (47.49,189.02) | 5.04 (2.21,11.59) | 6.01 (2.87,11.51) | 0.57 (0.00,1.15) | 0.051 |
| Lithuania | 53.35 (30.81,85.76) | 27.56 (15.66,43.95) | 7.55 (4.37,12.12) | 6.01 (3.41,9.60) | -0.80 (-1.31,-0.28) | 0.002 |
| Luxembourg | 3.06 (1.82,5.21) | 7.66 (3.74,12.55) | 3.83 (2.26,6.56) | 6.52 (3.14,10.80) | 1.85 (1.25,2.46) | 0 |
| Madagascar | 377.09 (106.22,924.27) | 731.66 (267.17,1615.85) | 17.93 (5.09,43.37) | 14.26 (5.25,31.17) | -0.85 (-1.35,-0.35) | 0.001 |
| Malawi | 90.04 (31.48,202.81) | 208.55 (85.68,434.73) | 5.63 (1.98,12.61) | 6.32 (2.60,13.01) | 0.38 (0.11,0.66) | 0.007 |
| Malaysia | 139.66 (61.18,276.67) | 393.72 (177.23,766.99) | 3.88 (1.70,7.67) | 5.31 (2.38,10.37) | 1.15 (0.58,1.73) | 0 |
| Maldives | 19.26 (7.90,41.63) | 44.28 (21.76,76.28) | 51.73 (21.20,113.49) | 24.82 (12.07,43.13) | -2.43 (-3.04,-1.82) | 0 |
| Mali | 60.58 (26.66,123.59) | 159.97 (81.07,281.18) | 4.82 (2.12,9.83) | 4.76 (2.40,8.34) | -0.03 (-0.17,0.12) | 0.711 |
| Malta | 5.37 (2.99,9.22) | 10.69 (5.64,16.83) | 7.39 (4.11,12.67) | 14.04 (7.29,22.34) | 2.22 (1.69,2.75) | 0 |
| Marshall Islands | 7.22 (3.15,14.16) | 10.19 (4.56,19.89) | 84.38 (37.38,164.87) | 82.98 (37.17,162.02) | -0.07 (-0.14,-0.01) | 0.034 |
| Mauritania | 12.67 (6.29,24.50) | 20.00 (11.22,32.65) | 3.71 (1.85,7.14) | 2.87 (1.61,4.67) | -0.88 (-0.98,-0.78) | 0 |
| Mauritius | 12.65 (5.88,24.95) | 21.95 (10.64,39.41) | 4.91 (2.29,9.66) | 9.40 (4.54,16.93) | 2.32 (1.70,2.95) | 0 |
| Mexico | 1732.96 (1290.19,2488.29) | 4303.86 (2131.34,6265.24) | 11.11 (8.25,15.85) | 17.65 (8.73,25.72) | 1.80 (1.21,2.40) | 0 |
| Micronesia (Federated States of) | 21.26 (8.48,44.62) | 17.80 (6.34,36.41) | 106.94 (42.98,224.59) | 84.32 (29.01,173.63) | -0.81 (-0.88,-0.75) | 0 |
| Monaco | 0.25 (0.13,0.46) | 0.26 (0.13,0.46) | 5.13 (2.61,9.47) | 5.36 (2.69,9.73) | 0.16 (0.12,0.20) | 0 |
| Mongolia | 71.74 (31.58,137.51) | 86.17 (39.38,169.20) | 16.45 (7.26,31.36) | 12.25 (5.58,24.10) | -1.04 (-1.51,-0.56) | 0 |
| Montenegro | 6.32 (3.17,11.05) | 5.68 (2.60,10.47) | 4.82 (2.42,8.42) | 4.95 (2.31,9.04) | -0.04 (-0.65,0.58) | 0.906 |
| Morocco | 202.16 (88.21,434.29) | 345.10 (169.44,647.90) | 4.18 (1.85,8.91) | 4.69 (2.30,8.81) | 0.36 (0.07,0.66) | 0.016 |
| Mozambique | 77.95 (29.96,174.27) | 262.10 (106.34,574.99) | 4.08 (1.58,9.02) | 5.50 (2.24,11.95) | 1.06 (0.82,1.30) | 0 |
| Myanmar | 368.51 (106.07,1202.26) | 496.98 (169.53,1235.81) | 4.42 (1.30,14.31) | 4.65 (1.59,11.56) | 0.12 (0.05,0.20) | 0.001 |
| Namibia | 18.65 (8.12,37.07) | 38.02 (18.30,69.14) | 8.04 (3.48,15.98) | 8.17 (3.92,14.91) | 0.06 (-0.14,0.26) | 0.534 |
| Nauru | 2.14 (0.90,4.43) | 1.98 (0.87,4.04) | 107.98 (45.60,224.01) | 88.07 (38.60,180.31) | -0.70 (-0.74,-0.65) | 0 |
| Nepal | 278.47 (104.74,713.07) | 437.25 (182.86,950.00) | 8.46 (3.19,21.74) | 7.87 (3.29,17.27) | -0.23 (-0.36,-0.09) | 0.001 |
| Netherlands | 88.05 (50.17,151.80) | 122.31 (52.30,211.12) | 2.76 (1.56,4.76) | 4.43 (1.89,7.65) | 1.61 (1.19,2.03) | 0 |
| New Zealand | 30.96 (19.10,50.66) | 45.49 (23.40,73.02) | 4.49 (2.77,7.35) | 6.57 (3.37,10.54) | 1.31 (0.83,1.79) | 0 |
| Nicaragua | 27.56 (15.33,46.75) | 97.11 (44.92,173.19) | 4.39 (2.46,7.41) | 7.03 (3.25,12.52) | 1.62 (1.22,2.02) | 0 |
| Niger | 50.61 (21.93,106.17) | 131.08 (66.44,245.81) | 4.19 (1.82,8.75) | 3.80 (1.94,7.10) | -0.32 (-0.59,-0.05) | 0.021 |
| Nigeria | 469.03 (256.87,752.05) | 1041.84 (644.96,1618.73) | 2.97 (1.64,4.76) | 2.94 (1.83,4.55) | -0.04 (-0.23,0.15) | 0.683 |
| Niue | 0.33 (0.15,0.63) | 0.16 (0.07,0.30) | 80.00 (35.67,153.86) | 54.08 (24.98,103.78) | -1.36 (-1.53,-1.18) | 0 |
| North Macedonia | 20.03 (10.03,35.13) | 23.38 (11.22,42.08) | 4.76 (2.39,8.34) | 5.22 (2.54,9.31) | 0.33 (0.13,0.53) | 0.001 |
| Northern Mariana Islands | 11.76 (5.41,21.58) | 5.60 (3.04,9.54) | 96.12 (44.06,175.88) | 81.95 (44.18,140.84) | -0.60 (-0.73,-0.47) | 0 |
| Norway | 65.18 (43.90,92.85) | 106.72 (60.91,150.41) | 7.73 (5.20,11.00) | 11.21 (6.38,15.80) | 1.39 (0.98,1.80) | 0 |
| Oman | 41.49 (14.95,103.28) | 122.84 (53.54,246.80) | 7.82 (2.79,19.57) | 6.47 (2.74,13.14) | -0.60 (-0.83,-0.38) | 0 |
| Pakistan | 2740.36 (1266.54,5326.13) | 6059.42 (3272.19,11029.16) | 14.80 (6.85,28.61) | 14.55 (7.88,26.48) | -0.05 (-0.25,0.15) | 0.622 |
| Palau | 5.90 (2.64,11.46) | 5.50 (2.63,10.24) | 167.28 (74.96,324.83) | 140.73 (67.54,260.90) | -0.60 (-0.73,-0.48) | 0 |
| Palestine | 42.72 (20.24,85.09) | 121.10 (68.26,196.88) | 12.33 (6.01,24.13) | 12.12 (6.88,19.65) | -0.12 (-0.53,0.30) | 0.577 |
| Panama | 41.50 (21.44,73.05) | 99.03 (52.10,167.90) | 8.30 (4.25,14.76) | 12.23 (6.46,20.69) | 1.38 (0.57,2.20) | 0.001 |
| Papua New Guinea | 467.93 (185.56,977.63) | 1185.48 (521.13,2303.01) | 55.66 (22.16,115.63) | 57.31 (25.27,111.38) | 0.06 (-0.07,0.18) | 0.384 |
| Paraguay | 29.72 (12.28,67.80) | 79.52 (36.94,159.09) | 4.00 (1.68,9.06) | 5.37 (2.50,10.68) | 1.07 (0.58,1.57) | 0 |
| Peru | 1777.79 (815.15,3122.64) | 2484.41 (1291.58,4222.12) | 43.14 (19.93,75.40) | 36.37 (18.92,61.77) | -0.47 (-1.48,0.54) | 0.359 |
| Philippines | 163.96 (105.19,235.67) | 395.93 (251.02,552.63) | 1.33 (0.86,1.91) | 1.72 (1.09,2.40) | 0.87 (0.68,1.06) | 0 |
| Poland | 796.56 (527.04,1111.56) | 703.57 (481.75,966.95) | 10.02 (6.62,13.99) | 9.42 (6.41,12.92) | -0.29 (-0.47,-0.11) | 0.001 |
| Portugal | 119.18 (68.39,222.07) | 158.87 (53.94,290.65) | 6.41 (3.68,11.94) | 9.99 (3.30,18.48) | 1.56 (0.29,2.84) | 0.016 |
| Puerto Rico | 72.34 (38.49,140.62) | 96.41 (52.75,160.47) | 11.02 (5.88,21.33) | 17.26 (9.44,28.73) | 1.50 (0.62,2.38) | 0.001 |
| Qatar | 7.11 (3.97,11.91) | 56.33 (30.85,92.27) | 3.76 (2.06,6.42) | 3.66 (2.00,6.05) | -0.06 (-0.44,0.32) | 0.745 |
| Republic of Korea | 809.15 (398.20,1702.78) | 646.86 (392.37,992.33) | 7.60 (3.75,15.92) | 6.54 (3.95,10.06) | -0.56 (-0.70,-0.41) | 0 |
| Republic of Moldova | 54.09 (31.41,87.04) | 42.39 (20.81,75.25) | 5.93 (3.45,9.53) | 5.47 (2.72,9.68) | -0.25 (-0.90,0.39) | 0.441 |
| Romania | 1166.48 (635.00,1898.87) | 447.98 (265.68,690.74) | 25.90 (14.04,42.30) | 14.24 (8.45,21.97) | -2.07 (-2.57,-1.57) | 0 |
| Russian Federation | 1971.54 (1243.99,2924.89) | 1592.34 (966.03,2493.93) | 6.20 (3.92,9.20) | 5.67 (3.45,8.87) | -0.21 (-0.87,0.44) | 0.522 |
| Rwanda | 127.81 (34.71,349.18) | 202.95 (80.48,435.02) | 10.05 (2.73,27.39) | 8.00 (3.17,17.07) | -0.77 (-1.04,-0.50) | 0 |
| Saint Kitts and Nevis | 0.46 (0.24,0.85) | 0.85 (0.27,1.71) | 5.79 (3.04,10.75) | 7.32 (2.43,14.62) | 1.17 (0.08,2.26) | 0.035 |
| Saint Lucia | 2.43 (1.33,4.15) | 7.27 (3.83,12.55) | 9.99 (5.51,17.01) | 21.16 (11.15,36.55) | 2.60 (1.87,3.34) | 0 |
| Saint Vincent and the Grenadines | 0.74 (0.29,1.69) | 1.85 (1.00,3.15) | 3.57 (1.43,8.20) | 8.71 (4.71,14.88) | 3.16 (2.14,4.18) | 0 |
| Samoa | 26.73 (11.09,51.58) | 25.64 (10.47,49.00) | 77.08 (32.33,148.09) | 62.44 (25.33,119.46) | -0.73 (-0.79,-0.66) | 0 |
| San Marino | 0.10 (0.05,0.17) | 0.15 (0.06,0.31) | 2.15 (1.17,3.68) | 2.92 (1.23,6.05) | 1.08 (0.94,1.22) | 0 |
| Sao Tome and Principe | 1.08 (0.47,2.22) | 2.41 (1.27,4.37) | 6.40 (2.82,13.08) | 5.85 (3.09,10.65) | -0.31 (-0.64,0.02) | 0.065 |
| Saudi Arabia | 290.30 (128.30,610.76) | 1410.66 (694.84,2700.07) | 7.69 (3.42,16.11) | 11.41 (5.57,21.91) | 1.41 (1.24,1.59) | 0 |
| Senegal | 45.03 (20.92,89.35) | 98.90 (55.60,164.35) | 3.84 (1.79,7.58) | 3.60 (2.03,5.97) | -0.28 (-0.96,0.40) | 0.422 |
| Serbia | 124.55 (69.91,206.23) | 98.92 (55.24,158.86) | 6.76 (3.79,11.22) | 6.31 (3.52,10.13) | -0.22 (-0.45,0.01) | 0.063 |
| Seychelles | 0.37 (0.15,0.83) | 0.59 (0.24,1.28) | 2.41 (0.97,5.37) | 2.61 (1.05,5.67) | 0.28 (-0.17,0.74) | 0.227 |
| Sierra Leone | 20.29 (9.97,38.90) | 52.34 (28.74,91.53) | 3.38 (1.67,6.45) | 3.36 (1.85,5.86) | -0.01 (-0.21,0.19) | 0.952 |
| Singapore | 131.86 (57.04,229.46) | 154.29 (64.26,277.22) | 16.66 (7.23,29.02) | 12.55 (5.35,22.15) | -0.85 (-1.38,-0.33) | 0.002 |
| Slovakia | 81.51 (44.82,135.00) | 89.55 (44.78,151.87) | 7.44 (4.11,12.30) | 8.67 (4.29,14.78) | 0.52 (0.27,0.76) | 0 |
| Slovenia | 26.06 (12.68,46.44) | 26.11 (12.44,47.11) | 6.21 (3.05,11.05) | 7.25 (3.54,12.89) | 0.62 (0.30,0.93) | 0 |
| Solomon Islands | 35.97 (12.31,92.15) | 86.05 (33.94,198.02) | 56.60 (19.23,148.49) | 67.19 (26.54,155.20) | 0.62 (0.39,0.85) | 0 |
| Somalia | 94.24 (25.94,265.05) | 248.63 (81.66,696.26) | 7.83 (2.16,22.01) | 6.69 (2.21,18.65) | -0.52 (-0.66,-0.37) | 0 |
| South Africa | 1140.00 (696.82,1607.36) | 1456.43 (946.38,2347.02) | 16.40 (9.97,23.13) | 11.46 (7.45,18.45) | -1.33 (-2.22,-0.43) | 0.004 |
| South Sudan | 77.26 (25.82,184.08) | 82.90 (35.73,177.85) | 7.02 (2.36,16.54) | 5.85 (2.56,12.31) | -0.56 (-0.93,-0.20) | 0.002 |
| Spain | 711.65 (430.91,1199.40) | 687.54 (318.41,1057.15) | 9.67 (5.88,16.29) | 9.44 (4.24,14.67) | -0.22 (-0.70,0.26) | 0.372 |
| Sri Lanka | 120.59 (54.12,250.54) | 107.27 (49.52,205.63) | 3.31 (1.50,6.85) | 2.71 (1.25,5.21) | -0.64 (-1.77,0.50) | 0.269 |
| Sudan | 219.10 (75.88,543.23) | 596.51 (243.86,1207.62) | 6.53 (2.30,16.17) | 7.58 (3.15,15.18) | 0.55 (0.44,0.66) | 0 |
| Suriname | 6.20 (2.96,11.20) | 16.21 (7.59,31.27) | 8.31 (4.01,15.03) | 15.19 (7.12,29.29) | 2.03 (0.60,3.49) | 0.005 |
| Sweden | 67.93 (42.65,110.35) | 116.85 (72.09,166.12) | 4.33 (2.71,7.06) | 6.77 (4.13,9.68) | 1.51 (1.15,1.87) | 0 |
| Switzerland | 99.63 (50.76,190.95) | 89.15 (50.39,138.94) | 6.92 (3.52,13.36) | 5.88 (3.28,9.19) | -0.66 (-1.32,0.01) | 0.054 |
| Syrian Arab Republic | 81.59 (42.39,143.93) | 106.34 (54.12,189.12) | 3.64 (1.93,6.32) | 4.56 (2.40,7.95) | 0.77 (0.56,0.98) | 0 |
| Taiwan (Province of China) | 116.61 (65.77,217.72) | 238.81 (100.91,422.36) | 2.44 (1.37,4.56) | 5.48 (2.30,9.72) | 2.87 (2.51,3.23) | 0 |
| Tajikistan | 225.93 (102.85,428.81) | 477.05 (178.79,934.91) | 25.14 (11.54,47.32) | 24.39 (9.12,47.67) | -0.11 (-0.71,0.49) | 0.717 |
| Thailand | 326.30 (142.24,683.06) | 581.57 (207.99,1245.35) | 2.54 (1.12,5.27) | 4.65 (1.66,10.04) | 2.16 (1.67,2.65) | 0 |
| Timor-Leste | 3.91 (1.16,9.56) | 7.43 (2.11,18.49) | 2.46 (0.75,5.96) | 2.91 (0.82,7.23) | 0.54 (-0.56,1.64) | 0.339 |
| Togo | 22.98 (11.24,44.57) | 52.56 (29.26,89.82) | 3.99 (1.96,7.68) | 3.74 (2.09,6.36) | -0.24 (-0.46,-0.01) | 0.04 |
| Tokelau | 0.19 (0.08,0.37) | 0.13 (0.06,0.24) | 67.74 (28.44,129.26) | 52.21 (22.59,96.96) | -0.89 (-0.92,-0.87) | 0 |
| Tonga | 7.91 (3.20,15.01) | 9.44 (4.11,17.98) | 46.72 (19.21,87.59) | 51.89 (22.71,98.28) | 0.34 (0.23,0.46) | 0 |
| Trinidad and Tobago | 28.11 (14.39,50.88) | 42.70 (20.02,81.06) | 11.65 (6.03,20.88) | 14.68 (6.84,28.24) | 0.75 (0.34,1.17) | 0 |
| Tunisia | 67.18 (30.44,132.14) | 124.58 (62.23,224.30) | 4.15 (1.92,8.09) | 5.38 (2.67,9.75) | 0.91 (0.78,1.03) | 0 |
| Turkey | 1586.64 (737.26,3178.47) | 1749.68 (916.73,3004.00) | 13.00 (6.04,26.14) | 9.61 (5.04,16.53) | -1.13 (-1.67,-0.59) | 0 |
| Turkmenistan | 62.03 (31.98,111.07) | 72.45 (39.76,126.81) | 8.85 (4.57,15.84) | 6.56 (3.59,11.49) | -1.04 (-1.62,-0.45) | 0.001 |
| Tuvalu | 1.53 (0.65,3.03) | 1.89 (0.93,3.35) | 91.28 (38.80,181.07) | 75.58 (37.38,134.06) | -0.65 (-0.72,-0.58) | 0 |
| Uganda | 244.30 (72.02,638.59) | 603.37 (241.38,1383.43) | 8.84 (2.63,22.81) | 8.79 (3.52,20.15) | -0.09 (-0.49,0.31) | 0.658 |
| Ukraine | 1379.98 (768.57,2209.33) | 934.44 (555.78,1515.28) | 13.86 (7.73,22.17) | 11.15 (6.58,18.15) | -0.73 (-2.16,0.73) | 0.326 |
| United Arab Emirates | 133.38 (46.12,331.16) | 1037.37 (395.73,2360.57) | 17.18 (5.83,43.04) | 22.55 (8.28,53.26) | 0.88 (0.62,1.14) | 0 |
| United Kingdom | 692.94 (482.13,1200.95) | 1326.84 (781.49,1648.46) | 6.55 (4.55,11.36) | 11.16 (6.53,13.88) | 1.84 (1.50,2.18) | 0 |
| United Republic of Tanzania | 223.99 (82.62,493.24) | 548.42 (235.30,1122.42) | 5.46 (2.02,11.92) | 5.73 (2.46,11.68) | 0.18 (0.04,0.32) | 0.012 |
| United States of America | 9281.06 (6914.88,11624.51) | 7478.23 (5791.99,9913.73) | 17.02 (12.68,21.36) | 12.98 (10.04,17.22) | -0.93 (-1.23,-0.63) | 0 |
| United States Virgin Islands | 2.57 (1.21,4.77) | 2.05 (1.02,3.94) | 13.83 (6.55,25.65) | 13.64 (6.75,26.22) | 0.20 (-0.51,0.92) | 0.576 |
| Uruguay | 28.03 (14.69,51.64) | 56.41 (24.96,101.76) | 5.05 (2.65,9.31) | 9.26 (4.12,16.68) | 2.42 (1.83,3.00) | 0 |
| Uzbekistan | 753.97 (338.83,1299.90) | 1111.09 (546.70,1780.74) | 18.77 (8.40,32.33) | 15.50 (7.60,24.84) | -0.69 (-1.26,-0.11) | 0.019 |
| Vanuatu | 26.19 (9.50,55.17) | 53.48 (21.27,106.85) | 93.38 (34.13,197.21) | 95.37 (38.11,190.59) | 0.07 (-0.13,0.26) | 0.516 |
| Venezuela (Bolivarian Republic of) | 254.65 (139.70,431.00) | 432.96 (243.97,724.06) | 6.68 (3.68,11.26) | 8.26 (4.65,13.84) | 0.89 (0.45,1.35) | 0 |
| Viet Nam | 313.85 (118.07,738.97) | 451.50 (178.71,984.79) | 2.33 (0.89,5.42) | 2.21 (0.87,4.84) | -0.17 (-0.29,-0.04) | 0.009 |
| Yemen | 96.66 (35.94,274.88) | 343.00 (162.91,708.80) | 4.72 (1.77,13.40) | 5.53 (2.64,11.40) | 0.59 (0.42,0.77) | 0 |
| Zambia | 77.47 (26.26,177.17) | 238.44 (100.63,481.60) | 6.08 (2.06,13.68) | 6.99 (2.95,14.06) | 0.49 (0.31,0.68) | 0 |
| Zimbabwe | 76.35 (34.99,137.96) | 136.75 (62.17,258.32) | 4.80 (2.16,8.70) | 5.13 (2.31,9.71) | 0.26 (0.13,0.39) | 0 |

NOTE:APPC,average annual percentage change;

| **Table 12.ASMR of interstitial lung disease and pulmonary sarcoidosis in adolescents and young adultsr in 1990 and 2019 for male and all nation, with AAPC from 2009 and 2019** | | | | | | |
| --- | --- | --- | --- | --- | --- | --- |
| location | Numbers in 1990 | Numbers in 2019 | Age-standardized rates in 1990   (95% CI) | Age standardized rates in 2019   (95% CI) | AAPC, %   (95% CI) | P |
| Afghanistan | 1.00 (0.19,3.70) | 4.29 (1.16,11.62) | 0.07 (0.01,0.25) | 0.06 (0.02,0.17) | -0.21 (-0.56,0.13) | 0.225 |
| Albania | 0.66 (0.30,1.23) | 0.33 (0.15,0.64) | 0.10 (0.04,0.18) | 0.07 (0.03,0.13) | -1.21 (-2.14,-0.28) | 0.011 |
| Algeria | 2.80 (0.75,8.55) | 5.97 (2.04,12.94) | 0.06 (0.02,0.18) | 0.07 (0.02,0.14) | 0.33 (0.05,0.62) | 0.023 |
| American Samoa | 0.06 (0.03,0.12) | 0.06 (0.02,0.11) | 0.62 (0.28,1.20) | 0.56 (0.25,1.11) | -0.33 (-0.57,-0.08) | 0.009 |
| Andorra | 0.02 (0.01,0.03) | 0.03 (0.01,0.06) | 0.11 (0.04,0.23) | 0.18 (0.06,0.37) | 1.52 (1.37,1.67) | 0 |
| Angola | 1.58 (0.33,4.50) | 3.37 (1.09,7.69) | 0.09 (0.02,0.24) | 0.07 (0.02,0.16) | -0.72 (-1.67,0.24) | 0.143 |
| Antigua and Barbuda | 0.00 (0.00,0.00) | 0.01 (0.00,0.01) | 0.02 (0.01,0.03) | 0.04 (0.02,0.06) | 2.77 (0.94,4.62) | 0.003 |
| Argentina | 10.25 (5.49,16.85) | 17.11 (7.95,29.66) | 0.17 (0.09,0.28) | 0.20 (0.09,0.34) | 0.46 (-0.02,0.94) | 0.059 |
| Armenia | 0.98 (0.40,1.92) | 0.58 (0.26,1.06) | 0.14 (0.06,0.27) | 0.09 (0.04,0.17) | -1.36 (-2.34,-0.36) | 0.008 |
| Australia | 1.79 (0.90,3.41) | 5.07 (2.04,9.07) | 0.05 (0.03,0.10) | 0.11 (0.05,0.21) | 2.80 (1.78,3.84) | 0 |
| Austria | 0.77 (0.40,1.34) | 0.99 (0.44,1.76) | 0.05 (0.03,0.09) | 0.06 (0.03,0.12) | 0.76 (0.50,1.03) | 0 |
| Azerbaijan | 1.12 (0.47,2.24) | 1.32 (0.41,2.97) | 0.08 (0.03,0.16) | 0.06 (0.02,0.13) | -1.17 (-1.61,-0.73) | 0 |
| Bahamas | 0.06 (0.03,0.11) | 0.17 (0.08,0.32) | 0.11 (0.06,0.20) | 0.24 (0.11,0.43) | 2.63 (2.13,3.13) | 0 |
| Bahrain | 0.14 (0.05,0.32) | 0.43 (0.20,0.83) | 0.08 (0.03,0.19) | 0.11 (0.05,0.20) | 0.90 (0.14,1.66) | 0.02 |
| Bangladesh | 56.76 (19.10,122.41) | 66.22 (24.19,127.65) | 0.29 (0.10,0.63) | 0.21 (0.08,0.40) | -1.18 (-1.81,-0.55) | 0 |
| Barbados | 0.03 (0.02,0.06) | 0.05 (0.03,0.09) | 0.06 (0.03,0.11) | 0.11 (0.06,0.19) | 2.03 (1.38,2.69) | 0 |
| Belarus | 2.07 (0.79,3.93) | 0.82 (0.25,1.76) | 0.10 (0.04,0.19) | 0.04 (0.01,0.09) | -2.84 (-3.73,-1.94) | 0 |
| Belgium | 1.41 (0.78,2.50) | 1.61 (0.75,2.73) | 0.07 (0.04,0.13) | 0.09 (0.04,0.14) | 0.58 (0.09,1.07) | 0.021 |
| Belize | 0.14 (0.07,0.24) | 1.08 (0.54,1.78) | 0.41 (0.19,0.71) | 1.29 (0.66,2.14) | 4.16 (3.62,4.70) | 0 |
| Benin | 0.33 (0.10,0.77) | 0.74 (0.30,1.52) | 0.05 (0.01,0.11) | 0.04 (0.01,0.07) | -0.98 (-1.15,-0.80) | 0 |
| Bermuda | 0.05 (0.02,0.08) | 0.04 (0.02,0.07) | 0.34 (0.17,0.58) | 0.43 (0.23,0.67) | 0.88 (0.60,1.17) | 0 |
| Bhutan | 0.19 (0.06,0.41) | 0.21 (0.07,0.47) | 0.16 (0.05,0.36) | 0.11 (0.04,0.25) | -1.22 (-1.31,-1.13) | 0 |
| Bolivia (Plurinational State of) | 4.66 (1.68,9.51) | 8.45 (2.97,16.52) | 0.42 (0.15,0.85) | 0.36 (0.13,0.70) | -0.54 (-0.63,-0.45) | 0 |
| Bosnia and Herzegovina | 0.68 (0.23,1.49) | 0.25 (0.11,0.49) | 0.07 (0.02,0.15) | 0.04 (0.02,0.08) | -1.59 (-2.21,-0.96) | 0 |
| Botswana | 0.30 (0.08,0.72) | 0.61 (0.21,1.35) | 0.15 (0.04,0.36) | 0.11 (0.04,0.25) | -0.95 (-1.18,-0.72) | 0 |
| Brazil | 53.71 (35.31,72.73) | 77.98 (47.90,101.90) | 0.19 (0.12,0.25) | 0.18 (0.11,0.23) | -0.23 (-0.62,0.16) | 0.253 |
| Brunei Darussalam | 0.22 (0.09,0.47) | 0.25 (0.13,0.44) | 0.33 (0.13,0.70) | 0.22 (0.11,0.38) | -1.45 (-2.00,-0.90) | 0 |
| Bulgaria | 0.81 (0.43,1.41) | 0.45 (0.22,0.85) | 0.05 (0.03,0.09) | 0.04 (0.02,0.07) | -0.85 (-1.24,-0.47) | 0 |
| Burkina Faso | 0.20 (0.06,0.50) | 0.63 (0.23,1.35) | 0.02 (0.00,0.04) | 0.02 (0.01,0.04) | 0.22 (-0.17,0.60) | 0.271 |
| Burundi | 1.30 (0.23,3.73) | 2.27 (0.67,5.55) | 0.14 (0.02,0.39) | 0.11 (0.03,0.26) | -0.86 (-1.25,-0.47) | 0 |
| Cabo Verde | 0.03 (0.01,0.07) | 0.02 (0.01,0.05) | 0.07 (0.03,0.14) | 0.02 (0.01,0.04) | -4.56 (-5.11,-4.01) | 0 |
| Cambodia | 0.52 (0.14,1.51) | 1.06 (0.32,2.56) | 0.03 (0.01,0.09) | 0.03 (0.01,0.07) | -0.08 (-0.18,0.03) | 0.137 |
| Cameroon | 0.94 (0.33,2.15) | 2.50 (1.02,5.22) | 0.06 (0.02,0.13) | 0.05 (0.02,0.10) | -0.75 (-1.08,-0.42) | 0 |
| Canada | 7.52 (4.01,13.51) | 8.80 (5.24,13.39) | 0.12 (0.07,0.23) | 0.14 (0.08,0.22) | 0.49 (0.10,0.89) | 0.014 |
| Central African Republic | 0.52 (0.08,1.81) | 0.91 (0.18,3.23) | 0.11 (0.02,0.39) | 0.10 (0.02,0.35) | -0.44 (-0.64,-0.23) | 0 |
| Chad | 0.38 (0.11,0.96) | 0.93 (0.35,2.06) | 0.04 (0.01,0.11) | 0.04 (0.02,0.09) | -0.21 (-0.43,0.01) | 0.062 |
| Chile | 4.76 (2.46,9.61) | 6.48 (3.85,9.98) | 0.18 (0.09,0.36) | 0.18 (0.11,0.28) | 0.07 (-0.39,0.53) | 0.768 |
| China | 113.39 (71.24,183.88) | 99.83 (65.32,141.42) | 0.04 (0.03,0.07) | 0.04 (0.02,0.05) | -0.50 (-0.85,-0.14) | 0.006 |
| Colombia | 3.34 (1.48,7.77) | 16.56 (4.47,34.79) | 0.05 (0.02,0.12) | 0.17 (0.05,0.37) | 4.34 (3.71,4.98) | 0 |
| Comoros | 0.07 (0.01,0.22) | 0.12 (0.03,0.29) | 0.10 (0.01,0.29) | 0.09 (0.02,0.20) | -0.52 (-3.00,2.02) | 0.684 |
| Congo | 0.54 (0.10,1.81) | 0.86 (0.28,1.99) | 0.13 (0.02,0.45) | 0.09 (0.03,0.20) | -1.52 (-2.20,-0.83) | 0 |
| Cook Islands | 0.02 (0.01,0.05) | 0.01 (0.00,0.02) | 0.64 (0.28,1.22) | 0.41 (0.17,0.77) | -1.52 (-1.68,-1.37) | 0 |
| Costa Rica | 0.96 (0.52,1.58) | 2.63 (1.42,4.39) | 0.16 (0.09,0.26) | 0.27 (0.15,0.46) | 2.19 (1.56,2.82) | 0 |
| Coted'Ivoire | 1.00 (0.30,2.46) | 1.66 (0.66,3.46) | 0.04 (0.01,0.11) | 0.03 (0.01,0.07) | -1.13 (-1.30,-0.96) | 0 |
| Croatia | 0.49 (0.24,0.84) | 0.37 (0.13,0.67) | 0.05 (0.02,0.08) | 0.05 (0.02,0.09) | -0.15 (-1.22,0.94) | 0.791 |
| Cuba | 1.44 (0.63,2.86) | 1.31 (0.55,2.48) | 0.06 (0.03,0.12) | 0.07 (0.03,0.13) | 0.32 (-0.34,1.00) | 0.343 |
| Cyprus | 0.12 (0.04,0.34) | 0.19 (0.05,0.48) | 0.08 (0.02,0.21) | 0.07 (0.02,0.17) | -0.42 (-0.74,-0.09) | 0.011 |
| Czechia | 1.08 (0.51,2.26) | 1.63 (0.52,3.09) | 0.05 (0.02,0.11) | 0.09 (0.03,0.17) | 1.91 (1.23,2.59) | 0 |
| Democratic People's Republic of Korea | 2.42 (0.81,5.89) | 3.03 (1.08,6.70) | 0.06 (0.02,0.15) | 0.05 (0.02,0.12) | -0.55 (-0.61,-0.49) | 0 |
| Democratic Republic of the Congo | 6.35 (1.48,17.02) | 16.15 (5.00,40.40) | 0.10 (0.02,0.27) | 0.10 (0.03,0.25) | -0.04 (-0.24,0.16) | 0.721 |
| Denmark | 1.44 (0.70,2.52) | 1.43 (0.71,2.38) | 0.14 (0.07,0.25) | 0.15 (0.08,0.25) | 0.17 (-0.51,0.85) | 0.619 |
| Djibouti | 0.10 (0.02,0.27) | 0.20 (0.05,0.51) | 0.10 (0.02,0.27) | 0.08 (0.02,0.20) | -0.98 (-1.25,-0.70) | 0 |
| Dominica | 0.01 (0.01,0.02) | 0.02 (0.01,0.04) | 0.09 (0.04,0.18) | 0.16 (0.07,0.31) | 1.85 (1.61,2.09) | 0 |
| Dominican Republic | 0.68 (0.27,1.32) | 1.69 (0.65,3.76) | 0.05 (0.02,0.10) | 0.08 (0.03,0.17) | 1.46 (0.88,2.05) | 0 |
| Ecuador | 4.85 (2.26,8.99) | 14.08 (6.69,24.27) | 0.26 (0.12,0.48) | 0.40 (0.19,0.69) | 1.31 (0.61,2.01) | 0 |
| Egypt | 9.09 (2.06,25.94) | 21.41 (6.38,48.85) | 0.09 (0.02,0.25) | 0.10 (0.03,0.23) | 0.67 (0.49,0.84) | 0 |
| El Salvador | 1.42 (0.63,2.88) | 1.80 (0.79,3.48) | 0.16 (0.07,0.32) | 0.16 (0.07,0.31) | 0.04 (-0.91,1.00) | 0.936 |
| Equatorial Guinea | 0.07 (0.01,0.25) | 0.29 (0.09,0.67) | 0.11 (0.02,0.41) | 0.09 (0.03,0.22) | -0.70 (-1.12,-0.29) | 0.001 |
| Eritrea | 0.61 (0.10,1.98) | 1.53 (0.39,3.94) | 0.11 (0.02,0.37) | 0.11 (0.03,0.29) | 0.00 (-0.24,0.24) | 0.997 |
| Estonia | 0.54 (0.24,0.95) | 0.33 (0.14,0.57) | 0.18 (0.08,0.31) | 0.13 (0.06,0.23) | -1.34 (-2.96,0.31) | 0.111 |
| Eswatini | 0.12 (0.04,0.29) | 0.26 (0.09,0.59) | 0.11 (0.04,0.26) | 0.12 (0.04,0.26) | 0.08 (-0.08,0.24) | 0.323 |
| Ethiopia | 5.91 (1.16,14.03) | 12.17 (4.30,23.43) | 0.07 (0.01,0.18) | 0.06 (0.02,0.12) | -0.54 (-0.75,-0.32) | 0 |
| Fiji | 0.56 (0.24,1.07) | 0.61 (0.28,1.12) | 0.35 (0.15,0.67) | 0.33 (0.15,0.61) | -0.25 (-0.47,-0.03) | 0.027 |
| Finland | 0.81 (0.33,1.94) | 0.61 (0.31,1.09) | 0.08 (0.03,0.19) | 0.06 (0.03,0.12) | -0.69 (-1.02,-0.36) | 0 |
| France | 8.31 (5.13,14.68) | 7.26 (3.86,11.79) | 0.07 (0.04,0.13) | 0.07 (0.04,0.11) | -0.16 (-0.86,0.54) | 0.658 |
| Gabon | 0.20 (0.04,0.54) | 0.28 (0.09,0.67) | 0.11 (0.02,0.30) | 0.08 (0.03,0.20) | -1.11 (-1.42,-0.81) | 0 |
| Gambia | 0.07 (0.02,0.17) | 0.16 (0.06,0.32) | 0.04 (0.01,0.10) | 0.04 (0.02,0.08) | -0.12 (-1.27,1.03) | 0.831 |
| Georgia | 1.08 (0.55,1.88) | 1.18 (0.53,2.14) | 0.10 (0.05,0.18) | 0.18 (0.08,0.33) | 1.98 (0.20,3.78) | 0.029 |
| Germany | 15.84 (8.36,28.94) | 13.47 (7.64,21.05) | 0.10 (0.05,0.18) | 0.09 (0.05,0.15) | -0.32 (-0.92,0.27) | 0.284 |
| Ghana | 1.55 (0.52,3.39) | 4.02 (1.39,8.44) | 0.06 (0.02,0.14) | 0.07 (0.02,0.14) | 0.15 (-0.36,0.66) | 0.57 |
| Greece | 0.57 (0.29,1.13) | 1.55 (0.53,3.03) | 0.03 (0.01,0.06) | 0.09 (0.03,0.18) | 3.84 (2.76,4.93) | 0 |
| Greenland | 0.02 (0.01,0.06) | 0.02 (0.01,0.04) | 0.14 (0.05,0.37) | 0.17 (0.07,0.36) | 0.70 (0.13,1.27) | 0.016 |
| Grenada | 0.01 (0.01,0.03) | 0.03 (0.01,0.05) | 0.10 (0.05,0.17) | 0.13 (0.06,0.24) | 0.94 (-0.06,1.95) | 0.066 |
| Guam | 0.64 (0.36,1.04) | 0.63 (0.35,1.01) | 1.90 (1.07,3.09) | 2.06 (1.15,3.32) | 0.25 (-0.23,0.73) | 0.304 |
| Guatemala | 4.88 (2.46,9.82) | 8.93 (4.68,15.25) | 0.38 (0.19,0.76) | 0.25 (0.13,0.42) | -1.41 (-2.68,-0.12) | 0.032 |
| Guinea | 0.36 (0.12,0.89) | 0.73 (0.29,1.52) | 0.04 (0.01,0.10) | 0.04 (0.01,0.08) | -0.33 (-0.42,-0.24) | 0 |
| Guinea-Bissau | 0.08 (0.02,0.20) | 0.15 (0.06,0.34) | 0.05 (0.01,0.13) | 0.04 (0.02,0.10) | -0.78 (-0.99,-0.56) | 0 |
| Guyana | 0.17 (0.08,0.33) | 0.33 (0.16,0.61) | 0.11 (0.05,0.21) | 0.22 (0.11,0.42) | 2.26 (1.60,2.91) | 0 |
| Haiti | 1.80 (0.42,4.64) | 5.52 (1.60,12.68) | 0.17 (0.04,0.43) | 0.23 (0.07,0.52) | 1.23 (0.88,1.57) | 0 |
| Honduras | 2.55 (1.01,5.22) | 3.95 (1.23,9.43) | 0.33 (0.13,0.68) | 0.21 (0.07,0.50) | -1.65 (-1.97,-1.32) | 0 |
| Hungary | 2.94 (1.57,4.98) | 1.46 (0.70,2.63) | 0.14 (0.08,0.24) | 0.09 (0.04,0.17) | -1.48 (-2.16,-0.80) | 0 |
| Iceland | 0.03 (0.01,0.06) | 0.06 (0.03,0.10) | 0.05 (0.03,0.11) | 0.09 (0.05,0.15) | 2.03 (1.43,2.64) | 0 |
| India | 369.89 (164.25,710.19) | 687.42 (361.47,1181.42) | 0.23 (0.10,0.44) | 0.23 (0.12,0.40) | 0.16 (-0.36,0.68) | 0.543 |
| Indonesia | 19.20 (8.76,44.89) | 28.40 (14.24,63.77) | 0.05 (0.02,0.12) | 0.05 (0.03,0.12) | 0.10 (-0.10,0.30) | 0.322 |
| Iran (Islamic Republic of) | 3.58 (1.90,6.70) | 10.65 (4.30,15.83) | 0.04 (0.02,0.07) | 0.05 (0.02,0.08) | 1.39 (1.21,1.57) | 0 |
| Iraq | 1.77 (0.66,3.87) | 6.01 (2.14,13.12) | 0.06 (0.02,0.12) | 0.07 (0.02,0.14) | 0.50 (0.22,0.78) | 0.001 |
| Ireland | 0.56 (0.27,1.08) | 1.22 (0.50,2.12) | 0.08 (0.04,0.16) | 0.15 (0.06,0.26) | 1.84 (1.17,2.51) | 0 |
| Israel | 0.80 (0.38,1.46) | 1.34 (0.70,2.21) | 0.09 (0.04,0.16) | 0.08 (0.04,0.13) | -0.21 (-0.76,0.35) | 0.462 |
| Italy | 4.33 (2.82,8.19) | 8.48 (2.51,12.72) | 0.04 (0.03,0.08) | 0.10 (0.03,0.15) | 3.04 (2.25,3.84) | 0 |
| Jamaica | 0.19 (0.08,0.38) | 0.70 (0.29,1.32) | 0.04 (0.02,0.09) | 0.12 (0.05,0.23) | 3.76 (1.49,6.07) | 0.001 |
| Japan | 27.14 (16.07,54.10) | 19.38 (13.79,30.00) | 0.12 (0.07,0.24) | 0.11 (0.08,0.17) | -0.27 (-0.55,0.01) | 0.06 |
| Jordan | 1.59 (0.71,3.13) | 5.70 (2.70,10.08) | 0.22 (0.10,0.43) | 0.21 (0.10,0.38) | -0.14 (-0.49,0.21) | 0.437 |
| Kazakhstan | 3.11 (1.42,6.08) | 4.76 (1.85,9.72) | 0.09 (0.04,0.18) | 0.13 (0.05,0.26) | 1.01 (-0.42,2.45) | 0.168 |
| Kenya | 3.10 (0.97,5.67) | 8.57 (3.24,14.76) | 0.08 (0.03,0.15) | 0.09 (0.03,0.15) | 0.13 (-0.03,0.30) | 0.114 |
| Kiribati | 0.14 (0.05,0.31) | 0.23 (0.08,0.53) | 0.99 (0.33,2.15) | 0.98 (0.34,2.27) | -0.07 (-0.24,0.10) | 0.417 |
| Kuwait | 0.34 (0.18,0.57) | 0.84 (0.45,1.42) | 0.06 (0.03,0.10) | 0.07 (0.04,0.12) | 0.45 (-1.20,2.11) | 0.597 |
| Kyrgyzstan | 0.44 (0.21,0.78) | 0.42 (0.19,0.82) | 0.05 (0.03,0.09) | 0.03 (0.01,0.06) | -1.58 (-2.50,-0.64) | 0.001 |
| Lao People's Democratic Republic | 0.34 (0.08,1.12) | 0.80 (0.21,2.07) | 0.05 (0.01,0.16) | 0.05 (0.01,0.13) | 0.18 (0.05,0.30) | 0.006 |
| Latvia | 0.50 (0.26,0.89) | 0.15 (0.06,0.31) | 0.10 (0.05,0.18) | 0.05 (0.02,0.09) | -2.66 (-3.77,-1.54) | 0 |
| Lebanon | 0.27 (0.08,0.70) | 0.66 (0.22,1.44) | 0.05 (0.01,0.13) | 0.06 (0.02,0.13) | 0.73 (0.58,0.87) | 0 |
| Lesotho | 0.25 (0.07,0.65) | 0.37 (0.12,0.91) | 0.09 (0.03,0.23) | 0.08 (0.03,0.20) | -0.37 (-0.63,-0.11) | 0.006 |
| Liberia | 0.06 (0.02,0.13) | 0.16 (0.06,0.32) | 0.02 (0.01,0.05) | 0.02 (0.01,0.04) | -0.53 (-0.85,-0.22) | 0.001 |
| Libya | 0.45 (0.12,1.35) | 1.17 (0.39,2.65) | 0.06 (0.01,0.17) | 0.07 (0.02,0.16) | 0.76 (-0.09,1.61) | 0.08 |
| Lithuania | 0.57 (0.27,1.03) | 0.24 (0.10,0.43) | 0.08 (0.04,0.15) | 0.05 (0.02,0.09) | -1.70 (-2.72,-0.68) | 0.001 |
| Luxembourg | 0.04 (0.02,0.08) | 0.12 (0.05,0.20) | 0.05 (0.03,0.10) | 0.10 (0.04,0.17) | 2.12 (1.30,2.95) | 0 |
| Madagascar | 5.80 (1.37,14.81) | 11.05 (3.44,25.38) | 0.28 (0.07,0.71) | 0.22 (0.07,0.50) | -0.92 (-1.44,-0.40) | 0.001 |
| Malawi | 1.19 (0.26,3.02) | 2.80 (0.82,6.44) | 0.08 (0.02,0.19) | 0.09 (0.03,0.20) | 0.43 (0.13,0.74) | 0.005 |
| Malaysia | 2.12 (0.84,4.43) | 6.23 (2.52,12.71) | 0.06 (0.02,0.12) | 0.08 (0.03,0.17) | 1.27 (0.64,1.92) | 0 |
| Maldives | 0.31 (0.12,0.68) | 0.74 (0.35,1.29) | 0.85 (0.33,1.91) | 0.40 (0.19,0.71) | -2.46 (-3.10,-1.82) | 0 |
| Mali | 0.73 (0.22,1.77) | 1.84 (0.69,3.80) | 0.06 (0.02,0.14) | 0.06 (0.02,0.12) | -0.18 (-0.38,0.03) | 0.093 |
| Malta | 0.08 (0.04,0.15) | 0.17 (0.08,0.27) | 0.11 (0.06,0.20) | 0.22 (0.11,0.36) | 2.31 (1.66,2.97) | 0 |
| Marshall Islands | 0.11 (0.05,0.22) | 0.16 (0.06,0.32) | 1.30 (0.54,2.60) | 1.30 (0.53,2.64) | 0.01 (-0.10,0.13) | 0.85 |
| Mauritania | 0.13 (0.04,0.32) | 0.16 (0.06,0.33) | 0.04 (0.01,0.10) | 0.02 (0.01,0.05) | -1.75 (-1.92,-1.58) | 0 |
| Mauritius | 0.19 (0.08,0.39) | 0.33 (0.15,0.61) | 0.07 (0.03,0.15) | 0.14 (0.07,0.26) | 2.41 (1.77,3.06) | 0 |
| Mexico | 25.84 (18.82,38.40) | 69.80 (32.37,103.74) | 0.17 (0.12,0.25) | 0.29 (0.13,0.43) | 2.07 (1.42,2.72) | 0 |
| Micronesia (Federated States of) | 0.33 (0.13,0.72) | 0.27 (0.08,0.59) | 1.71 (0.65,3.70) | 1.32 (0.38,2.84) | -0.89 (-0.96,-0.82) | 0 |
| Monaco | 0.00 (0.00,0.01) | 0.00 (0.00,0.01) | 0.07 (0.03,0.15) | 0.08 (0.03,0.15) | 0.21 (0.16,0.26) | 0 |
| Mongolia | 1.04 (0.40,2.07) | 1.24 (0.47,2.68) | 0.24 (0.09,0.48) | 0.18 (0.07,0.38) | -1.14 (-1.65,-0.62) | 0 |
| Montenegro | 0.04 (0.02,0.07) | 0.02 (0.01,0.04) | 0.03 (0.01,0.05) | 0.02 (0.01,0.04) | -1.37 (-2.23,-0.51) | 0.002 |
| Morocco | 2.00 (0.44,5.65) | 3.43 (1.03,8.17) | 0.04 (0.01,0.12) | 0.05 (0.01,0.11) | 0.23 (-0.30,0.77) | 0.393 |
| Mozambique | 0.94 (0.20,2.55) | 3.39 (0.91,8.47) | 0.05 (0.01,0.13) | 0.07 (0.02,0.18) | 1.33 (1.16,1.50) | 0 |
| Myanmar | 5.47 (1.27,18.82) | 7.41 (2.12,19.46) | 0.07 (0.02,0.23) | 0.07 (0.02,0.18) | 0.12 (0.04,0.19) | 0.003 |
| Namibia | 0.24 (0.07,0.55) | 0.48 (0.16,1.01) | 0.11 (0.03,0.24) | 0.11 (0.04,0.22) | 0.03 (-0.20,0.26) | 0.823 |
| Nauru | 0.03 (0.01,0.07) | 0.03 (0.01,0.07) | 1.74 (0.70,3.71) | 1.40 (0.58,2.98) | -0.74 (-0.78,-0.69) | 0 |
| Nepal | 4.12 (1.22,11.54) | 6.31 (2.14,14.92) | 0.13 (0.04,0.36) | 0.11 (0.04,0.28) | -0.29 (-0.39,-0.19) | 0 |
| Netherlands | 1.10 (0.53,2.14) | 1.76 (0.56,3.27) | 0.03 (0.02,0.07) | 0.06 (0.02,0.12) | 2.09 (1.55,2.63) | 0 |
| New Zealand | 0.37 (0.21,0.69) | 0.70 (0.32,1.18) | 0.05 (0.03,0.10) | 0.10 (0.05,0.17) | 2.25 (1.64,2.86) | 0 |
| Nicaragua | 0.37 (0.17,0.69) | 1.39 (0.54,2.66) | 0.06 (0.03,0.11) | 0.10 (0.04,0.19) | 1.80 (1.32,2.28) | 0 |
| Niger | 0.57 (0.15,1.49) | 1.35 (0.45,3.17) | 0.05 (0.01,0.12) | 0.04 (0.01,0.09) | -0.58 (-1.03,-0.14) | 0.01 |
| Nigeria | 3.90 (1.44,7.35) | 7.94 (4.09,13.82) | 0.03 (0.01,0.05) | 0.02 (0.01,0.04) | -0.35 (-0.53,-0.17) | 0 |
| Niue | 0.01 (0.00,0.01) | 0.00 (0.00,0.00) | 1.27 (0.53,2.52) | 0.82 (0.34,1.65) | -1.54 (-1.73,-1.34) | 0 |
| North Macedonia | 0.12 (0.06,0.22) | 0.11 (0.05,0.21) | 0.03 (0.01,0.05) | 0.03 (0.01,0.05) | -0.32 (-0.63,0.00) | 0.049 |
| Northern Mariana Islands | 0.19 (0.08,0.36) | 0.08 (0.04,0.15) | 1.53 (0.66,2.90) | 1.28 (0.64,2.28) | -0.65 (-0.89,-0.41) | 0 |
| Norway | 0.69 (0.46,0.99) | 1.33 (0.64,1.86) | 0.08 (0.05,0.12) | 0.14 (0.07,0.20) | 1.83 (1.43,2.23) | 0 |
| Oman | 0.54 (0.12,1.57) | 1.40 (0.39,3.39) | 0.10 (0.02,0.30) | 0.07 (0.02,0.18) | -0.97 (-1.34,-0.60) | 0 |
| Pakistan | 42.80 (17.28,87.71) | 94.19 (46.96,180.35) | 0.24 (0.10,0.48) | 0.23 (0.11,0.44) | -0.06 (-0.15,0.03) | 0.166 |
| Palau | 0.09 (0.04,0.19) | 0.09 (0.04,0.17) | 2.66 (1.10,5.34) | 2.19 (0.94,4.28) | -0.66 (-0.88,-0.43) | 0 |
| Palestine | 0.61 (0.25,1.29) | 1.70 (0.84,2.91) | 0.18 (0.08,0.38) | 0.17 (0.09,0.29) | -0.22 (-0.71,0.27) | 0.38 |
| Panama | 0.60 (0.28,1.12) | 1.49 (0.75,2.60) | 0.12 (0.05,0.23) | 0.18 (0.09,0.32) | 1.55 (0.71,2.40) | 0 |
| Papua New Guinea | 6.81 (2.28,15.01) | 16.78 (6.24,34.53) | 0.83 (0.28,1.81) | 0.82 (0.31,1.69) | -0.09 (-0.22,0.05) | 0.196 |
| Paraguay | 0.44 (0.15,1.07) | 1.25 (0.53,2.61) | 0.06 (0.02,0.14) | 0.08 (0.04,0.18) | 1.25 (0.69,1.81) | 0 |
| Peru | 28.55 (12.63,50.82) | 40.13 (20.10,69.03) | 0.71 (0.31,1.25) | 0.59 (0.30,1.01) | -0.51 (-1.56,0.55) | 0.347 |
| Philippines | 1.83 (1.03,2.64) | 4.74 (2.62,6.70) | 0.02 (0.01,0.02) | 0.02 (0.01,0.03) | 1.06 (0.83,1.30) | 0 |
| Poland | 8.55 (5.08,12.36) | 6.85 (4.04,9.43) | 0.11 (0.06,0.15) | 0.09 (0.05,0.13) | -0.61 (-0.89,-0.33) | 0 |
| Portugal | 1.82 (0.99,3.59) | 2.53 (0.72,4.76) | 0.10 (0.05,0.19) | 0.16 (0.04,0.30) | 1.65 (0.26,3.06) | 0.02 |
| Puerto Rico | 1.16 (0.58,2.34) | 1.58 (0.83,2.68) | 0.18 (0.09,0.36) | 0.28 (0.15,0.48) | 1.56 (0.58,2.54) | 0.002 |
| Qatar | 0.06 (0.02,0.13) | 0.44 (0.17,0.92) | 0.03 (0.01,0.07) | 0.03 (0.01,0.06) | -0.58 (-1.20,0.04) | 0.066 |
| Republic of Korea | 9.31 (3.53,23.99) | 6.52 (3.55,11.34) | 0.09 (0.03,0.22) | 0.07 (0.04,0.12) | -0.98 (-1.26,-0.70) | 0 |
| Republic of Moldova | 0.42 (0.20,0.79) | 0.27 (0.11,0.49) | 0.05 (0.02,0.09) | 0.03 (0.01,0.06) | -1.17 (-2.69,0.36) | 0.134 |
| Romania | 17.82 (9.13,29.90) | 5.79 (3.20,9.55) | 0.39 (0.20,0.66) | 0.18 (0.10,0.30) | -2.71 (-3.37,-2.04) | 0 |
| Russian Federation | 16.49 (10.49,23.09) | 11.20 (6.89,19.22) | 0.05 (0.03,0.07) | 0.04 (0.02,0.07) | -0.95 (-1.60,-0.29) | 0.005 |
| Rwanda | 1.88 (0.37,5.58) | 2.89 (0.90,6.76) | 0.15 (0.03,0.45) | 0.12 (0.04,0.27) | -0.94 (-1.21,-0.67) | 0 |
| Saint Kitts and Nevis | 0.01 (0.00,0.01) | 0.01 (0.00,0.03) | 0.09 (0.04,0.18) | 0.11 (0.03,0.23) | 1.10 (-0.25,2.47) | 0.11 |
| Saint Lucia | 0.04 (0.02,0.07) | 0.12 (0.06,0.21) | 0.16 (0.08,0.28) | 0.34 (0.17,0.61) | 2.68 (1.88,3.49) | 0 |
| Saint Vincent and the Grenadines | 0.01 (0.00,0.03) | 0.03 (0.01,0.05) | 0.05 (0.01,0.13) | 0.13 (0.07,0.24) | 3.59 (2.39,4.79) | 0 |
| Samoa | 0.40 (0.15,0.79) | 0.39 (0.14,0.77) | 1.19 (0.45,2.36) | 0.96 (0.34,1.92) | -0.74 (-0.80,-0.68) | 0 |
| San Marino | 0.00 (0.00,0.00) | 0.00 (0.00,0.00) | 0.02 (0.01,0.05) | 0.04 (0.01,0.09) | 1.60 (1.38,1.83) | 0 |
| Sao Tome and Principe | 0.01 (0.00,0.03) | 0.03 (0.01,0.06) | 0.09 (0.03,0.20) | 0.07 (0.03,0.15) | -0.57 (-0.97,-0.18) | 0.005 |
| Saudi Arabia | 3.82 (1.16,9.33) | 20.47 (8.55,43.22) | 0.10 (0.03,0.25) | 0.16 (0.07,0.34) | 1.64 (1.42,1.86) | 0 |
| Senegal | 0.49 (0.14,1.20) | 0.98 (0.41,1.91) | 0.04 (0.01,0.10) | 0.04 (0.02,0.07) | -0.57 (-1.12,-0.02) | 0.043 |
| Serbia | 1.40 (0.60,2.72) | 0.93 (0.37,1.73) | 0.08 (0.03,0.15) | 0.06 (0.02,0.11) | -0.87 (-1.28,-0.45) | 0 |
| Seychelles | 0.01 (0.00,0.01) | 0.01 (0.00,0.02) | 0.03 (0.01,0.09) | 0.04 (0.01,0.09) | 0.26 (-0.27,0.79) | 0.346 |
| Sierra Leone | 0.20 (0.06,0.50) | 0.50 (0.20,1.07) | 0.03 (0.01,0.08) | 0.03 (0.01,0.07) | -0.16 (-0.47,0.16) | 0.329 |
| Singapore | 1.44 (0.51,2.73) | 1.23 (0.32,2.26) | 0.18 (0.07,0.34) | 0.11 (0.03,0.20) | -1.66 (-2.85,-0.45) | 0.007 |
| Slovakia | 0.83 (0.42,1.51) | 0.89 (0.36,1.65) | 0.08 (0.04,0.14) | 0.09 (0.03,0.16) | 0.43 (0.16,0.69) | 0.002 |
| Slovenia | 0.20 (0.09,0.43) | 0.19 (0.09,0.31) | 0.05 (0.02,0.10) | 0.05 (0.03,0.09) | 0.37 (-0.40,1.14) | 0.347 |
| Solomon Islands | 0.54 (0.16,1.45) | 1.33 (0.48,3.21) | 0.87 (0.26,2.42) | 1.05 (0.38,2.55) | 0.67 (0.50,0.84) | 0 |
| Somalia | 1.35 (0.23,4.21) | 3.40 (0.69,10.76) | 0.11 (0.02,0.35) | 0.09 (0.02,0.29) | -0.65 (-0.78,-0.53) | 0 |
| South Africa | 17.04 (9.30,24.62) | 20.67 (12.52,36.25) | 0.25 (0.14,0.36) | 0.16 (0.10,0.28) | -1.62 (-2.80,-0.43) | 0.008 |
| South Sudan | 1.07 (0.23,2.83) | 1.09 (0.33,2.67) | 0.10 (0.02,0.26) | 0.08 (0.02,0.19) | -0.75 (-1.19,-0.31) | 0.001 |
| Spain | 11.05 (6.44,19.30) | 10.80 (4.44,17.16) | 0.15 (0.09,0.26) | 0.15 (0.06,0.23) | -0.25 (-0.68,0.17) | 0.245 |
| Sri Lanka | 1.79 (0.72,3.91) | 1.54 (0.61,3.18) | 0.05 (0.02,0.11) | 0.04 (0.02,0.08) | -0.81 (-1.97,0.37) | 0.177 |
| Sudan | 2.72 (0.49,8.08) | 7.48 (2.00,17.56) | 0.08 (0.02,0.24) | 0.10 (0.03,0.22) | 0.55 (0.43,0.67) | 0 |
| Suriname | 0.09 (0.04,0.18) | 0.26 (0.12,0.52) | 0.13 (0.06,0.25) | 0.25 (0.11,0.49) | 2.12 (0.54,3.73) | 0.008 |
| Sweden | 0.94 (0.55,1.66) | 1.71 (0.99,2.52) | 0.06 (0.03,0.11) | 0.10 (0.06,0.15) | 1.75 (1.28,2.23) | 0 |
| Switzerland | 1.48 (0.67,3.01) | 1.29 (0.67,2.11) | 0.10 (0.05,0.21) | 0.09 (0.04,0.14) | -0.83 (-1.98,0.34) | 0.166 |
| Syrian Arab Republic | 0.72 (0.24,1.63) | 1.01 (0.37,2.13) | 0.03 (0.01,0.07) | 0.04 (0.02,0.09) | 0.98 (0.66,1.29) | 0 |
| Taiwan (Province of China) | 1.72 (0.89,3.39) | 3.66 (1.33,6.76) | 0.04 (0.02,0.07) | 0.08 (0.03,0.15) | 2.89 (2.40,3.39) | 0 |
| Tajikistan | 3.62 (1.47,7.16) | 7.71 (2.51,15.77) | 0.41 (0.17,0.81) | 0.40 (0.13,0.81) | -0.12 (-0.78,0.54) | 0.721 |
| Thailand | 4.47 (1.63,10.03) | 9.00 (2.75,20.18) | 0.03 (0.01,0.08) | 0.07 (0.02,0.16) | 2.58 (2.00,3.17) | 0 |
| Timor-Leste | 0.06 (0.01,0.15) | 0.10 (0.02,0.28) | 0.04 (0.01,0.09) | 0.04 (0.01,0.11) | 0.54 (-0.64,1.74) | 0.369 |
| Togo | 0.25 (0.08,0.60) | 0.54 (0.22,1.12) | 0.04 (0.02,0.11) | 0.04 (0.02,0.08) | -0.43 (-0.88,0.01) | 0.055 |
| Tokelau | 0.00 (0.00,0.01) | 0.00 (0.00,0.00) | 1.07 (0.41,2.11) | 0.81 (0.31,1.55) | -0.97 (-1.00,-0.95) | 0 |
| Tonga | 0.12 (0.04,0.23) | 0.14 (0.05,0.28) | 0.72 (0.25,1.41) | 0.79 (0.31,1.55) | 0.31 (0.15,0.46) | 0 |
| Trinidad and Tobago | 0.46 (0.22,0.84) | 0.71 (0.31,1.39) | 0.19 (0.09,0.35) | 0.24 (0.10,0.48) | 0.79 (0.33,1.25) | 0.001 |
| Tunisia | 0.66 (0.16,1.68) | 1.37 (0.47,2.94) | 0.04 (0.01,0.10) | 0.06 (0.02,0.13) | 1.26 (1.12,1.41) | 0 |
| Turkey | 22.78 (8.96,49.34) | 24.27 (10.67,45.76) | 0.19 (0.07,0.41) | 0.13 (0.06,0.25) | -1.35 (-1.94,-0.75) | 0 |
| Turkmenistan | 0.87 (0.38,1.70) | 0.92 (0.40,1.83) | 0.13 (0.06,0.25) | 0.08 (0.04,0.17) | -1.44 (-2.13,-0.75) | 0 |
| Tuvalu | 0.02 (0.01,0.05) | 0.03 (0.01,0.05) | 1.45 (0.58,2.94) | 1.20 (0.56,2.19) | -0.65 (-0.74,-0.56) | 0 |
| Uganda | 3.53 (0.71,10.06) | 8.60 (2.73,21.33) | 0.13 (0.03,0.37) | 0.13 (0.04,0.32) | -0.15 (-0.61,0.32) | 0.532 |
| Ukraine | 18.11 (8.72,31.78) | 11.75 (5.98,21.26) | 0.18 (0.09,0.32) | 0.14 (0.07,0.25) | -0.96 (-3.05,1.18) | 0.378 |
| United Arab Emirates | 2.09 (0.60,5.53) | 17.14 (5.64,41.36) | 0.26 (0.07,0.70) | 0.35 (0.11,0.88) | 1.04 (0.75,1.33) | 0 |
| United Kingdom | 10.32 (7.19,18.92) | 20.89 (11.61,25.82) | 0.10 (0.07,0.18) | 0.17 (0.10,0.22) | 2.07 (1.64,2.49) | 0 |
| United Republic of Tanzania | 2.93 (0.71,7.31) | 7.22 (2.19,16.82) | 0.07 (0.02,0.18) | 0.08 (0.02,0.18) | 0.17 (-0.01,0.35) | 0.064 |
| United States of America | 134.82 (97.19,170.66) | 103.52 (78.73,145.17) | 0.25 (0.18,0.31) | 0.18 (0.14,0.25) | -1.06 (-1.37,-0.75) | 0 |
| United States Virgin Islands | 0.04 (0.02,0.08) | 0.03 (0.01,0.07) | 0.22 (0.10,0.43) | 0.22 (0.10,0.43) | 0.17 (-0.61,0.95) | 0.676 |
| Uruguay | 0.38 (0.17,0.78) | 0.84 (0.32,1.59) | 0.07 (0.03,0.14) | 0.14 (0.05,0.26) | 2.79 (2.11,3.47) | 0 |
| Uzbekistan | 11.45 (4.53,20.67) | 17.05 (7.40,28.96) | 0.29 (0.11,0.52) | 0.24 (0.10,0.41) | -0.69 (-1.29,-0.09) | 0.025 |
| Vanuatu | 0.41 (0.14,0.89) | 0.85 (0.32,1.74) | 1.50 (0.50,3.25) | 1.53 (0.58,3.15) | 0.03 (-0.13,0.20) | 0.693 |
| Venezuela (Bolivarian Republic of) | 3.66 (1.81,6.48) | 6.45 (3.33,11.37) | 0.10 (0.05,0.17) | 0.12 (0.06,0.22) | 0.98 (0.50,1.46) | 0 |
| Viet Nam | 4.33 (1.21,11.18) | 6.12 (1.81,14.81) | 0.03 (0.01,0.08) | 0.03 (0.01,0.07) | -0.27 (-0.43,-0.11) | 0.001 |
| Yemen | 1.04 (0.17,4.05) | 3.85 (1.12,9.89) | 0.05 (0.01,0.20) | 0.06 (0.02,0.16) | 0.71 (0.47,0.96) | 0 |
| Zambia | 1.04 (0.23,2.68) | 3.30 (1.04,7.38) | 0.08 (0.02,0.21) | 0.10 (0.03,0.22) | 0.54 (0.35,0.73) | 0 |
| Zimbabwe | 0.71 (0.16,1.64) | 1.33 (0.33,3.25) | 0.05 (0.01,0.11) | 0.05 (0.01,0.12) | 0.36 (-0.26,0.98) | 0.261 |

NOTE:;ASMR,age-standardised mortality rate;APPC,average annual percentage change;

| **Table 13 Incidence decomposition** | | | | | | | | | | | | | | | | | | | | | | | | |
| --- | --- | --- | --- | --- | --- | --- | --- | --- | --- | --- | --- | --- | --- | --- | --- | --- | --- | --- | --- | --- | --- | --- | --- | --- |
| location | Both | | | | | | | | | Male | | | | | | | | Female | | | | | | |
|  | Overll difference | | | Aging | Population | | | Epidemiological change | | Overll difference | | Aging | Population | | Epidemiological change | | | Overll difference | | Aging | Population | | | Epidemiological change |
| Global | | 889652.19  (50.93%) | 134145.28 (7.68%) | | | 652284.96 (37.34%) | 103221.95 (5.91%) | | 434906.76  (48.68%) | | 63327.2 (7.09%) | | | 331972.88 (37.16%) | | 39606.68 (4.43%) | 454745.43  (53.29%) | | 70907.82 (8.31%) | | | 320320.58 (37.54%) | 63517.03 (7.44%) | |
| High SDI | | 16490.93  (4.88%) | 17134.59 (5.07%) | | | 8711.38 (2.58%) | -9355.04 (-2.77%) | | 17097.13  (10.17%) | | 9072.11 (5.4%) | | | 7323.82 (4.36%) | | 701.2 (0.42%) | -606.2  (-0.36%) | | 8052.98 (4.75%) | | | 1349.19 (0.8%) | -10008.37 (-5.9%) | |
| High-middle SDI | | 29329.31  (6.39%) | 51072.72 (11.13%) | | | 31538.76 (6.87%) | -53282.17 (-11.61%) | | 14118.55  (5.9%) | | 25254.87 (10.55%) | | | 20582.1 (8.6%) | | -31718.41 (-13.25%) | 15210.76  (6.93%) | | 25869.19 (11.78%) | | | 11087.85 (5.05%) | -21746.28 (-9.9%) | |
| Middle SDI | | 264886.56  (60.68%) | 58506.2 (13.4%) | | | 125490.8 (28.75%) | 80889.56 (18.53%) | | 126529.45  (56.12%) | | 27119.48 (12.03%) | | | 63193.82 (28.03%) | | 36216.14 (16.06%) | 138357.11  (65.54%) | | 31421.39 (14.89%) | | | 62285.82 (29.51%) | 44649.9 (21.15%) | |
| Low-middle SDI | | 333104.87  (95.28%) | 28191.41 (8.06%) | | | 248178.95 (70.99%) | 56734.51 (16.23%) | | 159612.05  (88.32%) | | 13075.79 (7.24%) | | | 124426.74 (68.85%) | | 22109.52 (12.23%) | 173492.82  (102.73%) | | 15110.1 (8.95%) | | | 123718.01 (73.26%) | 34664.71 (20.53%) | |
| Low SDI | | 245144.9  (150.2%) | 65.51 (0.04%) | | | 222692.98 (136.45%) | 22386.41 (13.72%) | | 117184.57  (147.51%) | | -216.07 (-0.27%) | | | 107745.3 (135.63%) | | 9655.34 (12.15%) | 127960.34  (152.76%) | | 318.65 (0.38%) | | | 114948.47 (137.22%) | 12693.22 (15.15%) | |
| Andean Latin America | | 12813.78  (122.86%) | 1558.19 (14.94%) | | | 8042.1 (77.11%) | 3213.48 (30.81%) | | 7001.62  (125.09%) | | 751.79 (13.43%) | | | 4558.12 (81.43%) | | 1691.7 (30.22%) | 5812.16  (120.28%) | | 796.56 (16.48%) | | | 3518.39 (72.81%) | 1497.21 (30.99%) | |
| Australasia | | 1012.72  (16.74%) | 229.12 (3.79%) | | | 1147.89 (18.97%) | -364.29 (-6.02%) | | 560.32  (17.66%) | | 113.17 (3.57%) | | | 589.09 (18.57%) | | -141.94 (-4.47%) | 452.41  (15.72%) | | 114.29 (3.97%) | | | 557.35 (19.37%) | -219.23 (-7.62%) | |
| Caribbean | | 3216.41  (46.41%) | 654.88 (9.45%) | | | 1687.48 (24.35%) | 874.05 (12.61%) | | 1559.86  (46.24%) | | 292.19 (8.66%) | | | 876.71 (25.99%) | | 390.96 (11.59%) | 1656.54  (46.56%) | | 364.45 (10.24%) | | | 809.24 (22.75%) | 482.85 (13.57%) | |
| Central Asia | | 14544.05  (48.02%) | 2984.83 (9.86%) | | | 10570.27 (34.9%) | 988.96 (3.27%) | | 7899.72  (53.78%) | | 1454.39 (9.9%) | | | 5507.76 (37.49%) | | 937.56 (6.38%) | 6644.33  (42.6%) | | 1535.31 (9.84%) | | | 5053.64 (32.4%) | 55.38 (0.36%) | |
| Central Europe | | -6518.09  (-7.14%) | 6893.32 (7.55%) | | | -22671.44 (-24.82%) | 9260.03 (10.14%) | | -1597.01  (-3.1%) | | 4074.82 (7.91%) | | | -12670.39 (-24.61%) | | 6998.57 (13.59%) | -4921.09  (-12.35%) | | 2832.24 (7.11%) | | | -9903.08 (-24.85%) | 2149.75 (5.4%) | |
| Central Latin America | | 16130.5  (40.83%) | 4605.4 (11.66%) | | | 18547.43 (46.95%) | -7022.34 (-17.78%) | | 10069.75  (46.36%) | | 2145.5 (9.88%) | | | 10703.05 (49.28%) | | -2778.8 (-12.79%) | 6060.75  (34.08%) | | 2465.63 (13.86%) | | | 7923.57 (44.55%) | -4328.45 (-24.34%) | |
| Central Sub-Saharan Africa | | 29679.85  (169.88%) | 221.99 (1.27%) | | | 27183.06 (155.59%) | 2274.81 (13.02%) | | 14442.6  (172.92%) | | 136.13 (1.63%) | | | 13079.36 (156.6%) | | 1227.1 (14.69%) | 15237.25  (167.09%) | | 79.62 (0.87%) | | | 14102.94 (154.66%) | 1054.69 (11.57%) | |
| East Asia | | -52604.06  (-22.81%) | 30734.32 (13.33%) | | | -19380.49 (-8.4%) | -63957.88 (-27.74%) | | -29866.2  (-24.42%) | | 15219.65 (12.45%) | | | -10130.39 (-8.28%) | | -34955.46 (-28.58%) | -22737.85  (-20.99%) | | 15435.33 (14.25%) | | | -9247.06 (-8.54%) | -28926.12 (-26.71%) | |
| Eastern Europe | | -19096.47  (-9.97%) | 15281.74 (7.98%) | | | -40702.53 (-21.25%) | 6324.32 (3.3%) | | -10923.6  (-11.17%) | | 7175.54 (7.34%) | | | -20374.03 (-20.83%) | | 2274.89 (2.33%) | -8172.87  (-8.72%) | | 8142.48 (8.69%) | | | -20324.73 (-21.68%) | 4009.37 (4.28%) | |
| Eastern Sub-Saharan Africa | | 82225.8  (150.51%) | 950.25 (1.74%) | | | 76781.12 (140.55%) | 4494.43 (8.23%) | | 37781.54  (154.24%) | | 291.81 (1.19%) | | | 35279.35 (144.02%) | | 2210.38 (9.02%) | 44444.26  (147.49%) | | 696.83 (2.31%) | | | 41418.46 (137.44%) | 2328.96 (7.73%) | |
| High-income Asia Pacific | | -15559.64  (-24.16%) | 3388.36 (5.26%) | | | -14131.55 (-21.94%) | -4816.45 (-7.48%) | | -6286.85  (-17.48%) | | 2189.49 (6.09%) | | | -7696.02 (-21.4%) | | -780.32 (-2.17%) | -9272.78  (-32.6%) | | 1227.75 (4.32%) | | | -6304.03 (-22.16%) | -4196.5 (-14.75%) | |
| High-income North America | | -9089.15  (-4.2%) | -4371.22 (-2.02%) | | | 15266.06 (7.05%) | -19983.99 (-9.23%) | | 793.09  (0.79%) | | -2135.33 (-2.12%) | | | 7613.34 (7.57%) | | -4684.92 (-4.66%) | -9882.24  (-8.53%) | | -2220.93 (-1.92%) | | | 7611.47 (6.57%) | -15272.77 (-13.18%) | |
| North Africa and Middle East | | 216235.99  (139.58%) | 30380.27 (19.61%) | | | 157717.45 (101.8%) | 28138.27 (18.16%) | | 105758.72  (143.49%) | | 13969.48 (18.95%) | | | 77812.29 (105.57%) | | 13976.95 (18.96%) | 110477.27  (136.02%) | | 16283.98 (20.05%) | | | 79559.05 (97.96%) | 14634.24 (18.02%) | |
| Oceania | | 4955.92  (144.11%) | 201.9 (5.87%) | | | 4000.81 (116.34%) | 753.21 (21.9%) | | 3042.08  (140.96%) | | 91.54 (4.24%) | | | 2469.67 (114.44%) | | 480.88 (22.28%) | 1913.83  (149.42%) | | 105.72 (8.25%) | | | 1521.99 (118.82%) | 286.12 (22.34%) | |
| South Asia | | 472971.77  (117.52%) | 28869.6 (7.17%) | | | 348242.64 (86.53%) | 95859.53 (23.82%) | | 227712.72  (105.45%) | | 13487.84 (6.25%) | | | 177680.18 (82.28%) | | 36544.71 (16.92%) | 245259.05  (131.48%) | | 15345.83 (8.23%) | | | 170404.22 (91.35%) | 59509 (31.9%) | |
| Southeast Asia | | 27797.98  (59.3%) | 4457.2 (9.51%) | | | 19166.87 (40.89%) | 4173.91 (8.9%) | | 15598  (65.31%) | | 2222.89 (9.31%) | | | 10737.21 (44.96%) | | 2637.9 (11.04%) | 12199.98  (53.07%) | | 2235.43 (9.72%) | | | 8488.9 (36.92%) | 1475.65 (6.42%) | |
| Southern Latin America | | 4118  (34.82%) | 611.09 (5.17%) | | | 3956.86 (33.46%) | -449.95 (-3.8%) | | 2662.99  (45.36%) | | 304.1 (5.18%) | | | 2092.29 (35.64%) | | 266.6 (4.54%) | 1455.01  (24.43%) | | 304.73 (5.12%) | | | 1868.11 (31.37%) | -717.82 (-12.05%) | |
| Southern Sub-Saharan Africa | | 21129.22  (68.31%) | 3746.34 (12.11%) | | | 17384.57 (56.2%) | -1.69 (-0.01%) | | 10556.66  (71.94%) | | 1662 (11.33%) | | | 9024.75 (61.5%) | | -130.09 (-0.89%) | 10572.56  (65.03%) | | 2094.55 (12.88%) | | | 8330.76 (51.24%) | 147.25 (0.91%) | |
| Tropical Latin America | | -18416.4  (-39.7%) | 4562.14 (9.84%) | | | 13078.43 (28.19%) | -36056.97 (-77.73%) | | -8608.44  (-38.68%) | | 1991.31 (8.95%) | | | 6496.06 (29.19%) | | -17095.82 (-76.82%) | -9807.96  (-40.64%) | | 2586.8 (10.72%) | | | 6572.86 (27.24%) | -18967.62 (-78.6%) | |
| Western Europe | | -1381.72  (-5.09%) | 1255.42 (4.62%) | | | -2510.45 (-9.24%) | -126.69 (-0.47%) | | -818.52  (-5.16%) | | 693.78 (4.38%) | | | -1378.74 (-8.7%) | | -133.56 (-0.84%) | -563.2  (-4.98%) | | 552.18 (4.88%) | | | -1109.45 (-9.8%) | -5.92 (-0.05%) | |
| Western Sub-Saharan Africa | | 105485.73  (167.18%) | -126.59 (-0.2%) | | | 98774.01 (156.54%) | 6838.32 (10.84%) | | 47567.72  (160.74%) | | -836.2 (-2.83%) | | | 45150.05 (152.57%) | | 3253.87 (11%) | 57918.01  (172.86%) | | 878.82 (2.62%) | | | 53662.87 (160.16%) | 3376.33 (10.08%) | |

| **Table 14 DALYs decomposition** | | | | | | | | | | | | |
| --- | --- | --- | --- | --- | --- | --- | --- | --- | --- | --- | --- | --- |
| location | Both | | | | Male | | | | Female | | | |
|  | Overll difference | Aging | Population | Epidemiological change | Overll difference | Aging | Population | Epidemiological change | Overll difference | Aging | Population | Epidemiological change |
| Global | 82496.57 (52%) | 11344.49 (7.15%) | 59453.04 (37.47%) | 11699.04 (7.37%) | 43361.83 (48.31%) | 5879.95 (6.55%) | 33300.18 (37.1%) | 4181.71 (4.66%) | 39134.74 (56.8%) | 5408.55 (7.85%) | 26171.32 (37.99%) | 7554.88 (10.97%) |
| High SDI | 828 (2.47%) | 1522.01 (4.53%) | 856.98 (2.55%) | -1550.99 (-4.62%) | 630.42 (3.54%) | 851.71 (4.79%) | 752.04 (4.23%) | -973.33 (-5.47%) | 197.57 (1.25%) | 676.55 (4.28%) | 126.72 (0.8%) | -605.7 (-3.83%) |
| High-middle SDI | 1978.32 (6.25%) | 2637.06 (8.33%) | 2171.84 (6.86%) | -2830.59 (-8.94%) | 985.65 (5.68%) | 1406.47 (8.11%) | 1488.47 (8.58%) | -1909.29 (-11%) | 992.67 (6.94%) | 1226.08 (8.57%) | 721.98 (5.05%) | -955.39 (-6.68%) |
| Middle SDI | 24999.16 (62.6%) | 4680.85 (11.72%) | 11563.93 (28.96%) | 8754.37 (21.92%) | 12847.27 (59.06%) | 2317 (10.65%) | 6160.43 (28.32%) | 4369.84 (20.09%) | 12151.89 (66.84%) | 2342.83 (12.89%) | 5396.34 (29.68%) | 4412.72 (24.27%) |
| Low-middle SDI | 35682.14 (90.63%) | 3372.96 (8.57%) | 27590.4 (70.08%) | 4718.78 (11.99%) | 19029.05 (77.48%) | 1862.87 (7.59%) | 16393.07 (66.75%) | 773.12 (3.15%) | 16653.09 (112.43%) | 1457.49 (9.84%) | 11131.68 (75.15%) | 4063.91 (27.44%) |
| Low SDI | 18895.68 (135.04%) | 5.3 (0.04%) | 18490.96 (132.15%) | 399.42 (2.85%) | 9807.15 (119.06%) | -22.76 (-0.28%) | 10507.33 (127.56%) | -677.42 (-8.22%) | 9088.53 (157.92%) | 33.13 (0.58%) | 7981.62 (138.69%) | 1073.78 (18.66%) |
| Andean Latin America | 2615.35 (66.46%) | 276.17 (7.02%) | 2603.28 (66.16%) | -264.09 (-6.71%) | 1522.52 (64.12%) | 155.54 (6.55%) | 1640.41 (69.09%) | -273.43 (-11.52%) | 1092.83 (70.02%) | 117.64 (7.54%) | 990.74 (63.48%) | -15.54 (-1%) |
| Australasia | 365.29 (122.75%) | 18.21 (6.12%) | 81.52 (27.39%) | 265.57 (89.24%) | 214.71 (124.41%) | 9.08 (5.26%) | 46.38 (26.88%) | 159.24 (92.27%) | 150.58 (120.47%) | 8.91 (7.13%) | 34.81 (27.85%) | 106.86 (85.49%) |
| Caribbean | 970.6 (105.92%) | 99.39 (10.85%) | 271.69 (29.65%) | 599.51 (65.43%) | 460.75 (103.88%) | 41.48 (9.35%) | 139.49 (31.45%) | 279.78 (63.08%) | 509.85 (107.85%) | 57.94 (12.26%) | 131.76 (27.87%) | 320.15 (67.72%) |
| Central Asia | 921.76 (23.32%) | 319.04 (8.07%) | 1258.42 (31.84%) | -655.7 (-16.59%) | 740.02 (45.68%) | 141.43 (8.73%) | 589.28 (36.38%) | 9.31 (0.57%) | 181.74 (7.79%) | 177.32 (7.6%) | 660.72 (28.33%) | -656.3 (-28.14%) |
| Central Europe | -1711.94 (-34.35%) | 250.8 (5.03%) | -1043.29 (-20.93%) | -919.44 (-18.45%) | -961.88 (-33.76%) | 150.73 (5.29%) | -580.68 (-20.38%) | -531.93 (-18.67%) | -750.06 (-35.12%) | 100.81 (4.72%) | -458.46 (-21.47%) | -392.41 (-18.38%) |
| Central Latin America | 7542.73 (122.47%) | 664.99 (10.8%) | 3689.55 (59.91%) | 3188.19 (51.77%) | 4172.8 (141.96%) | 306.79 (10.44%) | 1905.92 (64.84%) | 1960.08 (66.68%) | 3369.93 (104.67%) | 359.73 (11.17%) | 1785.99 (55.48%) | 1224.22 (38.03%) |
| Central Sub-Saharan Africa | 1705.89 (142.82%) | 16.2 (1.36%) | 1762 (147.52%) | -72.32 (-6.05%) | 963.8 (142.6%) | 11.73 (1.73%) | 997.34 (147.56%) | -45.26 (-6.7%) | 742.09 (143.11%) | 4.82 (0.93%) | 764.83 (147.49%) | -27.57 (-5.32%) |
| East Asia | -2910.66 (-17.78%) | 1746.78 (10.67%) | -1411.3 (-8.62%) | -3246.14 (-19.83%) | -1575.78 (-17.08%) | 887.37 (9.62%) | -793.3 (-8.6%) | -1669.85 (-18.1%) | -1334.88 (-18.69%) | 851.06 (11.92%) | -617.38 (-8.64%) | -1568.57 (-21.96%) |
| Eastern Europe | -1987.69 (-27.57%) | 473.02 (6.56%) | -1377.41 (-19.11%) | -1083.31 (-15.03%) | -976.41 (-26.17%) | 257.93 (6.91%) | -710.21 (-19.04%) | -524.12 (-14.05%) | -1011.28 (-29.07%) | 213.96 (6.15%) | -666.46 (-19.16%) | -558.78 (-16.06%) |
| Eastern Sub-Saharan Africa | 5287.48 (139.81%) | 65.56 (1.73%) | 5198.82 (137.47%) | 23.1 (0.61%) | 2828.92 (126.86%) | 25.79 (1.16%) | 3033.91 (136.06%) | -230.79 (-10.35%) | 2458.57 (158.42%) | 40.63 (2.62%) | 2181.5 (140.57%) | 236.43 (15.23%) |
| High-income Asia Pacific | -1414.02 (-24.37%) | 313.5 (5.4%) | -1270.23 (-21.89%) | -457.29 (-7.88%) | -811.77 (-23.22%) | 199.98 (5.72%) | -721.5 (-20.64%) | -290.25 (-8.3%) | -602.25 (-26.11%) | 116.92 (5.07%) | -533.26 (-23.12%) | -185.91 (-8.06%) |
| High-income North America | -3049.64 (-15.52%) | -371.03 (-1.89%) | 1307.42 (6.65%) | -3986.03 (-20.29%) | -1720.42 (-17.42%) | -191.85 (-1.94%) | 681.69 (6.9%) | -2210.27 (-22.38%) | -1329.21 (-13.6%) | -177.73 (-1.82%) | 624.97 (6.4%) | -1776.46 (-18.18%) |
| North Africa and Middle East | 12075.03 (137.46%) | 1352.43 (15.4%) | 8886.3 (101.16%) | 1836.3 (20.9%) | 6247.47 (134.9%) | 714.34 (15.42%) | 4781.59 (103.25%) | 751.53 (16.23%) | 5827.56 (140.32%) | 636.64 (15.33%) | 4108.44 (98.93%) | 1082.48 (26.06%) |
| Oceania | 1344.09 (118.28%) | 25.74 (2.26%) | 1246.96 (109.73%) | 71.39 (6.28%) | 827.08 (109.74%) | 4.04 (0.54%) | 802.44 (106.47%) | 20.6 (2.73%) | 517.01 (135.1%) | 24.12 (6.3%) | 440.89 (115.21%) | 52 (13.59%) |
| South Asia | 48152.03 (100.07%) | 3599.31 (7.48%) | 39831.83 (82.78%) | 4720.89 (9.81%) | 24973.54 (81.87%) | 1909.69 (6.26%) | 23570.59 (77.27%) | -506.74 (-1.66%) | 23178.48 (131.6%) | 1627.87 (9.24%) | 16101.05 (91.41%) | 5449.56 (30.94%) |
| Southeast Asia | 3299.15 (67.37%) | 377.1 (7.7%) | 2055.53 (41.98%) | 866.52 (17.7%) | 1746.71 (60.69%) | 94.11 (3.27%) | 1273.56 (44.25%) | 379.04 (13.17%) | 1552.44 (76.91%) | 292.22 (14.48%) | 806.72 (39.97%) | 453.49 (22.47%) |
| Southern Latin America | 986.2 (44.9%) | 97.23 (4.43%) | 762.2 (34.7%) | 126.77 (5.77%) | 573.77 (57.38%) | 48.13 (4.81%) | 371.7 (37.17%) | 153.94 (15.39%) | 412.43 (34.48%) | 49.58 (4.14%) | 389.6 (32.57%) | -26.75 (-2.24%) |
| Southern Sub-Saharan Africa | 564.33 (20.38%) | 397.36 (14.35%) | 1335.56 (48.24%) | -1168.58 (-42.2%) | 442.41 (34.37%) | 208.88 (16.23%) | 707.39 (54.95%) | -473.87 (-36.81%) | 121.92 (8.23%) | 187.1 (12.63%) | 627.2 (42.33%) | -692.39 (-46.73%) |
| Tropical Latin America | 2701.51 (42.82%) | 648.24 (10.27%) | 2476.5 (39.25%) | -423.24 (-6.71%) | 1255.38 (35.68%) | 389.32 (11.06%) | 1388.24 (39.45%) | -522.18 (-14.84%) | 1446.13 (51.83%) | 253.71 (9.09%) | 1095.95 (39.28%) | 96.47 (3.46%) |
| Western Europe | 1623.29 (20.49%) | 383.68 (4.84%) | -831.65 (-10.5%) | 2071.26 (26.15%) | 893.48 (20.2%) | 218.21 (4.93%) | -436.51 (-9.87%) | 1111.78 (25.13%) | 729.81 (20.86%) | 161.74 (4.62%) | -390.31 (-11.16%) | 958.38 (27.4%) |
| Western Sub-Saharan Africa | 3415.79 (149.99%) | -11.9 (-0.52%) | 3446.76 (151.35%) | -19.08 (-0.84%) | 1544.72 (136.97%) | -35.95 (-3.19%) | 1640.01 (145.42%) | -59.34 (-5.26%) | 1871.07 (162.77%) | 28.51 (2.48%) | 1806.65 (157.16%) | 35.91 (3.12%) |

NOTE:;DALYs,disability-adjusted life years.

| **Table 15 Deaths decomposition** | | | | | | | | | | | | |
| --- | --- | --- | --- | --- | --- | --- | --- | --- | --- | --- | --- | --- |
| location | Both | | | | Male | | | | Female | | | |
|  | Overll difference | Aging | Population | Epidemiological change | Overll difference | Aging | Population | Epidemiological change | Overll difference | Aging | Population | Epidemiological change |
| Global | 1227.89 (55.81%) | 181.97 (8.27%) | 835.1 (37.96%) | 210.82 (9.58%) | 654.05 (51.4%) | 96.37 (7.57%) | 477.08 (37.5%) | 80.6 (6.33%) | 573.85 (61.85%) | 84.44 (9.1%) | 358.32 (38.62%) | 131.08 (14.13%) |
| High SDI | 16.33 (3.62%) | 22.92 (5.08%) | 11.57 (2.57%) | -18.16 (-4.03%) | 9.53 (3.93%) | 12.97 (5.35%) | 10.27 (4.24%) | -13.71 (-5.66%) | 6.8 (3.26%) | 10.05 (4.82%) | 1.69 (0.81%) | -4.95 (-2.37%) |
| High-middle SDI | 36.43 (8.95%) | 37.08 (9.11%) | 28.27 (6.95%) | -28.92 (-7.11%) | 18.65 (8.21%) | 20.29 (8.93%) | 19.72 (8.68%) | -21.35 (-9.4%) | 17.77 (9.89%) | 16.68 (9.28%) | 9.19 (5.12%) | -8.1 (-4.51%) |
| Middle SDI | 375.03 (67.3%) | 77.21 (13.86%) | 163.9 (29.41%) | 133.92 (24.03%) | 195.78 (63.82%) | 38.76 (12.64%) | 88.29 (28.78%) | 68.73 (22.41%) | 179.25 (71.57%) | 38.01 (15.18%) | 75.49 (30.14%) | 65.74 (26.25%) |
| Low-middle SDI | 539.93 (92.12%) | 57.99 (9.89%) | 412.41 (70.36%) | 69.54 (11.86%) | 293.89 (78.24%) | 32.69 (8.7%) | 251.28 (66.9%) | 9.92 (2.64%) | 246.04 (116.89%) | 24.28 (11.53%) | 159.99 (76.01%) | 61.77 (29.35%) |
| Low SDI | 258.5 (131.11%) | 0.14 (0.07%) | 258.35 (131.04%) | 0.01 (0%) | 135.29 (113.33%) | -0.39 (-0.32%) | 150.33 (125.93%) | -14.66 (-12.28%) | 123.21 (158.4%) | 0.58 (0.75%) | 107.98 (138.82%) | 14.64 (18.82%) |
| Andean Latin America | 42.11 (67.15%) | 5.52 (8.8%) | 41.59 (66.33%) | -5 (-7.97%) | 24.61 (64.66%) | 3.09 (8.13%) | 26.35 (69.24%) | -4.84 (-12.71%) | 17.5 (71%) | 2.34 (9.51%) | 15.7 (63.69%) | -0.54 (-2.19%) |
| Australasia | 6.17 (172.15%) | 0.29 (8.15%) | 1.12 (31.3%) | 4.75 (132.7%) | 3.61 (167.3%) | 0.15 (6.85%) | 0.65 (30.19%) | 2.81 (130.26%) | 2.56 (179.47%) | 0.14 (9.85%) | 0.47 (32.61%) | 1.95 (137.02%) |
| Caribbean | 16.04 (116.09%) | 1.81 (13.12%) | 4.22 (30.53%) | 10.01 (72.44%) | 7.56 (114.13%) | 0.78 (11.73%) | 2.15 (32.39%) | 4.64 (70.01%) | 8.48 (117.9%) | 1.04 (14.41%) | 2.06 (28.69%) | 5.38 (74.8%) |
| Central Asia | 13.6 (22.93%) | 5.45 (9.19%) | 18.87 (31.8%) | -10.71 (-18.06%) | 11.45 (48.28%) | 2.39 (10.08%) | 8.71 (36.7%) | 0.36 (1.5%) | 2.15 (6.04%) | 3.06 (8.58%) | 10.02 (28.15%) | -10.92 (-30.69%) |
| Central Europe | -27.78 (-45%) | 2.97 (4.82%) | -11.99 (-19.42%) | -18.76 (-30.4%) | -16.33 (-45.86%) | 1.79 (5.02%) | -6.67 (-18.73%) | -11.45 (-32.15%) | -11.45 (-43.83%) | 1.2 (4.58%) | -5.27 (-20.19%) | -7.37 (-28.22%) |
| Central Latin America | 126.32 (135.58%) | 12.44 (13.35%) | 57.71 (61.94%) | 56.17 (60.29%) | 69.39 (159.09%) | 5.66 (12.97%) | 29.47 (67.57%) | 34.26 (78.55%) | 56.93 (114.88%) | 6.81 (13.74%) | 28.25 (57.02%) | 21.86 (44.12%) |
| Central Sub-Saharan Africa | 21.25 (134.94%) | 0.26 (1.68%) | 22.86 (145.17%) | -1.87 (-11.91%) | 12.58 (135.82%) | 0.2 (2.16%) | 13.48 (145.54%) | -1.1 (-11.88%) | 8.67 (133.67%) | 0.07 (1.14%) | 9.38 (144.67%) | -0.79 (-12.15%) |
| East Asia | -21.43 (-10.52%) | 24.9 (12.22%) | -18.28 (-8.97%) | -28.04 (-13.76%) | -11.01 (-9.37%) | 13 (11.06%) | -10.54 (-8.97%) | -13.46 (-11.46%) | -10.42 (-12.08%) | 11.73 (13.61%) | -7.73 (-8.97%) | -14.42 (-16.72%) |
| Eastern Europe | -29.08 (-39.4%) | 4.86 (6.58%) | -13.05 (-17.68%) | -20.89 (-28.3%) | -13.95 (-36.05%) | 2.9 (7.5%) | -6.92 (-17.87%) | -9.94 (-25.68%) | -15.13 (-43.09%) | 1.92 (5.48%) | -6.12 (-17.44%) | -10.93 (-31.14%) |
| Eastern Sub-Saharan Africa | 68.14 (137.89%) | 1.06 (2.15%) | 67.66 (136.92%) | -0.58 (-1.18%) | 37.83 (122.7%) | 0.44 (1.42%) | 41.58 (134.85%) | -4.18 (-13.56%) | 30.31 (163.08%) | 0.62 (3.34%) | 26.37 (141.89%) | 3.32 (17.85%) |
| High-income Asia Pacific | -16.78 (-27.02%) | 3.35 (5.39%) | -13.36 (-21.52%) | -6.76 (-10.89%) | -10.73 (-28.16%) | 2.05 (5.39%) | -7.62 (-20%) | -5.16 (-13.55%) | -6.05 (-25.22%) | 1.32 (5.52%) | -5.58 (-23.26%) | -1.79 (-7.48%) |
| High-income North America | -49.54 (-17.94%) | -5.43 (-1.97%) | 18.14 (6.57%) | -62.25 (-22.55%) | -30.02 (-21.09%) | -2.89 (-2.03%) | 9.63 (6.77%) | -36.77 (-25.83%) | -19.52 (-14.6%) | -2.52 (-1.88%) | 8.51 (6.36%) | -25.51 (-19.08%) |
| North Africa and Middle East | 153.69 (144.44%) | 19.12 (17.97%) | 109.28 (102.7%) | 25.29 (23.77%) | 81.72 (140.65%) | 10.61 (18.25%) | 60.72 (104.51%) | 10.39 (17.89%) | 71.96 (148.99%) | 8.51 (17.63%) | 48.71 (100.86%) | 14.74 (30.51%) |
| Oceania | 19.51 (114.62%) | 0.63 (3.71%) | 18.51 (108.79%) | 0.36 (2.12%) | 11.63 (104.63%) | 0.18 (1.59%) | 11.69 (105.17%) | -0.24 (-2.13%) | 7.87 (133.44%) | 0.48 (8.11%) | 6.77 (114.77%) | 0.62 (10.56%) |
| South Asia | 721.85 (98.57%) | 62.08 (8.48%) | 603.86 (82.46%) | 55.91 (7.63%) | 380.59 (80.33%) | 33.47 (7.06%) | 364.57 (76.95%) | -17.45 (-3.68%) | 341.27 (131.99%) | 27.43 (10.61%) | 236.55 (91.49%) | 77.29 (29.89%) |
| Southeast Asia | 50.09 (72.07%) | 6.9 (9.93%) | 29.61 (42.6%) | 13.58 (19.55%) | 25.88 (63.6%) | 1.81 (4.44%) | 18.17 (44.65%) | 5.9 (14.51%) | 24.21 (84.04%) | 5.26 (18.26%) | 11.77 (40.86%) | 7.18 (24.93%) |
| Southern Latin America | 15.44 (45.66%) | 1.78 (5.25%) | 11.77 (34.8%) | 1.9 (5.61%) | 9.04 (58.73%) | 0.86 (5.59%) | 5.75 (37.34%) | 2.43 (15.8%) | 6.4 (34.75%) | 0.93 (5.02%) | 6.01 (32.61%) | -0.53 (-2.87%) |
| Southern Sub-Saharan Africa | 4.66 (11.7%) | 6.46 (16.25%) | 18.64 (46.85%) | -20.44 (-51.39%) | 5.07 (27.16%) | 3.45 (18.51%) | 10.03 (53.74%) | -8.42 (-45.1%) | -0.41 (-1.95%) | 2.98 (14.12%) | 8.62 (40.8%) | -12.01 (-56.87%) |
| Tropical Latin America | 53.68 (56.94%) | 12.04 (12.77%) | 38.79 (41.14%) | 2.86 (3.03%) | 25.07 (46.3%) | 7.3 (13.49%) | 22.16 (40.92%) | -4.39 (-8.11%) | 28.61 (71.3%) | 4.64 (11.55%) | 16.77 (41.8%) | 7.2 (17.95%) |
| Western Europe | 29.76 (27.12%) | 5.82 (5.3%) | -11.87 (-10.82%) | 35.82 (32.64%) | 16.3 (26.03%) | 3.42 (5.46%) | -6.34 (-10.13%) | 19.22 (30.71%) | 13.47 (28.56%) | 2.33 (4.95%) | -5.45 (-11.55%) | 16.58 (35.16%) |
| Western Sub-Saharan Africa | 30.17 (136.72%) | -0.16 (-0.73%) | 32.52 (147.35%) | -2.18 (-9.89%) | 13.75 (121.58%) | -0.43 (-3.84%) | 15.92 (140.78%) | -1.74 (-15.37%) | 16.42 (152.64%) | 0.31 (2.93%) | 16.59 (154.15%) | -0.48 (-4.44%) |
|  |  |  |  |  |  |  |  |  |  |  |  |  |
